# Gene Expression Profile of the A549 Human Non-Small Cell Lung Carcinoma Cell Line following Treatment with the Seeds of *Descurainia sophia*, a Potential Anticancer Drug

**DOI:** 10.1155/2013/584604

**Published:** 2013-06-27

**Authors:** Bu-Yeo Kim, Jun Lee, Sung Joon Park, Ok-Sun Bang, No Soo Kim

**Affiliations:** KM-Based Herbal Drug Research Group, Herbal Medicine Research Division, Korea Institute of Oriental Medicine, Daejeon 305-811, Republic of Korea

## Abstract

*Descurainia sophia* has been traditionally used in Korean medicine for treatment of diverse diseases and their symptoms, such as cough, asthma, and edema. Our previous results showed that ethanol extract of the seeds of *D. sophia* (EEDS) has a potent cytotoxic effect on human cancer cells. In this study, we reveal the molecular events that are induced by EEDS treatment in A549 human lung cancer cells. The dose-dependent effect of EEDS on gene expression was measured via a microarray analysis. Gene ontology and pathway analyses were performed to identify functional involvement of genes regulated by EEDS. From gene expression analyses, two major dose-dependent patterns were observed after EEDS treatment. One pattern consisted of 1,680 downregulated genes primarily involved in metabolic processes (FDR < 0.01). The second pattern consisted of 1,673 upregulated genes primarily involved in signaling processes (FDR < 0.01). Pathway activity analyses revealed that the metabolism-related pathways and signaling-related pathways were regulated by the EEDS in dose-dependent and reciprocal manners. In conclusion, the identified biphasic regulatory mechanism involving activation of signaling pathways may provide molecular evidence to explain the inhibitory effect of EEDS on A549 cell growth.

## 1. Introduction

Public health statistics indicate that neoplastic disease (commonly referred to as cancer) is a leading cause of death in the Republic of Korea, where more than 142 cancer-related deaths per 100,000 people occurred in 2011 (http://kostat.go.kr). Although a wide-range of anticancer drugs that target cancer-related molecules have been developed, the five-year relative survival rate of cancer patients, especially those with lung cancer, has not improved significantly (http://www.cancerresearchuk.org/cancer-info/cancerstats/survival/common-cancers/). This disappointing clinical outcome may be a consequence of the multifactorial nature of cancer and the acquisition of drug resistance by tumor cells [1, 2]. For these reasons, anticancer chemotherapy is now shifting from mono-substance therapy to combination therapy [3–5]. Extracts of medicinal herbs represent promising sources of novel multi-substance anticancer drugs [3].

*Descurainia sophia* (L.) Webb ex Prantl (Flixweed) is widely distributed in northeastern China and belongs to the family Brassicaceae (Cruciferae). In traditional Korean medicine (KM), the seeds of *D. sophia *have been used for the treatment of diverse diseases and their symptoms, such as cough, asthma, and edema [6]. According to the results of previous studies, *D. sophia* possesses biologically active secondary metabolites, such as cardiac glycosides [7], sulfur glycoside [8], nor-lignan [9], and lactones [10]. In our *in vitro* cytotoxic pre-screening system, the ethanol extract of *D. sophia* seeds (EEDS) displayed potent cytotoxicity against diverse human cancer cells. In addition, cytotoxic (helveticoside) and anti-inflammatory (quercetin and syringaresinol) active constituents were isolated from the EEDS [6]. 

Although the therapeutic constituents we identified in the EEDS have been well-characterized, the diverse composition of herbal extracts makes it difficult to elucidate their exact molecular mechanisms. Moreover, considering that a number of genes regulated by herbal extracts exert combined effects on various biological pathways, it is important to study the effects of herbal extracts at the genomic and molecular levels rather than at the individual gene level. Recent advances in the multi-target/multi-substance therapeutic approach have underscored the importance of using high-throughput analyses to identify the therapeutic mechanisms of complex drugs, such as herbal extracts [11]. Therefore, in the present study, we measured the *in vitro* anti-proliferative effects of the EEDS on human lung cancer cells and developed a gene expression profile using a microarray analysis. Dose-dependent analyses of the microarray data revealed that biological functions associated with signal transduction, such as apoptosis, were significantly elevated after EEDS treatment. 

## 2. Materials and Methods

### 2.1. Plant Materials

The dried seeds of *D. sophia* were purchased from the Kwangmyungdang Medicinal Herbs Co. (Ulsan, Republic of Korea) and identified by Dr. Go Ya Choi, Basic Herbal Medicine Research Group, Herbal Medicine Research Division, Korea Institute of Oriental Medicine, Republic of Korea. A voucher specimen (KIOM-CRC-5) was deposited at the Cancer Research Center, Herbal Medicine Research Division, Korea Institute of Oriental Medicine, Republic of Korea.

### 2.2. Preparation of EEDS

The dried seeds (9.0 kg) of *D. sophia* were ground and extracted by maceration (40 L of 80% EtOH for 48 h, 3 times) at room temperature. The combined extracts were filtered through Whatman filter paper (No. 2, Whatman International, Maidstone, UK) and concentrated using an EYELA rotary evaporation system (20 L, Tokyo Rikakikai, Tokyo, Japan) at 40°C to yield a two-phase extract (766.1 g), which consisted of an upper oil phase and a lower solid phase. The oil phase did not affect the proliferation of A549 cells. Therefore, we did not test the oil phase for further studies. The solid lower extract (535.7 g) was dried in a WiseVen vacuum oven (WOW-70, Daihan Scientific, Seoul, Republic of Korea) at 40°C for 24 h prior to use. The dried solid ethanol extract, that is EEDS, was dissolved in 100% dimethylsulfoxide (DMSO, Sigma, St Louis, MO, USA) at a concentration of 20 mg/mL and stored at −80°C.

### 2.3. Cell Lines and Culture Conditions

All human lung cancer cell lines, including the A549 cells, and the IMR-90 normal lung fibroblast cells used in this study were obtained from the American Type Culture Collection (ATCC, Manassas, VA, USA). All cells, with the exception of IMR-90 (DMEM), were grown in RPMI1640 (Invitrogen, Carlsbad, CA, USA) that had been supplemented with 10% (v/v) fetal bovine serum (FBS) (Invitrogen), 100 U/mL of penicillin, and 100 **μ**g/mL of streptomycin (Invitrogen) in 5% CO_2_ humidified air at 37°C.

### 2.4. Cell Proliferation Assays

Cell viability was quantified in a 96-well tissue culture plate using the Ez-Cytox cell proliferation assay kit (Daeil Lab Service, Seoul, Republic of Korea), as previously described [12]. Briefly, 5 × 10^3^ cells were seeded on culture plates containing 100 **μ**L/well of culture medium. After 24 h, the cells were exposed to various concentrations of the EEDS and maintained for the indicated time periods. The maximum concentration of DMSO vehicle was 0.5% (v/v). Following drug treatment, the cells were washed with phosphate-buffered saline (PBS) to minimize the interference of the EEDS with the Ez-Cytox reaction. Color development in the Ez-Cytox solution by live cells was monitored at 450 nm using the Emax microplate reader (Molecular Devices, Sunnyvale, CA, USA).

### 2.5. Colony Forming Assays

A549 cells were seeded on 6-well culture plates at a density of 200 cells/well and grown for 24 h. The cells were exposed to various concentrations of the EEDS or the vehicle control for 72 h. The culture medium was removed, and after a brief wash with PBS, the cells were grown for 10 days in fresh medium that did not contain the EEDS. After a brief wash with ice-cold PBS, the cells were fixed using an ice-cold neutralized 4% (w/v) paraformaldehyde solution (Biosesang, Seongnam, Republic of Korea) for 10 min. After removing the fixation solution, the colonies were stained with a 0.05% (w/v) crystal violet (Sigma) cell staining solution for 30 min. The free crystal violet solution was removed, and the cells were washed two times with tap water. The stained colonies were photographed, and the number of colonies was manually counted.

### 2.6. FACS Analysis

Apoptotic cell death was determined using the fluorescein isothiocyanate (FITC)-Annexin V apoptosis detection kit (BD Biosciences, San Jose, CA, USA) according to the manufacturer's instruction. In brief, A549 cells were seeded on 6-well culture plates at a density of 2 × 10^5^ cells/well. After 24 h, cells were exposed to EEDS (0 or 20 **μ**g/mL) for the indicated time periods. Cells were harvested, washed two times with ice-cold PBS, and resuspended in 100 **μ**L of 1x binding buffer. Then, 5 **μ**L of FITC-Annexin V and 5 **μ**L of propidium iodide (PI) solution were added to the cells, and the mixture was incubated at room temperature for 15 min in the dark. After addition of 400 **μ**L of 1x binding buffer, the cells were analyzed by flow cytometry (FACSCalibur, BD Biosciences).

### 2.7. Microarray Experiment

Total RNA from A549 cells that had been treated with either the EEDS or the vehicle control was prepared using the Easy-Spin total RNA extraction kit (iNtRON Biotechnology, Seoul, Republic of Korea) in accordance with the manufacturer's instructions. Before performing the microarray experiment, the quality of the isolated total RNA was confirmed by electropherogram. RNA integrity number (RIN) = 9.8–10.0, OD_260/280_ (>2.0), and OD_260/230_ (>2.2) were determined (see Supplementary Figure 1 and Supplementary Table 1 of the Supplementary Material online at http://dx.doi.org/10.1155/2013/584604), as previously described [13], by Genomictree, Inc. (Daejeon, Republic of Korea). Total RNA was amplified and labeled using the Low RNA Input Linear Amplification kit PLUS (Agilent Technologies, Santa Clara, CA, USA) and then hybridized to a microarray (Agilent Human whole genome 44 K, Agilent Technologies) containing approximately 44,000 probes (~21,600 unique genes), in accordance with the manufacturer's instructions. The arrays were scanned using an Agilent DNA Microarray Scanner.

### 2.8. Semiquantitative PCR (qPCR)

Single-stranded cDNA was synthesized from 1 **μ**g of total RNA using the SuperScript III first-strand synthesis system (Invitrogen) according to the manufacturer's instruction. The concentration of cDNA was quantified using the ND-1000 NanoDrop spectrophotometer (Thermo Scientific, Wilmington, DE, USA), and 100 ng of cDNA was used as a template for semi-qPCR reaction. The PCR products were analyzed by 1.5% agarose gel electrophoresis. The information of primer sequences and PCR reaction conditions are summarized in the Supplementary Table 3.

### 2.9. Dose-Dependent Microarray Analyses

The raw signal intensities were obtained using Agilent Feature Extraction Software (Agilent Technologies). Array elements with signal intensities below 1.4-fold of the local background were eliminated, and then the remaining elements were normalized using the quantile method [14]. After averaging the ratio of duplicated spots, the expression ratios were hierarchically clustered using the CLUSTER program (http://rana.lbl.gov/). The short time series expression miner (STEM) program, which was originally developed for the temporal analysis of gene expression [15], was used to identify dose-dependently expressed genes. The statistical significance of the resultant expression pattern was calculated as a false discovery rate (FDR) using 1,000 random permutations.

### 2.10. Public Microarray Dataset

The publically available microarray dataset with accession number of GSE4573, archived in the Gene Expression Omnibus (http://www.ncbi.nlm.nih.gov/geo), was used in the present study [16]. The dataset was composed of 130 squamous lung carcinoma tissues with survival information. We normalized the probe intensities of each array using the quantile method [14]. After averaging multiple probes, genes that were associated with survival were selected using BRB ArrayTools (version 4.2.1, http://linus.nci.nih.gov/BRB-ArrayTools.html), which compute a statistical significance for each gene using a Cox proportional hazard regression model with a univariate permutation test number of 10,000.

### 2.11. Gene Ontology (GO) Analyses

The Functional Annotation Tool of DAVID [17] and the High-Throughput GoMiner algorithm [18] were used for simple and dose-dependent GO analyses, respectively. Only the list of genes was used for DAVID, while both the list of genes and the expression ratios were applied as inputs for the GoMiner. In both cases, the *P* value of each GO-term was calculated using Fisher's exact test. For adjustments of multiple comparisons, the Benjamini-Hochberg procedure was used for DAVID [17]. For the GoMiner analysis, a random sampling-based FDR was calculated from 1,000 iterations. The resultant significant GO-terms from the GO analysis were entered into the REVIGO program to construct a network structure composed of nonredundant subsets of GO terms, where the distance between GO terms was measured based on the semantic similarity [19].

### 2.12. Pathway Analyses

A pathway enrichment analysis based on Fisher's exact test was performed using DAVID [17]. As with GO analysis, significantly enriched pathways were identified from an input list of genes and statistically adjusted using FDR. For a more systematic pathway analysis, we conducted a Signaling Pathway Impact Analysis (SPIA) [20], which identifies pathways relevant to the experimental conditions using a list of differentially expressed genes and their expression ratios combined with signaling pathway topology. By randomly bootstrapping the pathway topology (*n* = 3,000), two statistical measurements, *P*_NDE_ and *P*_PERT_, were calculated, which measure the overrepresentation of input genes in a pathway and the abnormal perturbation of a specific pathway, respectively. The global *P* value (*P*_G_) calculated from *P*_NDE_ and *P*_PERT_ was used as the selection criteria for significant pathways.

The pathway analysis methods outlined above primarily focused on the identification of enriched pathways using differentially expressed genes. We then measured dose-dependent changes in pathway activity by calculating a linear combination of the logarithmic value of the expression of all of the genes in each pathway, with a weight of 1. When the genes acted as repressors, the weight was multiplied by −1. The measured activities were normalized and hierarchically clustered. The statistical significance for each pathway was estimated using the random permutation-based method (*n* = 1, 000) [21] in which the FDR was determined by comparing the activity values with randomly permutated values. Only pathways with an FDR below 0.05 were included in the clustering analysis. The pathway information used in the present study was obtained from the Kyoto Encyclopedia of Genes and Genomes (KEGG, http://www.genome.jp/kegg/) database.

### 2.13. Pathway Similarity Matrix

Pathway similarity was measured based on the number of common genes between pathways. Briefly, a matrix of the number of common genes in distinct pathways was constructed and the relative similarity was measured using the Jaccard algorithm [22] in which the fraction of common genes between two pathways was used to calculate similarity. Therefore, the absence of a common gene in two pathways was not considered in measuring similarity. Finally, the relative similarity matrix was hierarchically clustered, and the pathway activity values obtained by linear combinations of the expression ratio, as described above, were merged into a similarity matrix.

## 3. Results

### 3.1. Cytotoxic Effects of the EEDS on Human Lung Cancer Cells

**Figure 1:**
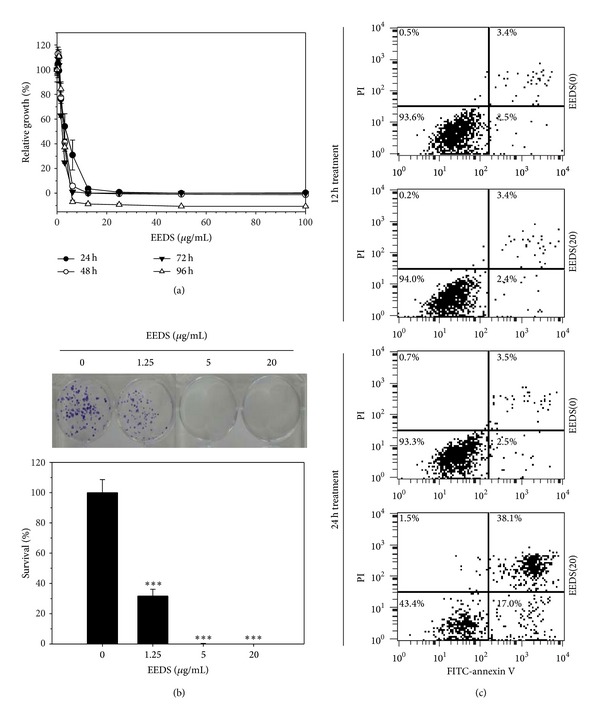
Cytotoxic effect of EEDS on A549 human lung cancer cells. (a) A549 was exposed to increasing concentrations (0–100 **μ**g/mL) of EEDS for various time periods (24–96 h). Cell viability was determined based on mitochondrial enzyme activity as described in the Materials and Methods section. The relative cell growth at each drug dose was calculated by comparison with the vehicle control (0 **μ**g/mL of EEDS, 0.5% DMSO) treatment. (b) A549 cells were exposed to increasing concentrations (0–20 **μ**g/mL) of EEDS for 72 h and then incubated for an additional 10 days in the absence of EEDS (upper panel). Colonies stained with crystal violet were counted and expressed as relative survival (%) compared to the vehicle control (0.5% DMSO) treatment (lower panel). All data are presented as the mean ± S.D. of triplicate experiments. The differences between the vehicle control and treated groups were determined using Student's *t*-test. ***P < 0.001. (c) Representative FITC-Annexin V/PI scatter plots for A549 cells following the EEDS treatment. A549 cells were exposed to EEDS (0 or 20 **μ**g/mL) for 12 h or 24 h, and then subjected to FACS analysis of FITC-Annexin V and PI.

To determine the cytotoxic effects of the EEDS, A549 human lung cancer cells were exposed to increasing concentrations of the EEDS or the vehicle control for indicated time periods. As shown in Figure 1(a), the EEDS efficiently inhibited A549 cell growth in a dose-dependent manner. Three other human lung cancer cell lines (NCI-H23, NCI-H226, and NCI-H460) and the IMR-90 normal human lung fibroblast cell line were also exposed to increasing concentrations of the EEDS for 48 h. The half maximal inhibitory concentrations (IC50s) of the EEDS against different cells lines were calculated and are summarized in Table 1. Among the tested cell lines, A549 (2.81 ± 0.19 **μ**g/mL) cells were the most sensitive, while NCI-H226 (13.18 ± 0.77 **μ**g/mL) and IMR-90 (10.54 ± 0.79 **μ**g/mL) were relatively resistant to EEDS. We also performed colony forming assays to determine whether the EEDS could affect the tumorigenic ability of A549 cells. The results indicated that treatment with 5 and 20 **μ**g/mL of the EEDS for 72 h completely inhibited colony formation from single cells, whereas cells treated with the vehicle control (0 **μ**g/mL of EEDS, 0.5% DMSO) were able to form colonies (Figure 1(b), upper panel). Relatively lower colony numbers were observed in A549 cells treated with a low concentration of the EEDS (1.25 **μ**g/mL) (Figure 1(b), lower panel). In order to elucidate how EEDS can inhibit cell proliferation, we assessed apoptotic cell death of A549 cells following EEDS treatment. Relative to the vehicle control, the percentage of A549 cells undergoing early (Annexin V positive and PI negative) and late (Annexin V positive and PI positive) apoptotic cell death was increased after 24 h treatment of 20 **μ**g/mL of EEDS. Taken together, these data suggest that the EEDS can inhibit cell proliferation and reduce the tumorigenicity of A549 cells through induction of apoptotic cell death. We selected A549 cells that had been treated with the EEDS for further analyses of gene expression profiling.

### 3.2. Gene Expression Profiles

**Figure 2:**
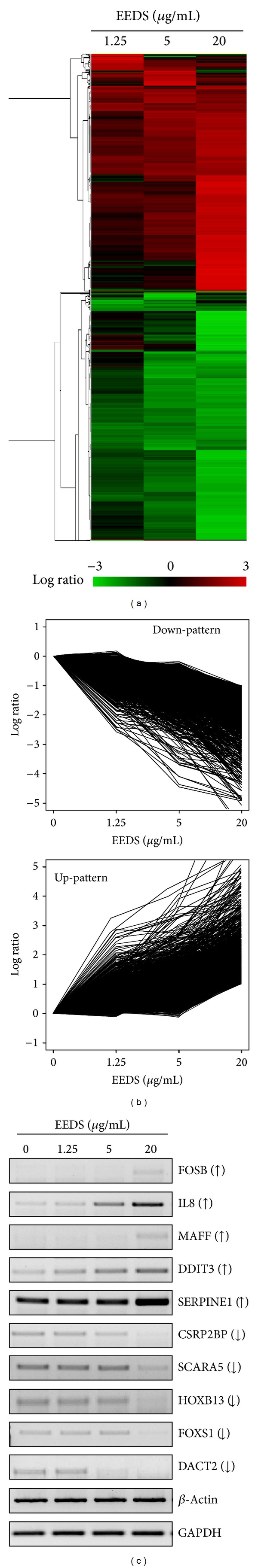
Dose-dependent gene expression by EEDS treatment in A549 cells. (a) Approximately 5,400 differentially expressed genes with a fold ratio greater than 2 or less than 0.5 (for up- and down-regulation, resp.) compared to the vehicle control group in at least one sample were clustered hierarchically. Columns and rows represent individual samples and genes, respectively. The expression ratio color scale ranges from red (high) to green (low), as indicated by the scale bar. Genes exhibiting statistically significant dose-dependent alterations were identified via the STEM program (FDR < 0.001). (b) The Down-pattern is composed of 1,680 genes and the Up-pattern is composed of 1,673 genes. (c) Data acquired from expression chip analysis was confirmed by semi-qPCR. Ten selected genes displaying Up- (↑) and Down- (↓) patterns were amplified using gene specific primers. *β*-actin and GAPDH were used as loading controls.

The overall pattern of gene expression in A549 cells after EEDS treatment is shown in Figure 2(a). Two subgroups of genes that were upregulated and downregulated in dose-dependent manners were identified. To obtain more quantitative analysis, we applied a dose-dependency analysis to the gene expression values. In accordance with the clustering profile of the genes, two significantly different patterns (Down- and Up-patterns) were observed (FDR < 0.001). The Down-pattern consisted of 1,680 genes that were downregulated in a dose-dependent manner, and the Up-pattern consisted of 1,673 genes that were upregulated in a dose-dependent manner. Expression plots of the two patterns are presented in Figure 2(b). The top 20 genes that displayed the greatest amount of variations in each pattern are listed in Table 2. In addition, the expression ratios for all of the genes included in Figure 2(a) are indicated in Supplementary Table 2. The results of the expression chip analysis were validated using semi-qPCR reactions of 10 selected genes displaying UP- and Down-patterns (Figure 2(c)).

### 3.3. Prognostic Implications

We investigated whether dose-dependent alteration in gene expression is implicated in clinical outcomes of lung cancer. First, using publically available lung cancer data (GSE4573), we identified survival-related genes (log-rank *P* value <0.05). We then examined the relationship between these prognostic genes and UP- or Down-pattern genes. As shown in Table 3, 48 Down-pattern and 50 Up-pattern genes were among the survival-related genes identified from lung cancer patients. Among the 48 Down-pattern genes, 32 Down-pattern genes displayed high-hazard ratios (>1), and 16 genes displayed low-hazard ratios (<1). However, there were a greater number of Up-pattern genes with low-hazard ratios (26 genes) than those with high-hazard ratios (24 genes). Although this reciprocal distribution of genes was marginally significant (*P* value of 0.069 in Fisher Exact test), considering that genes with high-hazard ratios were downregulated, while genes with low-hazard ratios were upregulated, we hypothesized that EEDS treatment may enhance antitumorigenic effects.

### 3.4. GO Analysis

To identify the biological function of the two patterns, a GO analysis was performed. The Down-pattern gene set was enriched with metabolic GO terms, including cofactor biosynthesis, heterocycle biosynthesis, and nitrogen compound biosynthesis. In contrast, signaling-related GO terms, including transcription regulation, protein kinase regulation, and apoptosis regulation, were enriched in the Up-pattern gene set. The top 10 statistically significant categories of GO terms (FDR < 0.01) are shown in Table 4 (for the full list of enriched GO terms, please see Supplementary Table 4).

### 3.5. Profiling of GO Terms

**Figure 3:**
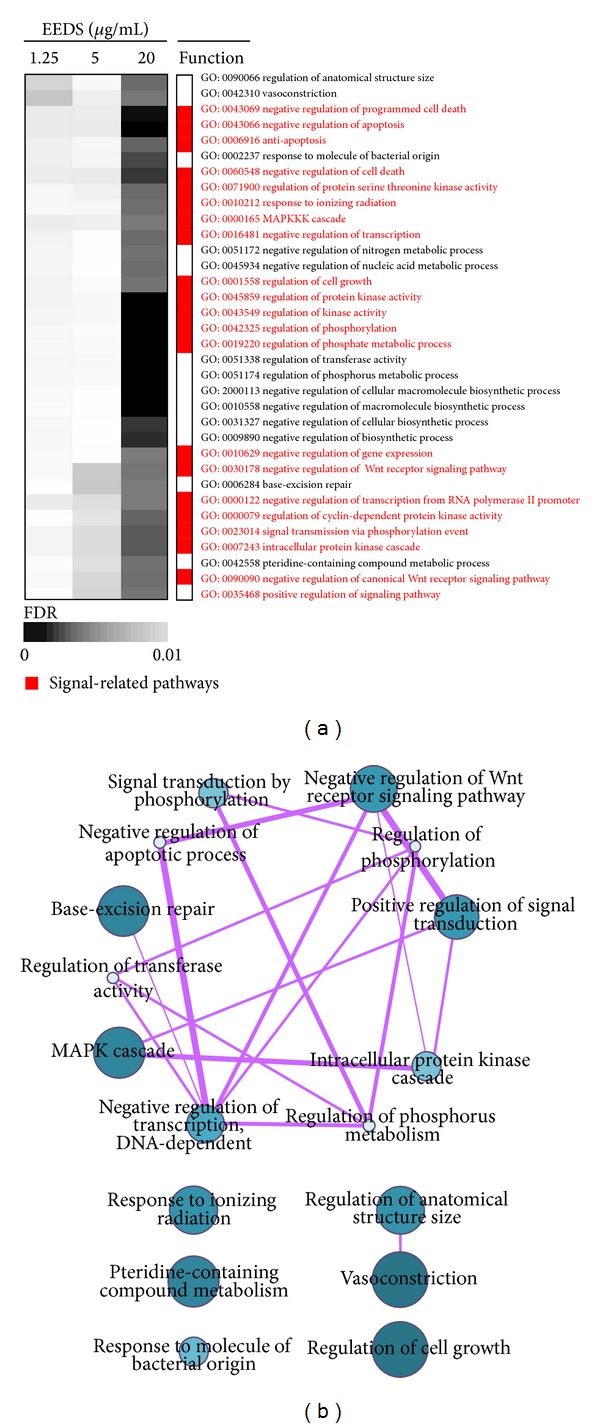
Distribution of GO terms altered by EEDS treatment in A549 cells. (a) GO terms associated with differentially expressed genes that had a fold ratio greater than 2 or less than 0.5 (for up- and down-regulation, resp.) were analyzed at each dose of EEDS using the High-Throughput GoMiner tool. Columns represent individual samples, and rows represents statistically significant GO terms (FDR < 0.01). The positions of signaling-related pathways are colored red. Statistical significance is represented by a gray color gradient, as indicated by the scale bar. (b) A network composed of all nonredundant statistically significant GO terms (FDR < 0.01) after EEDS treatment (20 **μ**g/mL) was constructed using the REVIGO program. The size and color density of each GO term are proportional to its statistical significance, and edge thickness represents the relatedness between two nodes.

The simple GO analysis considered only genes included in the Down- or Up-pattern gene sets. To identify dose-dependent changes in GO terms, all differentially expressed genes were considered in the analysis. As shown in Figure 3(a), most GO terms were altered by treatment with the highest EEDS concentration tested (20 **μ**g/mL; FDR < 0.01). Consistent with the results of the simple GO analysis, the major functions altered were associated with apoptosis and signaling processes.

The enriched GO categories included redundant terms, however, and it was therefore necessary to remove duplicate terms. We used the REVIGO program to obtain nonredundant GO terms (FDR < 0.01) that were altered by EEDS treatment and to measure the functional relationship of these terms in the network structure.  Figure 3(b) shows that signaling-related GO terms, including apoptosis, the MAPK cascade, protein kinase regulation, and phosphorylation regulation, were connected with each other as a cluster, suggesting an interrelationship of biological processes after EEDS treatment in A549 cells.

### 3.6. Pathway Analyses

**Figure 4:**
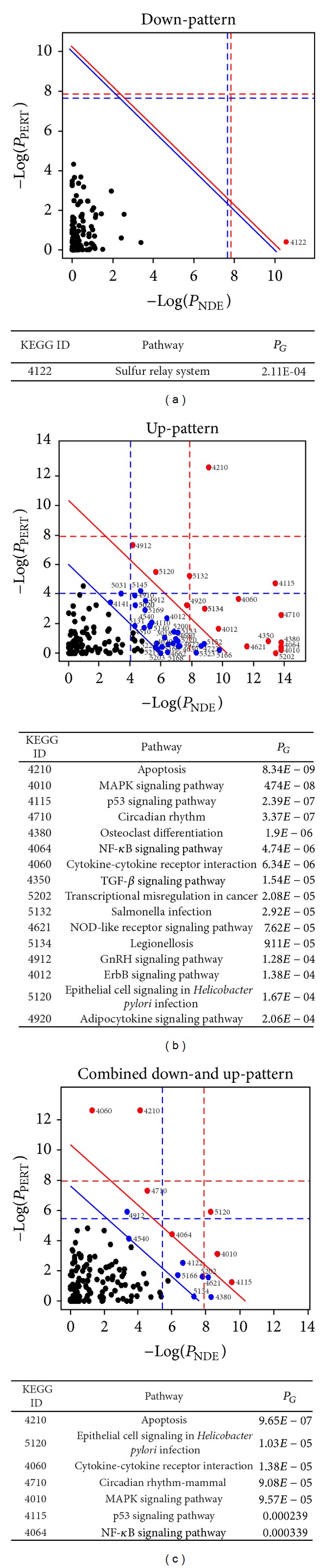
Pathways altered by EEDS treatment in A549 cells. Statistically significant pathways in (a) the Down-pattern, (b) the Up-pattern, and (c) the combined Down- and Up-patterns were analyzed by implementing the SPIA program. The horizontal or vertical axis represents the overrepresentation of a pathway (*P*_NDE_) or the perturbation of a pathway (*P*_PERT_), respectively. Red or blue dotted lines represent the Bonferroni- or FDR- corrected thresholds of significance (1%), respectively, for each axis value. Red and blue circles are significant pathways after Bonferroni and FDR correction (1%) of the global *P* values (*P*_G_), respectively. A list of pathways consisting of red circles (Bonferroni corrected *P*_G_ < 0.01) is shown below.

In addition to the GO analysis, we also examined the functional changes induced by EEDS treatment by performing pathway analyses. Enriched pathways (FDR < 0.01) identified from the Down- and the Up-pattern gene sets are listed in Table 5. Although only the base excision repair pathway (KEGG 03410) and pentose phosphate pathway (KEGG 00030) were significantly enriched in the Down-pattern gene set, signaling-related pathways, including the MAPK pathway (KEGG 4010), the apoptosis pathway (KEGG 4210), the p53 pathway (KEGG 4155), and the TGF-beta pathway, were enriched in the Up-pattern gene set. The list and positions of the Up- or Down-pattern genes in the pathways are depicted in Supplementary Figure 2. For more systematic analyses of the pathways, we conducted SPIA pathway analyses, which calculate a *P* value for a pathway based on random perturbations to the pathway network topology. In the Down-pattern gene set (Figure 4(a)), only the sulfur relay system pathway (KEGG 4211) was significant (*P*_G_ < 0.01), whereas in the Up-pattern gene set (Figure 4(b)), the MAPK pathway (KEGG 4010), the apoptosis pathway (KEGG 4210), the p53 pathway (KEGG 4155), and the TGF-beta pathway were significant (*P*_G_ < 0.01). These results are consistent with those of the simple pathway enrichment analysis (Table 5). We also obtained similarly enriched pathways using the combined Down- and Up-pattern gene sets (Figure 4(c)).

### 3.7. Pathway Activity Analyses

**Figure 5:**
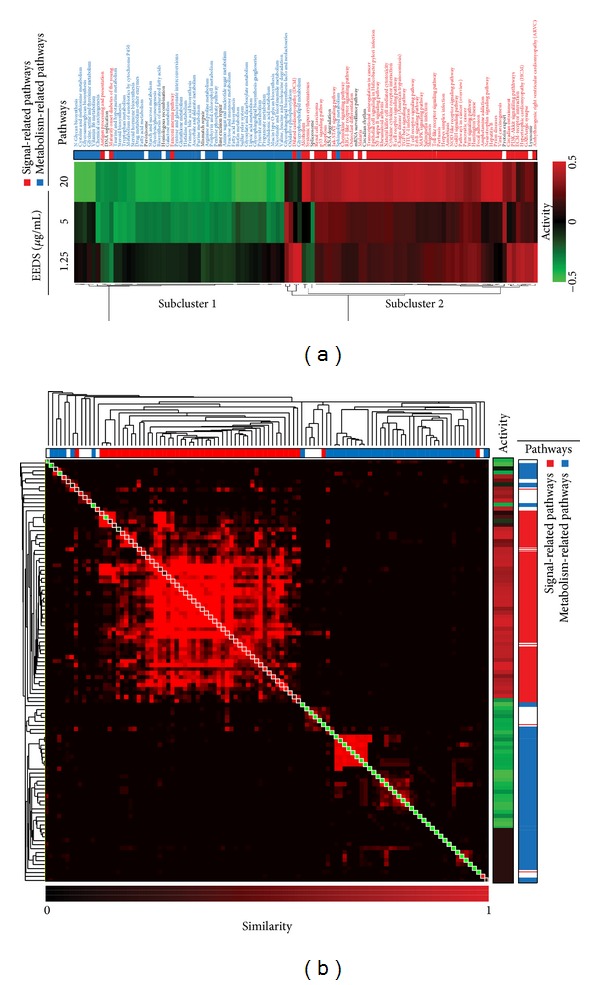
Pathway activities altered by EEDS treatment in A549 cells. (a) The dose-dependent change in pathway activities was measured and clustered hierarchically. Columns represent individual samples, and rows represent activities of pathways. The pathway activity color scale ranges from red (high) to green (low), as indicated by scale bars with arbitrary units. The pathway positions are colored green to indicate metabolic pathways and red to indicate signaling-related pathways. (b) After measurement of the similarity between pathways, the matrix of similarity was clustered hierarchically and merged with activities of pathways. The level of similarity is represented in red with a scale bar. The color in the box of a diagonal line or “Activity” on the right panel represents the activity of the pathway. The positions of signaling-related pathways are colored red and the metabolism-related pathways are colored blue.

Sequential changes in pathway activities based on the EEDS treatment dose were measured using a linear combination of the expression values of all genes in each pathway. Two major subclusters of pathways were grouped based on the statistically significant (FDR < 0.01) similarity of pathway activities (Figure 5(a)). Sub-cluster 1, which is composed of pathways with activities that decreased in a dose-dependent manner, is associated with several metabolism-related pathways. Sub-cluster 2, which is composed of pathways with activities that increased in a dose-dependent manner, is associated with signaling-related pathways as well as immune- and disease-related pathways. To identify the relationship between the statistically significant pathways, we constructed a similarity matrix of pathways based on component genes and pathway activities. As shown in Figure 5(b), subgroups of pathways were clustered based on the similarities of their component genes. Among them, one large subgroup was composed of signaling- and immune-related pathways. The full list of pathways is presented in Supplementary Figure 3. The pathway activities, depicted on a diagonal line or “Activity” on the right panel, indicate that pathways clustered in a subgroup have common levels of activity. For example, the signaling- and immune-related pathways that clustered in the same subgroup show similarly increased activities, suggesting that the interconnection of these diverse pathways may be involved in the response mechanism of A549 cells to EEDS treatment.

## 4. Discussion

Our previous study demonstrated that the EEDS is cytotoxic to human cancer cell lines and that a cardiac glycoside (helveticoside) is an active cytotoxic constituent of the EEDS [6]. In accordance with our previous work, the EEDS significantly inhibited cell growth and tumorigenicity in A549 human non-small cell lung carcinoma cells through induction of apoptotic cell death (Figure 1). Although the major cytotoxic constituent (helveticoside) of the EEDS was previously identified, the cellular mechanism underlying the therapeutic effects of the EEDS was not. One of the main limitations in elucidating the therapeutic mechanism of whole extracts is the complexity of the biological processes affected by the diverse components of extracts. Therefore, it is difficult to reveal the biological pathways associated with herbal drug treatment using a conventional approach based on the analysis of a handful of genes. In general, administration of herbal drugs induces or represses a large number of genes across the whole genome. In the present study, roughly 5,400 genes (approximately 25% of all genes) were found to be differentially expressed following EEDS treatment, as shown in Figure 2(a). Given that treatment with 20 *μ*g/mL of the EEDS significant inhibited cell growth, we also investigated whether the genes regulated by the EEDS are involved in cell growth signaling functions.

Among the differentially expressed genes, two statistically significant patterns of gene expression were observed (Figure 2(b)). The functional segregation of the two gene expression patterns was then validated in two manners. First, we investigated the clinical associations of the two patterns by comparing the gene sets with survival-related genes that were obtained from a public lung cancer dataset. Interestingly, there was a tendency for high-risk genes in lung cancer to be more heavily distributed in the Down-pattern gene set (32 versus 16), while low-risk genes were more heavily distributed in the Up-pattern gene set (26 versus 24), with marginal statistical significance (*P* value of 0.069). Although the number of genes that were common to the Up- or Down-pattern gene sets and survival-related genes was small, our results suggest that the EEDS could be effective in prolonging survival by inhibiting high-risk genes and activating low-risk genes. Second, differences in functional involvement between the Down-pattern and Up-pattern gene sets were measured using GO and pathway analyses. For example, genes grouped in the Down-pattern gene set, which exhibited dose-dependent decrease in expression, were involved in heterogeneous functions, such as metabolic processes or the base excision repair pathway, while genes in the Up-pattern gene set were predominately associated with cell growth signaling functions (Figure 4 and Table 5). Previous reports indicated that energy metabolism involving the pentose phosphate pathway can regulate lung cancer cells [23] and polymorphisms in the base excision repair pathway are related to lung cancer risk [24] and can modulate the effectiveness of chemotherapy in lung cancer patients [25]. Cell growth-related pathways including the MAPK pathway (KEGG 4010), the apoptosis pathway (KEGG 4210), the p53 pathway (KEGG 4155), and the TGF-beta pathway were also significantly enriched in the Up-pattern gene set (*P*_G_ < 0.01), raising the possibility that the EEDS stimulates functionally related biological pathways (Figure 4(b)). The MAPK pathway has been widely reported to be involved in the growth and invasion of lung cancer, and this pathway has been used for the development of anti-lung cancer drugs [26–28]. Activation of the apoptosis and p53 pathways is also one of the main targets of anti-lung cancer drugs, including herbal extracts [29–31]. Other functionally related pathways identified in the Up-pattern gene set included immune system- or infectious disease-related pathways, including cytokine-cytokine receptor interaction (KEGG 04060), NOD-like receptor signaling pathway (KEGG 04621), Helicobacter pylori infection (KEGG 05120), and Salmonella infection (KEGG 05132). Interestingly, immune-system regulation has been reported to improve lung cancer patient outcomes [32, 33]. Similar functional involvement was also observed when we performed a pathway analysis using all of the differentially expressed genes contained in the Down- or the Up-pattern gene sets (Figure 4(c)).

In addition to the Down- and Up-pattern gene sets, we used the expression values from all genes to measure changes in pathway activities. Intriguingly, the results of this analysis clearly demonstrated that metabolism-related pathways and signaling-related pathways were regulated in dose-dependent and reciprocal manners (Figure 5(a)). The activities of a group of metabolism-related pathways were significantly diminished, whereas a group of signaling pathways, including apoptosis and immune-related pathways, were significantly activated in a dose-dependent manner. The regulatory pattern observed in our system is in agreement with the postulated anti-carcinogenic effects of the EEDS, as previously proposed by many reports in which the inhibition of metabolic pathways and the activation of apoptosis or immune-related pathways were the main targets of lung cancer drug development [23, 26, 29, 31, 32]. The observation that the activities of diverse signaling and metabolic pathways appeared in separate clusters implies the existence of a common reciprocal regulatory mechanism. Therefore, we also measured the relationships between pathways based on the similarities of pathway component genes and integrated pathway activities. The results of this analysis indicate that signaling pathways with increased activity are grouped in a large cluster, suggesting that diverse signaling pathways are similarly affected by EEDS treatment (Figure 5(b)). In contrast, the results show that metabolic pathways with decreased activity are grouped in a small cluster. Our results show that the signaling and metabolic pathways were inter-connected through the complex network structure.

Despite these data, the growth inhibitory effect of the EEDS is difficult to explain. To fully elucidate the molecular mechanism underlying the activity of the EEDS, the biological implication of the reciprocal regulation of two biologically distinct groups of pathways must be determined and the exact relationships between the diverse pathways should be verified in more detail. Moreover, further rigorous studies should be done to determine whether the observed reciprocal regulation of biological functions is a general mechanism of herbal extracts. Nonetheless, our present results provide evidence in support of the importance of using whole genome approaches to elucidate pharmaceutical mechanisms. 

## 5. Conclusion

In conclusion, the results of the present study indicate that EEDS treatment induces dose-dependent responses in A549 human non-small cell lung carcinoma cells that involve the up-regulation of a large group of genes associated with cell growth-related signaling pathways and the downregulation of genes associated with metabolic function. This reciprocal regulatory mechanism may provide clues to further our understanding of the mechanism driving growth inhibition in human cancer cells treated with the EEDS, especially in A549 human lung cancer cells.

## Supplementary Material

The integrities of RNA samples used for gene expression analyses were determined by performing electropherogram (Figure S1 and Table S1). The positions of significant genes in each enriched pathways were depicted in Figure S2. The activities of pathways were clustered based on similarity of pathways in Figure S3. Full lists of genes were summarized in Table S2 with expression ratios. Expression of representative genes was confirmed by semiquantitative PCR. The reaction conditions and primer sequences were summarized in Table S3. The enriched GO terms were listed in Table S4.

## Figures and Tables

**Table 1:** IC_50_ of EEDS in human lung cancer cell lines.

Cell lines	NCI60 panel	Disease	Drug expose (h)	IC_50_	
				EEDS (*μ*g/mL)	Dox (*μ*M)^a^
A549	Yes	Lung cancer	24	3.72 ± 1.12	n.d.
			48	2.81 ± 0.19	0.04 ± 0.00
			72	2.15 ± 0.01	n.d.
			96	2.59 ± 0.16	n.d.
NCI-H23	Yes	Lung cancer	48	6.60 ± 0.18	0.22 ± 0.04
NCI-H226	Yes	Lung cancer	48	13.18 ± 0.77	0.33 ± 0.04
NCI-H460	Yes	Lung cancer	48	8.08 ± 0.01	0.31 ± 0.04
IMR-90	—	Normal lung	48	10.54 ± 0.79	0.40 ± 0.04

^a^Dox, doxorubicin, was included as a reference anticancer drug [12].

**Table 2:** Top 20 list of genes mostly down-regulated or up-regulated by EEDS.

Down-pattern				Up-pattern			
Symbol	EEDS (*μ*g/mL)			Symbol	EEDS (*μ*g/mL)		
	1.25	5	20		1.25	5	20
CSRP2BP	−1.35*	−3.05	−6.16	HBEGF	0.86	1.58	4.44
SCARA5	−0.28	−2.58	−5.71	C7orf53	2.86	2.73	4.46
HOXB13	−0.66	−2.42	−5.06	SERPINE1	1.54	3.50	4.59
FOXS1	0.12	−2.40	−4.92	CD274	2.59	3.23	4.60
SNORA12	−2.24	−4.44	−4.91	NOG	1.16	3.91	4.67
RIMBP3	−2.00	−2.74	−4.89	PER1	2.22	1.52	4.68
ST6GAL1	−1.82	−2.20	−4.88	ARC	0.78	1.69	4.79
DACT2	−2.47	−4.08	−4.87	GADD45B	1.23	1.95	4.82
TMEM37	−1.42	−4.37	−4.85	LTB	1.37	4.06	4.85
EEF2K	−1.55	−2.50	−4.84	FOS	1.25	0.52	4.87
GPRIN2	−2.57	−3.41	−4.79	DDIT3	1.66	2.78	4.90
SALL2	−2.08	−4.10	−4.76	C3orf52	1.04	2.26	4.96
CBR3	−1.56	−3.26	−4.57	MAFF	1.62	2.70	5.07
FANCF	−1.30	−1.88	−4.56	IL8	2.94	4.52	5.55
CCR7	−0.58	−1.72	−4.47	PPP1R15A	1.42	2.61	5.59
C5orf58	−0.90	−1.72	−4.38	LOC387763	3.52	4.33	5.67
VAV3	−0.91	−3.48	−4.36	ATF3	1.02	2.49	5.69
C14orf93	−0.64	−2.00	−4.31	EGR2	0.11	0.72	6.46
NTHL1	−0.57	−2.05	−4.31	FOSB	1.36	3.75	7.85
VASH1	−1.31	−3.69	−4.28	EGR1	1.44	4.10	10.02

*Fold induction represents log_2_⁡ expression ratio of gene compared with that of control.

**Table 3:** Clinical association of Down- or Up-pattern genes regulated by the EEDS with survival from lung cancer.

Down-pattern				Up-pattern			
Symbol	Fold induction*	*P* value**	Hazard ratio**	Symbol	Fold induction	*P* value	Hazard ratio
HMBS	−2.06	0.00225	1.41	YPEL5	1.99	0.000577	0.62
GMPPA	−2.80	0.00283	1.46	MNT	1.56	0.00344	1.42
CD79A	−1.27	0.00431	0.72	GGA3	1.11	0.00421	1.40
HSPA12A	−1.18	0.00759	1.43	MGC29506	1.00	0.00471	0.72
RUNX3	−1.19	0.00818	0.71	CHD7	1.63	0.00763	1.49
RBKS	−1.17	0.0123	1.47	ERCC6	1.52	0.00887	1.37
LRRC20	−2.88	0.0132	1.47	SSBP2	1.61	0.0122	0.72
RNF144A	−1.37	0.0136	0.75	NUFIP1	1.41	0.0129	1.33
C11orf60	−1.30	0.0148	1.36	CLK3	1.04	0.0159	1.37
BCS1L	−1.12	0.0149	1.32	PPP1R13L	2.74	0.0160	1.35
CHST12	−2.05	0.0155	0.75	TNFSF9	1.92	0.0162	1.34
CRYBB2P1	−1.09	0.0172	0.75	DDX52	1.80	0.0163	1.36
APITD1	−2.35	0.0176	1.35	FAM108B1	1.33	0.0192	0.73
AP1G2	−1.62	0.0177	1.39	ZMYM5	1.23	0.0203	1.32
ALDH3B1	−1.77	0.0185	1.36	PTPRH	1.89	0.0217	1.34
AUTS2	−1.89	0.0210	0.74	BTG2	3.33	0.0231	0.74
NIPSNAP1	−1.64	0.0229	1.29	ARHGEF15	1.52	0.0233	1.3
LCMT1	−1.69	0.0240	1.29	SPATA2L	1.68	0.0244	1.30
HERC6	−1.18	0.0250	0.75	PRKRIP1	1.56	0.0245	0.76
DYRK4	−1.21	0.0261	0.77	KIFC1	1.47	0.0247	1.32
TPD52L1	−1.30	0.0262	1.35	RUNX1	2.83	0.0247	1.32
VPS33B	−1.05	0.0274	1.32	GOLGA8A	2.02	0.0249	0.72
DHRS1	−1.83	0.0280	1.28	ABL1	1.15	0.0254	0.76
WDR61	−1.26	0.0292	1.32	CRABP2	2.34	0.0258	0.75
PYGL	−1.62	0.0314	1.31	DDIT3	4.91	0.0260	1.34
MKS1	−1.08	0.0316	1.29	IP6K2	2.19	0.0273	0.76
VASH1	−4.29	0.0336	0.77	LOC729806	1.2	0.0275	1.36
MRPL46	−1.52	0.0338	1.29	ELL	2.63	0.0284	0.79
APBA2	−2.56	0.0340	0.75	TRAF4	1.00	0.0294	1.39
LARGE	−2.96	0.0341	0.76	SEMA7A	1.16	0.0297	1.31
IMP3	−1.40	0.0354	1.28	PRNP	1.51	0.0303	0.75
AAAS	−1.55	0.0354	0.78	GADD45A	4.03	0.0314	0.76
TGIF1	−1.41	0.0359	1.26	GLIPR1	1.94	0.0330	0.77
C11orf80	−1.03	0.0363	1.32	PAPOLG	1.93	0.0331	0.75
AGAP11	−1.51	0.0387	1.33	ZNF484	2.81	0.0336	1.36
CCR7	−4.47	0.0390	0.78	RAP2C	1.46	0.0336	0.76
RRBP1	−1.05	0.0395	0.77	MED1	1.25	0.0389	1.24
CDC123	−1.09	0.0422	1.29	HERPUD1	1.01	0.0392	0.79
REEP4	−1.48	0.0433	1.28	DDR2	1.11	0.0404	0.79
PFKFB1	−1.40	0.0433	0.78	PLK4	1.59	0.0417	1.30
ZNF839	−2.28	0.0440	1.3	CDC42SE1	2.29	0.0417	0.76
CCDC53	−1.88	0.0460	0.80	AOC2	1.09	0.0430	1.29
RDX	−1.22	0.0466	1.29	RUNX2	1.80	0.0438	1.29
EIF2B3	−2.05	0.0470	1.27	SFRS12IP1	1.81	0.0451	0.76
PAAF1	−1.81	0.0471	1.35	INPP1	1.40	0.0456	0.76
PITPNC1	−1.39	0.0483	1.29	MCAM	1.57	0.0458	0.79
ATIC	−1.31	0.0492	1.27	LTB	4.85	0.0470	0.77
DDX28	−1.63	0.0498	1.28	ZCCHC10	1.13	0.0480	0.79
				SP2	1.59	0.0489	0.79
				PTHLH	2.81	0.0499	0.79

*Fold induction represents log_2_⁡ expression ratio of gene at treatment with 20 *μ*g/mL of EEDS.

**Log-rank *P* value and hazard ratio were measured in Cox-proportional hazard regression model performed in public lung cancer data [16].

**Table 4:** Top 10 GO terms associated with Up- and Down-patterns by EEDS treatment.

GO ID	GO terms	*P* value*	FDR**
Down-pattern			
GO:0051188	Cofactor biosynthetic process	9.26*E* − 08	2.57*E* − 04
GO:0051186	Cofactor metabolic process	1.79*E* − 07	2.49*E* − 04
GO:0018130	Heterocycle biosynthetic process	8.03*E* − 07	7.42*E* − 04
GO:0044271	Nitrogen compound biosynthetic process	6.15*E* − 06	4.25*E* − 03
GO:0006399	tRNA metabolic process	1.65*E* − 05	9.12*E* − 03
Up-pattern			
GO:0045449	Regulation of transcription	4.21*E* − 26	1.55*E* − 22
GO:0006350	Transcription	3.75*E* − 21	6.91*E* − 18
GO:0006355	Regulation of transcription, DNA-dependent	3.11*E* − 14	3.82*E* − 11
GO:0051252	Regulation of RNA metabolic process	3.78*E* − 14	3.49*E* − 11
GO:0006357	Regulation of transcription from RNA polymerase II promoter	2.05*E* − 13	1.51*E* − 10
GO:0042325	Regulation of phosphorylation	2.03*E* − 11	1.25*E* − 08
GO:0051173	Positive regulation of nitrogen compound metabolic process	2.39*E* − 11	1.26*E* − 08
GO:0045859	Regulation of protein kinase activity	2.44*E* − 11	1.12*E* − 08
GO:0019220	Regulation of phosphate metabolic process	2.68*E* − 11	1.10*E* − 08
GO:0051174	Regulation of phosphorus metabolic process	2.68*E* − 11	1.10*E* − 08

*P values were calculated using Fischer's test.

**FDR corrections were calculated using the Benjamini-Hochberg procedure in DAVID program [17].

**Table 5:** Pathways associated with Up- and Down-patterns by EEDS treatment.

KEGG ID	Pathway	*P* value*	FDR**
Down-pattern			
03410	Base excision repair	3.90*E* − 04	6.66*E* − 03
00030	Pentose phosphate pathway	1.47*E* − 04	1.22*E* − 03
Up-pattern			
04010	MAPK signaling pathway	1.16*E* − 07	1.83*E* − 05
04210	Apoptosis	4.99*E* − 06	3.94*E* − 04
04115	p53 signaling pathway	2.46*E* − 05	1.29*E* − 03
04350	TGF-beta signaling pathway	5.36*E* − 05	2.11*E* − 03
04060	Cytokine-cytokine receptor interaction	1.14*E* − 04	3.60*E* − 03
04710	Circadian rhythm	3.20*E* − 04	8.38*E* − 03
04621	NOD-like receptor signaling pathway	3.39*E* − 04	7.61*E* − 03

*P values were calculated using Fischer's test.

**FDR corrections were calculated using the Benjamini-Hochberg procedure [17].
