# Supplementary material for: Gene Expression Profile of the A549 Human Non-Small Cell Lung Carcinoma Cell Line following Treatment with the Seeds of Descurainia sophia, a Potential Anticancer Drug
Source: Evid Based Complement Alternat Med. 2013 Jun 27;2013:584604. doi: 10.1155/2013/584604 (PMC3712200; doi:10.1155/2013/584604)

Gene expression profile of the A549 human non-small cell lung carcinoma cell line following treatment with the seeds of *Descurainia sophia*, a potential anticancer drug

Bu-Yeo Kim, Jun Lee, Sung Joon Park, Ok-Sun Bang, and No Soo Kim

Supplementary Data

Figure Legends

Supplementary Figure 1. Electropherogram of total RNA samples used for gene expression profiles. The A549 cells were treated with increasing concentrations of EEDS (0-20 μg/mL). After 24 h drug treatment, total RNAs were extracted from the cells, and their integrities were determined by electropherogram. RIN values range from 10 (intact) to 1(totally degraded).

Supplementary Figure 2. Pathways enriched (FDR<0.01) in the Up- and Down-patterns. The genes that are shown in Table 5 and Figure 4, and involved in each pathway are highlighted in green

Supplementary Figure 3. The level of similarity is represented in red with a scale bar. The color in the box of a diagonal line or "Activity" on the right panel represents the activity of the pathway. The positions and names of signaling-related pathways are colored red and the metabolism-related pathways are colored blue.

Supplementary Table 1. Quality control of total RNA samples used for gene expression analysis.

EEDS (μg/mL)	$OD_{260/280}$	$OD_{260/230}$	Ratio (28s/18s)	RIN*	Result
0	2.04	2.22	2.3	9.8	Pass
1.25	2.03	2.28	2.2	10.0	Pass
5	2.03	2.28	2.1	10.0	Pass
20	2.04	2.23	2.0	10.0	Pass

*RIN values, 10 (intact) to 1 (completely degraded).

Supplementary Table 2. Full list of genes regulated by EEDS.
Fold induction represents log2 expression ratio of gene compared with that of control.

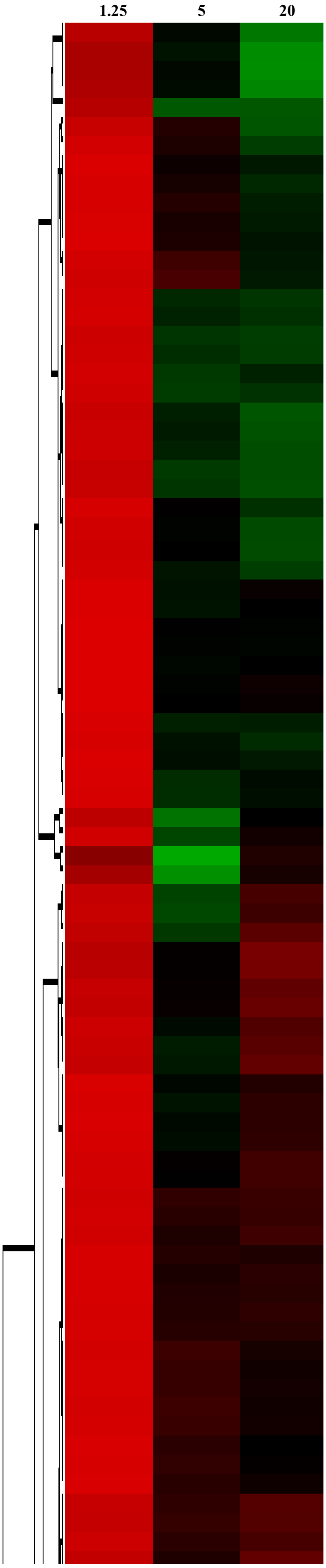

	EEDS (μg/mL)			
**symbol**	**1.25**	**5**	**20**	**Pattern**
CAPS2	1.58	-0.076	-1.03	
GOLIM4	1.033	-0.117	-0.853	
DKFZp667F0711	1.172	-0.06	-0.983	
AGTR1	1.422	-0.095	-1.096	
RORC	1.294	-0.626	-0.624	
LOC644587	1.082	0.205	-0.469	
ITIH4	1.064	0.147	-0.312	
IL11RA	1.007	0.055	-0.111	
C2orf73	1.236	0.129	-0.234	
C3orf42	1.14	0.2	-0.157	
TMF1	1.028	0.118	-0.125	
SYT6	1.384	0.181	-0.126	
SPRR4	1.47	0.445	-0.159	
ITGA10	2.656	0.935	-0.327	
OR1N2	1.909	-0.365	-0.488	
CYP3A5	1.171	-0.192	-0.269	
SLC25A35	1.006	-0.256	-0.303	
RAB17	1.087	-0.236	-0.317	
LEFTY1	1.854	-0.505	-0.298	
HEATR7B1	1.386	-0.402	-0.331	
LOC400756	1.302	-0.198	-0.552	
FAM167B	1.409	-0.185	-0.568	
STAC3	1.285	-0.212	-0.485	
KNG1	1.067	-0.314	-0.418	
IFITM1	1.473	-0.393	-0.58	
C2orf48	1.565	0.021	-0.352	
NOSTRIN	1.518	-0.026	-0.541	
LOC100129311	1.545	0.002	-0.564	
HRCT1	1.577	-0.153	-0.457	
LOC727838	1.297	-0.101	0.058	
BEX4	1.65	-0.148	-0.004	
TRIM42	1.498	-0.007	-0.025	
CYP4F3	1.304	-0.023	-0.032	
LOC100132483	1.292	-0.044	-0.004	
TMEM190	1.211	-0.022	0.078	
LOC646139	2.5	-0.011	0.099	
PHYHIPL	1.449	-0.219	-0.197	
TPPP2	2.148	-0.172	-0.432	
STK38L	1.231	-0.079	-0.146	
LCT	1.055	-0.217	-0.057	
CSMD1	1.229	-0.262	-0.084	
GLRA1	1.03	-0.632	-0.005	
C3orf66	1.765	-0.6	0.153	
ST8SIA2	0.996	-1.226	0.241	
PEG10	1.587	-1.379	0.219	
TPPP3	1.569	-0.534	0.571	
SYT5	1.383	-0.5	0.428	
C4orf51	1.213	-0.352	0.569	
GLS	1.177	0.033	0.771	
FRMD4B	1.778	0.057	1.133	
FLJ42392	1.18	0.037	0.572	
FGFBP1	1.84	0.082	0.997	
WFDC11	1.1	-0.055	0.438	
PAK7	1.206	-0.169	0.533	
FAM18A	1.007	-0.123	0.503	
ACHE	2.703	-0.098	0.437	
SERPINA12	1.264	-0.114	0.268	
SORCS1	1.395	-0.058	0.291	
SLITRK1	1.044	-0.06	0.229	
RIPPLY1	1.657	0.038	0.512	
FLJ39639	1.094	-0.009	0.337	
VENTXP7	1.242	0.293	0.342	
DUSP7	2.134	0.407	0.56	
LOC100128164	1.62	0.224	0.485	
LOC339192	1.515	0.259	0.22	
GVIN1	1.052	0.134	0.209	
ACRC	1.799	0.268	0.327	
CTSG	1.212	0.202	0.268	
CLDND2	1.594	0.291	0.298	
OAF	1.289	0.37	0.146	
TRIM5	1.037	0.268	0.086	
LOC100129559	1.768	0.449	0.17	
RESP18	1.695	0.485	0.136	
LOC154761	1.721	0.47	0.153	
LOC399715	1.004	0.2	0	
DEFB127	1.344	0.307	0.034	
SLC2A13	1.246	0.23	0.091	
LOC100130778	1.598	0.378	0.692	
C11orf9	1.092	0.298	0.457	
SMR3B	1.051	0.221	0.37	
RNASE3	1.055	0.172	0.534	

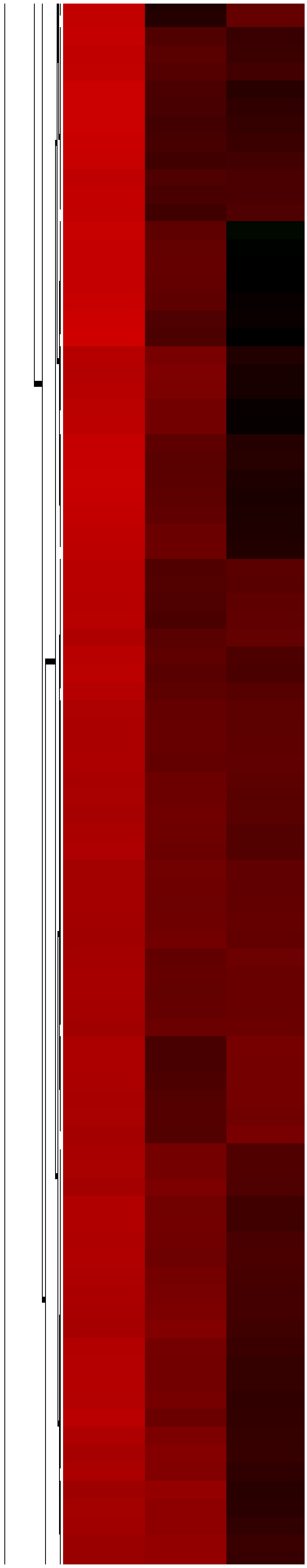

LOC100129081	1.022	0.194	0.533
COL5A2	1.028	0.424	0.297
MAP9	1.167	0.543	0.357
ASPM	1.02	0.445	0.35
FIGF	1.874	0.687	0.381
CYP3A7	1.288	0.458	0.306
LOC100132111	1.177	0.392	0.312
FLJ44342	1.068	0.383	0.311
LOC441294	1.726	0.568	0.582
OR5I1	1.159	0.48	0.451
ENTPD1	1.949	0.723	0.75
CEP152	1.314	0.436	0.537
COPG2IT1	1.251	0.587	-0.054
CECR4	1.933	0.974	-0.026
MCTS1	1.108	0.566	0.006
C1orf213	1.277	0.637	0.011
DKFZp434J0226	1.297	0.619	0.056
TNFSF15	1.852	0.716	0.09
TMEM133	1.195	0.44	0.005
C8orf4	1.044	0.687	0.191
LOC645722	1.212	0.858	0.168
KRTCAP3	1.152	0.778	0.142
LOC729570	1.419	0.876	0.066
CEP290	2.084	1.273	0.075
ZNF783	1.055	0.506	0.199
PPP1R1B	1.216	0.55	0.241
LOC728175	1.048	0.476	0.163
HEMGN	1.942	0.908	0.267
DSCAML1	1.066	0.533	0.162
GSDMB	1.521	0.858	0.251
EDEM3	1.468	0.845	0.273
EP400NL	1.164	0.515	0.574
CCL26	1.15	0.524	0.549
PAQR6	1.419	0.622	0.742
C13orf18	1.092	0.448	0.575
ARHGAP20	1.082	0.55	0.613
HS3ST6	1.367	0.703	0.565
FCGR2C	1.082	0.508	0.432
CR1	1.528	0.793	0.735
SMC6	1.383	0.799	0.733
PCYT1B	1.242	0.743	0.676
LOC100130387	2.04	1.213	1.118
FLJ45983	1.416	0.808	0.797
SPAM1	1.543	0.99	0.85
CCDC146	1.229	0.788	0.651
CPEB4	1.129	0.759	0.606
LOC389493	1.098	0.709	0.53
FAM174A	1.207	0.734	0.574
FBLN2	1.037	0.721	0.61
SEMA3A	1.37	0.918	0.811
KIAA1109	1.154	0.772	0.692
FYB	1.878	1.28	1.177
C16orf79	1.96	1.415	1.201
RBM44	1.453	0.869	0.963
LOC729078	1.008	0.607	0.63
LOC285000	1.083	0.647	0.684
CENPJ	1.078	0.657	0.682
ANKRD13C	1.071	0.71	0.707
GOLGA2LY1	1.469	0.623	1.017
CMTM2	1.091	0.466	0.732
LOC730045	1.11	0.508	0.765
LGI4	1.69	0.84	1.168
GPC6	1.108	0.549	0.737
PTGS2	1.257	0.635	0.921
WDR72	1.123	0.786	0.542
LARP7	1.039	0.713	0.498
CYP27C1	1.207	0.913	0.582
FOXC1	1.097	0.701	0.397
EPM2AIP1	1.111	0.72	0.396
SEC31B	2.224	1.431	0.938
AHSA2	2.09	1.294	0.897
JMJD7-PLA2G4B	1.268	0.855	0.521
LOC100130128	1.126	0.799	0.45
AADAC	1.263	0.945	0.513
SLC4A7	1.066	0.835	0.409
AQP7P3	1.576	1.035	0.516
SLCO4A1	1.213	0.781	0.362
GABRE	2.198	1.387	0.644
PP14571	1.772	1.173	0.483
LEPR	1.272	0.731	0.35
DYNLT3	1.016	0.723	0.31
NTS	1.202	0.946	0.398
CCDC88A	1.152	0.879	0.323
MGAT4A	1.441	1.346	0.369
LOC401097	1.447	1.259	0.381
ANKRA2	1.441	1.27	0.441
TTC32	1.632	1.554	0.621
KCNJ11	1.492	1.444	0.528

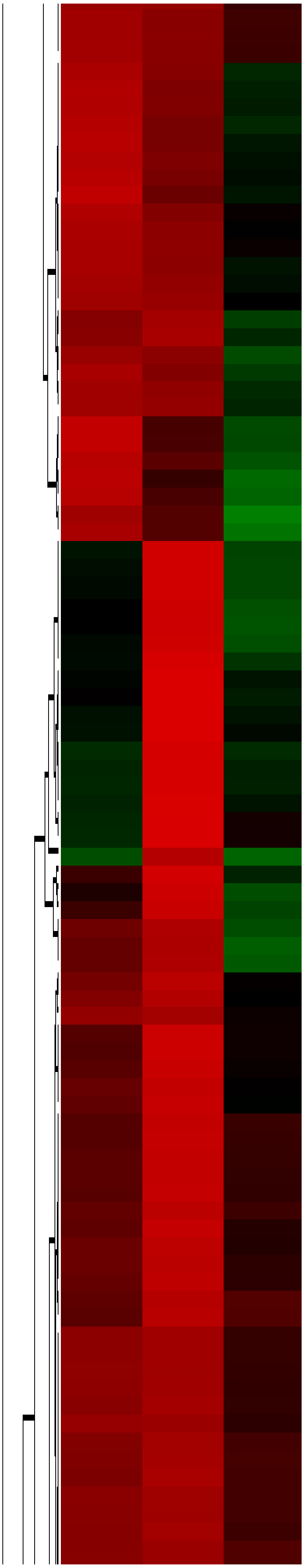

LIX1	1.32	1.128	0.521
CCDC68	2.178	1.877	0.823
ARSI	1.286	1.088	0.461
APOL6	1.173	0.929	-0.269
CCDC18	1.06	0.751	-0.186
C6orf26	1.95	1.41	-0.298
C16orf73	1.473	0.968	-0.321
MGEA5	1.048	0.68	-0.127
CCPG1	1.342	0.947	-0.121
CCDC46	1.338	0.882	-0.093
IFT74	1.265	0.703	-0.144
EGFL8	2.067	1.529	0.101
SLC26A2	1.062	0.877	-0.009
LAMB2L	1.211	1.024	0.078
POL3S	1.193	1.003	-0.132
CRADD	1.128	1.015	-0.092
LOC388279	1.878	1.792	0.002
HOXB9	0.859	1.057	-0.401
FOXL1	1.428	1.763	-0.399
PTP4A3	1.046	0.948	-0.517
FLJ45244	2.048	1.585	-0.715
ZFYVE28	1.106	1.007	-0.287
PPIL6	1.256	1.176	-0.279
LRRC32	1.237	0.444	-0.474
GRAMD4	1.085	0.402	-0.4
TBX18	1.55	0.762	-0.708
LOC100129354	1.027	0.296	-0.585
IL15	1.167	0.46	-0.64
LOC100128242	1.631	0.853	-1.3
ETAA1	1.114	0.552	-0.763
PCDH21	-0.149	1.706	-0.538
OR2G3	-0.074	1.135	-0.379
GPR112	-0.044	1.02	-0.336
NCRNA00119	0.006	1.036	-0.408
CD1C	0.004	1.334	-0.551
CILP	-0.066	1.427	-0.544
INSC	-0.049	1.005	-0.239
LOC100130850	-0.04	1.565	-0.135
AAA1	0.01	1.006	-0.133
UNQ9368	-0.096	1.24	-0.105
JPH1	-0.113	1.421	-0.053
LOC100129711	-0.393	1.927	-0.397
MAPK15	-0.189	1.119	-0.159
LOC100129162	-0.185	1.046	-0.159
SLC37A1	-0.161	1.027	-0.089
SULT1C3	-0.338	1.918	0.194
LOC100290415	-0.235	1.254	0.119
LOC100293499	-0.618	1.438	-0.797
DAD1L	0.448	1.601	-0.26
LOC283692	0.188	1.285	-0.485
DCAF4L1	0.421	1.424	-0.47
LOC148189	0.958	1.49	-0.661
LOC284080	0.644	1.1	-0.602
GPT2	1.018	1.754	-0.895
DKFZp686A1627	0.838	1.343	0.034
CHAC1	2.351	3.203	0.026
DMRTA1	2.097	2.331	0.197
USP51	0.693	1.665	0.121
FUT1	1.434	3.636	0.254
PRG2	0.673	1.527	0.073
LOC284561	0.946	1.781	0.022
FOXQ1	0.711	1.447	-0.017
FLNB	0.498	1.161	0.342
C6orf140	0.452	1.039	0.288
SLC1A4	1.195	2.545	0.696
SLC1A5	0.879	1.893	0.48
PSAT1	0.917	2.067	0.507
ABCG1	1.175	2.233	0.728
LOC283713	0.547	1.138	0.212
DRAM2	0.578	1.02	0.192
RPL23AP64	0.592	1.038	0.253
OR52E2	0.896	1.704	0.4
SERPINE2	0.615	1.168	0.531
ITGA3	0.603	1.249	0.549
PKD1L2	1.662	1.907	0.62
LYRM5	1.043	1.2	0.386
LOC158402	1.304	1.445	0.467
FLJ35024	1.402	1.59	0.48
FLJ43663	1.632	1.962	0.585
MUSTN1	1.103	1.142	0.325
KRTAP20-3	0.881	1.095	0.448
KIRREL3	0.868	1.112	0.468
TMEM91	1.13	1.526	0.599
SLC35A1	0.908	1.048	0.436
GUCY1B2	1.091	1.259	0.533
RAET1E	1.046	1.271	0.474
MYH3	1.012	1.163	0.615
C21orf111	0.941	1.07	0.546

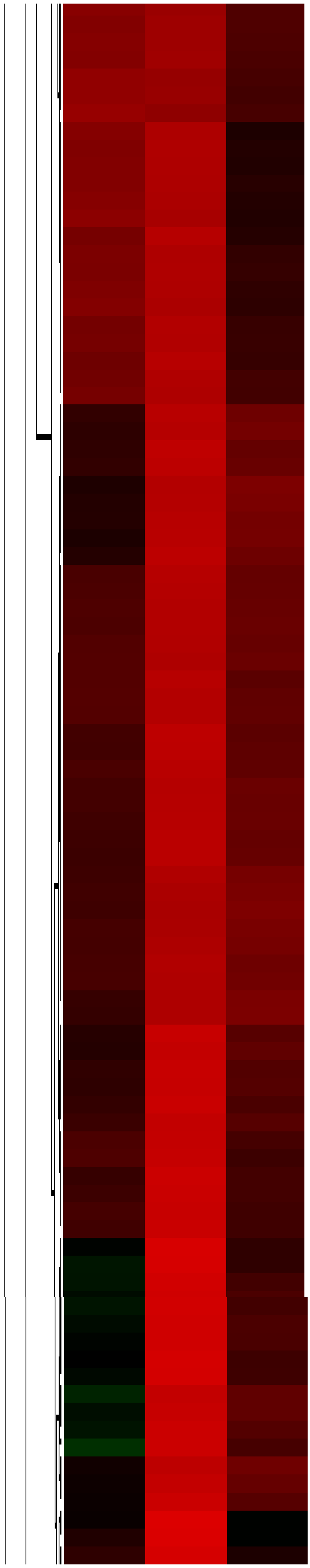

hCG_2045830	1.283	1.557	0.783
C5orf28	0.845	1.004	0.494
LOC728510	0.853	1.041	0.479
SIP1	0.998	1.028	0.482
NEDD4	1.066	1.115	0.491
AP1AR	1.281	1.209	0.604
ADRBK2	0.946	1.233	0.206
LOC283501	0.992	1.35	0.277
C20orf70	0.821	1.099	0.212
HPVC1	0.751	1	0.231
MID1IP1	0.869	1.111	0.227
LOC100128086	1.039	1.239	0.248
CEBPG	0.85	1.311	0.266
C9orf167	0.827	1.16	0.335
C8ORFK29	1.039	1.475	0.454
CLN8	1.309	1.791	0.493
ARL6IP6	0.872	1.144	0.299
TRIB3	1.246	1.91	0.594
TPBG	0.719	1.081	0.342
LRP11	0.635	1.054	0.316
LOC57399	0.859	1.333	0.506
ITGA2	1.566	2.309	0.877
LOC644662	0.463	1.694	1.011
ABCC3	0.274	1.108	0.712
FBXO17	0.261	1.049	0.549
BCAT1	0.574	2.123	1.175
SFT2D1	0.193	1.124	0.786
PIGL	0.195	1.005	0.682
PTH	0.249	1.247	0.792
ATP2A3	0.217	1.412	0.899
SLC43A1	0.288	1.498	0.875
ZMAT3	0.573	1.427	0.79
HYI	0.523	1.247	0.702
MAF	1.259	2.881	1.677
BMPER	0.621	1.45	0.856
UHRF1BP1	0.608	1.305	0.755
EXPH5	0.542	1.141	0.697
MT2A	0.499	1.097	0.54
XYLT1	0.56	1.192	0.641
STC2	1.942	4.175	2.263
WNT16	0.537	1.526	0.732
C19orf71	0.515	1.472	0.735
LOC285216	0.497	1.241	0.64
TLR4	0.416	1.124	0.657
ANAPC13	0.449	1.221	0.698
FLJ43315	1.119	3.208	1.847
IL18BP	0.444	1.342	0.727
CDS1	0.342	1.078	0.59
GALC	0.363	1.038	0.696
KRTAP23-1	0.38	1.015	0.724
ASNS	1.26	3.401	2.546
LOC100131397	0.428	1.058	0.754
C12orf39	1.497	3.796	2.58
KRTAP1-5	0.46	1.16	0.723
ANXA3	0.554	1.361	0.885
MYO15A	0.373	1.206	0.856
MMP10	1.042	3.496	2.488
HKDC1	0.244	1.276	0.558
ENC1	0.372	1.984	0.979
SLC2A7	0.381	1.57	0.65
SCAND3	0.289	1.236	0.522
OR52N2	0.428	1.683	0.625
CALB2	0.616	2.036	0.911
LOC100132338	0.769	1.981	0.71
EIF4EBP1	0.44	1.112	0.34
PCK2	0.349	1.297	0.429
KCNG1	0.633	2.116	0.707
COL13A1	0.453	1.299	0.414
C15orf17	0.516	1.618	0.51
TYR	-0.02	1.078	0.238
ASB15	-0.15	1.496	0.328
PYCR1	-0.105	1.093	0.348
LOC729187	-0.065	1.177	0.43
ABHD10	-0.041	1.908	0.682
RP11-374F3.4	-0.001	1.922	0.561
LOC100132893	-0.059	1.434	0.425
ENPP2	-0.253	1.393	0.685
KLHL10	-0.068	1.02	0.494
CD52	-0.094	1	0.411
GIPC2	-0.437	1.868	0.649
NDUFA4L2	0.132	1.439	0.85
ABCA6	0.071	1.031	0.536
FAM19A1	0.082	1.388	0.59
GPA33	0.063	1.437	-0.015
CYYR1	0.17	1.155	-0.013
KCNJ2	0.305	1.402	0.188
LPO	0.498	1.794	0.247
DKFZp686K1684	0.316	1.205	0.211

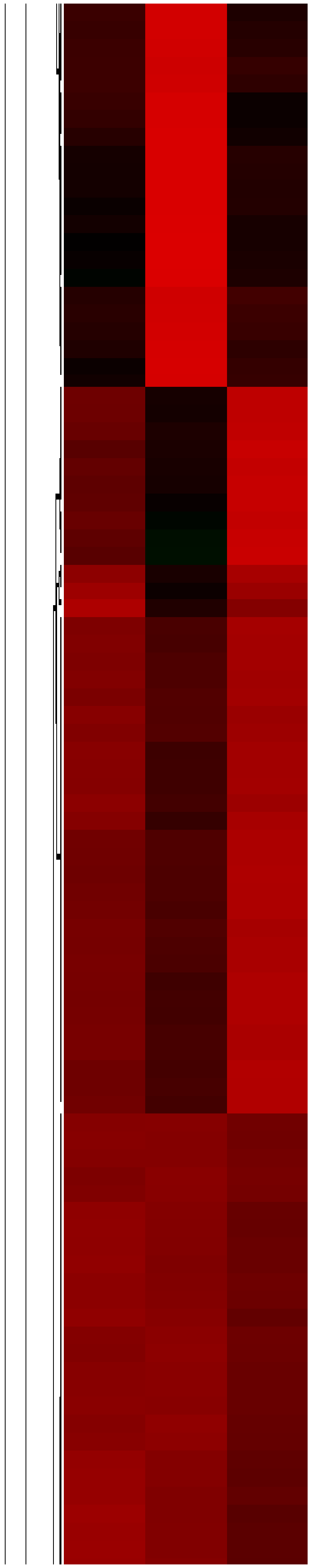

AARS	0.286	1.002	0.194	
LOC100132829	0.476	1.617	0.417	
FLJ16341	0.35	1.19	0.267	
SLC6A9	0.422	1.581	0.087	
C1orf210	0.26	1.07	0.058	
APOL3	0.184	1.012	0.08	
ALDH1L2	0.187	1.842	0.331	
LOC201651	0.094	1.009	0.172	
AKNA	0.099	1.035	0.167	
HMGA2	0.056	1.113	0.184	
MYOZ1	0.115	1.271	0.136	
LOC283867	0.017	1.433	0.153	
C1orf120	0.074	1.879	0.229	
TTC23L	-0.022	1.08	0.146	
OR7G3	0.399	2.203	0.708	
SLC45A3	0.253	1.281	0.366	
PHGDH	0.356	1.99	0.532	
PRF1	0.336	2.291	0.491	
LOC100128571	0.076	1.357	0.335	
CBS	0.164	2.054	0.529	
C8orf22	0.796	0.164	1.373	
C5orf22	0.621	0.114	1.087	
ZC3H12A	1.146	0.323	2.101	
TRPM7	0.755	0.235	1.716	
TBX3	0.73	0.179	1.434	
CTAG1A	0.678	0.159	1.416	
NOTUM	0.583	0.051	1.196	
KCNQ1OT1	0.797	-0.051	1.484	
NFKBIA	0.559	-0.087	1.179	
FLJ33065	0.446	-0.08	1.012	
NME5	0.937	0.166	1.11	
MOXD1	1.145	0.091	1.122	
ERLIN1	1.071	0.201	0.814	
SMC4	0.867	0.505	1.141	
DIRAS3	0.936	0.519	1.181	
KIF14	0.859	0.526	1.116	
CCL2	1.566	0.932	1.933	
ATAD2	0.795	0.532	1.045	
CENPF	0.991	0.593	1.124	
C1orf27	1.134	0.734	1.378	
C20orf107	1.125	0.507	1.338	
ZNF615	0.993	0.47	1.2	
DKFZp686M1136	1.456	0.697	1.789	
ESCO1	0.971	0.466	1.078	
PER2	1.349	0.537	1.692	
TRIP11	0.802	0.562	1.218	UP
CSPP1	0.779	0.552	1.19	UP
SCML1	0.979	0.691	1.549	UP
MYBL1	0.788	0.537	1.203	UP
DNTTIP2	0.748	0.477	1.128	UP
SNAPC1	0.83	0.565	1.16	UP
TRIB1	0.855	0.563	1.223	UP
C14orf106	0.967	0.607	1.358	
LRRC69	0.807	0.43	1.171	
SBNO1	0.757	0.438	1.128	
HAT1	0.687	0.387	1.015	
CEP97	1.114	0.66	1.583	
C7orf63	1.075	0.642	1.522	
C10orf118	1.638	1.012	2.636	UP
ARGLU1	1.021	0.66	1.646	UP
GOLGA8A	1.29	0.767	2.018	UP
STXBP3	1	1.003	0.848	
CLIP1	1.469	1.438	1.221	
NPFF	1.101	1.099	0.97	
PSG5	0.953	1.045	0.905	
LOC440995	0.952	1.008	0.868	
TCP11L2	2.342	2.166	1.688	
CEP70	1.619	1.478	1.148	
VAMP4	1.448	1.315	1.065	
LYG1	2.026	1.778	1.465	
LOC439911	1.134	1.049	0.896	
ACYP1	1.552	1.446	1.199	
CAPZA3	1.04	0.975	0.71	
LOC390595	2.308	2.431	1.915	
CXCL5	1.411	1.53	1.171	
DRD4	1.472	1.503	1.14	
CDKL3	1.277	1.293	0.975	
ANGPTL4	1.733	1.703	1.309	
RFK	1.053	1.138	0.799	
FBXW10	1.627	1.682	1.197	
SCLT1	1.866	1.673	1.206	
AGER	1.331	1.176	0.825	
CD70	1.031	0.876	0.672	
GLRA3	1.049	0.858	0.594	
UCN	1.099	0.926	0.655	
FLJ25006	1.606	1.345	0.95	
INTS2	1.107	0.959	0.654	
ZBTB41	1.13	1.095	0.719	

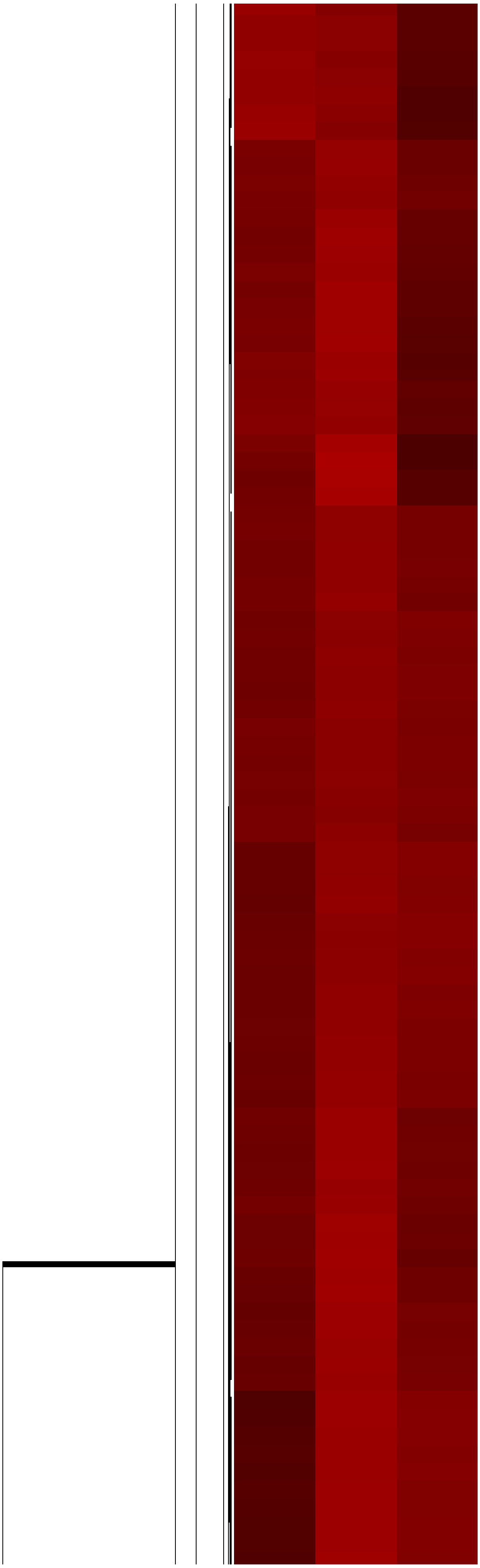

MBNL2	1.836	1.767	1.151
POLI	1.346	1.219	0.797
CYorf15B	1.662	1.571	0.992
NAALAD2	1.009	0.975	0.55
HINT3	1.089	0.986	0.573
CDH19	1.033	0.889	0.556
NCRNA00204B	1.106	1.354	0.963
MIMT1	0.879	1.088	0.779
UBL3	0.847	1.007	0.76
C20orf144	0.878	1.051	0.827
NCRNA00081	1.288	1.684	1.111
IL17B	0.979	1.353	0.871
BCAP29	0.894	1.201	0.765
C1GALT1C1	0.913	1.145	0.732
TUBE1	1.818	2.515	1.464
RHOF	0.885	1.172	0.692
NLRC3	0.802	1.045	0.6
MTHFD2	1.291	1.721	0.951
WNT4	1.061	1.275	0.713
CHST7	1.007	1.255	0.713
PSORS1C3	2.384	2.791	1.831
ZNF506	0.915	1.041	0.665
TNPO1	1.442	1.59	1.059
PDIK1L	0.775	1.041	0.489
ARL6	0.758	1.105	0.503
PYROXD1	0.755	1.147	0.595
NT5E	1.129	1.628	0.847
SCG5	1.736	2.12	1.75
PAQR5	1.37	1.662	1.365
OR8H3	0.879	1.107	0.899
CMPK1	0.857	1.086	0.898
STAMBPL1	1.149	1.44	1.175
SNHG8	0.978	1.247	0.967
DKFZp686L14188	2.238	2.77	2.548
C5orf44	1.115	1.348	1.244
CG030	0.885	1.121	0.99
ANAPC10	0.852	1.07	0.955
SLC36A4	1.253	1.579	1.432
SDCCAG10	0.865	1.079	0.93
CD97	0.9	1.026	0.912
TWSG1	0.971	1.13	1.014
CDSN	0.934	1.102	0.991
C6orf174	1.038	1.216	1.074
GKN2	1.037	1.208	1.117
CSAG3	1.07	1.194	1.109
LOC728769	1.079	1.257	1.069
LOC284288	1.203	1.677	1.556
BCL2A1	1.204	1.695	1.566
MPP3	1.122	1.591	1.415
IKBIP	1.067	1.55	1.383
HIST3H2A	1.272	1.672	1.624
C12orf35	1.324	1.72	1.679
STX16	0.99	1.286	1.207
AMN1	1.613	2.103	1.98
LOC730441	0.772	1.061	0.924
HSPA13	1.138	1.541	1.364
FAM72D	0.797	1.054	0.908
EPN2	0.831	1.123	0.951
LOC92249	1.181	1.613	1.387
OXR1	0.811	1.112	0.917
OR7C1	0.83	1.187	1.002
PCMTD1	0.779	1.072	0.759
INPP4A	0.896	1.262	0.913
EDEM1	1.154	1.65	1.206
ULK1	1.156	1.647	1.17
CD68	0.82	1.121	0.855
C2orf82	1.301	1.705	1.237
TM2D1	0.929	1.36	0.903
KCTD16	0.894	1.299	0.885
LOC646736	1.144	1.649	1.046
RAP1GAP2	0.671	1.007	0.71
C9orf40	0.731	1.135	0.782
PLAU	1.178	1.842	1.402
CCBL1	1.072	1.626	1.207
TBRG1	0.739	1.066	0.822
C18orf32	0.713	1.079	0.837
BGLAP	0.904	1.316	1.039
VAV1	1.007	1.994	1.679
GARS	0.732	1.424	1.217
RSL24D1	0.859	1.555	1.341
FOLH1B	0.685	1.231	1.046
TMEM216	0.556	1.023	0.884
RANGRF	0.654	1.178	0.976
ULBP1	2.274	4.235	3.488
PTCHD2	0.7	1.315	1.1
GREM2	0.544	1.024	0.846
PODXL	1.045	2.083	1.696
LOC100130721	0.821	1.669	1.351

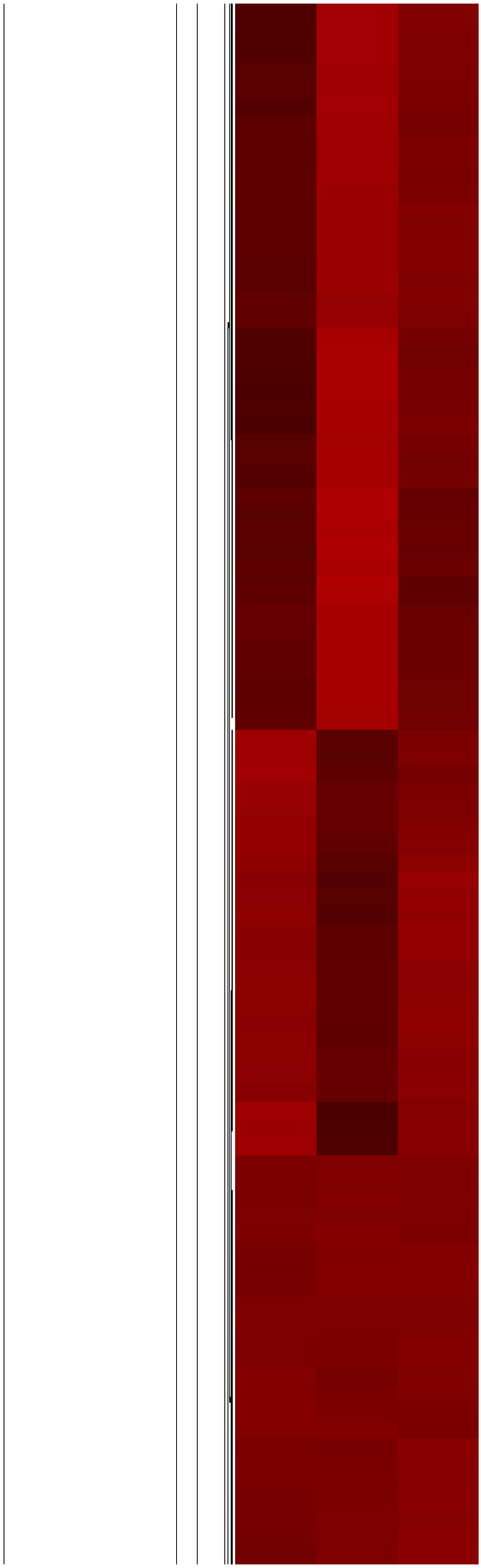

ANKRD20B	0.725	1.477	1.16	
ADAM19	0.549	1.101	0.88	
PRKCE	0.954	1.757	1.4	
LOC100289026	1.275	2.242	1.763	
AP1S2	0.713	1.364	1.023	
DVL1	0.712	1.187	0.917	
WNT7A	0.667	1.112	0.879	
ACCN3	1.265	2.127	1.695	
RHCG	1.237	2.018	1.586	
COL7A1	0.916	1.491	1.193	
SPANXB2	0.7	1.162	0.988	
LOC391081	0.753	1.235	1.053	
ORMDL1	0.818	1.354	1.155	
DPY19L1	0.647	1.098	0.933	
SLC2A1	0.685	1.158	0.963	
SPCS3	0.82	1.24	1.079	
PCDH1	0.811	1.254	1.044	
ZNF773	0.966	2.014	1.403	
SLC22A4	0.596	1.24	0.829	
CARS	0.541	1.204	0.833	
BCL2L2	0.496	1.145	0.784	
TMEM158	1.11	2.358	1.704	
SLC38A1	0.658	1.457	1.074	
VLDLR	0.915	1.672	1.207	
LSM5	0.55	1.027	0.713	
CLCN4	0.724	1.426	1.01	
SNTB1	0.571	1.067	0.623	
TTTY5	0.626	1.205	0.717	
DLEC1	0.587	1.103	0.669	
TXK	0.652	1.303	0.77	
SLAMF8	0.718	1.31	0.822	
NRN1L	1.437	2.785	1.504	
CEACAM21	0.607	1.107	0.621	
COX17	0.648	1.023	0.656	
NUPR1	2.129	3.48	2.283	
LOC643401	1.119	1.884	1.209	
BMF	0.724	1.226	0.784	
TRAPPC2P1	1.071	1.943	1.275	
NFE2L1	0.596	1.057	0.706	
MTL5	1.092	1.845	1.268	
HAPLN3	1.039	0.603	0.814	
CD38	1.113	0.635	0.91	
AHI1	1.577	0.909	1.146	
SMC2	1.007	0.678	0.817	
OVOS	1.026	0.69	0.87	
PILRB	1.677	1.146	1.498	
LOC729051	1.165	0.78	1.067	
C4orf21	1.085	0.66	1.051	
SPINK6	1.108	0.69	1.212	
RGMA	1.208	0.759	1.272	
LUC7L3	1.282	0.768	1.313	
SAT1	0.943	0.642	1.012	
BOD1L	1.568	1.087	1.722	
TBC1D3G	1.552	1.097	1.545	
PILRA	1.429	0.981	1.41	
BREA2	1.095	0.762	1.094	
HOXA1	0.977	0.688	1.008	
GOLGA4	1.531	1.025	1.541	
ST3GAL5	1.302	0.931	1.274	
SETDB1	1.023	0.757	0.999	
CENPE	1.794	1.364	1.846	
KTN1	1.113	0.533	0.938	
CUZD1	1.396	0.7	1.228	
ANKRD12	1.319	0.663	1.136	
C2orf60	1.228	1.259	1.266	UP
BICD1	1.37	1.412	1.385	
REC8	3.007	3.162	3.119	
SFRS18	1.848	1.868	1.864	
USP53	1.821	1.945	1.836	
KLF9	1.152	1.234	1.246	UP
CHKB	1.089	1.188	1.21	UP
ZC3H7A	1.005	1.092	1.082	
TIMD4	2.043	2.02	2.059	UP
RNF215	1.302	1.281	1.305	UP
RB1CC1	0.966	0.956	1.006	UP
ITGAV	0.979	0.952	1.006	UP
RICTOR	1.256	1.149	1.226	UP
NUF2	1.275	1.19	1.25	UP
GNRH1	3.177	3.029	2.994	
CASD1	1.65	1.622	1.535	
TMEM206	0.927	0.913	1.029	UP
BNIP2	1.064	1.037	1.142	UP
N4BP2L2	1.229	1.253	1.365	UP
C8orf68	0.992	1.031	1.121	UP
C13orf37	1.336	1.402	1.463	UP
KIAA0408	1.372	1.495	1.621	UP
C12orf73	0.884	0.973	1.057	UP
LOC400027	1.885	1.996	2.227	UP

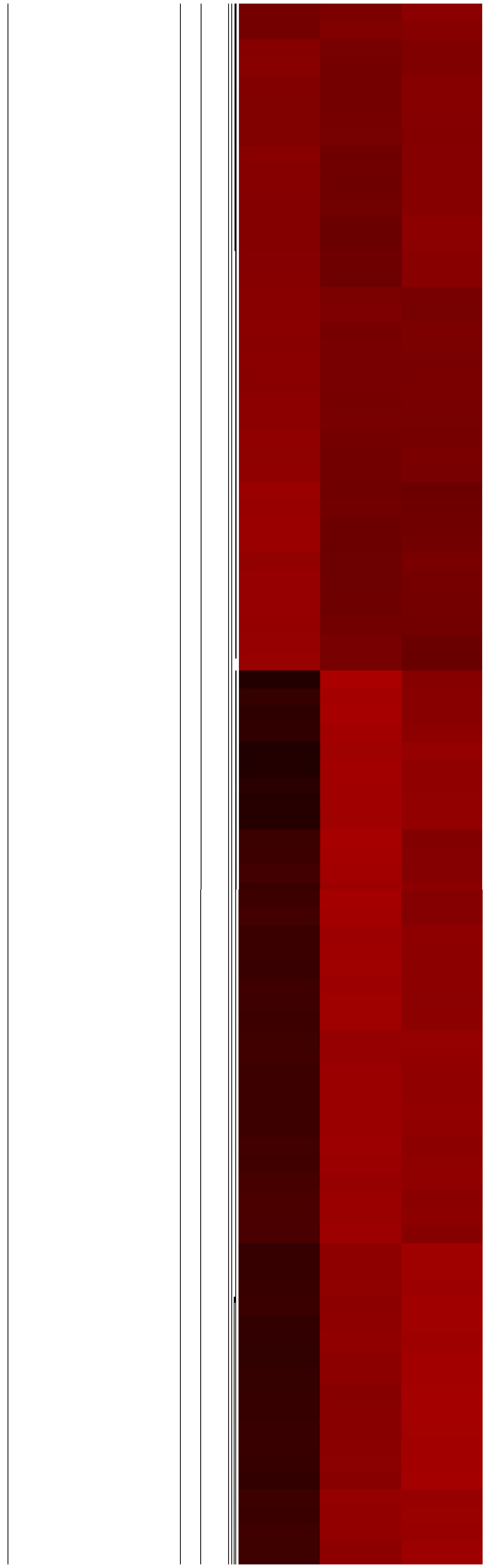

CTSK	2.583	2.848	2.971	UP
ZDHHC11B	1.835	1.628	1.753	UP
CCDC116	1.116	0.95	1.049	UP
ZDHHC11	1.736	1.55	1.795	UP
NCRNA00201	1.63	1.473	1.695	UP
TYW3	1.541	1.413	1.593	UP
C1orf162	1.349	1.247	1.378	UP
GOLGA6L10	1.345	1.091	1.321	
ARHGAP11A	1.191	0.989	1.185	UP
hCG_2015435	1.573	1.297	1.572	UP
NKTR	1.224	1.049	1.24	UP
ROCK1	1.448	1.165	1.534	UP
LOC284513	1.664	1.351	1.749	UP
LOC374890	1.902	1.567	1.942	UP
CWF19L2	1.692	1.379	1.735	UP
MAN2A2	1.916	1.725	1.674	
LMO7	1.056	0.975	0.921	
NCRNA00115	1.217	1.043	1.097	
CCDC82	1.129	0.984	1.014	UP
CCDC126	1.279	1.121	1.119	
ABCB10	1.109	0.978	0.985	UP
IFI27	1.23	1.032	1.051	
ADRA1B	1.842	1.565	1.552	
RAB40C	1.126	0.914	0.922	
CPT1B	2.332	1.884	1.993	
ARHGAP5	1.274	1.014	1.055	
HERC2P2	1.661	1.2	1.158	
ELMO3	1.026	0.77	0.751	
LOC643008	2.16	1.497	1.562	
LOC283588	1.295	0.907	0.955	
AKAP9	1.499	1.117	1.247	
SIAE	1.211	0.879	0.95	
LOC285771	1.217	0.898	0.939	
CALML4	1.46	1.065	1.121	
KCNA10	1.104	0.854	0.86	
OSGIN1	1.51	1.205	1.066	
OR2B6	1.176	0.913	0.804	
CMTM3	0.227	1.098	0.857	
DDB2	0.357	1.128	0.927	
AP4S1	0.311	1.108	0.912	
TXNIP	1.079	3.602	3.142	
NKX2-4	0.22	1.076	1.01	
LOC646470	0.238	1.123	1.008	
CDCP1	0.571	2.168	1.97	
YARS	0.32	1.326	1.213	
OGFRL1	0.365	1.53	1.41	
ST3GAL1	0.45	1.254	0.991	
SERAC1	0.392	1.065	0.86	
C15orf48	1.224	2.933	2.409	
SLC7A1	0.585	1.584	1.433	
TSC22D3	0.654	1.792	1.601	
LOC100130938	0.68	1.92	1.705	
ZNF655	0.396	1.001	0.897	
ESAM	1.157	2.935	2.555	
CLIC4	0.515	1.369	1.199	
TTC17	0.494	1.171	1.171	UP
ALG13	0.496	1.159	1.12	UP
KSR2	0.773	1.941	1.815	
CCDC112	0.761	1.935	1.831	
SKA2	0.462	1.179	1.117	
PANX3	0.455	1.165	1.088	
TMEM87B	0.587	1.392	1.238	
TFAP2B	0.517	1.217	1.125	
HIST1H1T	0.534	1.154	1.1	UP
UCN2	0.816	1.668	1.496	
CITED4	0.483	1.001	0.915	
SLC39A1	0.533	1.131	0.97	
GRB10	0.478	1.246	1.39	UP
BOD1	0.344	0.904	1.014	UP
RYK	0.423	1.062	1.168	UP
CDAN1	0.509	1.218	1.393	UP
PEAR1	1.056	2.941	3.327	UP
AXL	0.576	1.648	1.808	UP
ZBTB44	0.426	1.229	1.412	UP
TFAP2C	0.471	1.248	1.445	UP
TMEM126A	0.36	0.901	1.096	UP
ITPRIP	0.534	1.338	1.602	UP
IL10RB	0.44	1.083	1.285	UP
BRWD1	0.36	0.875	1.025	UP
TRIAP1	0.449	1.125	1.309	UP
RASGRF2	0.401	1.076	1.296	UP
FAM24B	1.008	2.506	2.543	UP
C1orf133	1.082	2.755	2.863	UP
H1FX	0.676	1.591	1.656	UP
DOCK4	0.563	1.255	1.386	UP
DDX6	0.56	1.298	1.417	UP
TFPI2	0.72	1.675	1.791	UP
IL20RB	0.749	1.717	1.878	UP

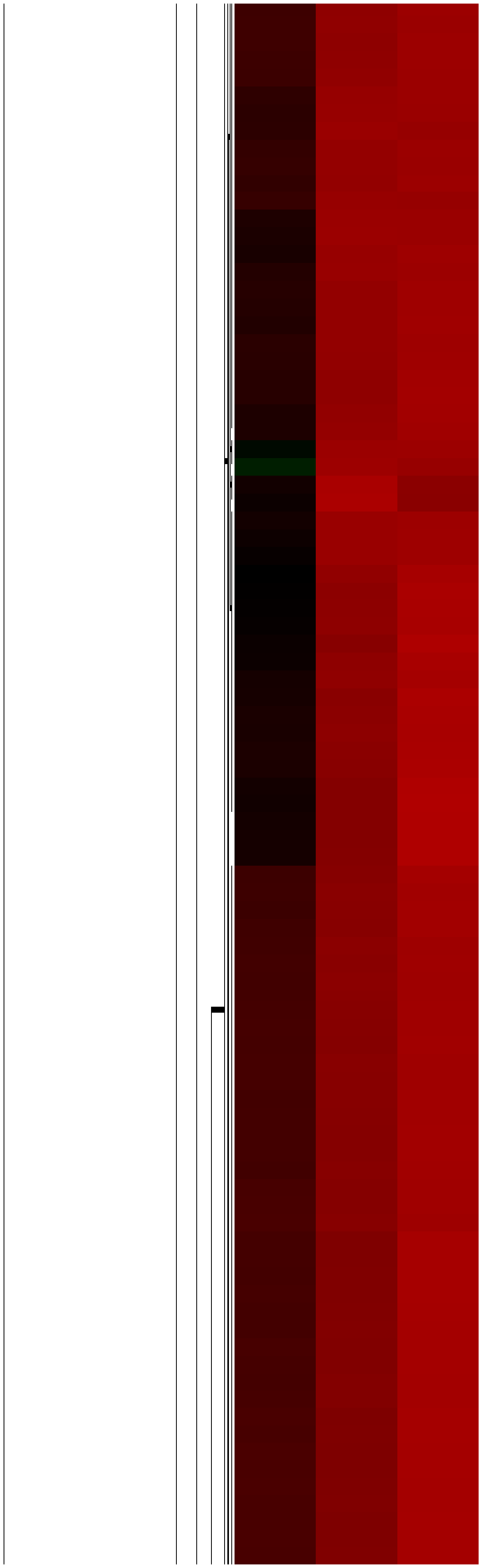

GDI1	0.39	0.922	1.003	UP
SLC22A5	0.603	1.468	1.572	UP
LAT2	0.817	2.641	2.733	
ASB10	0.486	1.685	1.713	
DLC1	0.324	1.126	1.098	
SLC3A2	0.498	1.422	1.489	
SEC61A2	0.516	1.422	1.481	UP
FEZ2	0.328	0.98	1.037	
TRIT1	0.404	1.119	1.112	
SLC25A32	0.247	1.26	1.257	
FAM18B2	0.183	1.035	1.033	
SAMD5	0.278	1.691	1.775	
LAMC2	0.694	3.013	3.093	
NPC1	0.33	1.276	1.378	
LOC283585	0.259	1.051	1.137	
RSPH9	0.274	1.193	1.293	
SESN1	0.497	1.748	1.874	
LYPD1	0.386	1.366	1.486	
LOC285758	0.265	0.951	1.078	
ARHGEF18	0.309	1.125	1.286	
ODC1	0.292	1.455	1.617	
MMP9	0.261	1.312	1.423	
CYP26A1	-0.084	1.329	1.336	
CCR1	-0.21	1.084	1.042	
MOBKL2B	0.114	1.042	0.857	
FAM90A7	0.072	1.015	0.821	
EPHB2	0.215	1.693	1.747	
ANKRD18A	0.126	1.424	1.471	
UPP1	0.092	1.769	1.837	
TLE3	0.006	1.402	1.603	
LOC100133728	0.018	0.95	1.151	
IRX2	0.031	0.949	1.135	
HIST1H2AC	0.04	0.921	1.093	
LOC100133660	0.067	0.783	1.006	
H1F0	0.153	1.747	2.063	
IRX4	0.15	0.996	1.145	
RAB11B	0.161	1.002	1.255	
LOC388965	0.195	1.037	1.256	
FAM196B	0.241	1.341	1.597	
ZNF14	0.232	1.144	1.399	
KIAA1644	0.377	1.894	2.38	
SAMD4A	0.249	1.658	2.208	
EIF2B5	0.102	0.774	1.03	
LOC392335	0.267	1.741	2.301	
CLEC18B	0.128	0.8	1.065	
PLEK2	0.199	1.24	1.642	
ZNF609	0.478	1.069	1.305	UP
LY6K	1.47	3.309	3.886	UP
CALML6	0.636	1.455	1.732	UP
ABTB2	0.831	1.773	2.122	UP
SLC22A1	0.558	1.205	1.371	UP
PVR	0.919	1.91	2.206	UP
HERPUD1	0.419	0.894	1.015	UP
C12orf60	1.009	2.091	2.421	UP
CNNM3	0.5	0.984	1.171	UP
ALG14	0.476	0.917	1.095	UP
GFM1	0.566	1.112	1.346	UP
GDF15	1.072	2.099	2.452	UP
CSGALNACT2	0.917	1.781	2.1	UP
ELOVL1	0.467	0.953	1.136	UP
EDA2R	0.651	1.3	1.568	UP
CDKN1A	1.341	2.658	3.215	UP
CCBE1	0.657	1.289	1.57	UP
PLCXD2	0.626	1.286	1.531	UP
QSOX1	0.527	0.982	1.175	UP
MED30	0.752	1.39	1.662	UP
PTTG3P	0.466	0.856	1	UP
GRPEL2	0.423	0.786	1.025	UP
CSNK1E	0.502	0.932	1.215	UP
VASN	1.014	1.92	2.497	UP
C20orf194	0.534	0.989	1.284	UP
C10orf54	1.118	2.143	2.737	UP
TERF2	0.413	0.804	1.016	UP
SERINC1	0.605	1.097	1.39	UP
RG9MTD2	0.909	1.689	2.151	UP
LOC100130320	0.52	1.003	1.245	UP
FOXD3	1.026	1.913	2.396	UP
HECA	0.647	1.111	1.459	UP
CDKN1C	0.629	1.11	1.447	UP
ZNRD1	0.506	0.854	1.113	UP
YPEL2	0.569	0.983	1.28	UP
UTY	0.817	1.403	1.805	UP
TMEM167B	1.067	1.877	2.41	UP
SEMA4C	0.469	0.815	1.065	UP
C6orf35	1.095	1.903	2.458	UP
AKT3	0.468	0.832	1.067	UP
ELK3	0.898	1.591	1.988	UP
ATG4D	0.82	1.42	1.798	UP

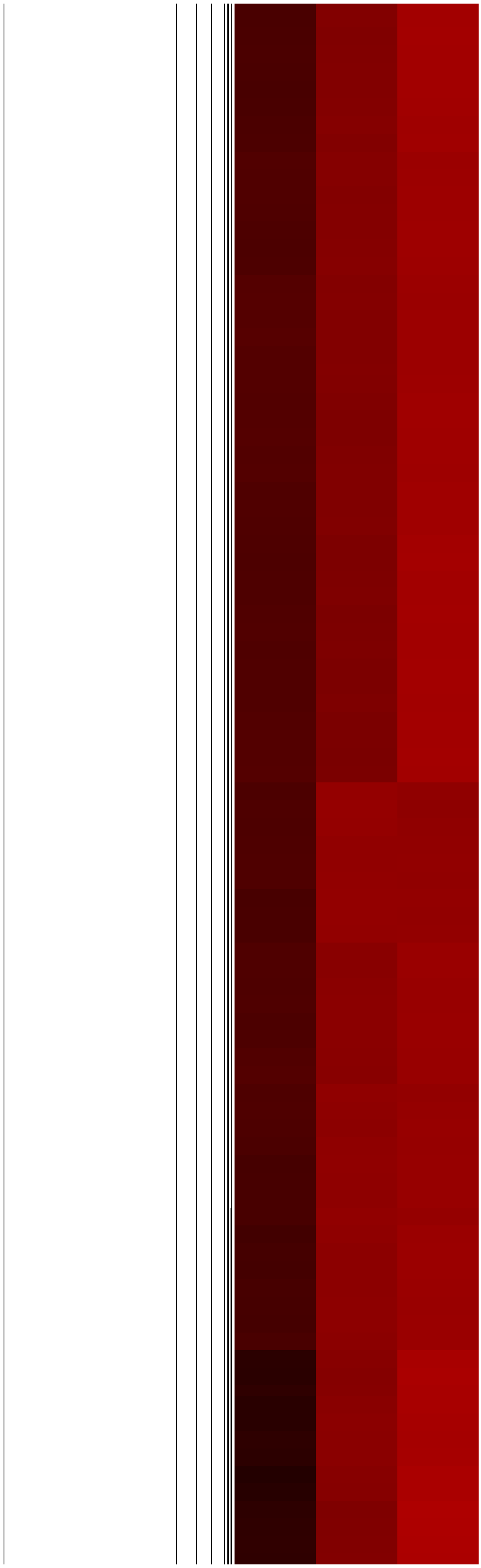

FRZB	0.613	1.045	1.316	UP
MYEF2	0.78	1.367	1.704	UP
B4GALT3	0.486	0.872	1.082	UP
WIPF1	0.734	1.314	1.617	UP
MICA	0.525	0.931	1.116	UP
MAP2K1	0.49	0.841	1.034	UP
PDP1	0.709	1.163	1.359	UP
BAK1	0.729	1.218	1.422	UP
ATAD1	1	1.631	1.957	UP
C10orf35	0.629	1.053	1.253	UP
ABHD2	0.681	1.164	1.387	UP
SLC35F2	1.118	1.958	2.314	UP
CSNK1A1L	0.558	0.965	1.131	UP
LOC730167	0.692	1.076	1.268	UP
LOC221710	1.291	2.006	2.345	UP
STK40	1.098	1.688	2.024	UP
BMI1	0.632	0.955	1.146	UP
UFM1	1.186	1.848	2.217	UP
CTNNAL1	1.122	1.755	2.093	UP
CCDC58	0.627	0.971	1.176	UP
FLJ45721	0.563	0.87	1.086	UP
C1orf77	1.024	1.563	1.979	UP
TMEM87A	1.019	1.519	1.92	UP
TNFRSF10D	1.632	2.553	3.172	UP
IL8	2.94	4.529	5.556	UP
SETD7	0.619	1.015	1.252	UP
C9orf21	0.752	1.199	1.502	UP
ZNF805	1.119	1.827	2.262	UP
KCNJ14	0.731	1.168	1.525	UP
GOLT1B	0.562	0.917	1.196	UP
FAM18B	0.682	1.105	1.417	UP
DGKH	0.5	0.816	1.034	UP
PTPLB	0.917	1.409	1.852	UP
EREG	1.932	2.974	3.872	UP
CLDN1	1.184	1.875	2.415	UP
MARCH4	1.434	2.228	2.935	UP
SESN2	1.375	2.138	2.804	UP
RABL3	0.541	0.845	1.088	UP
SBDSP	0.806	1.196	1.59	UP
RPL22L1	0.588	0.868	1.142	UP
ROR1	1.229	1.811	2.416	UP
PLOD2	0.906	1.335	1.752	UP
SLC35A3	0.527	1.022	0.989	UP
DDR2	0.61	1.168	1.115	UP
TAP1	0.736	1.417	1.376	UP
PRNP	0.811	1.507	1.507	UP
FST	1.193	2.195	2.207	UP
C10orf47	0.997	1.846	1.832	UP
RNF41	0.573	1.158	1.16	UP
PRR24	0.96	1.941	1.931	UP
JHDM1D	1.033	2.051	2.063	UP
RNF152	0.524	0.909	1.011	UP
ING5	0.685	1.177	1.335	UP
SLMO1	1.333	2.347	2.578	UP
KIAA0513	0.681	1.191	1.302	UP
TCAP	0.742	1.346	1.482	UP
MYEOV	0.623	1.11	1.233	UP
ZIC5	0.585	0.986	1.09	UP
MOSPD1	1.345	2.2	2.458	UP
PAIP2	0.745	1.363	1.398	UP
EIF5A2	0.858	1.53	1.606	UP
SNHG9	0.892	1.621	1.74	UP
CYCS	0.512	0.971	1.02	UP
TIMM44	0.907	1.845	1.94	UP
CHST11	0.84	1.682	1.78	UP
TRIM15	0.731	1.431	1.534	UP
TMBIM1	0.606	1.209	1.247	UP
SMG5	0.874	1.872	2.017	UP
DPH3	0.57	1.157	1.284	UP
C4orf32	0.88	1.799	1.994	UP
MCTP1	1.671	3.294	3.62	UP
C19orf28	0.723	1.456	1.578	UP
TM9SF3	0.454	0.926	1.014	UP
GLIPR1	0.922	1.743	1.939	UP
PFKP	0.278	0.859	1.066	UP
GREM1	1.048	3.277	4.168	UP
SLC39A14	0.446	1.271	1.595	UP
SERPINB8	0.706	2.349	2.879	UP
NOG	1.162	3.913	4.671	UP
LTB	1.375	4.067	4.852	UP
COL4A1	0.526	1.632	1.955	UP
RAB3B	0.284	1.079	1.359	UP
GOLT1A	0.838	2.881	3.654	UP
PRKAG2	0.477	1.327	1.82	UP
NIPAL1	0.644	1.752	2.369	UP
TAF8	0.362	0.953	1.273	UP
SPTLC1	0.43	1.172	1.54	UP
NHS	0.41	1.182	1.563	UP

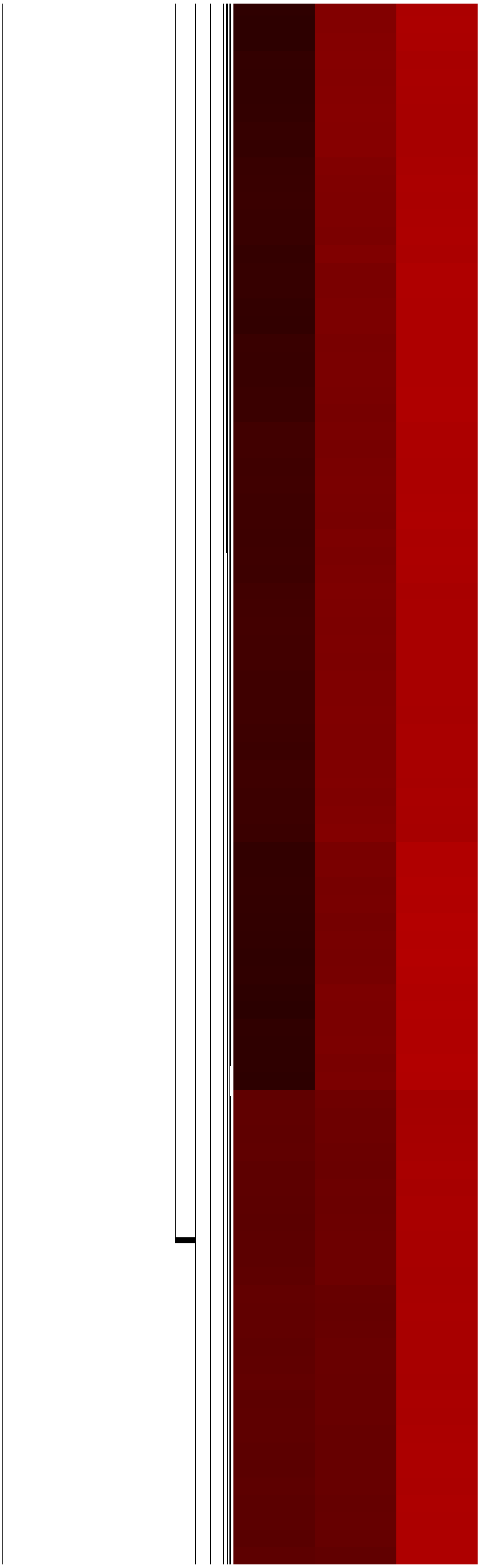

MIS12	0.356	1.042	1.35	UP
TMEM33	0.536	1.382	1.747	UP
DR1	0.462	1.199	1.539	UP
VGF	0.489	1.308	1.647	UP
TRPV1	0.531	1.395	1.735	UP
LOC100127983	0.629	1.527	1.922	UP
CNNM4	0.428	1.092	1.361	UP
SERPINE1	1.541	3.501	4.595	UP
C10orf110	1.359	3.034	4.07	UP
ZDBF2	0.658	1.425	1.918	UP
ELFN2	0.648	1.447	1.986	UP
DNAJB2	1.254	2.728	3.823	UP
SLC7A6OS	0.478	1.17	1.567	UP
MTSS1L	0.636	1.432	2.065	UP
LOC392288	0.699	1.581	2.259	UP
NIPAL4	0.653	1.587	2.226	UP
KIAA0556	0.434	1.068	1.497	UP
SDC4	1.006	2.119	3.043	UP
SAR1B	0.547	1.179	1.679	UP
DPH3B	0.513	1.101	1.576	UP
CGB	0.376	0.782	1.132	UP
TOP1P2	0.751	1.527	2.241	UP
TSPAN9	0.534	0.99	1.407	UP
DAGLB	0.716	1.318	1.91	UP
PFDN2	0.595	1.138	1.617	UP
LOC100128009	0.518	0.987	1.394	UP
NUDT4	0.555	1.079	1.538	UP
CYTH1	0.638	1.232	1.776	UP
BRWD3	0.586	1.193	1.643	UP
BBC3	1.217	2.403	3.378	UP
LOC100130996	0.978	1.998	2.751	UP
NCBP2	0.53	1.027	1.371	UP
LOC400568	0.517	0.973	1.318	UP
KIAA1467	0.552	1.011	1.376	UP
FKRP	0.53	0.997	1.343	UP
TNFRSF10A	1.044	1.956	2.656	UP
BRF2	0.569	1.147	1.519	UP
ATG9A	0.592	1.177	1.563	UP
RASA2	0.738	1.501	1.954	UP
TMEM189	0.408	0.855	1.135	UP
KRT10	0.498	1.067	1.418	UP
PSEN1	0.452	0.932	1.229	UP
FNTA	0.455	0.939	1.214	UP
DNMBP	0.494	1.039	1.377	UP
DEPDC1B	0.404	0.861	1.116	UP
ZNF649	0.391	0.876	1.115	UP
CAV3	0.383	0.918	1.343	UP
B3GNT5	0.862	2.06	2.983	UP
WBSCR16	0.295	0.678	1.012	UP
TNFSF9	0.555	1.299	1.919	UP
SUSD1	0.287	0.673	1.032	UP
PIM1	0.641	1.559	2.338	UP
KDM6B	1.056	2.621	3.995	UP
INTU	0.348	0.86	1.283	UP
UHMK1	0.286	0.79	1.116	UP
SLC25A17	0.347	0.981	1.406	UP
TBC1D22B	0.563	1.439	2.057	UP
GALNT2	0.305	0.799	1.134	UP
RPS19BP1	0.369	0.955	1.396	UP
C1orf109	0.336	0.91	1.313	UP
SKIL	1.304	1.503	2.221	UP
AOC3	2.36	2.672	4.05	UP
FGFR1OP2	1.003	1.151	1.752	UP
ANKRD1	1.908	2.13	3.322	UP
LRIG2	0.762	0.868	1.377	UP
KPNA5	1.686	1.954	3.024	UP
PTP4A1	0.555	0.647	1.024	UP
ZNF192	0.583	0.696	1.082	UP
MTM1	0.614	0.733	1.121	UP
DNAJC25	1.211	1.434	2.211	UP
DCUN1D1	0.657	0.763	1.165	UP
ZNF292	0.761	0.802	1.304	UP
RNGTT	0.652	0.69	1.137	UP
PVRL4	0.823	0.879	1.426	UP
NIPBL	0.806	0.889	1.425	UP
IMPA1	0.783	0.853	1.363	UP
ANLN	0.842	0.892	1.428	UP
ZNF644	0.614	0.684	1.117	UP
ZBTB11	1.12	1.24	2.003	UP
ZC3H11A	0.796	0.871	1.448	UP
TOR1AIP1	0.714	0.79	1.334	UP
LIN28B	0.686	0.779	1.294	UP
DDX3Y	1.336	1.486	2.451	UP
GUCA1A	0.54	0.601	1.029	UP
BCL6B	0.615	0.693	1.163	UP
THAP3	1.538	1.734	2.994	UP
LARP1B	0.617	0.643	1.149	UP
HSF2	0.779	0.82	1.437	UP

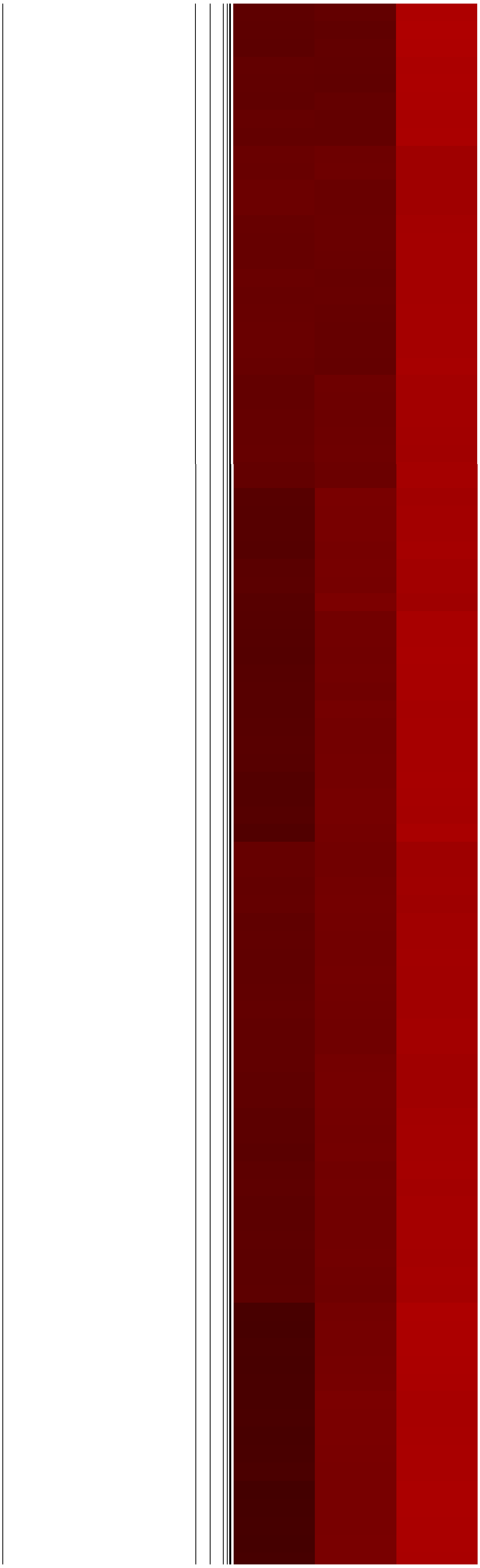

ZNF721	0.938	0.966	1.754	UP
MAP3K15	0.691	0.735	1.305	UP
TM4SF1	0.687	0.68	1.196	UP
STK3	0.662	0.651	1.169	UP
BHLHE40	0.75	0.784	1.342	UP
POU5F1	0.831	0.815	1.393	UP
GGNBP2	0.836	0.832	1.431	UP
TAPT1	1.275	1.327	1.929	UP
OTUD3	1.556	1.644	2.379	UP
PHIP	0.749	0.726	1.107	UP
MAPK8IP3	2.044	1.995	3.031	UP
GPN3	0.702	0.723	1.111	UP
FAM63B	1.07	1.109	1.717	UP
ZNF34	0.928	0.962	1.496	UP
RNF112	0.93	0.908	1.442	UP
LOC100133180	2.07	2.089	3.269	UP
PUM2	0.947	0.927	1.493	UP
C7orf53	2.86	2.737	4.466	UP
RNF160	1.078	1.039	1.687	UP
GAD1	0.932	0.908	1.509	UP
ZBTB1	0.826	0.913	1.356	UP
RFPL3S	1.351	1.481	2.21	UP
KLB	1.617	1.724	2.611	UP
DNAJC3	0.698	0.756	1.119	UP
SELT	0.697	0.751	1.102	UP
KLHL24	0.737	0.801	1.217	UP
DOT1L	0.962	1.04	1.607	UP
ZGLP1	1.198	1.662	2.206	UP
RC3H1	0.87	1.213	1.64	UP
C5orf41	1.717	2.385	3.258	UP
BIRC2	0.931	1.29	1.793	UP
SGPP1	1.139	1.508	2.045	UP
SCML2	1.29	1.67	2.284	UP
LSM1	0.638	0.908	1.16	UP
TCERG1	0.632	0.838	1.232	UP
CPNE8	1.035	1.382	2.043	UP
ZNF460	1.091	1.465	2.191	UP
TNFRSF10B	1.224	1.616	2.38	UP
PLK3	1.475	1.892	2.834	UP
ZMYM2	0.604	0.814	1.152	UP
TMEM184A	1.136	1.496	2.145	UP
SFRS12IP1	0.965	1.256	1.81	UP
HIST2H2AA4	0.718	0.955	1.366	UP
SBDS	0.964	1.345	1.935	UP
TCF7L2	0.797	1.125	1.587	UP
MAG	0.711	0.989	1.385	UP
IRAK2	1.713	2.469	3.575	UP
RC3H2	0.683	0.757	1.053	UP
JMJD1C	1.695	1.889	2.667	UP
SLTM	0.891	1.032	1.437	UP
EZH1	1.247	1.452	1.979	UP
KAT2B	0.649	0.776	1.087	UP
HCN3	1.661	1.959	2.756	UP
FBXO36	1.04	1.234	1.743	UP
CLDND1	1.048	1.255	1.751	UP
CXADR	0.883	1.031	1.462	UP
ARG2	0.813	0.928	1.327	UP
PLAUR	0.864	0.993	1.449	UP
BAZ2B	0.877	1.012	1.477	UP
KCTD9	1.088	1.3	1.783	UP
CENPQ	0.72	0.885	1.206	UP
ZNF238	1.088	1.343	1.838	UP
DDX10	1.203	1.505	2.109	UP
CD274	2.59	3.238	4.609	UP
DUSP5	1.026	1.317	1.861	UP
EXOC8	0.584	0.708	1.035	UP
DOHH	1.484	1.808	2.581	UP
CPEB2	1.397	1.713	2.512	UP
CNOT8	0.597	0.727	1.062	UP
UPF3B	1.473	1.796	2.613	UP
PIBF1	1.049	1.29	1.863	UP
SLC39A7	0.625	0.761	1.123	UP
MNT	0.881	1.048	1.556	UP
DIAPH1	0.58	0.935	1.39	UP
CCDC47	0.497	0.812	1.189	UP
RASSF1	1.538	2.458	3.603	UP
TNFRSF12A	1.512	2.478	3.594	UP
PDGFB	1.628	2.626	3.752	UP
VSTM2A	0.821	1.367	1.86	UP
LAT	0.48	0.788	1.08	UP
SLC16A1	0.594	1.013	1.391	UP
E2F4	0.55	0.906	1.261	UP
CXorf26	0.45	0.718	1.01	UP
INPP1	0.558	0.978	1.396	UP
CCDC28A	0.472	0.821	1.173	UP
SNX16	0.639	1.087	1.542	UP
MGC16703	0.918	1.595	2.251	UP
E2F5	0.613	1.044	1.458	UP

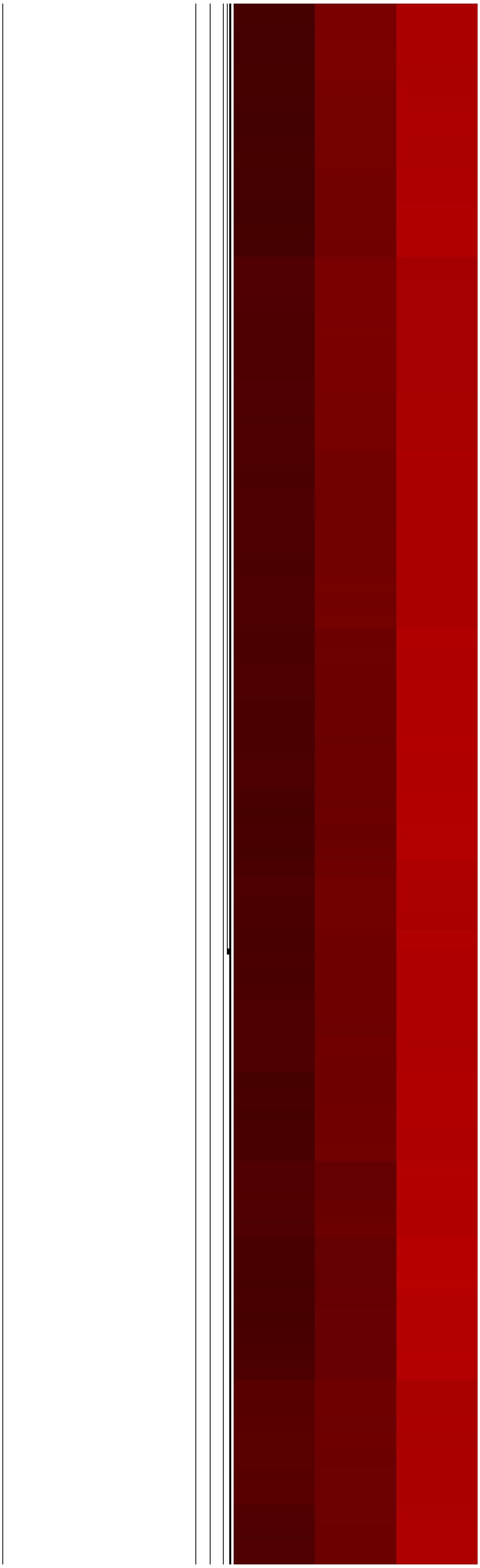

PHC2	0.764	1.321	1.845	UP
NEK7	0.923	1.648	2.284	UP
GABARAPL1	1.223	2.192	3.029	UP
CEP135	0.767	1.369	1.881	UP
SERTAD4	1.307	2.23	3.243	UP
PRKAB2	0.775	1.338	1.976	UP
CCDC91	0.415	0.733	1.085	UP
AREG	1.333	2.28	3.376	UP
TMEM170A	0.425	0.71	1.057	UP
RBM9	0.532	0.896	1.362	UP
FSTL3	0.818	1.383	2.091	UP
CAV1	0.386	0.664	1.013	UP
STX1A	0.813	1.329	2.059	UP
FAM3C	0.468	0.77	1.216	UP
PTHLH	1.367	2.058	2.811	UP
DOLPP1	0.613	0.918	1.258	UP
ZNF550	0.839	1.277	1.775	UP
PTX3	0.884	1.368	1.9	UP
ARSJ	0.568	0.897	1.223	UP
TAF9	0.634	1.001	1.388	UP
NADK	0.516	0.788	1.099	UP
C6orf145	1.068	1.623	2.266	UP
WBP4	0.968	1.472	2.096	UP
JUB	1.169	1.828	2.586	UP
FKBP14	0.586	0.893	1.284	UP
SH2D3A	0.695	1.031	1.544	UP
KRR1	0.692	1.051	1.573	UP
LRRFIP2	0.481	0.695	1.056	UP
C3orf19	0.649	0.944	1.434	UP
BDNF	0.665	0.977	1.468	UP
ASH1L	0.556	0.83	1.254	UP
UBIAD1	0.453	0.691	1.039	UP
LOC642521	0.551	0.833	1.23	UP
C1GALT1	1.057	1.557	2.302	UP
SLC19A2	1.201	1.79	2.632	UP
ZNF335	1.059	1.528	2.488	UP
PRDM10	0.44	0.638	1.02	UP
PLEKHM2	0.458	0.639	1.036	UP
MITF	0.832	1.155	1.913	UP
MKLN1	0.577	0.819	1.344	UP
ELK4	0.503	0.735	1.208	UP
SPAG1	0.427	0.619	1.026	UP
SS18	0.792	1.105	1.841	UP
SMCR8	0.595	0.824	1.367	UP
TDG	0.868	1.263	2.091	UP
PSMC3IP	0.693	1.047	1.728	UP
MYST3	0.557	0.81	1.378	UP
CFLAR	0.65	0.973	1.621	UP
TNPO2	0.833	1.245	1.947	UP
NUDCD1	0.495	0.734	1.128	UP
CWC22	1.14	1.71	2.622	UP
CRLF3	0.517	0.79	1.183	UP
FAM161B	0.526	0.786	1.258	UP
DMPK	0.795	1.189	1.86	UP
C1D	0.43	0.659	1.037	UP
ANKRD37	0.621	0.932	1.459	UP
KIAA0182	0.656	0.942	1.496	UP
GPATCH8	0.779	1.086	1.736	UP
FEM1A	0.573	0.834	1.285	UP
C16orf87	0.615	0.876	1.378	UP
BAZ2A	0.451	0.715	1.141	UP
ARHGAP19	1.103	1.682	2.706	UP
S100A14	0.538	0.851	1.33	UP
NKIRAS1	0.766	1.185	1.86	UP
HLA-DQB1	0.42	0.653	1.012	UP
RHBDF1	1.048	1.317	2.335	UP
PPP4R2	0.649	0.815	1.425	UP
LOC100132672	1.071	1.395	2.348	UP
ARID4B	0.721	0.97	1.603	UP
LIFR	0.671	0.912	1.672	UP
FIZ1	0.421	0.59	1.063	UP
RNF111	0.411	0.583	1.056	UP
RUSC2	0.8	1.108	1.961	UP
PAPOLG	0.758	1.097	1.927	UP
IFNGR2	0.51	0.733	1.277	UP
IER3	0.771	1.047	1.835	UP
ARPP19	0.679	0.919	1.588	UP
LOC389831	0.865	1.08	1.658	UP
CHERP	0.523	0.675	1.029	UP
ZC3H15	0.571	0.712	1.104	UP
MORC3	1.309	1.611	2.468	UP
FBXL3	0.73	0.892	1.394	UP
EPHA2	1.702	2.118	3.311	UP
DDX52	0.931	1.154	1.796	UP
NCEH1	0.886	1.185	1.886	UP
LCAT	1.968	2.684	4.313	UP
SMAD3	0.54	0.733	1.176	UP
STRN3	1.094	1.474	2.335	UP

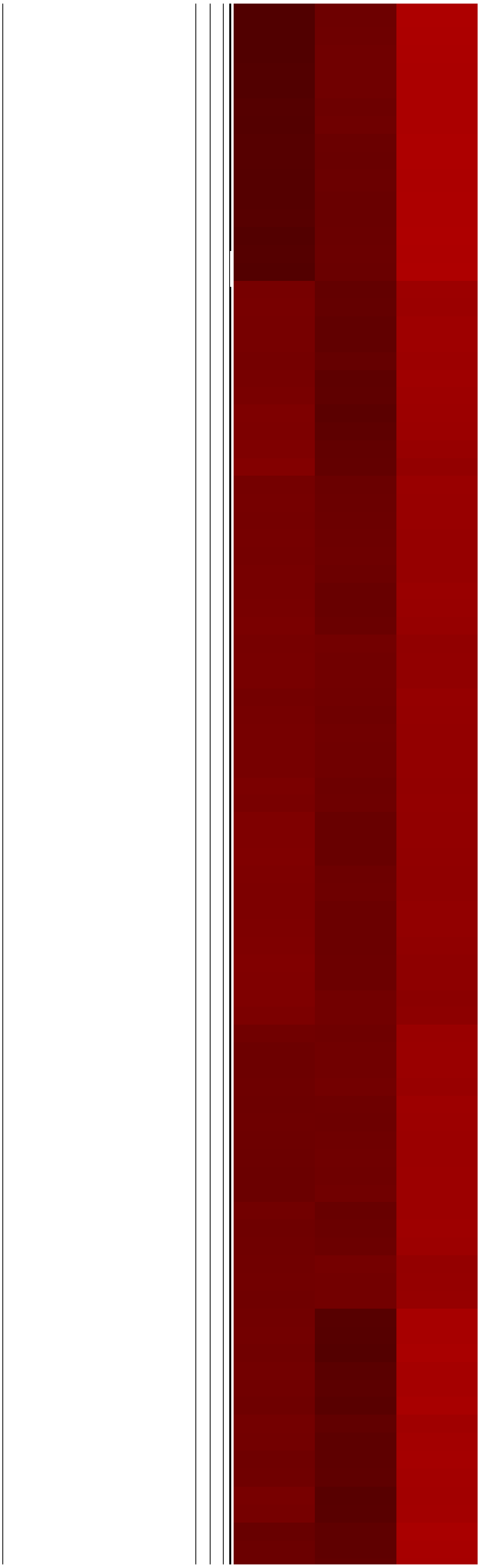

LIF	1.273	1.709	2.691	UP
RAB28	0.528	0.714	1.113	UP
CLK1	1.018	1.41	2.151	UP
YAP1	0.632	0.858	1.299	UP
RBM24	1.134	1.552	2.364	UP
TOP1	1.21	1.545	2.432	UP
NUPL1	0.763	1.004	1.545	UP
APOE	0.637	0.784	1.277	UP
AKAP8L	0.956	1.168	1.91	UP
SOCS3	1.581	2.001	3.2	UP
RPF2	0.645	0.795	1.304	UP
GPR75	0.513	0.615	1.015	UP
PDHX	0.59	0.749	1.231	UP
DCP2	1.029	1.295	2.092	UP
YPEL5	0.95	1.219	1.993	UP
PAPD4	0.9	0.749	1.183	UP
C1orf9	0.976	0.81	1.256	UP
EEA1	1.272	1.032	1.681	UP
ATL2	0.844	0.691	1.118	UP
PKN2	0.922	0.796	1.225	UP
UBR3	0.854	0.678	1.14	UP
KLHL28	1.934	1.512	2.503	UP
KIF18A	1.254	0.911	1.557	UP
GCC2	0.847	0.639	1.05	UP
TAC3	1.204	0.932	1.428	UP
AGAP7	1.578	1.196	1.772	
OSBPL8	1.088	0.984	1.414	UP
ETNK1	1.036	0.942	1.338	UP
TBX19	1.325	1.224	1.725	UP
THOC2	1.172	1.083	1.493	UP
CEPT1	1.349	1.271	1.734	UP
ZMYND11	0.862	0.782	1.083	UP
TBC1D3B	0.992	0.868	1.278	UP
MAGOH	1.373	1.189	1.744	UP
TAX1BP1	0.821	0.712	1.008	UP
THOC1	0.908	0.871	1.101	UP
STIM2	1.032	0.973	1.255	UP
TNFAIP6	1.307	1.245	1.587	UP
ZNF92	1.01	0.985	1.296	UP
KIF20B	1.194	1.121	1.493	UP
KIAA1033	0.914	0.868	1.127	UP
FNBP4	1.384	1.302	1.71	UP
FAM105B	1.148	1.078	1.421	UP
RUFY2	0.955	0.85	1.131	UP
C21orf66	0.923	0.841	1.115	UP
KIAA1731	1.119	0.918	1.291	UP
C15orf29	1.425	1.171	1.645	UP
PNPLA8	0.978	0.795	1.109	UP
E2F8	1.289	1.109	1.463	UP
AMH	1.169	1.03	1.345	UP
ZNF224	1.482	1.27	1.724	UP
ZBTB7A	0.961	0.815	1.112	UP
DNAJC12	0.974	0.827	1.112	UP
RP11-94I2.2	1.211	1.019	1.33	UP
EFHA2	1.424	1.199	1.577	UP
SLK	1.214	1.093	1.335	UP
MAPK8	1.151	1.06	1.308	UP
PRKAR2A	0.892	0.876	1.202	UP
LOC642513	1.085	1.129	1.532	UP
CHMP2B	0.894	0.923	1.253	UP
MEP1A	0.729	0.762	1.004	UP
RNF19B	1.193	1.201	1.715	UP
CAPRIN2	1.562	1.55	2.215	UP
ZNF341	1.789	1.818	2.543	UP
ATP1B1	0.815	0.842	1.165	UP
ADCY6	0.928	0.963	1.349	UP
KIF21A	1.369	1.448	1.992	UP
SUCLA2	0.912	0.834	1.246	UP
PRPF39	1.756	1.666	2.484	UP
MBP	0.78	0.75	1.081	UP
NPR2	1.697	1.754	2.215	UP
SMARCAD1	0.971	0.978	1.271	UP
NIPA1	1.14	1.164	1.525	UP
SGOL2	1.05	0.797	1.529	UP
AOC2	0.753	0.564	1.093	UP
HIPK3	0.931	0.69	1.39	UP
HIAT1	0.799	0.63	1.147	UP
GLCCI1	1.48	1.205	2.171	UP
DRAM1	0.723	0.583	1.09	UP
CCDC21	0.945	0.79	1.308	UP
C20orf106	1.864	1.515	2.633	UP
HMGB2	0.771	0.649	1.138	UP
CP110	0.921	0.783	1.339	UP
PJA2	0.748	0.554	1.013	UP
PHF10	0.842	0.635	1.154	UP
PRPF4B	0.839	0.768	1.354	UP
NPIP	0.649	0.574	1.009	UP
ARFIP1	0.795	0.72	1.232	UP

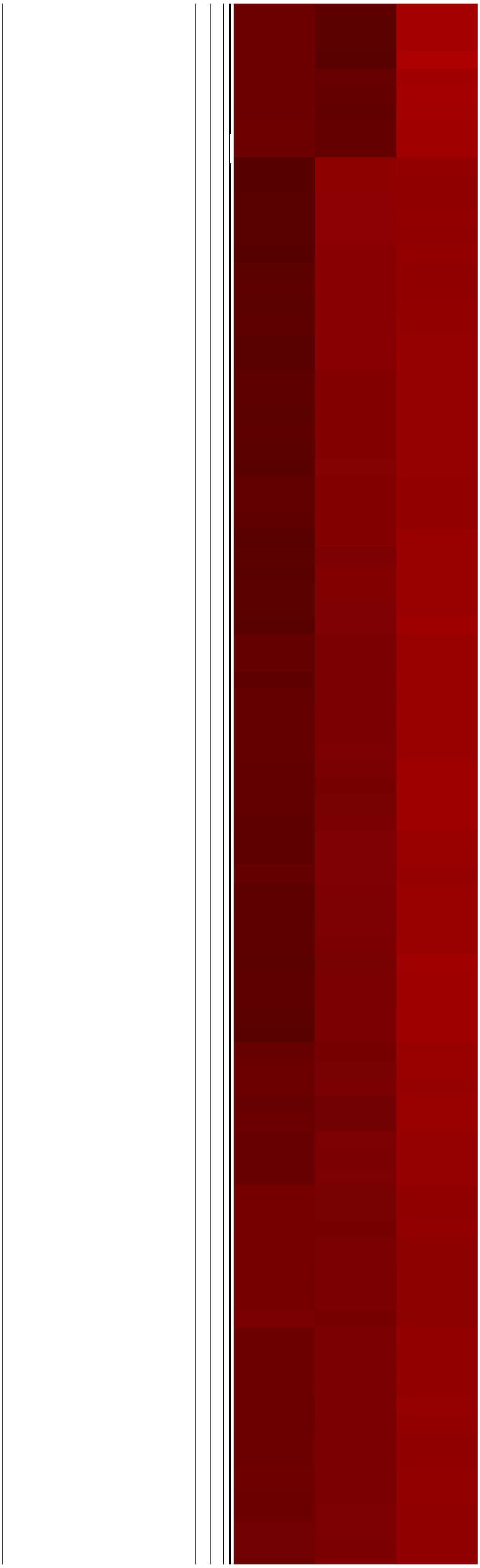

ZNF767	0.88	0.752	1.358	UP
BDP1	1.203	1.045	1.868	UP
BZW1	0.812	0.686	1.305	UP
LOC389024	0.767	0.722	1.149	UP
SATB2	1.273	1.185	1.962	UP
ABAT	0.759	0.681	1.129	UP
STK19	1.897	1.675	2.737	UP
CDC2	0.931	0.837	1.355	UP
LOC399959	1.622	2.597	2.707	UP
GPR111	0.607	0.988	1.012	UP
KLF12	0.825	1.268	1.33	UP
CCAR1	0.769	1.191	1.251	UP
LONRF3	1.438	2.198	2.299	UP
VEGFA	1.455	2.282	2.465	UP
GBP3	1.066	1.588	1.68	UP
FAM72A	0.999	1.451	1.547	UP
PMAIP1	1.204	1.741	1.88	UP
COMMD10	0.907	1.29	1.424	UP
ZFY	1.066	1.589	1.764	UP
TNFRSF9	1.234	1.886	2.11	UP
PYGO1	0.718	0.983	1.149	UP
IL1RAP	1.897	2.639	3.003	UP
FAM177A1	0.691	0.963	1.111	UP
SLC35D1	1.219	1.708	1.939	UP
VDR	1.075	1.539	1.75	UP
LAMC1	0.801	1.165	1.317	UP
SBF2	0.718	0.951	1.087	UP
RRM2B	1.175	1.559	1.754	UP
AMD1	1.262	1.691	1.928	UP
EMP1	1.733	2.552	2.998	UP
TMEM156	1.972	2.677	3.314	UP
PHF5A	0.72	1.015	1.223	UP
IDS	0.625	0.863	1.036	UP
C2orf63	1.24	1.715	2.075	UP
PKD2L2	0.952	1.351	1.666	UP
SEMA3C	1.347	1.594	2.059	UP
MAP3K2	0.868	1.027	1.327	UP
CNOT6L	0.811	1.013	1.288	UP
CHD1	0.963	1.167	1.491	UP
ZFR	0.686	0.837	1.055	UP
PPL	1.454	1.779	2.207	UP
HOXA4	0.953	1.194	1.478	UP
WBP5	0.692	0.848	1.105	UP
TNFAIP2	1.268	1.523	2.028	UP
REM2	2.712	3.295	4.322	UP
LOC387763	3.528	4.336	5.671	UP
MOBKL1A	0.698	0.932	1.11	UP
C12orf30	0.709	0.938	1.121	UP
B3GALTL	0.811	1.035	1.227	UP
THAP9	1.399	1.86	2.288	UP
NEFM	1.034	1.341	1.675	UP
CLK4	1.363	1.748	2.153	UP
BIRC3	2.593	3.285	4.134	UP
MAFK	1.302	1.668	2.226	UP
SH3D20	0.738	0.939	1.226	UP
YOD1	1.238	1.604	2.086	UP
PDCD10	0.791	1.042	1.331	UP
TMEM22	0.956	1.286	1.659	UP
SLC38A2	1.505	1.69	2.176	UP
MCL1	1.342	1.51	1.93	UP
IQCB1	1.136	1.28	1.604	UP
PHLDA1	1.115	1.228	1.654	UP
ACER3	1.18	1.287	1.728	UP
GJC2	1.273	1.525	1.834	UP
FAM73B	0.855	1.026	1.247	UP
LOC100128416	1.176	1.412	1.712	UP
TROVE2	0.943	0.968	1.166	UP
C3orf58	0.876	0.899	1.082	UP
TMED1	1.15	1.147	1.445	UP
TMED5	0.919	0.937	1.093	UP
FAM135A	1.317	1.348	1.576	UP
TMEM79	1.934	2.013	2.326	UP
OSBP2	0.852	0.899	1.033	UP
ADAM8	1.699	1.655	1.99	UP
GKAP1	0.884	0.991	1.2	UP
FOXN2	0.844	0.964	1.153	UP
C16orf61	0.862	0.979	1.178	UP
ATXN1	1.348	1.525	1.805	UP
STC1	1.917	2.201	2.687	UP
LOC157562	2.274	2.498	3.083	UP
PTAR1	0.874	0.976	1.15	UP
P2RY11	1.31	1.46	1.71	UP
MORG1	0.822	0.895	1.072	UP
FAM132A	2.02	2.229	2.655	UP
PHF1	1.324	1.469	1.705	UP
ESF1	1.121	1.236	1.42	UP
ZNF146	1.33	1.447	1.676	UP
MOSPD2	1.42	1.698	1.922	UP

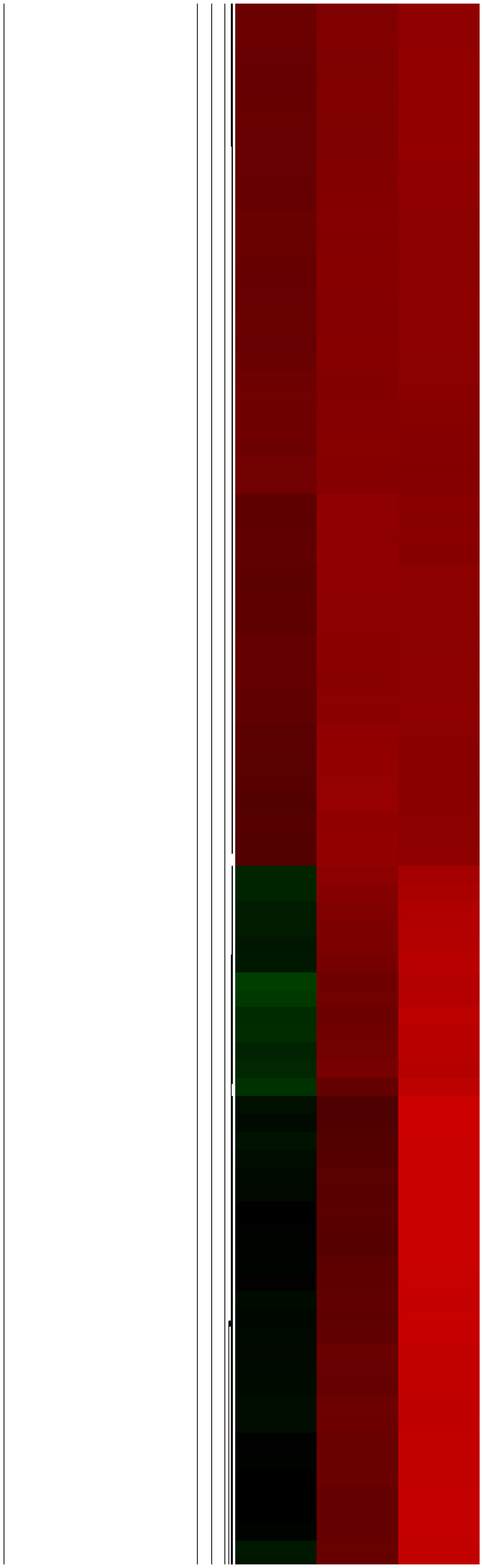

LIX1L	0.99	1.182	1.314	UP
TRIM23	1.446	1.772	1.946	UP
COL12A1	0.775	0.952	1.097	UP
C19orf61	2.143	2.683	3.082	UP
LOC100286937	1.157	1.47	1.654	UP
MMP14	0.866	1.058	1.208	UP
IFRD1	1.888	2.324	2.669	UP
UNC84B	1.562	1.921	2.242	UP
HSPBAP1	0.983	1.245	1.363	UP
CAV2	1.083	1.386	1.543	UP
OSTM1	0.885	1.147	1.243	UP
TTC33	1.176	1.474	1.557	UP
SCG2	0.928	1.169	1.246	UP
CA2	1.048	1.352	1.408	UP
ATF1	0.928	1.223	1.27	UP
QSOX2	1.096	1.427	1.488	UP
F2R	0.753	0.973	1.044	UP
CYCSP52	0.996	1.277	1.354	UP
LOC100131209	1.974	2.545	2.685	UP
CD55	1.014	1.302	1.345	UP
SMOX	2.826	3.386	3.589	UP
TMEM161B	0.853	0.984	1.019	UP
RPGR	1.462	1.754	1.791	UP
TMTC3	1.041	1.253	1.247	
LOC401218	0.901	1.122	1.097	
SLC25A37	1.228	1.452	1.423	
GUCA1B	2.223	2.58	2.597	UP
LOC644717	0.778	1.187	1.141	
C13orf15	0.824	1.245	1.184	
RND3	1.073	1.583	1.543	
BCAR3	0.946	1.412	1.314	
MFSD8	0.793	1.224	1.211	
EIF1B	0.862	1.332	1.32	
DAAM1	2.439	3.625	3.665	UP
C5orf32	0.775	1.159	1.154	
FAM82A1	1.334	1.848	1.851	UP
CAB39L	1.351	1.882	1.907	UP
LOC643037	0.761	1.023	1.064	UP
LOC100130178	1.176	1.699	1.709	UP
LETM2	1.699	2.412	2.489	UP
POLR2K	0.708	1.138	1.093	
TMEM38B	0.909	1.47	1.355	
YIPF4	1.275	2.111	1.98	
C6orf72	0.641	1.095	0.999	
C9orf91	0.864	1.585	1.453	
PTS	0.656	1.088	1.051	
FAS	1.325	2.299	2.191	
CREM	0.651	1.164	1.131	UP
FOXF2	-0.363	1.411	1.641	
COTL1	-0.227	0.823	1.055	
ZHX2	-0.177	0.801	1.094	
LOC283454	-0.237	1.065	1.521	
BTN2A2	-0.208	1.12	1.646	
AKAP2	-0.156	0.745	1.164	
SLC36A1	-0.347	0.613	1.005	
MARVELD2	-0.328	0.647	1.029	
SLC8A3	-0.296	0.75	1.312	
EXOC2	-0.277	0.697	1.142	
PGF	-0.354	1.167	1.878	
LOC100131726	-0.241	0.73	1.11	
LVRN	-0.356	0.72	1.356	
LARS2	-0.086	0.431	1.112	
HRH1	-0.078	0.588	1.525	
KIAA1949	-0.09	0.413	1.003	
B3GNT2	-0.077	0.502	1.186	
FHL2	-0.065	0.613	1.379	UP
FAM171A1	-0.057	0.583	1.337	UP
ZNF274	0.004	0.707	1.571	UP
WDR66	-0.008	0.614	1.401	UP
PGBD3	-0.014	0.519	1.209	UP
PHLDB3	-0.046	0.876	1.9	UP
FAM103A1	0.022	0.581	1.21	UP
PCGF2	-0.095	0.77	1.567	UP
DUSP2	-0.05	0.666	1.393	UP
ATP1B4	-0.048	0.51	1.041	UP
OCLN	-0.082	1.003	1.814	UP
BRPF3	-0.081	0.832	1.557	UP
UPF1	-0.105	0.95	1.83	UP
PRDM8	-0.103	0.831	1.445	
METRNL	-0.178	1.42	2.522	
ZNF202	-0.014	0.978	1.831	UP
CDK5R1	-0.019	0.599	1.108	UP
PKP2	-0.007	1.468	2.645	UP
UBE2E2	-0.003	0.578	1.123	UP
RELA	0.005	0.537	1.036	UP
GAN	-0.043	0.877	1.726	UP
TNRC6C	-0.125	0.544	1.007	
CCDC50	-0.131	0.613	1.169	

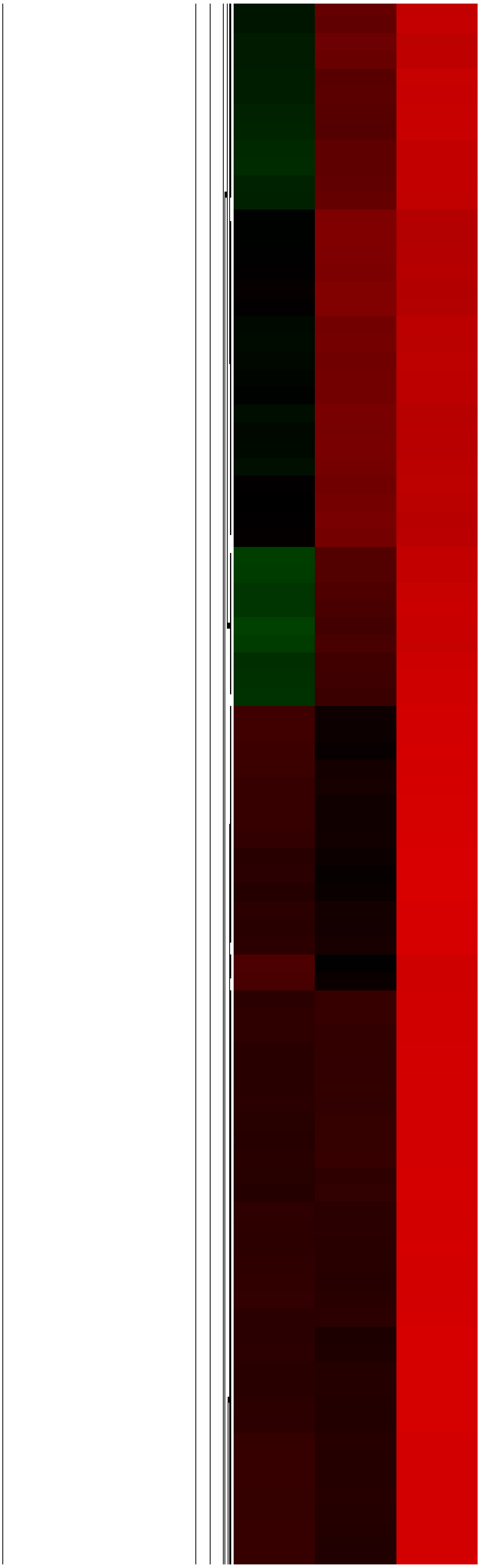

ARHGAP10	-0.112	0.5	1.01	
LOC400573	-0.155	0.618	1.072	
INA	-0.171	0.659	1.193	
HN1L	-0.16	0.478	1.074	
EHD4	-0.158	0.5	1.081	
OBFC2A	-0.337	0.844	1.927	
LOC387647	-0.255	0.611	1.428	
SLC8A2	-0.34	0.784	1.595	
GFRA1	-0.248	0.532	1.102	
TTTY15	-0.292	0.814	1.651	
TRAM2	-0.203	0.6	1.156	
ZNF251	-0.016	0.961	1.37	
LOC550112	-0.028	1.197	1.668	
KRTAP21-1	-0.011	0.829	1.176	
SLC5A12	0.029	1.656	2.437	
PEA15	0.084	1.689	2.339	
KRT80	0.021	1.128	1.567	
MPZL3	-0.103	1.241	2.027	
C19orf76	-0.067	0.702	1.135	
LOC100128813	-0.074	1.037	1.748	
PLD6	-0.061	1.326	2.161	
PGM5	-0.019	1.002	1.654	UP
CRYZL1	-0.105	0.898	1.356	
MEG8	-0.065	0.895	1.39	
ALDH1A3	-0.055	0.74	1.125	
NCRNA00086	-0.101	0.76	1.224	
TLE4	0.034	1.582	2.659	UP
FOXJ2	0.01	1.015	1.639	UP
TP53INP2	0.03	1.266	1.946	
CCK	0.026	0.659	1.043	UP
VPS37C	-0.368	0.488	1.153	
TNK1	-0.33	0.45	1.065	
RRP12	-0.327	0.491	1.256	
FJX1	-0.386	0.534	1.496	
PINX1	-0.332	0.35	1.037	
NFYB	-0.369	0.456	1.246	
RIMS4	-0.42	0.599	1.866	
SMAP2	-0.262	0.336	1.113	
IL12A	-0.269	0.319	1.128	
SAP18	0.303	0.07	1.025	
CFP	0.381	0.072	1.253	
PIP5K1A	0.338	0.048	1.172	
SYCE1	0.381	0.134	1.35	UP
FOS	1.259	0.523	4.872	UP
MTF2	0.281	0.083	1.093	UP
CRY2	0.621	0.183	2.434	UP
LOC728065	0.382	0.144	1.631	UP
HIVEP2	0.355	0.103	1.857	UP
C12orf67	0.212	0.038	1.044	UP
LOC100129617	0.182	0.05	1.079	UP
MAP3K7IP2	0.217	0.108	1.074	UP
FAM108B1	0.256	0.129	1.331	UP
ZUFSP	0.31	0.166	1.482	UP
SGK1	0.472	-0.009	1.276	
OR2D3	0.381	0.053	1.076	
SERTAD2	0.362	0.462	1.741	UP
HOXB3	0.363	0.428	1.623	UP
DKFZp451A211	0.268	0.286	1.2	UP
RBBP5	0.301	0.383	1.566	UP
FUBP3	0.281	0.355	1.482	UP
RIMKLB	0.465	0.57	2.355	UP
CNOT4	0.218	0.25	1.058	UP
MYSM1	0.386	0.521	2.08	UP
GORASP1	0.272	0.372	1.484	UP
RLIM	0.323	0.427	1.657	UP
PDE10A	0.285	0.34	1.52	UP
BRIX1	0.192	0.262	1.129	UP
PRPF38B	0.458	0.414	2.077	UP
MURC	0.218	0.204	1.004	UP
LOC387810	0.3	0.268	1.395	UP
ZNF474	0.382	0.323	1.697	UP
ATE1	0.239	0.19	1.034	UP
C4orf29	0.319	0.266	1.344	UP
KIFC1	0.293	0.311	1.468	UP
SPRY2	0.251	0.167	1.272	UP
FLJ42627	0.3	0.213	1.446	UP
ZNF367	0.476	0.428	2.429	UP
LOC100131176	0.27	0.24	1.4	UP
KRT25	0.507	0.398	2.374	UP
ACTA1	0.261	0.207	1.259	UP
LOC727878	0.406	0.296	1.612	UP
LOC100128460	0.279	0.19	1.069	UP
SFRS11	0.384	0.253	1.471	UP
RPS6KA6	0.351	0.254	1.397	UP
RNMT	0.376	0.265	1.482	UP
PTPRH	0.498	0.309	1.889	UP
ARID3C	0.326	0.19	1.229	UP
DNAJC27	0.28	0.179	1.105	UP

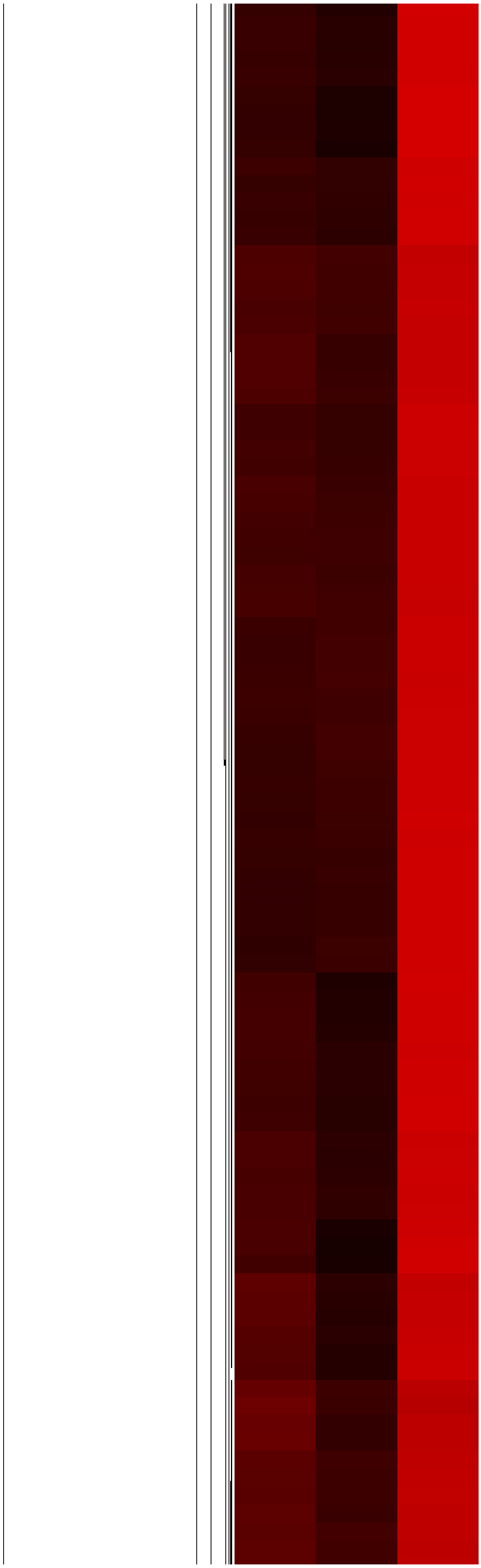

C21orf89	0.443	0.311	1.627	UP
ASMT	0.925	0.645	3.419	UP
SRGAP1	0.269	0.199	1.024	UP
SON	0.401	0.293	1.446	UP
NFKBID	0.716	0.401	2.854	UP
ARHGAP23	0.415	0.237	1.699	UP
TAF7	0.28	0.17	1.142	UP
C17orf74	0.285	0.145	1.149	UP
LOC729130	0.348	0.286	1.193	UP
NRL	0.378	0.357	1.506	UP
CLDN15	0.467	0.403	1.727	UP
MLL	0.354	0.296	1.335	UP
KIF2C	0.317	0.256	1.17	UP
TSHZ2	0.595	0.511	1.494	UP
GORAB	0.433	0.361	1.088	UP
C2orf24	0.593	0.497	1.505	UP
SASS6	0.607	0.527	1.653	UP
MCAM	0.596	0.508	1.566	UP
LIN7B	0.57	0.387	1.387	UP
CASP3	0.694	0.478	1.691	UP
C3orf71	0.428	0.304	1.051	UP
DCUN1D2	0.575	0.438	1.47	UP
UBR5	0.401	0.345	1.315	UP
DCAF15	0.447	0.375	1.467	UP
LEMD1	0.428	0.332	1.297	UP
C7orf40	0.639	0.546	1.998	UP
HELQ	0.571	0.45	1.57	UP
FBXO11	0.404	0.338	1.133	UP
STAT3	0.47	0.438	1.43	UP
SEC24A	0.504	0.486	1.561	UP
CCDC45	0.535	0.529	1.694	UP
WEE1	0.663	0.581	1.957	UP
STAM	0.642	0.578	1.84	UP
SFRS12	0.549	0.516	1.557	UP
SNX31	0.476	0.531	1.652	UP
INTS6	0.568	0.652	2.002	UP
FAM126A	0.305	0.36	1.093	UP
FMR1	0.305	0.354	1.049	UP
SELV	0.366	0.373	1.219	UP
ERCC6	0.435	0.465	1.522	UP
TET2	0.52	0.628	1.931	UP
RRAD	0.643	0.801	2.451	UP
FRK	0.361	0.439	1.415	UP
ZBTB48	0.46	0.511	1.731	UP
PPP2CA	0.345	0.399	1.345	UP
EPB41L2	0.495	0.57	1.972	UP
JMY	0.292	0.307	1.085	UP
RNF103	0.315	0.332	1.255	UP
OVGP1	0.656	0.73	2.646	UP
MGC16142	0.448	0.492	1.826	UP
CCNT1	0.495	0.525	1.986	UP
SMNDC1	0.29	0.316	1.172	UP
MAGEA12	0.248	0.312	1.077	UP
EIF2C4	0.404	0.479	1.687	UP
ABL1	0.353	0.172	1.147	UP
AHRR	0.322	0.168	1.011	UP
RSRC2	0.946	0.476	2.843	UP
OAZ3	0.375	0.198	1.135	UP
C16orf46	0.668	0.435	2.05	UP
PITPNB	0.316	0.215	1.021	UP
DNAJC2	0.322	0.213	1.048	UP
ZNF389	0.485	0.314	1.633	UP
USPL1	0.355	0.221	1.173	UP
HCFC2	0.549	0.325	1.476	UP
C8orf46	0.424	0.246	1.161	UP
DUSP14	0.653	0.413	1.871	UP
WWTR1	0.511	0.345	1.439	UP
RBMS3	0.47	0.309	1.316	UP
TLK1	0.376	0.13	1.021	
LOC100130465	0.381	0.121	1.069	
CRABP2	0.736	0.286	2.338	UP
FAM90A1	0.572	0.271	1.178	
SGMS2	1.18	0.521	2.57	
NCRNA00174	0.62	0.265	1.348	
TEF	0.972	0.489	2.25	UP
USP19	0.525	0.227	1.26	
CCNL2	0.81	0.37	2.032	UP
TBC1D23	0.568	0.346	1.041	UP
CLEC2A	0.656	0.348	1.097	
NARG1L	0.638	0.315	1.166	
GAS2L3	0.838	0.405	1.516	
PER1	2.221	1.522	4.681	UP
UTP11L	0.537	0.369	1.158	UP
CLEC3B	0.491	0.332	1.068	UP
FRYL	0.499	0.312	1.034	UP
BAZ1A	0.58	0.438	1.237	UP
RPRD1B	0.769	0.544	1.558	UP
CES4	0.836	0.586	1.721	UP

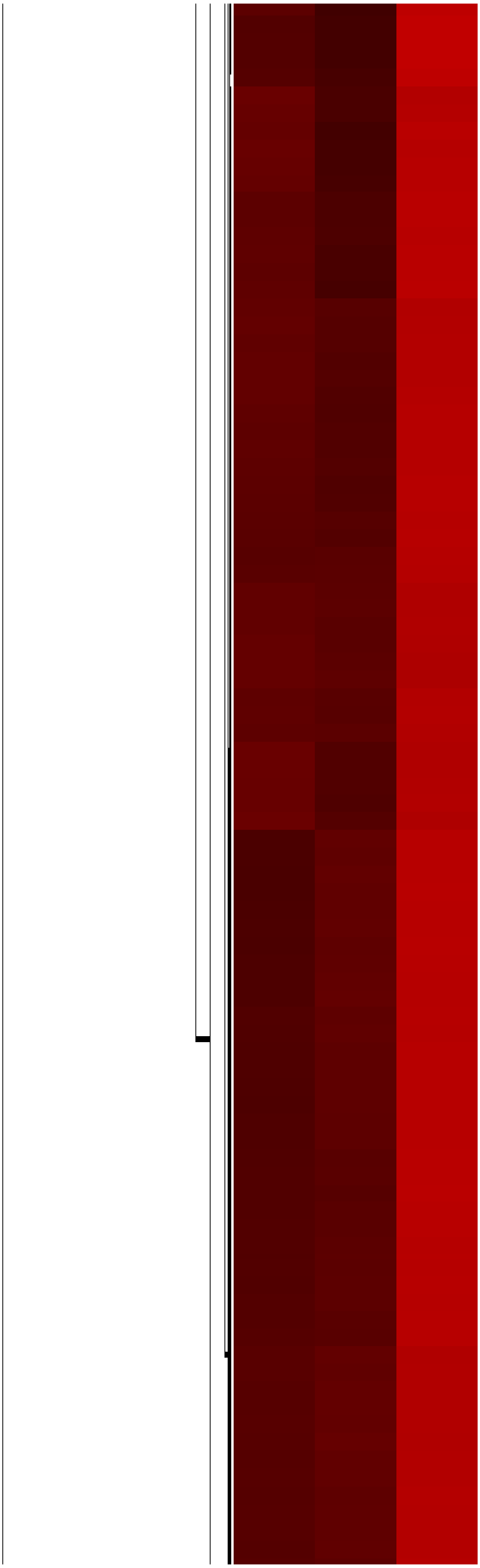

LOC100134372	0.598	0.489	1.406	UP
ID4	1.034	0.827	2.381	UP
ARID4A	0.687	0.556	1.592	UP
ING1	0.708	0.595	1.58	UP
NDC80	0.767	0.542	1.297	UP
LMAN1	0.755	0.548	1.334	UP
TBK1	0.647	0.435	1.197	UP
PRKAR1A	0.867	0.574	1.509	UP
NPHP3	0.654	0.443	1.17	UP
REST	0.722	0.511	1.313	UP
CXCL3	1.128	0.931	2.271	UP
BAG4	0.591	0.493	1.187	UP
GPR64	0.606	0.494	1.176	UP
LOC100129652	0.517	0.397	1.003	UP
ADAM17	0.718	0.566	1.428	UP
FAM193B	1.58	1.167	3.097	UP
CBFA2T2	0.582	0.519	1.059	UP
ATAD2B	1.16	0.997	2.086	UP
ZBTB2	1.15	1.02	2.135	UP
REV1	1.196	1.002	2.179	UP
GOLGA8F	0.819	0.707	1.491	UP
SECISBP2L	0.991	0.813	1.806	UP
ZNF451	0.598	0.504	1.142	UP
PPP1R12A	0.57	0.501	1.111	UP
CAB39	0.692	0.59	1.314	UP
CDC37L1	0.799	0.716	1.553	UP
ZNF484	1.417	1.225	2.811	UP
CBFB	0.505	0.448	1.014	UP
TGFB2	0.746	0.712	1.492	UP
CDKN2D	0.908	0.848	1.853	UP
ZCCHC10	0.546	0.556	1.13	UP
FAM40A	0.522	0.532	1.055	UP
LOC283663	1.666	1.575	3.01	UP
CCDC13	0.612	0.578	1.094	UP
SMARCA4	1.01	0.933	1.84	UP
NR4A3	0.798	0.713	1.398	UP
SCAPER	0.713	0.65	1.227	UP
LOC100216546	0.939	0.881	1.619	UP
STK17B	0.829	0.775	1.561	UP
FAM160B1	0.895	0.826	1.686	UP
SNHG1	0.932	0.912	1.771	UP
H2BFWT	0.701	0.543	1.168	UP
LOC144438	0.676	0.526	1.136	UP
AVPI1	1.064	0.835	1.824	UP
UBD	0.734	0.569	1.27	UP
CIR1	1.151	0.914	1.886	UP
PLEKHA7	0.508	0.654	1.228	UP
NFAT5	0.997	1.262	2.42	UP
KIAA0907	0.664	0.882	1.645	UP
EDN2	1.473	1.918	3.649	UP
ZNF330	0.445	0.572	1.084	UP
ZEB1	0.76	0.967	1.821	UP
SRFBP1	0.711	0.888	1.732	UP
PPP3R1	0.552	0.681	1.305	UP
PPTC7	0.782	0.982	1.833	UP
DDX21	0.567	0.726	1.326	UP
WDR20	0.943	1.1	2.118	UP
EFNA1	1.126	1.345	2.532	UP
ZNF639	0.737	0.873	1.705	UP
LOC729806	0.521	0.616	1.198	UP
CXCL1	1.273	1.533	2.989	UP
ATP2A1	0.679	0.831	1.61	UP
C14orf138	1.183	1.399	2.733	UP
ADPGK	0.496	0.583	1.149	UP
FAM91A1	0.896	0.99	2.068	UP
AAGAB	0.689	0.757	1.572	UP
TMEM81	0.655	0.701	1.5	UP
TRAF2	0.659	0.725	1.49	UP
PLK4	0.709	0.77	1.587	UP
TMEFF1	0.641	0.711	1.415	UP
REV3L	0.836	0.949	1.881	UP
RBM5	0.647	0.712	1.41	UP
SETD2	0.887	0.949	1.945	UP
LOC100128727	0.866	0.898	1.865	UP
NR4A2	0.63	0.7	1.261	UP
GGA3	0.556	0.603	1.113	UP
LIN52	1.086	1.245	2.229	UP
CSNK1A1	0.504	0.581	1.032	UP
THUMPD2	1.008	1.143	2.052	UP
PANK4	0.617	0.73	1.262	UP
SETD8	0.561	0.634	1.169	UP
SENP7	0.76	0.863	1.577	UP
RAGE	0.947	1.05	2.005	UP
KDM3A	0.89	0.98	1.845	UP
KLHL11	0.648	0.718	1.35	UP
CRY1	1.307	1.498	2.776	UP
TMC7	0.591	0.631	1.239	UP
TBC1D15	1.012	1.109	2.145	UP

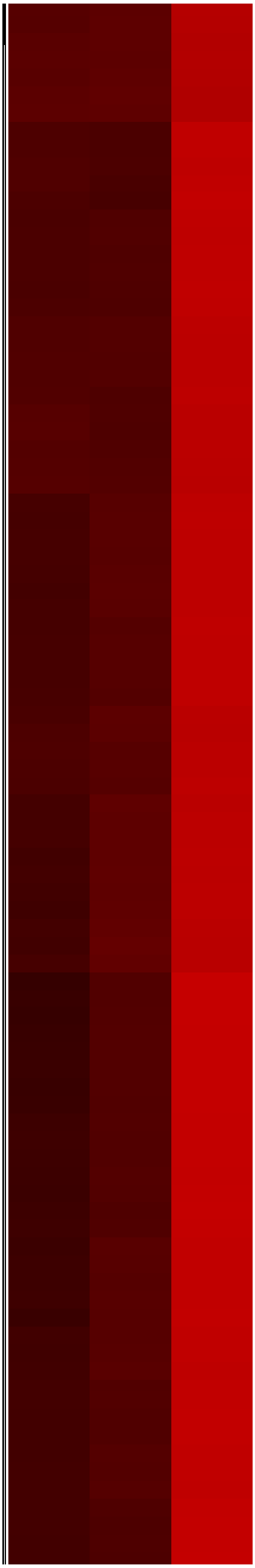

COL4A3BP	0.861	0.904	1.719	UP
C1orf124	0.613	0.631	1.217	UP
RGPD5	0.753	0.798	1.517	UP
TERF2IP	1.237	1.307	2.401	UP
LOC100129138	1.062	1.083	2.015	UP
RSF1	0.801	0.783	1.991	UP
TLR6	0.896	0.865	2.192	UP
G2E3	0.824	0.779	1.954	UP
RABGAP1	0.429	0.388	1.02	UP
C6orf204	0.408	0.382	1.031	UP
SNHG12	0.964	1.038	2.466	UP
PPM1A	0.549	0.598	1.395	UP
ERO1LB	0.735	0.772	1.852	UP
CXCL2	0.848	0.903	2.162	UP
USP12	0.74	0.784	1.904	UP
RAB2B	0.452	0.465	1.127	UP
TMEM41B	0.426	0.443	1.002	UP
JUND	0.599	0.616	1.392	UP
C9orf102	0.638	0.653	1.48	UP
C11orf30	0.552	0.572	1.288	UP
RGS2	0.769	0.745	1.793	UP
OCLM	0.519	0.477	1.122	UP
MBNL3	0.767	0.692	1.636	UP
PHTF2	0.457	0.439	1.025	UP
CSF2	1.676	1.638	3.723	UP
HTR1D	0.515	0.502	1.111	UP
TM2D3	0.386	0.478	1.035	UP
SMC5	0.68	0.864	1.86	UP
SLU7	0.476	0.597	1.28	UP
PHLDA2	0.68	0.841	1.817	UP
LOC728836	0.527	0.683	1.447	UP
KBTBD2	0.621	0.83	1.733	UP
TRIML2	0.917	1.185	2.513	UP
ITSN2	0.477	0.578	1.305	UP
ID2	0.921	1.126	2.486	UP
ZNF654	0.947	1.151	2.542	UP
ZBTB17	0.453	0.553	1.235	UP
TAF2	0.389	0.455	1.038	UP
TTLL3	0.539	0.676	1.371	UP
TMEM18	0.496	0.588	1.193	UP
RIT1	1.192	1.315	2.866	UP
MYNN	0.747	0.855	1.822	UP
MED28	0.5	0.567	1.262	UP
SMURF1	0.629	0.874	1.71	UP
SCYL2	0.66	0.894	1.807	UP
FBXO33	0.69	0.931	1.85	UP
MAPKSP1	0.565	0.816	1.623	UP
CYP27B1	0.612	0.87	1.706	UP
LOC100132541	0.645	0.938	1.862	UP
ARL13B	0.85	1.27	2.509	UP
DGKE	0.369	0.54	1.026	UP
DCP1A	0.758	1.158	2.149	UP
PPP3CC	0.555	0.775	1.467	UP
MSX1	0.418	0.631	1.536	UP
FERMT2	0.703	1.022	2.478	UP
WDR45L	0.373	0.563	1.341	UP
NR1D1	1.22	1.819	4.271	UP
KLK13	0.314	0.452	1.062	UP
FNIP1	0.756	1.062	2.52	UP
FBXO3	0.587	0.861	2.041	UP
CARD9	0.39	0.55	1.325	UP
GATA6	0.912	1.209	2.881	UP
ARRDC2	0.534	0.7	1.67	UP
RBM33	0.559	0.739	1.771	UP
SIRT1	0.4	0.551	1.279	UP
MAP2K3	0.852	1.188	2.812	UP
NUP50	0.555	0.721	1.775	UP
LOC100288600	0.477	0.614	1.489	UP
IER2	0.78	1.149	2.516	UP
GAPVD1	0.422	0.597	1.326	UP
TSC1	0.545	0.756	1.705	UP
PPP1R15B	0.526	0.738	1.635	UP
ZBTB34	0.444	0.664	1.494	UP
MCC	0.382	0.508	1.129	UP
KDM5C	0.596	0.807	1.782	UP
ZNF800	0.397	0.547	1.164	UP
ZNF653	0.414	0.502	1.193	UP
RYBP	0.64	0.782	1.841	UP
FUT3	0.476	0.582	1.4	UP
CCNC	0.369	0.446	1.071	UP
MYLIP	0.544	0.691	1.57	UP
ADH7	0.698	0.863	1.982	UP
SYDE2	0.812	1.039	2.318	UP
FIGN	0.705	0.826	2.021	UP
C2orf69	0.361	0.416	1.049	UP
AMMECR1L	0.464	0.545	1.359	UP
MARCH7	0.565	0.662	1.678	UP
UBE2H	0.39	0.463	1.144	UP

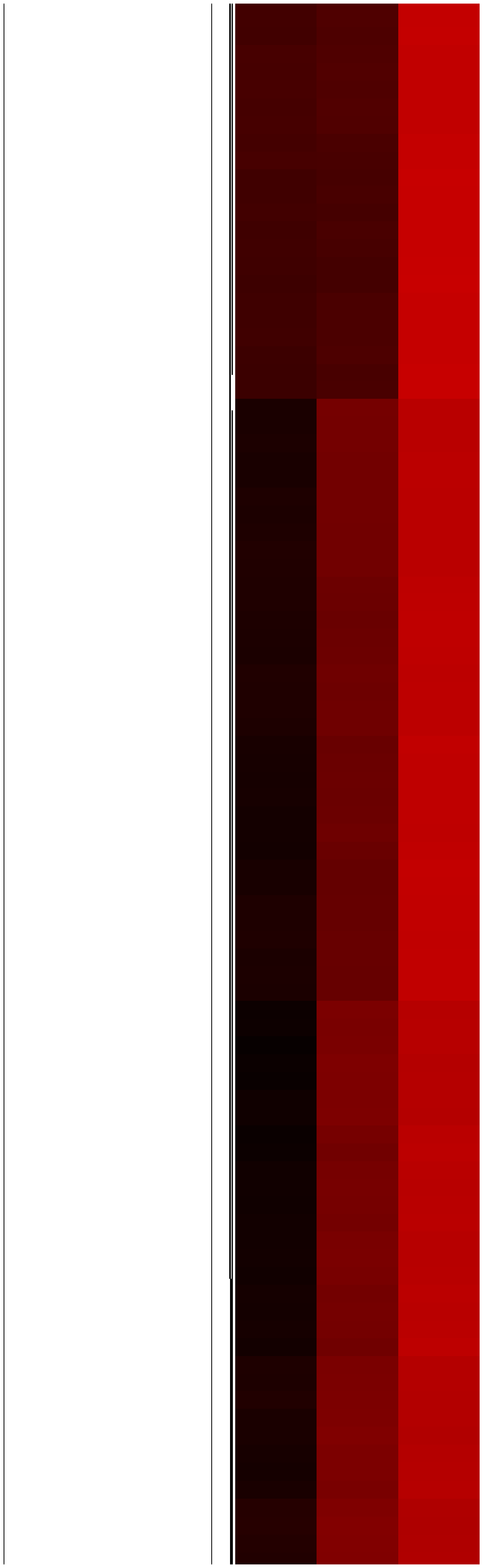

PPIL4	0.344	0.405	1.029	UP
GOLGA2	0.591	0.661	1.608	UP
GCH1	0.412	0.478	1.136	UP
SMPD1	0.592	0.66	1.611	UP
QKI	0.607	0.704	1.676	UP
ODF2	0.457	0.521	1.259	UP
IL6ST	0.492	0.534	1.402	UP
FRS2	0.409	0.417	1.127	UP
NCRNA00160	0.41	0.444	1.258	UP
DSCR9	0.379	0.423	1.161	UP
NGFR	0.502	0.525	1.49	UP
SLC16A7	0.354	0.408	1.1	UP
RFX1	0.514	0.573	1.597	UP
PPAP2B	0.598	0.64	1.871	UP
LOC256880	0.326	0.359	1.058	UP
PRH2	0.427	0.5	1.327	UP
CDKN2AIP	0.816	0.967	2.525	UP
WAC	0.449	0.524	1.365	UP
SPAG9	0.583	0.742	1.903	UP
NBR1	0.315	0.379	1.048	UP
FBXW7	0.595	0.736	2.009	UP
TOLLIP	0.24	1.067	1.688	UP
TMEM194B	0.192	0.854	1.357	UP
ST7L	0.157	0.642	1.017	UP
TCF12	0.164	0.736	1.205	UP
CYTH3	0.178	0.788	1.29	UP
PPP1R1C	0.342	1.304	2.117	UP
NKX3-1	0.179	0.713	1.165	UP
ZNF776	0.35	1.292	2.13	UP
RAPGEF6	0.21	0.705	1.162	UP
ARL8B	0.294	1.011	1.661	UP
UPF2	0.216	0.736	1.292	UP
SLC30A7	0.247	0.836	1.487	UP
CBL	0.241	0.851	1.537	UP
BTBD10	0.309	1.184	2.108	UP
MRPL42P5	0.313	1.227	2.143	UP
RARA	0.183	0.62	1.05	UP
PPP2CB	0.234	0.804	1.394	UP
ZNF136	0.457	1.635	2.762	UP
C1orf69	0.483	1.838	3.114	UP
RFX6	0.217	0.919	1.705	UP
SLC31A2	0.14	0.645	1.159	UP
LOC100133659	0.141	0.664	1.187	UP
KCTD5	0.223	1.009	1.817	UP
STAM2	0.121	0.625	1.112	UP
CAPN6	0.114	0.616	1.065	UP
M6PR	0.115	0.62	1.13	UP
TNS1	0.184	0.753	1.472	UP
PPP1R13L	0.347	1.413	2.737	UP
SHOC2	0.213	0.713	1.353	UP
FOSL1	0.68	2.255	4.329	UP
LIG4	0.238	0.789	1.459	UP
SCN3B	0.228	0.856	1.6	UP
MAP4K3	0.252	0.895	1.681	UP
CHIC2	0.315	1.196	2.251	UP
SEMA7A	0.079	0.783	1.163	UP
OR7E104P	0.073	0.67	1.013	UP
SRF	0.103	1.588	2.382	
ZNF75A	0.077	0.852	1.218	
TSPAN5	0.098	1.339	1.947	
PLA2G4C	0.312	2.349	3.41	UP
FUNDC2	0.132	1.014	1.458	UP
TRIM38	0.081	0.929	1.466	UP
OLFM2	0.146	1.339	2.222	UP
HCCS	0.095	0.658	1.033	UP
FAM168B	0.149	1.009	1.561	UP
ZNF407	0.156	1.123	1.754	UP
ZNF697	0.237	1.507	2.411	UP
TMEM88	0.225	1.497	2.25	UP
ADAMTS16	0.109	0.702	1.052	UP
ZNF276	0.112	0.768	1.169	UP
SYS1	0.149	0.785	1.274	UP
LOC100291714	0.113	0.642	1.012	UP
GOT1	0.145	0.757	1.214	UP
STX3	0.403	2.316	3.892	UP
GMEB1	0.207	0.835	1.231	UP
ADRB2	0.494	2.038	2.976	UP
IL32	0.542	2.015	2.901	UP
HRK	0.387	1.808	2.596	UP
ARHGEF15	0.23	1.1	1.522	UP
VWCE	0.174	0.917	1.329	UP
PFKFB4	0.314	1.788	2.612	UP
TNFRSF10C	0.243	1.142	1.725	UP
MAP1LC3A	0.231	0.785	1.08	UP
CLLU1OS	0.229	0.774	1.034	UP
MYO5B	0.329	1.203	1.633	UP
ADRB1	0.317	1.226	1.679	UP
MAP1LC3B	0.389	1.409	1.871	UP

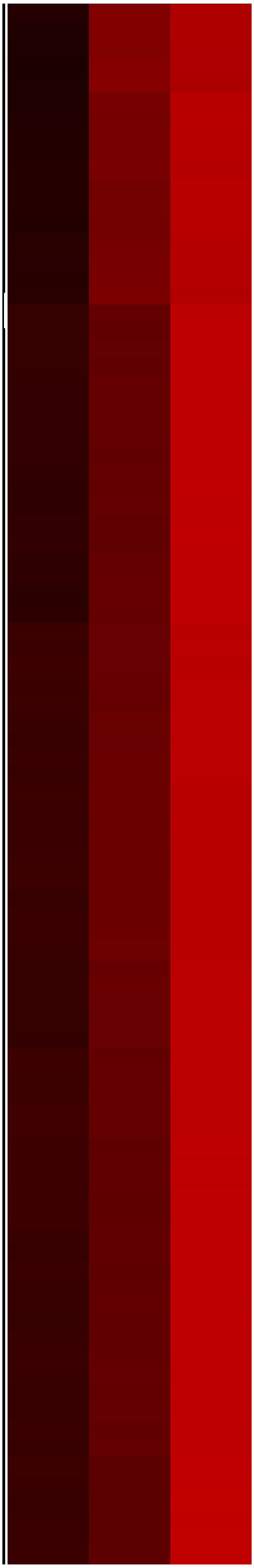

ZIC2	0.246	0.986	1.357	UP
CCDC29	0.392	1.642	2.225	UP
MED10	0.333	1.552	2.034	UP
CDC42EP1	0.341	1.478	1.893	UP
PHLDA3	0.214	0.799	1.223	UP
AIFM2	0.186	0.666	1.054	UP
RAB32	0.228	0.772	1.177	UP
C11orf68	0.203	0.701	1.076	UP
ITGA5	0.286	0.961	1.431	UP
TAF1A	0.518	1.643	2.664	UP
HAS2	0.647	2.071	3.269	UP
FLJ31813	0.226	0.732	1.176	UP
NECAP1	0.404	1.201	1.875	UP
EHD1	0.442	1.27	1.921	UP
ARID3A	0.49	1.539	2.317	UP
TMEM27	0.542	1.614	2.354	UP
UGCG	1.035	1.937	3.702	UP
SPEN	0.431	0.799	1.557	UP
GEM	0.965	1.832	3.515	UP
FGFR1OP	0.577	1.081	2.115	UP
RBM18	0.283	0.558	1.035	UP
NR1D2	0.624	1.226	2.289	UP
FAM83G	0.587	1.183	2.214	UP
BTN2A1	0.551	1.084	2.078	UP
ZFP36L1	0.383	0.75	1.466	UP
THAP1	0.571	1.164	2.244	UP
LATS2	0.585	1.215	2.378	UP
CHD7	0.398	0.845	1.63	UP
ZNF529	0.584	1.151	2.294	UP
TXNL4B	0.87	1.685	3.323	UP
SMN1	0.297	0.64	1.197	UP
NUDCD3	0.342	0.724	1.365	UP
SBNO2	0.272	0.635	1.183	UP
BACH1	0.365	0.816	1.539	UP
NFKBIB	0.894	1.575	2.828	UP
CTDSPL2	0.419	0.754	1.355	UP
TUBA4A	0.811	1.394	2.503	UP
RRAS2	0.562	0.991	1.806	UP
PDPK1	0.476	0.815	1.495	UP
TICAM1	0.776	1.446	2.575	UP
TBX2	0.588	1.091	1.968	UP
HOXC13	0.506	0.941	1.678	UP
BNC1	0.58	1.107	1.966	UP
LOC145814	0.464	0.855	1.472	UP
JUNB	0.843	1.574	2.75	UP
UBTD1	0.346	0.631	1.105	UP
FAF2	0.336	0.621	1.072	UP
ACSL4	0.484	0.853	1.5	UP
PARD6B	0.44	0.795	1.361	UP
SPRY4	0.465	0.909	1.554	UP
POLH	0.358	0.671	1.161	UP
MEX3C	0.307	0.591	1.007	UP
DUSP1	0.762	1.499	2.521	UP
KLF7	0.373	0.709	1.301	UP
KCNF1	0.512	1.015	1.824	UP
TAF13	0.507	0.999	1.822	UP
PI4KAP2	0.325	0.626	1.137	UP
PI4K2A	0.686	1.369	2.487	UP
SNX9	0.713	1.141	2.18	UP
SFXN1	0.354	0.593	1.124	UP
MAFF	1.623	2.708	5.074	UP
LOC286109	0.788	1.267	2.365	UP
ZNF143	0.841	1.372	2.518	UP
RIOK3	0.682	1.072	2.125	UP
CDK6	0.556	0.888	1.724	UP
TGM2	0.532	0.829	1.623	UP
DNAJB9	0.398	0.629	1.266	UP
CLCF1	0.947	1.463	2.935	UP
MXD1	1.043	1.778	3.48	UP
MTX3	0.612	1.055	2.084	UP
PTRH2	0.37	0.625	1.258	UP
CA5BP	0.419	0.739	1.468	UP
ATG12	0.573	1.028	1.986	UP
VKORC1L1	0.352	0.636	1.256	UP
COQ10B	0.611	1.042	2.027	UP
CCNJ	0.401	0.672	1.294	UP
PCTK2	0.577	1.03	1.944	UP
NOC3L	0.487	0.871	1.681	UP
JAZF1	0.699	1.258	2.376	UP
DUSP6	0.674	1.178	2.285	UP
ZNF383	0.47	0.86	1.632	UP
PRDM1	0.769	1.168	2.493	UP
LDLRAD3	0.354	0.548	1.156	UP
ZCCHC8	0.387	0.628	1.339	UP
TFAM	0.522	0.848	1.777	UP
SBSN	0.967	1.557	3.296	UP
PRR4	0.383	0.606	1.263	UP
ZNF267	0.51	0.838	1.76	UP

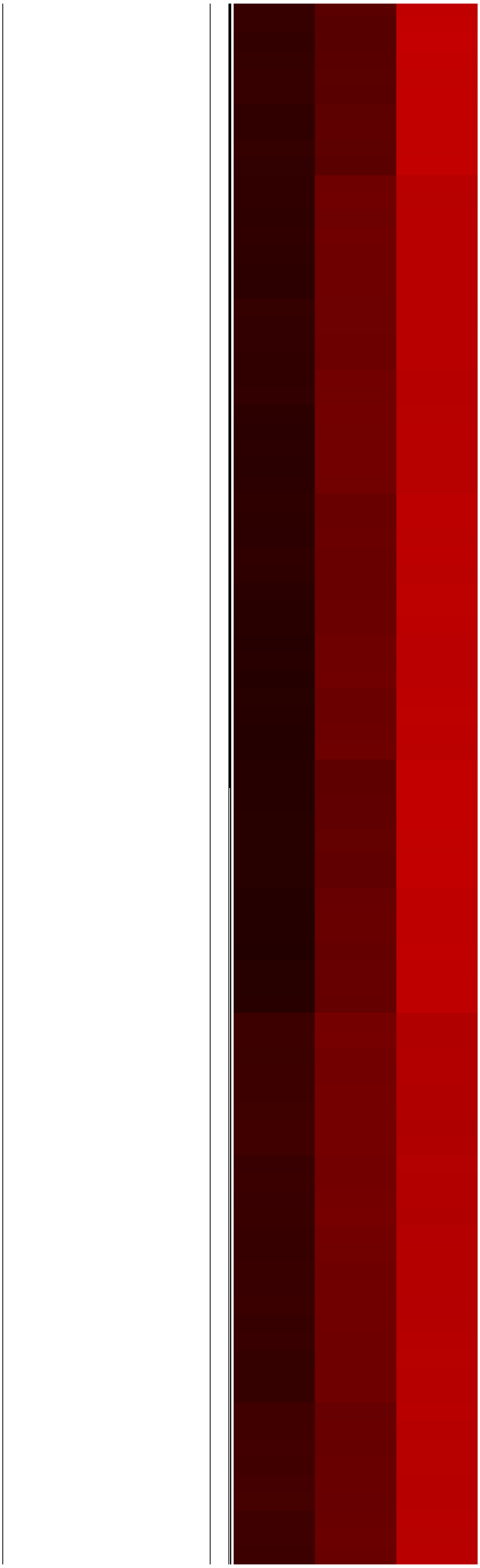

PAN3	0.428	0.688	1.553	UP
TRAF4	0.265	0.443	1.003	UP
GTPBP1	0.518	0.873	1.925	UP
DKFZp667E0512	0.326	0.527	1.138	UP
CSRNP1	1	1.618	3.585	UP
SSH1	0.599	1.16	2.412	UP
OMP	0.283	0.553	1.135	UP
TOPORS	0.345	0.613	1.28	UP
PPP1R15A	1.42	2.617	5.596	UP
BRSK1	0.288	0.662	1.104	UP
AEN	0.963	2.233	3.649	UP
PRKAB1	0.586	1.353	2.283	UP
LOC652636	0.273	0.631	1.036	UP
CDC42SE1	0.594	1.39	2.29	UP
ZRANB1	0.479	1.169	1.936	UP
FRMD6	0.817	2.022	3.394	UP
ZNF212	0.353	0.739	1.254	UP
MAP3K14	0.966	2.121	3.57	UP
LEMD2	0.416	0.892	1.529	UP
AMHR2	0.446	1.003	1.713	UP
VSTM1	0.376	0.89	1.422	UP
SMAD7	0.399	0.903	1.454	UP
OTUD7B	0.361	0.909	1.46	UP
NRAS	0.362	0.936	1.515	UP
IL11	0.808	2.1	3.363	UP
C6orf150	0.319	0.837	1.341	UP
PAX5	0.326	0.831	1.342	UP
EIF2C3	0.254	0.579	1.029	UP
C13orf1	0.259	0.602	1.08	UP
PIM3	0.587	1.404	2.453	UP
RAP2C	0.376	0.809	1.459	UP
LRRC8C	0.674	1.52	2.696	UP
IGF2BP2	0.599	1.488	2.684	UP
DSCR3	0.32	0.84	1.5	UP
PEX13	0.352	0.896	1.587	UP
NARG1	0.293	0.854	1.424	UP
ARRDC4	0.695	1.897	3.197	UP
ZEB2	0.434	1.32	2.197	UP
ZNF674	0.392	1.078	1.888	UP
PLIN3	0.215	0.59	1.047	UP
ZCCHC6	0.442	1.272	2.2	UP
ORAOV1	0.386	1.159	1.958	UP
RNF182	0.322	0.79	1.636	UP
FAM66D	0.228	0.559	1.157	UP
SLC41A2	0.243	0.624	1.236	UP
RSBN1	0.249	0.615	1.243	UP
PGM5P1	0.622	1.579	3.061	UP
FGD4	0.435	1.073	2.133	UP
VWC2L	0.262	0.649	1.299	UP
KLF4	0.788	2.268	4.175	UP
GJB3	0.487	1.382	2.526	UP
GAR1	0.236	0.684	1.247	UP
ARID1A	0.253	0.733	1.388	UP
PCF11	0.333	0.866	1.595	UP
CCNH	0.421	1.083	2.026	UP
POGZ	0.251	0.634	1.199	UP
TMEM194A	0.442	0.848	1.282	UP
NCRNA00152	0.725	1.402	2.085	UP
MFAP3	0.475	0.92	1.416	UP
DDX3X	0.398	0.745	1.166	UP
MRPL49	0.496	0.933	1.419	UP
JUN	1.205	2.221	3.334	UP
IL27RA	0.586	1.06	1.594	UP
DHX35	0.403	0.737	1.109	UP
UBE2S	0.469	0.959	1.482	UP
LYST	0.95	1.873	2.896	UP
RELB	0.879	1.832	2.787	UP
LOC652191	0.678	1.391	2.088	UP
LRRC8A	0.622	1.284	2.033	UP
BNIP1	0.437	0.901	1.441	UP
SHB	0.366	0.713	1.167	UP
MED15	0.425	0.837	1.353	UP
TNRC6A	0.383	0.738	1.181	UP
UBE2W	0.514	1.034	1.679	UP
TUBA3C	0.76	1.476	2.439	UP
KIAA1688	0.286	0.604	1.001	UP
AMOTL2	0.682	1.419	2.32	UP
TUBA8	0.729	1.51	2.467	UP
YES1	0.395	0.634	1.133	UP
OXTR	1.134	1.807	3.14	UP
NFKB2	1.141	1.752	3.138	UP
MIER1	0.529	0.844	1.489	UP
ORAI1	0.539	0.824	1.444	UP
CTGF	1.132	1.677	2.947	UP
DDIT3	1.669	2.789	4.907	UP
BTG3	0.471	0.803	1.382	UP
TES	0.472	0.804	1.411	UP
NEU1	0.516	0.875	1.566	UP

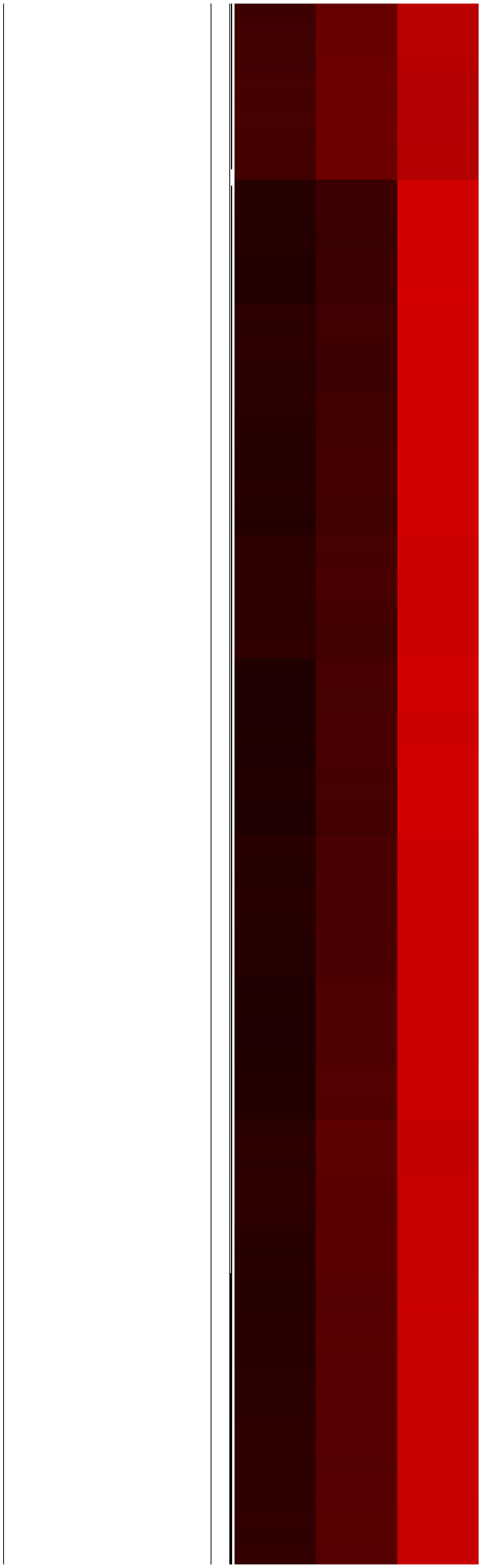

EGFR	0.693	1.155	2.031	UP
TNNC2	0.741	1.244	2.151	UP
GNAQ	0.571	0.978	1.655	UP
HERC4	0.395	0.611	1.015	UP
AVL9	0.528	0.831	1.391	UP
MTO1	0.596	0.936	1.544	UP
RBBP6	0.906	1.489	2.453	UP
ABCE1	0.472	0.767	1.296	UP
RAB3GAP1	0.414	0.692	1.121	UP
ZNF498	0.209	0.322	1.116	UP
KLHL18	0.299	0.459	1.592	UP
TAF5	0.351	0.548	1.969	UP
HTR1A	0.391	0.579	2.12	UP
WBP11	0.178	0.308	1.074	UP
FRG2	0.174	0.295	1.014	UP
PAK1IP1	0.249	0.42	1.519	UP
DUSP10	0.67	0.942	3.117	UP
BCL10	0.426	0.62	2.007	UP
CCNL1	0.667	0.91	3.082	UP
ZFX	0.292	0.426	1.444	UP
DDAH1	0.312	0.431	1.507	UP
TBC1D10A	0.306	0.466	1.499	UP
SPTY2D1	0.261	0.417	1.334	UP
KDM5D	0.31	0.517	1.631	UP
ELF1	0.23	0.41	1.259	UP
SOCS2	0.34	0.622	1.918	UP
SCRN1	0.312	0.515	1.567	UP
EXOC3L2	0.765	1.33	4.344	UP
CLK3	0.179	0.329	1.041	UP
IL6R	0.343	0.525	1.545	UP
C11orf82	0.359	0.54	1.625	UP
C3orf35	0.486	0.756	2.115	UP
BLZF1	0.455	0.692	1.995	UP
TSC22D2	0.517	0.754	2.334	UP
KIAA1432	0.28	0.393	1.223	UP
CYLD	0.468	0.631	1.963	UP
LOC100127955	0.508	1.056	3.11	UP
IL1A	0.284	0.627	1.85	UP
PXN	0.258	0.589	1.771	UP
BTN2A3	0.178	0.41	1.153	UP
ARC	0.781	1.692	4.797	UP
DSC2	0.211	0.498	1.42	UP
RSC1A1	0.45	0.896	2.731	UP
PICALM	0.227	0.444	1.325	UP
RAB5A	0.175	0.372	1.139	UP
KIAA0895	0.37	0.758	2.37	UP
ZBTB6	0.322	0.587	1.636	UP
LATS1	0.356	0.647	1.835	UP
IP6K2	0.405	0.774	2.19	UP
ICAM1	0.273	0.497	1.391	UP
HBEGF	0.861	1.584	4.445	UP
FAM126B	0.484	0.949	2.593	UP
DYSFIP1	0.195	0.397	1.101	UP
CRAMP1L	0.211	0.43	1.187	UP
IPPK	0.296	0.729	1.886	UP
C10orf12	0.321	0.77	1.973	UP
RNF24	0.221	0.538	1.386	UP
NPC1L1	0.195	0.501	1.291	UP
EIF2C2	0.233	0.532	1.433	UP
SLC25A25	0.278	0.656	1.616	UP
LRRC57	0.236	0.54	1.322	UP
FXR2	0.295	0.677	1.68	UP
LOC645978	0.681	1.463	3.058	UP
CUGBP1	0.273	0.551	1.165	UP
ARL8A	0.244	0.512	1.09	UP
ZSWIM4	0.465	0.932	2.014	UP
YRDC	0.628	1.204	2.674	UP
TUFT1	0.537	1.072	2.388	UP
GRM4	0.233	0.519	1.122	UP
C1orf128	0.374	0.882	1.908	UP
C3orf52	1.048	2.263	4.964	UP
MSL2	0.286	0.623	1.464	UP
MIER3	0.342	0.711	1.718	UP
HIST1H2AE	0.248	0.512	1.188	UP
ELOVL7	0.356	0.748	1.708	UP
PDE12	0.404	0.855	1.994	UP
GATA3	0.346	0.694	1.622	UP
EFNB2	0.537	1.055	2.46	UP
PPM1D	0.559	1.105	2.623	UP
ZBTB10	0.425	0.779	1.806	UP
PHLPP2	0.368	0.662	1.536	UP
NFKB1	0.414	0.723	1.717	UP
LIN54	0.359	0.636	1.484	UP
ZNF669	0.472	0.863	1.988	UP
ITFG2	0.382	0.669	1.512	UP
COG3	0.376	0.69	1.544	UP
RNF139	0.307	0.518	1.199	UP
NLGN3	0.271	0.473	1.182	UP

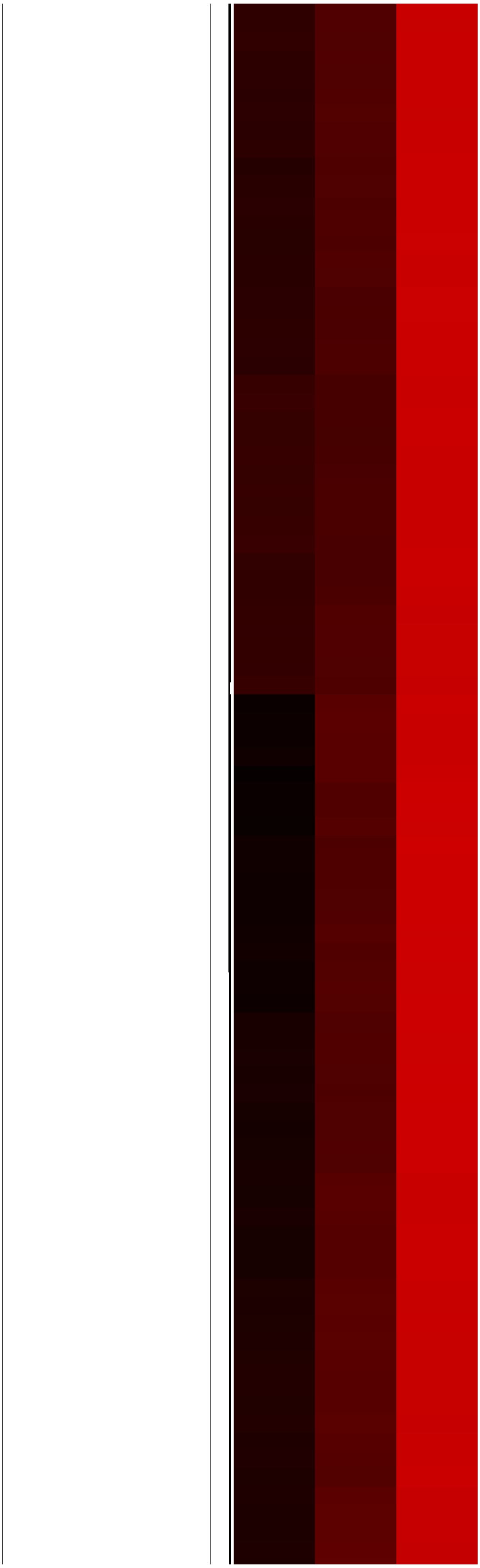

KIAA0415	0.468	0.791	1.96	UP
VNN2	0.508	0.828	2.099	UP
ZNF761	0.352	0.621	1.541	UP
NFYA	0.329	0.593	1.499	UP
SLC7A6	0.504	0.934	2.309	UP
LOC100131257	0.239	0.443	1.067	UP
IER5	0.564	1.041	2.598	UP
ARL5B	0.875	1.544	3.839	UP
C16orf11	0.225	0.467	1.205	UP
LSM14A	0.237	0.457	1.167	UP
HIVEP1	0.489	0.897	2.337	UP
RNF219	0.247	0.468	1.211	UP
CPSF7	0.214	0.41	1.087	UP
CREB5	0.446	0.872	2.154	UP
C10orf28	0.372	0.729	1.841	UP
GTF2B	0.32	0.547	1.481	UP
C1orf26	0.359	0.625	1.692	UP
TJAP1	0.24	0.393	1.073	UP
TRIM11	0.302	0.515	1.368	UP
POFUT2	0.559	1.002	2.654	UP
SSBP2	0.434	0.565	1.61	UP
KIN	0.294	0.369	1.043	UP
HBP1	0.443	0.595	1.7	UP
GADD45G	0.648	0.848	2.475	UP
DDX47	0.374	0.491	1.393	UP
CCL20	0.914	1.243	3.452	UP
UBE2G1	0.293	0.4	1.065	UP
C9orf80	0.38	0.535	1.425	UP
WDR47	0.589	0.8	2.125	UP
CKS2	0.415	0.538	1.465	UP
REL	0.691	1.033	2.846	UP
LINS1	0.525	0.792	2.179	UP
HIST2H2BE	0.42	0.647	1.736	UP
KLHL15	0.747	1.185	2.871	UP
GLYAT	0.32	0.505	1.257	UP
GADD45B	1.235	1.951	4.82	UP
PVT1	0.425	0.663	1.67	UP
C18orf25	0.301	0.433	1.091	UP
RANGAP1	0.079	0.644	1.481	UP
GABPB1	0.145	1.032	2.272	UP
CRCP	0.129	0.846	1.931	UP
BEND7	0.105	0.531	1.202	UP
ICOSLG	0.051	0.629	1.43	UP
UHRF2	0.055	0.442	1.112	UP
TRMT61A	0.102	0.717	1.803	UP
GLTP	0.082	0.725	1.745	UP
MAMLD1	0.108	0.502	1.338	UP
MAFG	0.087	0.416	1.096	UP
ZNF791	0.09	0.463	1.218	UP
TNKS2	0.08	0.45	1.158	UP
SAMD8	0.242	1.245	3.17	UP
FLJ31104	0.09	0.434	1.05	UP
C16orf80	0.114	0.495	1.243	UP
MGC29506	0.077	0.412	1.002	UP
ARF4	0.088	0.514	1.231	UP
SMN2	0.085	0.534	1.306	UP
ARID3B	0.363	1.194	3.051	UP
AIM1L	0.241	0.758	1.896	UP
VPS37B	0.298	0.92	2.309	UP
TIMP3	0.199	0.606	1.569	UP
CDR2	0.154	0.456	1.201	UP
NFKBIE	0.402	1.402	3.603	UP
KIRREL	0.217	0.811	2.082	UP
EDC3	0.132	0.472	1.193	UP
ATXN2	0.138	0.454	1.163	UP
EIF3J	0.166	0.58	1.345	UP
ARL5C	0.295	1.128	2.577	UP
PCID2	0.176	0.576	1.32	UP
PDRG1	0.303	1.113	2.66	UP
CXCR4	0.214	0.782	1.882	UP
C7orf20	0.221	0.817	1.924	UP
MDM4	0.209	0.628	1.424	UP
FRRS1	0.184	0.58	1.288	UP
SPATA2L	0.251	0.744	1.678	UP
PWWP2A	0.17	0.485	1.083	UP
PHF8	0.214	0.57	1.279	UP
MUL1	0.176	0.45	1.028	UP
ATF3	1.021	2.492	5.696	UP
ZNF699	0.276	0.717	1.628	UP
SMURF2	0.379	0.947	2.114	UP
HIC2	0.313	0.893	2.082	UP
DNAL1	0.179	0.459	1.096	UP
EGR1	1.445	4.102	10.025	UP
EPC1	0.207	0.62	1.342	UP
AKAP12	0.342	1.101	2.358	UP
TIPARP	0.292	0.943	2.01	UP
STX6	0.218	0.687	1.414	UP
GADD45A	0.594	1.992	4.031	UP

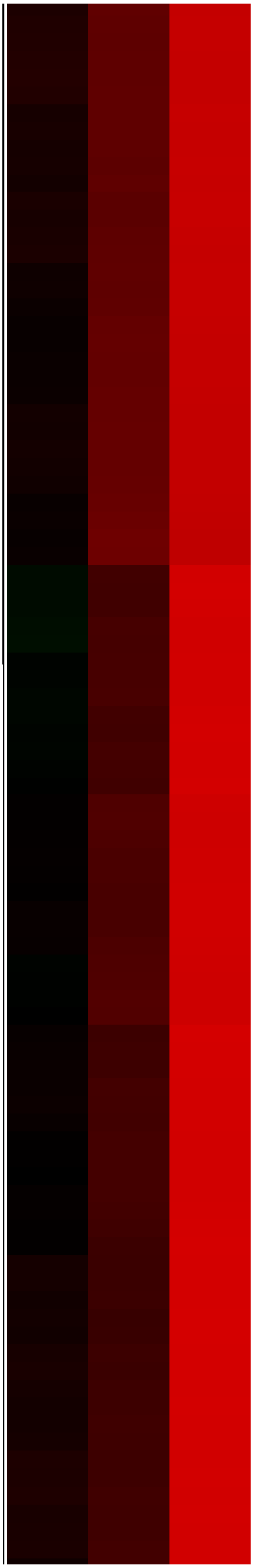

FBXL12	0.411	1.235	2.589	UP
ACVR2A	0.405	1.179	2.492	UP
FOSB	1.36	3.751	7.85	UP
EAF1	0.351	0.945	1.958	UP
DEFB112	0.181	0.51	1.053	UP
LOC100131154	0.165	0.698	1.455	UP
DUSP21	0.151	0.601	1.239	UP
MED1	0.139	0.593	1.251	UP
LOC100128893	0.172	0.695	1.49	UP
RUNX2	0.178	0.865	1.797	UP
STARD13	0.342	1.177	2.585	UP
SMTN	0.156	0.61	1.349	UP
HIST1H1C	0.148	0.544	1.157	UP
CYR61	0.535	1.864	3.892	UP
ZFAND3	0.091	0.583	1.21	UP
LOC400043	0.082	0.503	1.059	UP
TMEM39A	0.081	0.65	1.343	UP
SP2	0.071	0.801	1.591	UP
FOXD1	0.117	1.459	2.843	UP
LOC727962	0.055	0.526	1.037	UP
FLJ41170	0.098	0.903	1.815	UP
PTPN14	0.075	0.677	1.325	UP
CDCA4	0.207	1.071	2.083	UP
BTG1	0.215	1.193	2.3	UP
CSNK1D	0.149	0.746	1.462	UP
CHD2	0.251	1.345	2.62	UP
RAPGEF1	0.087	0.547	1.059	UP
GMEB2	0.086	1.005	1.893	UP
CHMP4C	0.159	1.585	2.876	UP
NFYC	0.043	0.611	1.059	UP
LOC100128591	0.057	0.674	1.177	UP
HIF1AN	-0.066	0.395	1.268	
ATF7IP	-0.093	0.539	1.775	
TIAL1	-0.06	0.331	1.075	
RCL1	-0.1	0.523	1.54	
MERTK	-0.089	0.421	1.26	
LRRC8E	-0.038	0.714	2.12	
LIMA1	-0.037	0.606	1.774	
PIK3CD	-0.062	0.728	2.107	
HIST1H2AA	-0.058	0.585	1.871	
EP300	-0.024	0.316	1.031	
POLR2D	-0.029	0.391	1.205	
MTHFR	-0.02	0.389	1.223	
SLC25A26	-0.007	0.386	1.265	
RAB21	0.02	0.4	1.039	UP
ADM	0.053	1.268	3.257	UP
NANP	0.029	0.513	1.369	UP
GOLGA7B	0.043	0.635	1.773	UP
BTG2	0.065	1.199	3.331	UP
HINFP	0.01	0.348	1.004	UP
RP9P	0.051	0.451	1.291	UP
ANXA5	0.056	0.447	1.287	UP
LHFP	0.068	0.726	1.969	UP
LY6G6F	-0.017	0.404	1.063	
LOC644100	-0.017	0.626	1.611	
GDF6	-0.038	1.251	3.127	
LOC401490	-0.001	0.496	1.254	UP
LIPT1	0.048	0.375	1.306	UP
KCTD11	0.116	0.79	2.67	UP
CORO1C	0.071	0.406	1.395	UP
UAP1L1	0.09	0.585	1.832	UP
IGHMBP2	0.107	0.566	1.781	UP
MAK	0.044	0.334	1.064	UP
NUP35	0.016	0.399	1.218	UP
MAP3K1	0.013	0.421	1.289	UP
RAB30	-0.001	1.018	3.128	
SQSTM1	0.036	0.444	1.366	UP
RBM22	0.044	0.558	1.754	UP
RNF185	0.032	0.638	2.129	UP
LOC100129380	0.029	0.477	1.699	UP
TP53BP2	0.171	0.477	1.725	UP
ELL	0.261	0.731	2.627	UP
RUNX1	0.24	0.799	2.835	UP
RNF121	0.109	0.316	1.203	UP
PRKRIP1	0.126	0.411	1.558	UP
RELT	0.288	0.735	2.579	UP
CXorf15	0.147	0.33	1.199	UP
MBD5	0.151	0.428	1.455	UP
LOC100128528	0.097	0.303	1.043	UP
KLF10	0.268	0.876	2.891	UP
GFRA2	0.145	0.434	1.453	UP
ORC6L	0.195	0.405	1.382	UP
LOC642278	0.156	0.337	1.154	UP
BTAF1	0.164	0.349	1.138	UP
KRTAP6-3	0.152	0.386	1.28	UP
HABP4	0.126	0.319	1.053	UP
SIAH1	0.201	0.504	1.593	UP
DDA1	0.084	0.368	1.157	UP

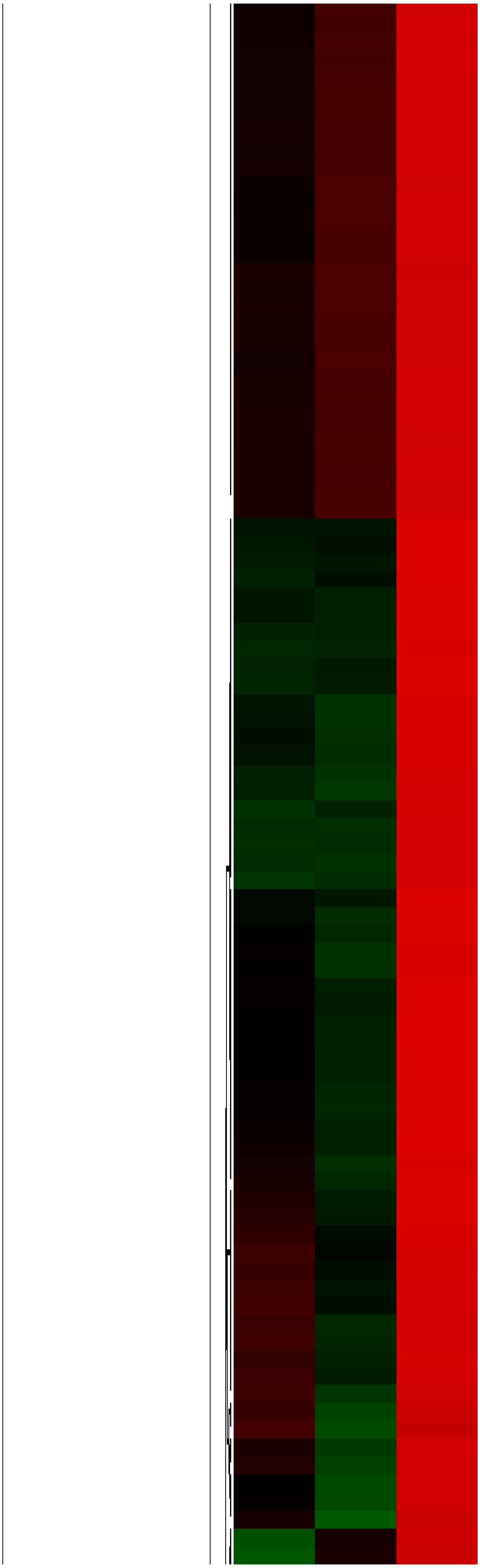

CHRNA10	0.133	0.606	1.956	UP
ETS1	0.242	1.083	3.347	UP
LOC100129112	0.132	0.501	1.628	UP
SLC26A8	0.131	0.489	1.507	UP
ALKBH1	0.151	0.561	1.687	UP
SNRK	0.127	0.452	1.349	UP
C5orf40	0.235	0.741	2.324	UP
C14orf118	0.128	0.435	1.375	UP
MAP3K7IP3	0.139	0.454	1.43	UP
SOCS7	0.067	0.366	1.038	UP
RMND5A	0.09	0.568	1.611	UP
MAML2	0.078	0.457	1.307	UP
HELB	0.117	0.592	1.761	UP
C15orf57	0.113	0.684	2.007	UP
SETD4	0.204	0.546	1.495	UP
PLGLB1	0.118	0.372	1.054	UP
ABL2	0.283	0.867	2.383	UP
SLC12A4	0.149	0.477	1.378	UP
LOC100128130	0.152	0.484	1.397	UP
AKIRIN1	0.133	0.503	1.377	UP
ZCCHC3	0.186	0.539	1.635	UP
TNFAIP3	0.495	1.457	4.397	UP
E2F3	0.213	0.553	1.695	UP
CCNT2	0.337	0.876	2.594	UP
CRK	0.158	0.43	1.294	UP
C20orf4	0.167	0.471	1.438	UP
PISD	0.242	0.634	1.833	UP
LEMD3	0.141	0.392	1.147	UP
MAP3K9	-0.215	-0.212	2.578	
C9orf50	-0.13	-0.087	1.188	
NXF1	-0.177	-0.138	1.422	
KLHL26	-0.196	-0.094	1.394	
MCTP2	-0.16	-0.231	1.608	
EIF2AK3	-0.119	-0.176	1.191	
ZNF26	-0.192	-0.177	1.234	
LILRB3	-0.208	-0.189	1.19	
ZNF473	-0.217	-0.148	1.359	
FOXA3	-0.381	-0.26	2.245	
DNAJB4	-0.139	-0.303	1.325	
LOC645277	-0.092	-0.271	1.154	
TSC22D1	-0.076	-0.232	1.078	
DHRS2	-0.119	-0.309	1.477	
SPRYD5	-0.212	-0.348	1.46	
CRB1	-0.166	-0.308	1.18	
RP11-470P4.2	-0.259	-0.169	1.105	
FHL3	-0.302	-0.324	1.484	
ARVCF	-0.293	-0.27	1.336	
LOC645225	-0.278	-0.331	1.381	
DGCR11	-0.282	-0.23	1.142	
RORA	-0.053	-0.141	1.356	
ITGB1BP2	-0.077	-0.409	1.989	
C17orf99	-0.041	-0.618	3.356	
APOA5	0.042	-0.442	1.887	
ADAM20	0.018	-0.404	1.792	
SHC2	0.04	-0.216	1.622	
AFF2	0.037	-0.225	1.764	UP
ZNF780A	-0.01	-0.164	1.122	
TSPYL2	-0.019	-0.352	2.332	
EDN1	0.02	-0.201	1.303	
CXorf1	0.024	-0.308	2.033	
ZNF286A	0.028	-0.18	1.094	
AHSP	0.022	-0.189	1.105	
LDLR	0.053	-0.139	1.02	
CDO1	0.08	-0.157	1.129	
LOC283483	0.132	-0.31	1.426	
BCLAF1	0.122	-0.202	1.132	
RGS6	0.26	-0.237	1.771	
C15orf55	0.184	-0.114	1.022	
CC2D2B	0.223	-0.044	1.032	
THOC6	0.326	-0.032	1.158	
ZFAND5	0.286	-0.077	1.125	
RBM39	0.506	-0.157	1.806	
ARRDC3	0.365	-0.09	1.2	
PLEKHG1	0.28	-0.199	1.025	
NR4A1	0.673	-0.378	2.252	
LOC285626	0.334	-0.202	1.431	
ASGR1	0.303	-0.15	1.12	
GABRA6	0.421	-0.371	1.47	
LOC728152	0.297	-0.363	1.092	
LOC100133264	0.41	-0.462	1.203	
PELI1	0.125	-0.283	1.027	
LOC731852	0.171	-0.342	1.084	
CREBZF	-0.013	-0.459	1.308	
ATF7IP2	0.029	-0.391	1.121	
GDF9	0.183	-0.795	1.79	
LOC255025	-0.541	0.148	1.411	
LOC100130141	-0.634	0.201	1.48	
ZNF462	-0.57	0.169	1.146	

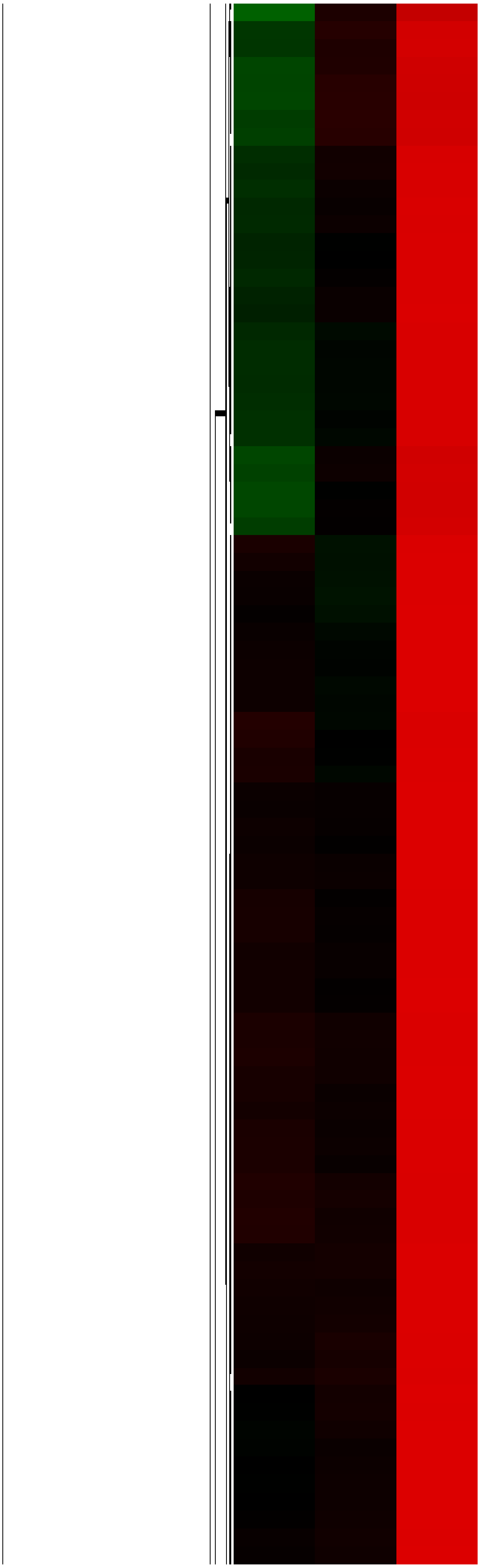

GRHL1	-0.328	0.223	1.258	
FUBP1	-0.349	0.197	1.373	
PLCL2	-0.568	0.258	1.682	
NMT2	-0.612	0.356	1.846	
EGR4	-0.975	0.568	2.882	
RRAGC	-0.377	0.257	1.303	
HIST4H4	-0.671	0.418	2.191	
PRB4	-0.23	0.09	1.083	
EIF1AD	-0.257	0.115	1.322	
LOC100292680	-0.389	0.098	1.819	
ZFYVE1	-0.266	0.055	1.438	
HIST3H2BB	-0.444	0.13	2.34	
KIAA1875	-0.271	-0.01	1.669	
DIDO1	-0.17	0.002	1.002	
LOC100134423	-0.338	0.035	1.817	
ZNF155	-0.211	0.066	1.341	
NWD1	-0.269	0.089	1.842	
LOC219690	-0.296	-0.076	1.588	
DCTN5	-0.289	-0.038	1.415	
CNKSR1	-0.33	-0.051	1.612	
ZNF8	-0.223	-0.034	1.105	
KGFLP1	-0.234	-0.038	1.119	
GUCY2D	-0.408	-0.032	1.794	
CDC25A	-0.304	-0.048	1.342	
STXBP6	-0.573	0.092	1.692	
KCNJ9	-0.352	0.072	1.138	
PPRC1	-0.38	-0.005	1.116	
HIST1H4J	-0.448	0.027	1.334	
ZNF16	-0.407	0.024	1.392	
MLL5	0.16	-0.102	1.31	
LAG3	0.145	-0.127	1.684	UP
ZKSCAN5	0.053	-0.091	1.113	UP
IRF1	0.101	-0.203	2.29	UP
NUFIP1	0.027	-0.103	1.408	UP
CCDC49	0.058	-0.05	1.369	UP
NUP153	0.075	-0.023	1.271	UP
ARIH1	0.07	-0.02	1.13	UP
KRT27	0.091	-0.055	1.469	UP
GNLY	0.153	-0.075	2.458	UP
NPAT	0.168	-0.036	1.023	
LOC100132855	0.402	0.012	2.669	UP
ZNF317	0.181	-0.007	1.546	UP
LOC100132653	0.173	-0.048	1.434	UP
IPMK	0.06	0.038	1.131	UP
ALS2	0.067	0.049	1.495	UP
TNF	0.077	0.038	1.23	UP
LOC541471	0.078	0.016	1.503	UP
PNN	0.096	0.071	1.506	UP
PLCB4	0.075	0.062	1.153	UP
ZNF764	0.172	0.024	1.707	UP
XAB2	0.12	0.035	1.104	UP
KLHDC10	0.111	0.024	1.019	UP
LOC728528	0.122	0.059	1.527	UP
INO80	0.098	0.043	1.155	UP
RRN3	0.13	0.026	1.55	UP
FOXR1	0.135	0.037	1.622	UP
C7orf43	0.231	0.141	1.855	UP
ATP6V1D	0.155	0.1	1.29	UP
CCKBR	0.314	0.178	2.418	UP
WAPAL	0.146	0.101	1.378	UP
USP43	0.212	0.096	1.986	UP
C10orf62	0.148	0.091	1.64	UP
TOR1AIP2	0.177	0.073	1.462	UP
C1orf52	0.154	0.073	1.243	UP
IL6	0.188	0.059	1.499	UP
ZFP36	0.34	0.208	2.307	UP
F3	0.312	0.225	2.233	UP
LOC90586	0.187	0.091	1.231	UP
C6orf94	0.168	0.091	1.111	UP
SF3B4	0.13	0.157	1.712	UP
LOC346329	0.121	0.123	1.333	UP
PASD1	0.08	0.072	1.002	UP
CLEC4A	0.127	0.141	1.819	UP
YY1AP1	0.07	0.098	1.086	UP
TAS2R10	0.105	0.193	1.677	UP
PLAC4	0.064	0.124	1.217	UP
CCRN4L	0.284	0.423	3.436	UP
FOSL2	-0.007	0.152	1.752	
ARAP2	-0.019	0.199	2.153	
SOCS1	-0.052	0.22	2.853	
C7orf64	-0.017	0.058	1.215	
UBXN7	-0.003	0.089	1.547	
HUS1	-0.006	0.073	1.166	
FLCN	0.002	0.067	1.104	UP
LOC643837	0.022	0.145	2.125	UP
KLHL36	0.061	0.121	1.536	UP
BAGE4	0.038	0.095	1.393	UP
BMP2K	-0.072	0.082	1.281	

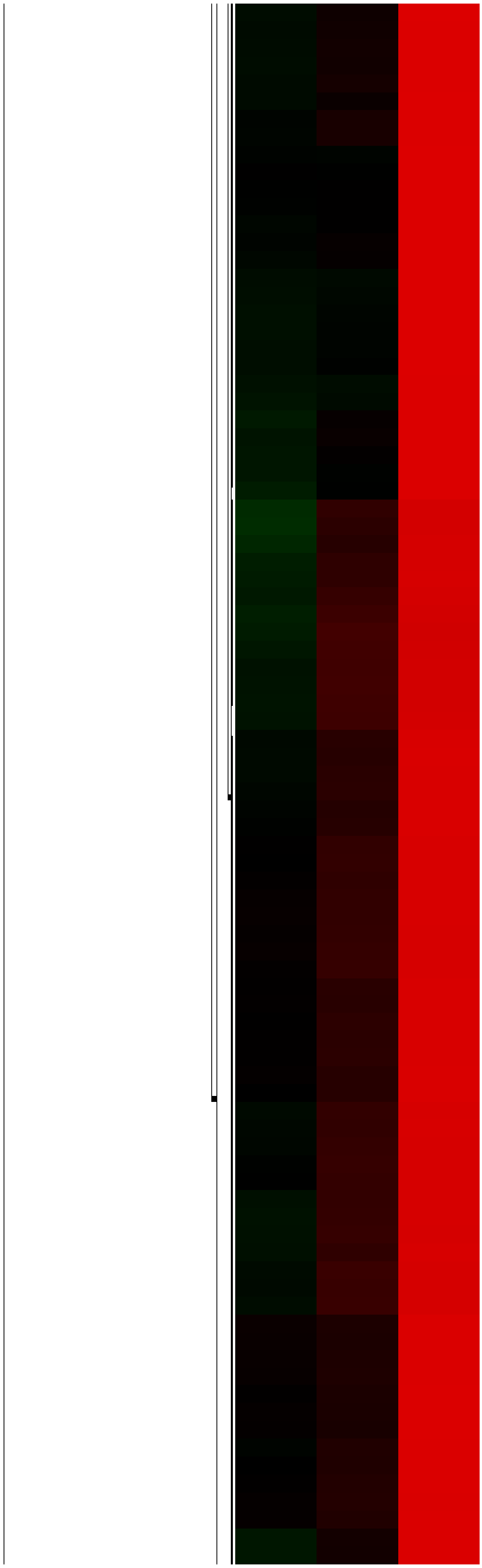

LOC100130419	-0.055	0.089	1.167	
GLYR1	-0.061	0.095	1.147	
DACT1	-0.089	0.129	1.619	
RNU4ATAC	-0.113	0.232	2.342	
IL4R	-0.073	0.071	1.513	
RASAL2	-0.039	0.212	1.889	
KIAA1267	-0.034	0.151	1.331	
MS4A6E	-0.044	-0.061	2.878	
ID3	0.008	-0.006	1.035	UP
CYTH2	-0.007	0.006	1.07	
CXorf21	-0.017	0.011	1.314	
LOC100127937	-0.04	0.008	1.314	
LOC729739	-0.034	0.051	1.622	
C10orf111	-0.046	0.038	1.45	
ZNF595	-0.065	-0.052	1.164	
PTGER4	-0.187	-0.099	3.085	
MMP25	-0.196	-0.068	2.722	
LOC646626	-0.152	-0.049	2.182	
NUDT17	-0.125	-0.04	2	
DUSP16	-0.064	-0.014	1.057	
GNL1	-0.083	-0.061	1.098	
CRTAM	-0.118	-0.064	1.339	
TGIF2	-0.148	0.04	1.265	
CD83	-0.422	0.201	4.731	
LOC648556	-0.116	0.018	1.174	
CYSLTR1	-0.102	-0.01	1.044	
ARID5B	-0.169	-0.009	1.318	
OR52K1	-0.469	0.547	2.357	
C17orf85	-0.258	0.255	1.232	
FAM193A	-0.314	0.314	1.733	
KIAA0174	-0.205	0.32	1.468	
BCL2L13	-0.157	0.265	1.221	
ZSCAN22	-0.163	0.361	1.438	
SP4	-0.178	0.346	1.232	
KLC3	-0.334	0.811	2.539	
SERPINB5	-0.16	0.46	1.497	
PHYHD1	-0.085	0.316	1.043	
BEST3	-0.098	0.338	1.096	
PLAGL2	-0.096	0.299	1.018	
NECAP2	-0.09	0.3	1.04	
C3orf59	-0.103	0.459	2.491	
BCAR1	-0.048	0.191	1.083	
TRANK1	-0.068	0.309	1.61	
C3orf38	-0.042	0.267	1.373	
MOBP	-0.021	0.187	1.115	
AKIRIN2	-0.013	0.197	1.105	
ZSWIM6	0.014	0.371	1.615	UP
C5orf49	0.005	0.268	1.154	UP
TEPP	0.028	0.36	1.627	UP
ZNF175	0.036	0.293	1.273	UP
SPATA2	0.061	0.431	1.86	UP
KISS1	0.054	0.457	1.95	UP
hCG_1795283	0.072	0.515	2.097	UP
TP53RK	0.027	0.428	1.73	UP
THBS1	0.014	0.282	1.467	UP
LOC286059	0.017	0.201	1.065	UP
KLF6	0.015	0.503	2.471	UP
C20orf111	0.015	0.285	1.46	UP
OR5C1	0.014	0.231	1.144	UP
ZNF256	0.048	0.445	2.506	UP
WDR26	0.001	0.208	1.181	UP
FBXO28	-0.05	0.277	1.179	
FAM71F2	-0.035	0.242	1.073	
GPN2	-0.054	0.439	1.847	
TCF20	-0.012	0.265	1.05	
NEXN	-0.018	0.527	2.218	
YTHDF1	-0.116	0.391	1.645	
SART3	-0.099	0.289	1.208	
ANKLE2	-0.131	0.415	1.67	
CLTB	-0.089	0.281	1.269	
DAPK3	-0.091	0.486	1.812	
AMICA1	-0.051	0.287	1.1	
QTRTD1	-0.066	0.298	1.13	
C1orf63	0.113	0.313	2.404	UP
BRD1	0.046	0.132	1.044	UP
ZNFX1	0.062	0.218	1.608	UP
ZNF79	0.039	0.149	1.079	UP
KDM5B	0.017	0.175	1.402	UP
TRAF1	0.069	0.355	2.975	UP
EGR2	0.118	0.72	6.465	UP
USP42	-0.024	0.174	1.185	
NUAK2	0.006	0.446	3.1	UP
LOC100129767	0.012	0.173	1.08	UP
SNIP1	0.056	0.361	2.208	UP
C17orf86	0.029	0.186	1.247	UP
POU2F1	-0.148	0.127	1.442	
OR5H1	-0.112	0.091	1.068	
HMGXB3	-0.19	0.118	1.436	

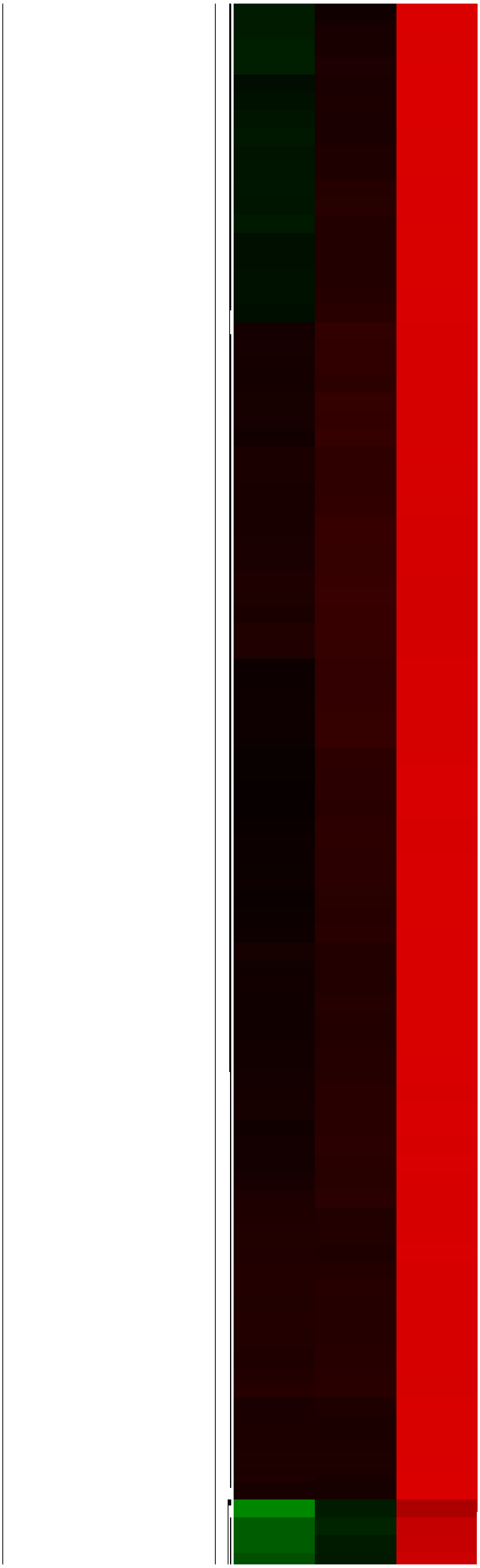

ZNF542	-0.276	0.253	2.164	
KIAA1543	-0.153	0.124	1.072	
PLEKHO2	-0.307	0.286	2.079	
ZBTB7B	-0.084	0.172	1.395	
MIDN	-0.101	0.162	1.255	
RAD18	-0.203	0.249	2.049	
PLEKHF2	-0.149	0.159	1.319	
ZNF530	-0.139	0.208	1.471	
PNO1	-0.1	0.144	1.021	
MAEA	-0.123	0.202	1.173	
LOC100129390	-0.148	0.27	1.485	
SUV420H1	-0.172	0.231	1.398	
ZNF35	-0.132	0.304	1.908	
ZNF134	-0.172	0.384	2.445	
KATNA1	-0.112	0.223	1.399	
ZNF263	-0.124	0.276	1.583	
NEURL3	-0.224	0.646	3.45	
WDR43	0.108	0.256	1.116	UP
KIAA0226	0.126	0.268	1.208	UP
ELK1	0.183	0.422	1.859	UP
EYA3	0.103	0.217	1.032	UP
DDI2	0.263	0.616	2.49	UP
CBLL1	0.135	0.303	1.258	UP
GPRC5B	0.206	0.579	2.378	UP
MLL4	0.146	0.245	1.128	UP
AFTPH	0.157	0.264	1.203	UP
ZMYM5	0.144	0.276	1.234	UP
SPRED3	0.218	0.416	1.827	UP
ZNF223	0.15	0.33	1.274	UP
SERTAD1	0.447	0.929	3.57	UP
OR7E5P	0.189	0.38	1.516	UP
SNW1	0.167	0.297	1.156	UP
LOC100132469	0.336	0.629	2.376	UP
LOC100130828	0.254	0.504	1.927	UP
ZNF193	0.166	0.275	1.061	UP
ANKRD33B	0.358	0.582	2.348	UP
GPR84	0.062	0.249	1.058	UP
HMGCS1	0.151	0.543	2.353	UP
FAM53C	0.121	0.383	1.629	UP
SULT1B1	0.083	0.29	1.164	UP
CYP1A1	0.233	0.952	3.794	UP
ZNF321	0.057	0.268	1.287	UP
DLX2	0.184	0.85	4.299	UP
CCDC64B	0.146	0.72	3.683	UP
C1orf230	0.051	0.264	1.368	UP
ZNF121	0.096	0.372	1.794	UP
CBX4	0.087	0.304	1.434	UP
KIAA0753	0.06	0.204	1.031	UP
ZBTB43	0.136	0.389	1.95	UP
NRF1	0.101	0.333	1.845	UP
AHR	0.071	0.217	1.227	UP
SLC1A3	0.091	0.261	1.407	UP
MAGEA1	0.115	0.181	1.113	UP
SYNJ1	0.164	0.326	2.023	UP
NAB2	0.236	0.43	2.789	UP
CPEB3	0.178	0.383	2.295	UP
C1orf55	0.173	0.353	2.183	UP
ZNF227	0.148	0.32	1.95	UP
ZNF586	0.169	0.342	1.982	UP
ATF6	0.098	0.186	1.06	UP
TAP2	0.119	0.229	1.193	UP
LY6G5B	0.13	0.236	1.27	UP
TBX21	0.243	0.57	2.953	UP
MED21	0.103	0.217	1.097	UP
MAGEA2B	0.114	0.239	1.265	UP
ZNF827	0.119	0.185	1.011	UP
GPR146	0.262	0.382	1.93	UP
OLR1	0.207	0.225	1.434	UP
DUSP8	0.21	0.219	1.361	UP
YTHDF3	0.206	0.196	1.35	UP
GTPBP4	0.181	0.181	1.081	UP
C17orf54	0.25	0.271	1.523	UP
ZNHIT6	0.216	0.246	1.403	UP
TRIO	0.235	0.255	1.452	UP
MECP2	0.161	0.183	1.021	UP
IKZF5	0.306	0.383	2.166	UP
ZBTB40	0.242	0.287	1.464	UP
LOC100128233	0.243	0.258	1.358	UP
IL28A	0.172	0.197	1.405	UP
IQCA1L	0.26	0.247	1.971	UP
FAM46B	0.255	0.258	1.951	UP
SERTAD3	0.211	0.213	1.542	UP
RLF	0.347	0.345	2.621	UP
GLIS3	0.192	0.161	1.342	UP
KDM6A	0.199	0.18	1.7	UP
KLF2	-0.969	-0.195	1.234	
PIK3IP1	-0.618	-0.236	1.311	
DEFB136	-0.619	-0.184	1.333	

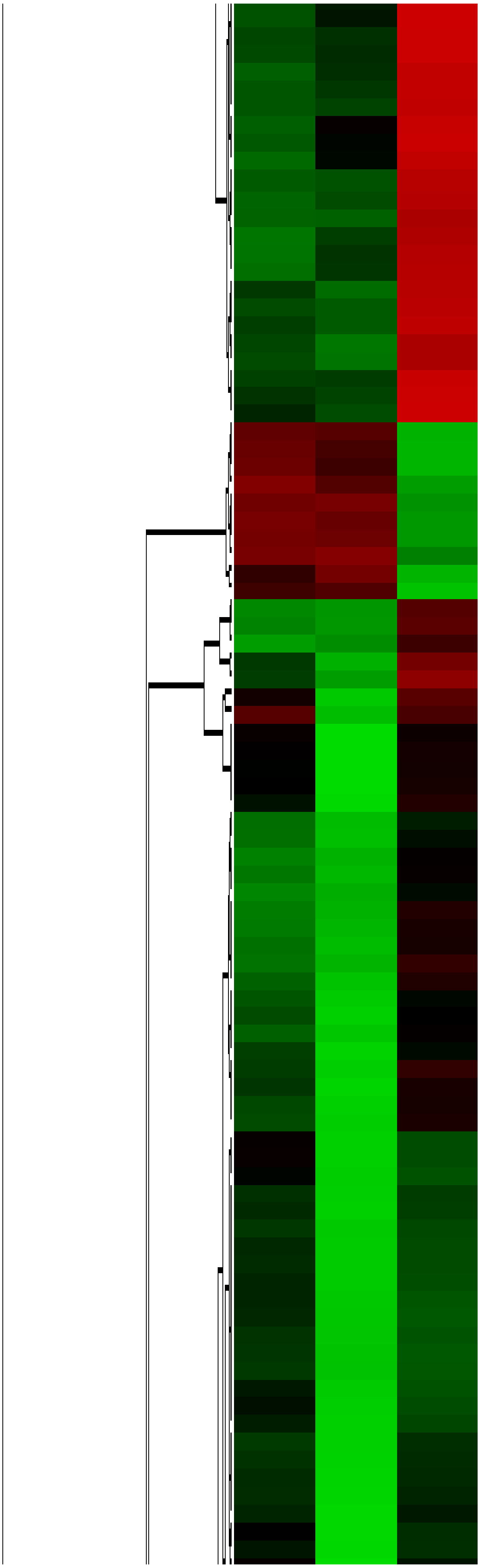

TEX10	-0.435	-0.139	1.104
COL29A1	-0.441	-0.111	1.109
MED26	-0.468	-0.316	1.352
FAM46C	-0.863	-0.508	2.377
GYPE	-0.618	-0.304	1.252
C17orf104	-0.465	-0.298	1.045
FASTKD5	-0.45	-0.36	1.017
WWP2	-0.577	0.046	1.209
HIST1H4F	-0.621	-0.035	1.413
HIST2H4B	-0.645	-0.048	1.181
RASD1	-0.814	-0.74	1.638
HIST2H3A	-0.635	-0.473	1.128
CENPL	-0.582	-0.575	1.006
ZNF670	-0.974	-0.516	1.444
RHOB	-0.99	-0.435	1.524
MAPK8IP2	-0.616	-0.293	1.007
IL17C	-0.338	-0.646	1.097
ZNF207	-0.505	-0.62	1.255
EMX2	-0.506	-0.73	1.553
TMEM233	-0.563	-0.979	1.396
PCDHGB3	-0.547	-0.843	1.237
ZNF222	-0.381	-0.352	1.172
GPATCH2	-0.258	-0.337	1.009
PLK2	-0.216	-0.457	1.221
C8orf44	0.689	0.621	-1.263
SMOC1	0.641	0.427	-1.119
LOC100129387	0.601	0.341	-1.003
TYSND1	1.045	0.67	-1.268
JDP2	0.895	0.966	-1.169
SH3PXD2A	0.842	0.718	-1.053
C18orf56	0.948	0.892	-1.241
C5orf53	0.903	1.002	-0.965
PSPH	0.327	0.797	-1.222
ASAM	0.496	0.629	-1.527
TRA2A	-1.316	-1.436	0.819
PLEKHA4	-1.225	-1.413	0.855
CGN	-1.026	-0.922	0.4
ZMAT1	-0.389	-1.199	0.796
CYP8B1	-0.444	-1.134	1.025
LOC644246	0.117	-1.239	0.545
HES1	0.498	-1.076	0.413
IBSP	0.041	-1.013	0.059
PCSK9	0.023	-2.209	0.202
DKK1	-0.012	-1.004	0.089
CFHR4	-0.002	-1.141	0.117
EZH2	-0.086	-1.037	0.168
EVI2B	-0.62	-1.057	-0.164
ELP2P	-0.69	-1.182	-0.091
TCTA	-1.298	-1.784	0.044
hCG_1820661	-0.745	-1.144	0.041
TAOK2	-1.006	-1.306	-0.077
RINL	-1.308	-1.862	0.358
PIK3R3	-0.966	-1.438	0.195
SFRS2	-0.827	-1.371	0.163
TP53BP1	-0.717	-1.12	0.316
HCST	-0.546	-1.088	0.184
GFER	-0.587	-1.4	-0.046
FKBP5	-0.542	-1.48	0.009
C1orf211	-0.69	-1.414	0.029
VEZT	-0.31	-1.032	-0.045
C6orf41	-0.313	-1.093	0.26
SRRM1	-0.261	-1.065	0.128
LOC644422	-0.383	-1.093	0.124
FLJ36848	-0.443	-1.216	0.162
PDE11A	0.082	-2.382	-0.881
CD82	0.051	-1.321	-0.485
NINJ2	-0.027	-1.115	-0.444
RASL11A	-0.309	-1.306	-0.386
GINS1	-0.254	-1.258	-0.379
FBLN5	-0.28	-1.039	-0.372
GDA	-0.262	-1.321	-0.488
DAPK2	-0.252	-1.212	-0.453
ABHD1	-0.441	-2.429	-0.931
STK11	-0.209	-1.147	-0.488
hCG_2022304	-0.223	-1.108	-0.491
PTPLAD2	-0.32	-1.221	-0.515
GDF11	-0.466	-1.724	-0.779
DHCR24	-1.151	-3.876	-1.754
LOC729968	-0.251	-2.128	-0.849
ARHGDIB	-0.135	-1.853	-0.691
IVNS1ABP	-0.182	-1.264	-0.427
JMJD7	-0.381	-1.362	-0.305
PRPH	-0.247	-1.034	-0.211
LOC400684	-0.271	-1.366	-0.264
CANT1	-0.242	-1.157	-0.192
CALCR	-0.228	-1.389	-0.15
KRT23	-0.014	-2.047	-0.428
FDXACB1	-0.131	-1.32	-0.283

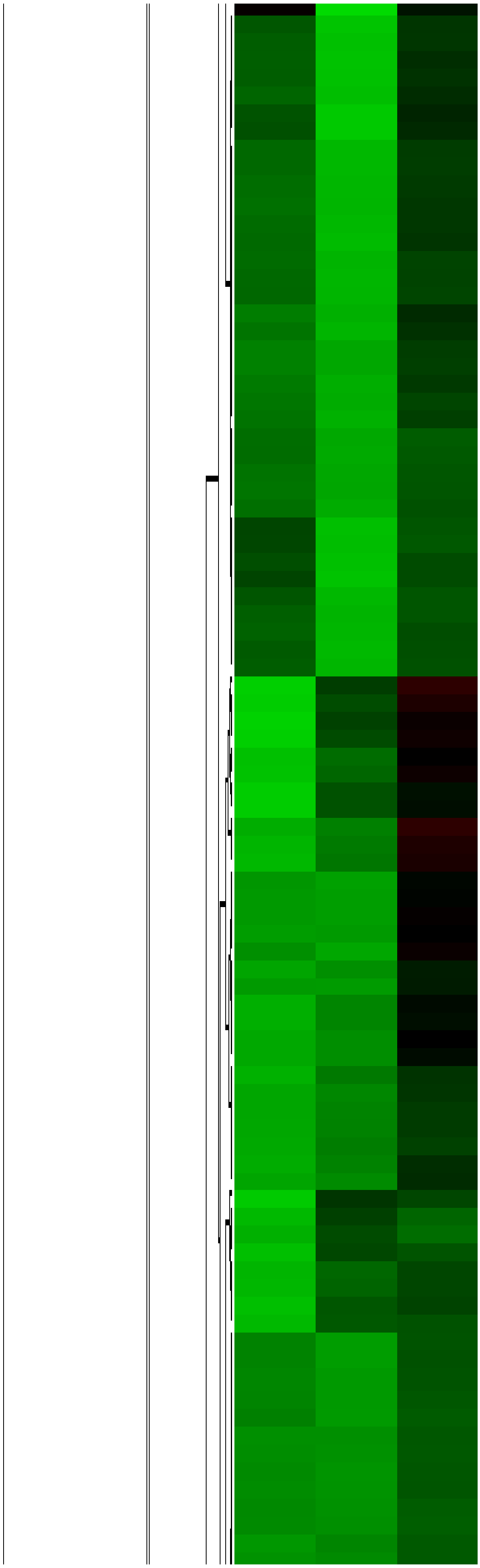

LOC100131000	0.031	-1.037	-0.094
C12orf34	-0.561	-1.28	-0.346
WTAP	-0.531	-1.088	-0.311
LOC100128437	-0.515	-1.054	-0.249
FAM47B	-0.547	-1.134	-0.293
HIC1	-0.96	-1.798	-0.415
NTF4	-0.559	-1.341	-0.242
MMRN1	-0.512	-1.288	-0.266
PGBD4	-0.758	-1.334	-0.434
C6orf52	-0.649	-1.144	-0.384
NRGN	-0.851	-1.409	-0.455
GSG2	-0.695	-1.125	-0.346
SUSD5	-0.722	-1.241	-0.372
KATNAL2	-0.818	-1.455	-0.408
ETNK2	-0.674	-1.127	-0.429
SLC45A4	-1.336	-2.333	-0.861
GRID2IP	-0.92	-1.609	-0.619
THY1	-0.829	-1.161	-0.283
MBLAC1	-0.804	-1.252	-0.34
HEJ1	-0.832	-1.077	-0.394
DNAJA1	-0.886	-1.142	-0.433
GBA	-0.745	-1.057	-0.35
LOC440181	-1.342	-1.952	-0.782
FKBP15	-0.731	-1.127	-0.406
RAB36	-0.804	-1.24	-0.679
C8orf38	-0.652	-1.025	-0.538
SH2D3C	-0.898	-1.299	-0.674
AFF3	-0.842	-1.187	-0.607
TMEM114	-0.743	-1.145	-0.543
OAZ2	-0.516	-1.424	-0.634
CDKN2C	-0.62	-1.677	-0.784
LOC729013	-0.653	-1.588	-0.623
HNRPDL	-0.595	-1.697	-0.653
LOC100133408	-0.565	-1.226	-0.568
EXT1	-0.54	-1.022	-0.484
ARMS2	-0.795	-1.471	-0.623
SFRS6	-0.777	-1.585	-0.679
C6orf62	-0.851	-1.67	-0.741
FUS	-1.062	-0.317	0.233
C17orf51	-1.23	-0.46	0.18
LOC145694	-1.09	-0.337	0.056
ADD1	-1.222	-0.443	0.092
TMEM182	-1.124	-0.632	0.011
LOC100129675	-1.023	-0.539	0.071
PCBP3OT	-1.018	-0.404	-0.083
BCL9	-1.331	-0.539	-0.087
SHROOM2	-1.532	-1.134	0.399
ZNF316	-2.083	-1.4	0.354
SNAR-A3	-1.312	-0.843	0.185
ZNF187	-0.963	-1.036	-0.04
LOC100134102	-1.222	-1.262	-0.036
ZNF280A	-1.022	-1.056	0.038
GPRASP1	-1.127	-1.106	-0.001
SFRS7	-1.101	-1.286	0.079
HIST1H2AL	-1.848	-1.6	-0.321
GPR160	-1.335	-1.344	-0.236
HYMAI	-1.283	-0.973	-0.073
CNN2	-1.16	-0.88	-0.095
TSPAN10	-1.018	-0.857	0.001
ANKRD34B	-1.288	-1.086	-0.086
HIST1H2AI	-1.083	-0.74	-0.313
GMIP	-1.269	-1.03	-0.405
HDX	-1.035	-0.818	-0.369
ACTRT1	-1.08	-0.84	-0.382
RAD51	-1.077	-0.802	-0.418
LOC390760	-2.045	-1.548	-0.545
HHEX	-1.055	-0.887	-0.278
FLJ46020	-1.084	-0.286	-0.377
LOC729856	-1.037	-0.362	-0.57
CYFIP2	-1.024	-0.438	-0.638
RFTN1	-2.019	-0.756	-0.892
PLIN2	-1.074	-0.614	-0.417
APLNR	-1.372	-0.754	-0.523
OR56A1	-1.162	-0.525	-0.408
EPHB3	-1.621	-0.773	-0.719
SNHG7	-1.131	-1.365	-0.725
ANK2	-0.925	-1.11	-0.571
ZNF512	-1.264	-1.439	-0.786
C17orf96	-0.94	-1.084	-0.622
ARNT2	-0.835	-1.006	-0.594
LOC100130370	-1.428	-1.432	-0.869
GPR82	-1.715	-1.764	-1.073
SLC43A2	-2.064	-2.219	-1.284
FABP12	-0.966	-1.011	-0.585
RNU12	-1.567	-1.667	-1.06
MRAS	-1.272	-1.316	-0.867
MED12	-1.305	-1.149	-0.767
SYT8	-2.645	-2.499	-1.619

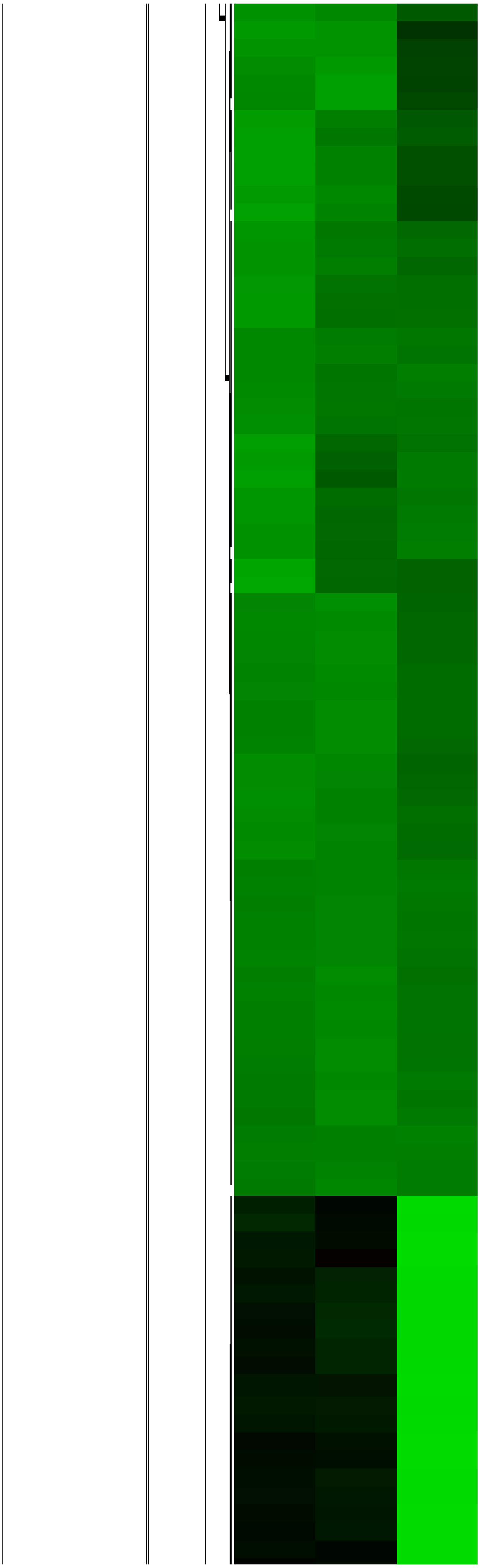

LOC729683	-1.36	-1.277	-0.844
ALPK3	-1.436	-1.387	-0.48
ABHD11	-1.223	-1.223	-0.532
LOC100134041	-1.173	-1.264	-0.565
ZNF434	-1.45	-1.682	-0.714
FLVCR2	-0.913	-1.077	-0.487
HIST1H1B	-1.227	-1.001	-0.696
HEYL	-2.409	-1.784	-1.39
SUPT16H	-1.057	-0.863	-0.536
RFC2	-1.061	-0.855	-0.53
RGAG4	-1.023	-0.905	-0.492
C22orf25	-1.061	-0.87	-0.482
TTTY17A	-1.627	-1.301	-1.153
SLC4A2	-1.272	-1.041	-0.945
LIMS2	-1.301	-1.105	-0.902
OTUB2	-1.007	-0.761	-0.741
MTG1	-1.264	-0.919	-0.91
LOC400655	-1.434	-1.047	-1.055
UNQ9374	-1.401	-1.272	-1.235
MANF	-1.291	-1.193	-1.094
LOC348761	-1.342	-1.142	-1.239
FAM117A	-1.135	-0.978	-1.003
LUZP2	-1.594	-1.343	-1.316
ACOT11	-1.074	-0.872	-0.895
APOA4	-1.168	-0.754	-0.848
LOC100128292	-1.037	-0.65	-0.817
FAM22B	-1.122	-0.638	-0.867
GJC3	-1.033	-0.746	-0.816
C22orf27	-1.523	-1.056	-1.24
PF4V1	-1.068	-0.772	-0.91
ANKRD2	-1.161	-0.818	-1.007
ZNF239	-1.183	-0.756	-0.707
HOMER2	-1.124	-0.679	-0.66
LOC727916	-0.939	-1.001	-0.701
TFCP2L1	-2.223	-2.256	-1.682
PRR19	-1.404	-1.452	-1.075
LOC729669	-1.247	-1.318	-0.975
LOC151121	-1.372	-1.425	-1.131
LOC100132015	-1.146	-1.16	-0.929
FBXO41	-1.209	-1.322	-1.017
FBXO25	-1.401	-1.537	-1.171
C17orf72	-1.774	-1.915	-1.434
LOC283480	-1.814	-1.731	-1.305
C11orf45	-1.113	-1.058	-0.814
ITIH1	-1.064	-0.968	-0.779
H1FNT	-1.083	-1.001	-0.862
SNORA20	-1.828	-1.756	-1.433
NCRNA00118	-1.167	-1.09	-0.888
NAP1L2	-1.243	-1.284	-1.17
LOC100130264	-1.584	-1.607	-1.482
GLI3	-1.628	-1.705	-1.537
C15orf34	-1.679	-1.747	-1.544
XYLB	-0.98	-1.014	-0.903
LILRA1	-1.371	-1.383	-1.196
ZNF777	-0.929	-1.025	-0.818
RBMXL2	-1.324	-1.398	-1.184
PIP5K1C	-0.994	-1.079	-0.903
MAPK8IP1	-1.171	-1.254	-1.062
STON1-GTF2A1L	-1.077	-1.187	-0.979
PRO1596	-1.006	-1.124	-0.941
TMEM173	-1.275	-1.419	-1.251
TMEM110	-0.941	-1.069	-0.908
TLX2	-1.799	-2.094	-1.813
PROK2	-2.045	-2.119	-2.13
EDNRB	-1.039	-1.053	-1.034
SRR	-1.256	-1.33	-1.269
C19orf47	-1.125	-1.241	-1.145
KIAA1407	-0.185	-0.041	-1.315
CALHM2	-0.348	-0.087	-1.912
hCG_1646157	-0.126	-0.065	-1.181
PAQR4	-0.152	0.034	-1.267
GALNTL4	-0.17	-0.303	-1.908
FLJ40330	-0.205	-0.308	-1.845
TRERF1	-0.136	-0.376	-1.967
C9orf140	-0.146	-0.451	-2.296
ZFP161	-0.17	-0.378	-2.167
CNO	-0.075	-0.212	-1.24
FLJ33630	-0.212	-0.194	-2.059
PEX12	-0.176	-0.186	-1.463
CCNO	-0.13	-0.154	-1.29
LACTB2	-0.043	-0.09	-1.097
BIN1	-0.075	-0.089	-1.345
FLJ38109	-0.076	-0.14	-1.129
TUSC1	-0.086	-0.128	-1.184
B3GNTL1	-0.113	-0.2	-1.919
TBC1D4	-0.072	-0.191	-1.728
ZNF524	-0.082	-0.035	-1.264
C9orf64	-0.025	-0.042	-1.489

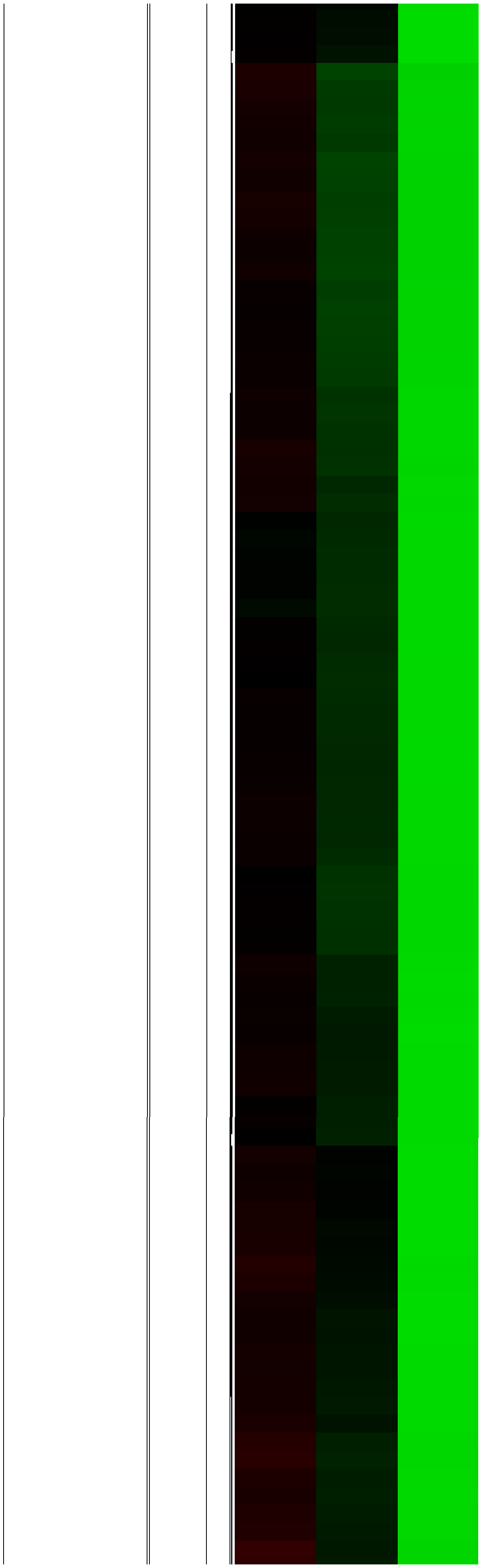

SHMT2	0.022	-0.073	-1.318
CCDC90B	0.013	-0.081	-1.273
ENDOG	0.029	-0.095	-1.105
IRAK4	0.143	-0.341	-1.047
GGCT	0.137	-0.299	-1.082
BMP6	0.213	-0.588	-2.173
TMCO6	0.119	-0.39	-1.375
CXXC4	0.153	-0.513	-1.892
IRAK1BP1	0.138	-0.445	-1.407
ELF3	0.1	-0.359	-1.177
SIPA1	0.112	-0.307	-1.039
CPNE4	0.174	-0.516	-1.71
FAM117B	0.088	-0.395	-1.291
CYP2R1	0.176	-0.867	-2.79
LOC283028	0.095	-0.38	-1.202
ZNF33B	0.078	-0.565	-1.954
NBN	0.053	-0.503	-1.657
MND1	0.058	-0.419	-1.405
MED11	0.058	-0.412	-1.396
MAT2A	0.074	-0.394	-1.407
C6orf114	0.073	-0.4	-1.442
TUBGCP5	0.108	-0.373	-1.59
C14orf128	0.104	-0.412	-1.694
WIPI1	0.071	-0.289	-1.226
CYP24A1	0.152	-0.296	-1.346
CBX8	0.154	-0.387	-1.624
C20orf177	0.198	-0.423	-2.262
B3GNT9	0.115	-0.258	-1.256
OSGEPL1	-0.04	-0.674	-3.682
C3orf63	-0.034	-0.201	-1.06
SLC16A5	-0.03	-0.315	-1.571
LOC729983	-0.042	-0.562	-2.798
C3orf72	-0.018	-0.223	-1.086
FBXL4	-0.061	-0.265	-1.285
NARS2	0.024	-0.294	-1.522
DLG3	0.063	-0.495	-2.722
WDR35	0.011	-0.242	-1.211
CDC7	0.008	-0.509	-2.473
LOC286254	0.047	-0.228	-1.147
HDHD2	0.044	-0.265	-1.383
FZD8	0.103	-0.598	-3.084
ZNF862	0.069	-0.349	-1.94
S100PBP	0.059	-0.25	-1.425
CHRNB1	0.05	-0.217	-1.178
LOC100270746	0.14	-0.456	-2.517
BCR	0.101	-0.311	-1.659
TMEM129	0.096	-0.365	-1.992
C3orf34	0.06	-0.216	-1.091
AKR1A1	0.01	-0.352	-1.492
ABCC5	0.023	-0.597	-2.471
MTMR9L	0.027	-0.264	-1.156
LOC100131564	0.035	-0.469	-2.154
HERC2P4	0.038	-0.362	-1.602
C18orf55	0.071	-0.163	-1.068
MTIF2	0.084	-0.257	-1.695
GTPBP3	0.06	-0.238	-1.504
PRMT2	0.072	-0.213	-1.62
MBLAC2	0.133	-0.416	-3.6
TMTC4	0.093	-0.159	-1.317
LIAS	0.114	-0.212	-1.705
C1orf66	0.1	-0.169	-1.322
MNX1	0.026	-0.257	-1.77
C9orf150	0.085	-0.448	-3.072
ZFP64	0.006	-0.16	-1.039
REP15	0.122	-0.019	-1.411
CLTCL1	0.106	-0.036	-1.535
C6orf120	0.16	-0.034	-2.131
MKNK2	0.116	-0.021	-1.125
PANK1	0.136	-0.053	-1.35
EPS8L2	0.145	-0.043	-1.253
EIF2S2	0.172	-0.049	-1.09
C12orf24	0.168	-0.065	-1.292
CELSR2	0.149	-0.105	-1.65
CAMK2G	0.09	-0.116	-1.225
BMS1P1	0.082	-0.085	-1.026
SMCR6	0.164	-0.163	-1.684
RIN1	0.159	-0.169	-1.748
NUDT6	0.216	-0.234	-2.198
KIAA0430	0.104	-0.125	-1.063
XYLT2	0.129	-0.089	-1.075
TMEM19	0.264	-0.224	-1.539
ANKRD50	0.216	-0.183	-1.14
HISPPD1	0.231	-0.246	-1.782
EID2B	0.118	-0.142	-1.025
SLC35B4	0.356	-0.319	-2.464
ARL3	0.163	-0.129	-1.072
ZNF823	0.44	-0.204	-1.89
CCDC25	0.24	-0.142	-1.129

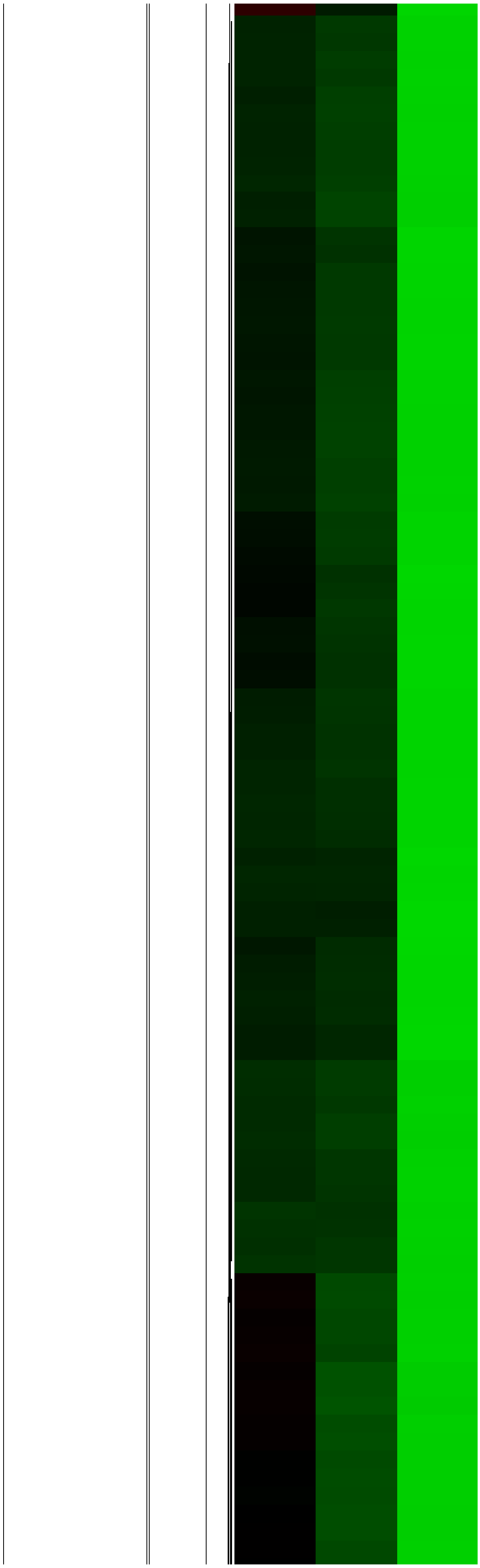

STRADB	-0.29	-0.484	-1.781	Down
LOC100131138	-0.323	-0.505	-1.926	Down
POLR3B	-0.265	-0.451	-1.575	Down
ANKRD36	-0.288	-0.468	-1.698	Down
RNF26	-0.16	-0.321	-1.051	Down
HEATR3	-0.41	-0.749	-2.431	Down
EHD3	-0.412	-0.742	-2.502	Down
N6AMT1	-0.333	-0.59	-2.004	Down
HORMAD1	-0.215	-0.377	-1.272	Down
hCG_1993592	-0.402	-0.654	-2.154	Down
NUDT7	-0.444	-0.95	-2.88	Down
NR1H3	-0.308	-0.621	-1.916	Down
TFB1M	-0.158	-0.398	-1.635	
ASCC1	-0.212	-0.458	-1.975	
GTF3A	-0.183	-0.529	-1.948	Down
CLDN23	-0.19	-0.516	-1.897	Down
LOC340508	-0.11	-0.285	-1.047	Down
ACTR3C	-0.19	-0.465	-1.689	Down
USP18	-0.257	-0.692	-2.538	Down
PRAGMIN	-0.122	-0.345	-1.267	Down
FZD4	-0.28	-0.755	-2.495	Down
COQ5	-0.158	-0.474	-1.537	Down
ZFP14	-0.222	-0.628	-2.001	Down
TSEN2	-0.192	-0.536	-1.686	Down
RPUSD3	-0.167	-0.429	-1.36	Down
MEN1	-0.18	-0.43	-1.416	Down
LOC729678	-0.254	-0.625	-2.078	Down
WDR24	-0.227	-0.549	-1.748	Down
OXSM	-0.141	-0.58	-2.082	
LYPLAL1	-0.181	-0.782	-2.751	
BMPR2	-0.081	-0.44	-1.592	
DPRXP4	-0.115	-0.7	-3.057	
BTBD11	-0.082	-0.661	-2.701	
ZBTB47	-0.083	-0.718	-2.716	
TRIM66	-0.153	-0.534	-2.13	
MAVS	-0.142	-0.452	-1.887	
SCRN3	-0.078	-0.297	-1.294	
JRKL	-0.084	-0.3	-1.291	
PPP1R3D	-0.213	-0.397	-1.578	Down
PIK3C2B	-0.448	-0.806	-3.245	Down
MSL3L2	-0.174	-0.268	-1.123	Down
FAM45A	-0.176	-0.27	-1.146	Down
LOC284219	-0.346	-0.498	-1.991	Down
hCG_1986447	-0.394	-0.508	-2.282	Down
C9orf123	-0.415	-0.514	-2.281	Down
C12orf26	-0.375	-0.457	-2.099	Down
SNX33	-0.351	-0.401	-1.93	Down
C9orf9	-0.167	-0.183	-1.076	
C2orf68	-0.516	-0.515	-2.862	
ACOXL	-0.307	-0.316	-1.812	
FAM177B	-0.661	-0.633	-4.438	
DPY19L1P1	-0.163	-0.163	-1.102	
STEAP2	-0.199	-0.368	-1.81	
GPR135	-0.223	-0.352	-1.704	
C11orf73	-0.147	-0.219	-1.043	
ZNF837	-0.172	-0.224	-1.098	
MRPS25	-0.236	-0.332	-1.612	
RCCD1	-0.186	-0.257	-1.422	
LOC344065	-0.15	-0.202	-1.125	
PIN4	-0.249	-0.325	-1.145	Down
ARV1	-0.395	-0.524	-1.828	Down
LOC151162	-0.422	-0.557	-2.058	Down
ZNF500	-0.37	-0.537	-1.792	Down
C16orf75	-0.231	-0.323	-1.064	Down
RPTOR	-0.217	-0.288	-1.103	Down
PLXND1	-0.207	-0.28	-1.084	Down
COMMD3	-0.218	-0.28	-1.126	Down
DHFRL1	-0.501	-0.476	-1.99	Down
ANKRD27	-0.276	-0.284	-1.174	Down
ITPKB	-0.529	-0.596	-2.301	Down
C10orf41	-0.519	-0.552	-2.09	Down
SKP2	0.101	-0.863	-2.446	
SC65	0.075	-0.549	-1.529	
NAIP	0.03	-0.373	-1.082	
LOC642413	0.037	-0.365	-1.06	
FAM82B	0.066	-0.497	-1.542	
HEATR5A	0.066	-1.022	-2.525	
BTBD12	0.053	-0.568	-1.437	
TNFRSF1A	0.069	-0.787	-1.9	
RAP2B	0.039	-0.564	-1.531	
LOC284232	0.033	-0.471	-1.231	
ZNF30	-0.015	-1.056	-2.95	Down
ITGB4	-0.015	-0.651	-1.77	Down
FAM20C	-0.044	-0.905	-2.493	Down
CCDC101	-0.001	-0.599	-1.591	Down
C5orf24	0.007	-0.434	-1.166	
TCEAL8	0.012	-0.545	-1.575	
LGSN	0.005	-0.544	-1.567	

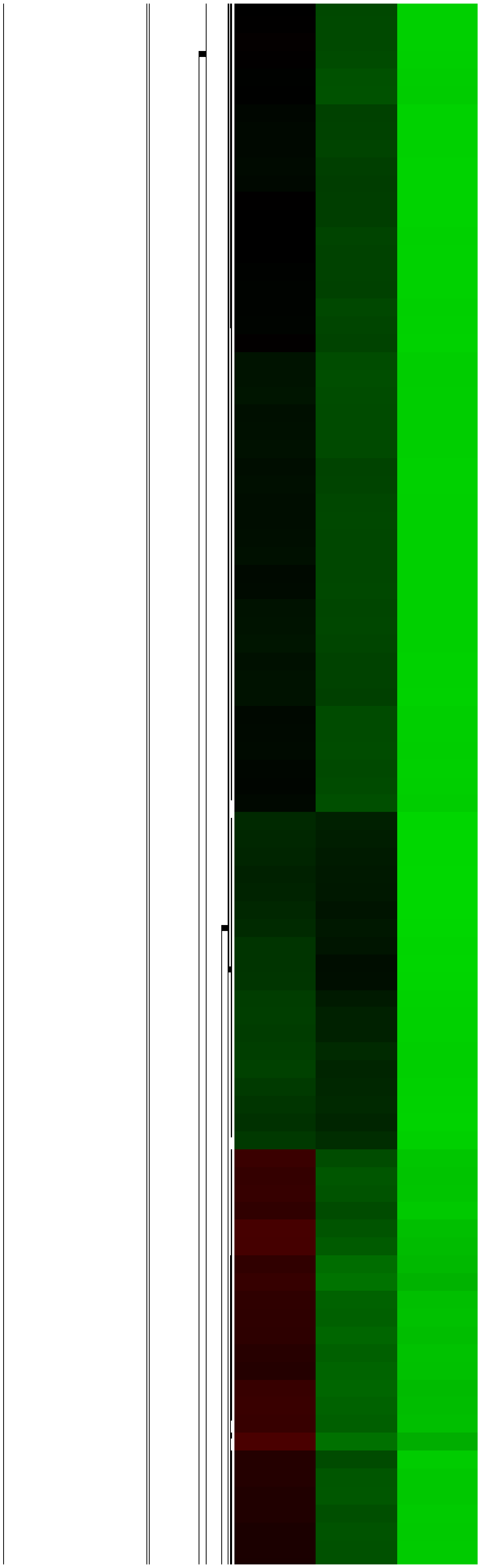

ESYT2	0.017	-0.659	-1.86	
TBC1D14	0.031	-0.463	-1.31	
SYNPO	0.015	-0.524	-1.446	
PCYOX1	-0.016	-0.517	-1.308	Down
MRPS34	-0.02	-0.91	-2.256	Down
HLA-DMB	-0.198	-1.866	-6.047	
GOLGA1	-0.074	-0.589	-1.855	
ZCCHC24	-0.071	-0.567	-1.739	Down
RWDD2A	-0.077	-0.491	-1.627	
LOC25845	-0.054	-0.405	-1.391	
RARB	0.012	-0.563	-1.903	
GLRX	-0.007	-0.766	-2.591	
MUCL1	-0.006	-0.4	-1.229	
hCG_1776007	-0.009	-0.85	-2.694	
DPYSL2	-0.028	-0.705	-2.24	
BTN3A2	-0.035	-0.608	-1.952	
PAR5	-0.018	-0.368	-1.065	
MPG	-0.03	-0.508	-1.543	
LOC728975	0.017	-0.396	-1.231	
MTR	-0.18	-0.721	-1.938	Down
C17orf90	-0.191	-0.752	-1.972	Down
THAP7	-0.286	-1.01	-2.726	Down
MFSD3	-0.297	-1.42	-3.891	Down
KCNQ3	-0.102	-0.462	-1.261	Down
TBL1Y	-0.098	-0.404	-1.131	Down
RNF141	-0.096	-0.49	-1.511	Down
HAUS1	-0.107	-0.466	-1.417	Down
PPA2	-0.089	-0.479	-1.395	Down
KCNJ6	-0.207	-1.098	-3.157	Down
BPGM	-0.089	-0.449	-1.302	Down
MRPS33	-0.108	-0.481	-1.394	Down
ZDHHC8	-0.066	-0.478	-1.401	Down
MIB1	-0.108	-0.722	-2.077	Down
FAM102A	-0.223	-0.853	-2.508	Down
DSCC1	-0.182	-0.682	-1.981	Down
RCAN3	-0.133	-0.421	-1.267	Down
IDH1	-0.256	-1.02	-3.235	Down
LOC100287820	-0.117	-0.41	-1.302	Down
FAM173A	-0.102	-0.353	-1.143	Down
LOC100130840	-0.051	-0.46	-1.263	Down
GLT8D1	-0.063	-0.497	-1.366	Down
SNPH	-0.149	-1.075	-2.914	Down
RAB40B	-0.041	-0.493	-1.395	Down
MMP15	-0.069	-0.875	-2.422	Down
PCP2	-0.051	-0.484	-1.244	Down
WNT5B	-0.234	-0.184	-1.222	
ACTA2	-0.214	-0.165	-1.165	
TRAK2	-0.442	-0.323	-2.515	
LOC150381	-0.242	-0.181	-1.544	
C9orf95	-0.375	-0.257	-2.269	
PTPDC1	-0.362	-0.191	-2.003	
C1RL	-0.283	-0.166	-1.429	
C1orf195	-0.305	-0.121	-1.23	
SLC7A11	-0.259	-0.065	-1.056	
C10orf104	-0.305	-0.083	-1.22	
UNQ1944	-0.645	-0.278	-2.216	Down
RNF187	-0.354	-0.193	-1.189	Down
MGC24103	-0.624	-0.348	-2.167	Down
TTC25	-0.523	-0.354	-1.731	Down
MGC16384	-0.617	-0.358	-1.966	Down
MOCOS	-0.458	-0.307	-1.635	Down
LOC100131089	-0.324	-0.251	-1.276	Down
CHURC1	-0.24	-0.178	-1.021	Down
ZNF573	-0.536	-0.423	-1.945	Down
CEP63	0.44	-0.579	-1.499	
FGG	0.304	-0.499	-1.137	
ELL2	0.324	-0.497	-1.174	
ZNF33A	0.24	-0.376	-1.008	
POU4F1	0.375	-0.453	-1.023	
CORO2A	0.784	-1.035	-2.12	
NDST2	0.33	-0.741	-1.251	
LOC730184	0.318	-0.673	-1.046	
NAT6	0.537	-1.124	-2.177	
FMO5	0.283	-0.58	-1.164	
LOC645101	0.249	-0.562	-1.045	
TMEM90B	0.406	-0.983	-1.991	
PLA2G4A	0.364	-0.988	-1.905	
SYT13	0.396	-0.741	-1.349	
RP5-1022P6.2	0.376	-0.652	-1.247	
NTN5	0.372	-0.634	-1.272	
KIAA0319	0.482	-0.732	-1.123	
CBR4	0.368	-0.762	-2.069	
STK16	0.254	-0.606	-1.406	
CCDC40	0.267	-0.716	-1.641	
SPRY1	0.161	-0.397	-1.012	
SLC39A11	0.143	-0.436	-1.062	
ZFYVE19	0.172	-0.505	-1.262	
PLCXD3	0.354	-1.089	-2.737	

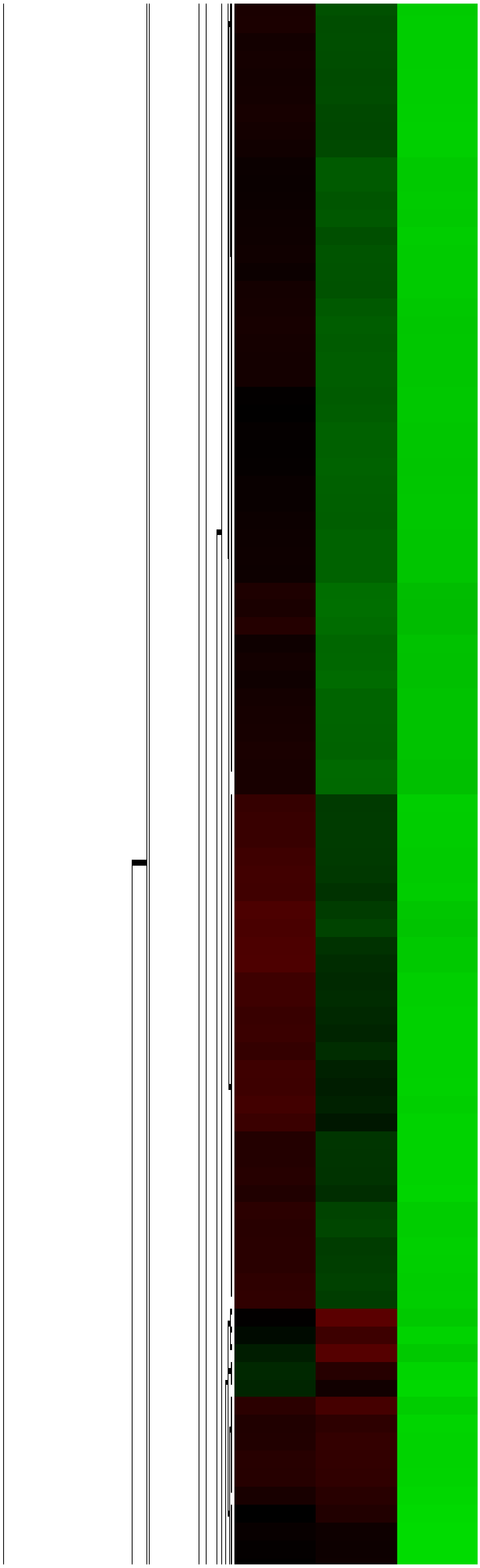

FAM129B	0.14	-0.398	-1.054	
GRB14	0.178	-0.7	-1.828	
C1orf35	0.182	-0.645	-1.717	
NAPEPLD	0.231	-0.912	-2.478	
MTMR15	0.154	-0.57	-1.54	
TARBP1	0.312	-0.945	-2.714	
PRDM12	0.162	-0.568	-1.662	
C11orf74	0.102	-0.436	-1.228	
PTPN3	0.064	-0.5	-1.11	
ANKS4B	0.076	-0.667	-1.487	Down
NHLRC3	0.131	-0.993	-2.346	
NAV1	0.177	-1.175	-2.685	
BRSK2	0.075	-0.418	-1.075	
VPS36	0.13	-0.668	-1.612	
OCEL1	0.209	-1.401	-3.411	
TSKU	0.231	-0.919	-2.264	
SNX18	0.118	-0.49	-1.106	
HOXB6	0.266	-1.041	-2.224	
PTPN13	0.162	-0.688	-1.498	
NBEAL2	0.166	-0.734	-1.571	
ANXA13	0.362	-1.647	-3.492	
NR2F6	0.025	-0.662	-1.455	Down
ICAM3	0.013	-0.739	-1.592	Down
FOXS1	0.127	-2.408	-4.92	Down
C4orf41	0.027	-0.555	-1.145	Down
GNPTAB	0.035	-0.595	-1.19	Down
RHOBTB2	0.117	-1.22	-2.504	Down
DOK4	0.105	-1.057	-2.213	Down
FAM161A	0.073	-0.567	-1.202	Down
KCNN4	0.087	-0.618	-1.243	Down
HPGD	0.185	-1.229	-2.457	Down
ARHGEF16	0.115	-0.856	-1.717	Down
STARD8	0.213	-0.779	-1.339	
BIVM	0.263	-1.126	-1.906	
OPHN1	0.319	-0.949	-1.648	
HOXB8	0.138	-0.954	-1.813	Down
LRRK2	0.268	-1.418	-2.646	
MARCH2	0.085	-0.574	-1.028	
CAMK2D	0.114	-0.548	-1.063	
ADD3	0.182	-0.809	-1.564	
ADAL	0.171	-0.718	-1.417	
ALPK1	0.311	-1.166	-2.322	
ST6GAL2	0.195	-0.832	-1.517	
ADAMTS9	0.139	-0.605	-1.116	
RAP1GAP	0.399	-0.432	-1.526	
ZYG11B	0.28	-0.299	-1.023	
CTSL1	0.324	-0.341	-1.186	
MTIF3	0.389	-0.367	-1.273	
IQCD	0.378	-0.328	-1.183	
FILIP1	0.573	-0.446	-1.823	
SLITRK6	0.831	-0.656	-2.17	
LMO4	0.411	-0.38	-1.104	
CEP250	0.408	-0.268	-1.077	
CACNG6	0.493	-0.28	-1.281	
DBT	0.34	-0.226	-1.135	
ALS2CR8	0.476	-0.338	-1.572	
SLC35A5	0.321	-0.232	-1.198	
B3GNT1	0.517	-0.318	-1.837	
BBS2	0.41	-0.353	-1.63	
NOTCH4	0.322	-0.172	-1.084	
ADCY9	0.371	-0.18	-1.271	
DAG1	0.414	-0.212	-1.302	
NEBL	0.306	-0.124	-1.115	
PBXIP1	0.183	-0.273	-1.083	
C5orf45	0.205	-0.3	-1.207	
LIMD1	0.198	-0.264	-1.075	
hCG_1817306	0.158	-0.222	-1.05	
TNS4	0.527	-0.823	-2.489	
BDKRB2	0.518	-0.898	-2.626	
LOC220930	0.225	-0.331	-1.134	
KANK2	0.277	-0.421	-1.403	
TOP2B	0.297	-0.421	-1.318	
SLC7A2	0.312	-0.396	-1.33	
ZNF605	0.014	0.469	-1.038	
NCRNA00169	-0.049	0.294	-1.019	
ATP13A5	-0.156	0.451	-1.063	
XBP1	-0.221	0.208	-1.168	
LOC346887	-0.196	0.093	-1.136	
PDE5A	0.261	0.414	-1.231	
ANKRD29	0.249	0.354	-1.645	
RFXAP	0.184	0.281	-1.148	
ZNF302	0.329	0.417	-1.754	
RPH3AL	0.252	0.315	-1.384	
C15orf52	0.215	0.353	-1.881	
GPX8	-0.001	0.174	-1.128	
LOC91450	0.052	0.085	-1.322	
LOC100130093	0.024	0.069	-1.146	
HDAC4	0.014	0.077	-1.19	

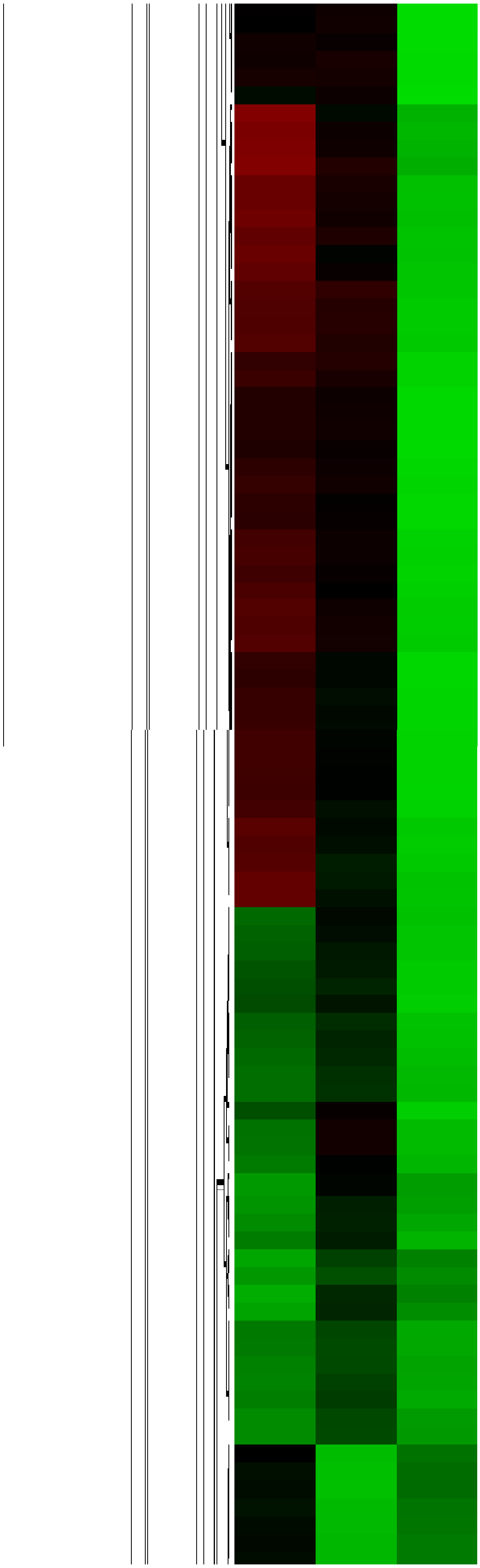

BBX	0.009	0.084	-1.136	
PIK3R1	0.106	0.054	-1.345	
SMARCA2	0.067	0.119	-1.007	
RSPO3	0.11	0.103	-1.045	
ANKRD5	-0.07	0.081	-1.323	
HMMR	0.811	-0.06	-1.11	
SDS	1.69	0.175	-2.532	
PGM2L1	0.818	0.096	-1.148	
HOXA11AS	0.77	0.205	-1.024	
SMAD6	0.558	0.133	-1.023	
ANG	0.579	0.118	-1.078	
RAB27B	0.765	0.115	-1.316	
NIN	0.521	0.166	-1.052	
OBFC1	0.876	-0.029	-1.625	
HLTF	0.608	0.055	-1.231	
DDIT4	0.862	0.508	-2.067	
IGFBP1	0.429	0.197	-1.091	
C12orf76	0.51	0.253	-1.32	
PARD6A	0.488	0.199	-1.188	
SFRP1	0.274	0.204	-1.169	
ITPRIPL2	0.291	0.127	-1.056	
PPARGC1B	0.28	0.102	-1.774	
PABPC4L	0.224	0.096	-1.369	
KLHL3	0.201	0.099	-1.26	
CCDC111	0.18	0.051	-1.266	
TRIM65	0.322	0.099	-1.537	
LOC100129233	0.359	0.112	-1.469	
THBD	0.374	0.029	-1.765	
LRFN4	0.269	0.046	-1.337	
PCMTD2	0.481	0.094	-1.483	
ACN9	0.417	0.073	-1.207	
CETN3	0.454	0.058	-1.543	
PLD1	0.594	0.015	-1.621	
SLC22A18AS	0.434	0.073	-1.065	
RDH10	0.498	0.121	-1.311	
ATP11A	0.424	0.1	-1.042	
MIPOL1	0.273	-0.043	-1.197	
ANO5	0.307	-0.044	-1.454	
LETMD1	0.278	-0.071	-1.099	
C11orf86	0.69	-0.106	-2.709	
LOC344887	0.518	-0.099	-1.933	
IFT80	0.365	-0.068	-1.372	
FLJ27352	0.719	-0.058	-2.338	
GPRIN3	0.489	-0.025	-1.608	
FANCL	0.327	-0.015	-1.088	
LGR4	0.574	-0.026	-2.023	
SDPR	0.524	-0.119	-1.644	
TTC37	0.474	-0.053	-1.075	
MPHOSPH9	0.464	-0.084	-1.166	
LOC100128822	0.574	-0.197	-1.361	
FLRT3	0.56	-0.149	-1.116	
EHBP1L1	0.551	-0.09	-1.1	
SLC35B3	-1.028	-0.086	-1.877	
LOC157860	-1.033	-0.138	-2.069	
CHML	-0.521	-0.135	-1.073	
SLC10A4	-0.474	-0.151	-1.137	
CASP4	-0.494	-0.231	-1.281	Down
CAD	-0.38	-0.101	-1.04	
TMEM62	-0.676	-0.321	-1.375	
NPM3	-0.537	-0.195	-1.043	
CCDC92	-0.638	-0.241	-1.134	
KCTD21	-0.761	-0.329	-1.283	
GHRLOS	-0.775	-0.344	-1.297	
PRDM13	-0.42	0.038	-1.102	
HPR	-0.752	0.124	-1.235	
FAM129A	-1.323	0.208	-2.157	
ARGFX	-0.697	-0.013	-1.032	
PCDHB7	-1.233	-0.047	-1.261	
LYSMD4	-1.218	-0.249	-1.296	
C10orf79	-1.249	-0.303	-1.506	
TMEM213	-0.757	-0.172	-1.095	
MS4A10	-1.011	-0.39	-0.796	
LOC727677	-1.762	-0.931	-1.615	
FMNL3	-1.021	-0.241	-0.763	
CD86	-1.298	-0.299	-1.127	
PSD	-1.019	-0.592	-1.42	
DEM1	-1.404	-0.851	-1.926	
CAMSAP1	-0.804	-0.454	-1.013	
C3AR1	-0.801	-0.392	-1.004	
MAGEA8	-0.862	-0.405	-1.16	
NDUFS2	-1.028	-0.537	-1.147	
KIAA1659	-1.018	-0.535	-1.122	
DECR2	0.006	-1.068	-0.65	
TM4SF20	-0.273	-3.483	-1.969	
LOC731656	-0.065	-1.021	-0.57	
RFX2	-0.111	-1.066	-0.668	
CREB3L2	-0.074	-1.184	-0.757	
SLC17A3	-0.051	-1.061	-0.712	

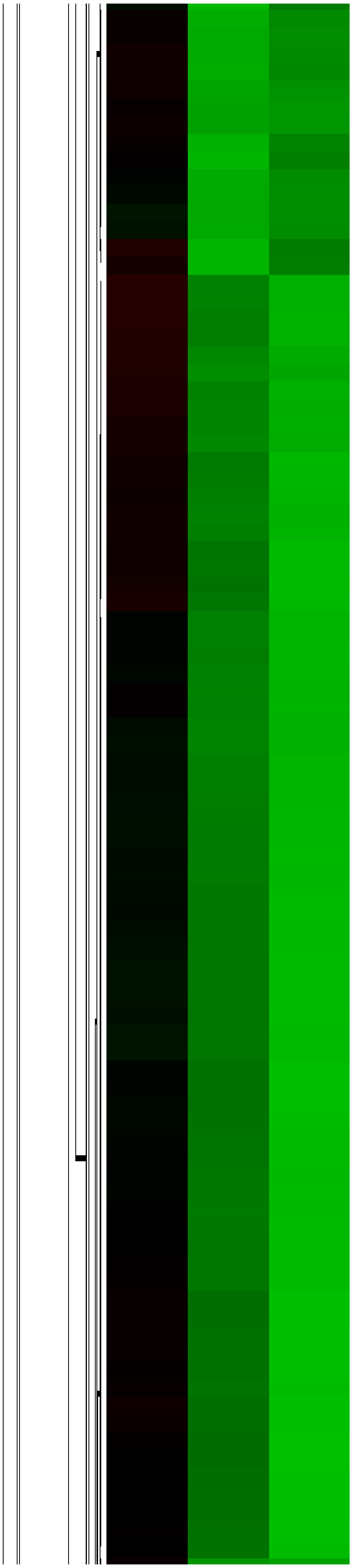

PODXL2	-0.073	-1.047	-0.702	
LOC401980	0.1	-1.841	-1.477	
DEC1	0.062	-1.126	-0.943	
PLCD4	0.172	-1.64	-1.341	
CD14	0.19	-2.028	-1.606	
UGT1A8	0.093	-1.069	-0.948	
ZDHHC8P	0.125	-2.484	-2.309	
PRKAR2B	0.097	-1.189	-1.124	
SLC6A12	0.05	-1.315	-0.99	
LOC100134361	0.015	-1.208	-0.849	
PIGW	-0.029	-1.202	-0.989	
HHAT	-0.09	-1.603	-1.345	
PCSK4	-0.141	-1.155	-0.968	
GMDS	-0.156	-1.518	-1.253	
MAN1C1	0.198	-1.134	-0.78	
BTBD16	0.172	-1.429	-0.988	
SEMA3B	0.312	-1.106	-1.513	
AKR1B10	0.236	-0.801	-1.088	
PDE4B	0.311	-1.028	-1.468	
GDPD2	0.197	-0.747	-1.06	
PLCE1	0.247	-1.021	-1.29	
LPGAT1	0.177	-0.857	-1.01	
OAS1	0.434	-2.157	-2.931	
HSD11B1L	0.234	-1.163	-1.51	
PON1	0.114	-0.789	-1.031	
FANCI	0.119	-0.792	-1	
C9orf37	0.164	-1.193	-1.785	
C11orf71	0.198	-1.523	-2.245	
C2orf27A	0.113	-1.077	-1.531	
KIAA0101	0.132	-0.99	-1.354	
EHF	0.12	-0.862	-1.224	
MMD	0.11	-0.885	-1.418	
C19orf73	0.097	-0.825	-1.292	
MKRNP6	0.217	-1.29	-2.119	
MAP3K12	0.287	-1.396	-2.158	
THSD7A	-0.053	-1.721	-2.41	
HYAL2	-0.03	-1.252	-1.746	
CCDC80	-0.052	-1.741	-2.533	
SOX21	-0.128	-2.29	-3.183	
PEX1	0.026	-1.245	-1.705	
AKR1B15	0.019	-0.911	-1.267	
SCRN2	-0.095	-0.971	-1.307	
GLE1	-0.116	-1.098	-1.461	
LOC92973	-0.092	-0.972	-1.386	
IL1R1	-0.08	-0.806	-1.155	
PHF11	-0.1	-0.917	-1.327	Down
FAM162A	-0.111	-0.919	-1.355	Down
SPATA7	-0.188	-1.632	-2.428	Down
SCARA3	-0.088	-0.994	-1.483	Down
PTGFRN	-0.124	-1.345	-1.997	Down
tcag7.1196	-0.118	-1.332	-2.038	Down
ITPR1	-0.077	-1.071	-1.659	Down
SKAP1	-0.076	-0.767	-1.177	Down
ABCB9	-0.106	-0.943	-1.438	Down
SYTL2	-0.243	-1.725	-2.719	Down
SESTD1	-0.137	-0.948	-1.494	Down
EPHX1	-0.119	-0.794	-1.239	Down
C1orf21	-0.124	-1.114	-1.767	Down
SLC12A2	-0.17	-1.009	-1.553	Down
RASGEF1A	-0.141	-0.797	-1.251	Down
SLC40A1	-0.054	-1.514	-2.509	Down
KLHL13	-0.022	-0.734	-1.226	Down
SLC46A3	-0.045	-0.805	-1.351	Down
NNMT	-0.136	-2.317	-3.8	Down
ZNF248	-0.038	-0.863	-1.384	Down
CXorf66	-0.023	-0.727	-1.164	Down
TMEM160	-0.029	-0.789	-1.205	
SLC25A28	-0.034	-0.764	-1.182	Down
ADSSL1	-0.012	-1.271	-1.93	
TC2N	0.004	-1.613	-2.502	
OSTbeta	0.012	-1.953	-3.098	
GABRB3	0.032	-1.864	-2.914	
DCAF17	0.025	-0.673	-1.058	
C1QTNF6	0.137	-1.725	-3.029	Down
ARHGAP26	0.069	-1.195	-2.062	Down
PPM1M	0.091	-1.169	-1.975	
IFT81	0.066	-0.945	-1.598	
PPFIBP2	0.032	-0.757	-1.257	
PDGFRL	0.042	-0.749	-1.219	
ORC1L	0.075	-0.595	-1.012	
MAN1A2	0.068	-0.662	-1.125	
KIAA0528	0.029	-0.923	-1.64	Down
ANKMY2	0.009	-0.761	-1.357	Down
KDELC1	-0.002	-1.069	-1.851	Down
CUEDC2	0.002	-0.596	-1.016	Down
SSPN	0.007	-0.736	-1.261	Down
RHOQ	0.019	-0.906	-1.525	Down
RHOV	0.019	-2.128	-3.49	Down

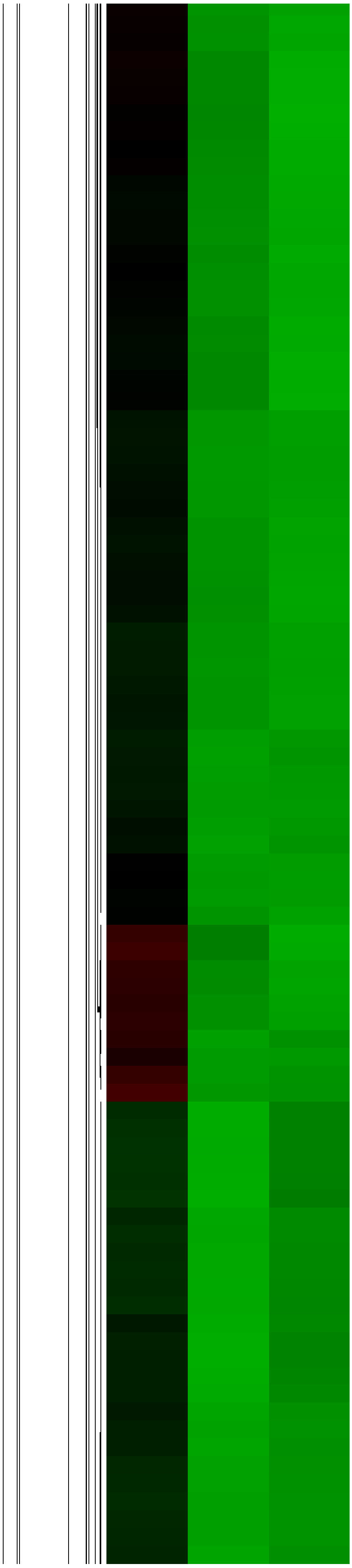

MATN2	0.104	-1.429	-1.546
FAM108A1	0.061	-1.003	-1.164
C14orf132	0.087	-1.897	-2.153
GALNT11	0.123	-1.368	-1.71
PPIL3	0.076	-1.118	-1.421
PDE1A	0.083	-1.333	-1.703
SLC26A9	0.043	-1.501	-1.952
SIDT1	0.089	-2.485	-3.214
PTGR1	0.004	-0.901	-1.118
AKR1B1	0.034	-0.892	-1.094
HNRNPA1	-0.058	-1.024	-1.231
CA12	-0.091	-1.206	-1.422
FANK1	-0.056	-0.995	-1.164
DCDC2	-0.076	-1.308	-1.509
C20orf46	-0.041	-1.64	-1.981
ALDH7A1	-0.009	-1.375	-1.607
C20orf96	-0.022	-0.869	-1.005
ADCK4	-0.045	-1.133	-1.316
GPX2	-0.17	-2.679	-3.311
C21orf84	-0.121	-1.439	-1.765
CRYAB	-0.182	-2.456	-3.122
SGCD	-0.072	-2.081	-2.6
LACE1	-0.031	-1.113	-1.42
LOC729970	-0.217	-1.705	-1.787
HYAL1	-0.183	-1.333	-1.404
GTF2I	-0.14	-1.172	-1.207
C18orf1	-0.192	-1.781	-1.834
CYB5A	-0.129	-1.44	-1.496
CDK5	-0.105	-1.43	-1.509
LRP4	-0.174	-1.541	-1.7
ACCN2	-0.121	-0.964	-1.062
ATP8A1	-0.147	-1.417	-1.578
VSTM2L	-0.174	-1.86	-2.11
MBOAT2	-0.084	-0.869	-1.007
ROM1	-0.129	-1.041	-1.195
TP63	-0.228	-1.149	-1.233
MAGED1	-0.27	-1.46	-1.569
C10orf58	-0.191	-1.059	-1.128
PEBP1	-0.177	-1.067	-1.14
FGB	-0.176	-1.204	-1.307
ALDH5A1	-0.215	-1.437	-1.567
MKL2	-0.2	-1.126	-1.075
GALM	-0.458	-2.873	-2.671
UGT2B11	-0.164	-1.102	-1.058
OMA1	-0.341	-2.074	-2.045
KCNK3	-0.168	-1.19	-1.186
SLC27A5	-0.105	-1.116	-1.075
METTL7A	-0.223	-2.111	-1.958
LOC282997	-0.015	-1.484	-1.491
FAM131B	-0.003	-1.645	-1.671
NOTCH3	-0.056	-1.735	-1.745
EPB49	-0.016	-1.031	-1.126
LOC100132707	0.319	-0.762	-1.037
DBP	0.736	-1.514	-2.031
STAT1	0.338	-0.994	-1.164
CNNM1	0.313	-0.99	-1.175
LOC100133487	0.411	-1.446	-1.625
CCDC89	0.286	-0.905	-1.002
RAC3	0.302	-1.184	-1.07
LOC727938	0.295	-1.699	-1.695
SLC1A7	0.413	-1.192	-1.131
CP	0.462	-1.053	-1.021
MUDENG	-0.283	-1.143	-0.865
C9orf116	-0.549	-2.007	-1.524
LOC100128163	-0.314	-1.085	-0.832
SLC23A1	-0.495	-1.681	-1.275
GALNT12	-0.604	-2.13	-1.591
RSPH1	-0.307	-1.04	-0.748
TIMP4	-0.232	-1.007	-0.835
LOC93463	-0.276	-1.019	-0.846
PRR3	-0.395	-1.617	-1.303
NOTCH2	-0.385	-1.52	-1.226
DHCR7	-0.4	-1.644	-1.317
KLHDC9	-0.671	-2.498	-1.993
CAT	-0.239	-1.647	-1.315
CD24	-0.411	-2.161	-1.656
ABCC6	-0.202	-1.104	-0.836
SPON2	-0.207	-1.178	-0.913
ITGA11	-0.202	-1.144	-0.915
INO80B	-0.283	-1.853	-1.623
TNFRSF21	-0.215	-1.128	-1.02
CRYM	-0.728	-3.923	-3.41
MOSPD3	-0.334	-1.356	-1.215
FRAS1	-0.416	-1.65	-1.484
RILP	-0.299	-1.135	-1.041
LAD1	-0.549	-2.221	-2.059
IGBP1	-0.338	-1.407	-1.302
NTRK3	-0.56	-2.544	-2.218

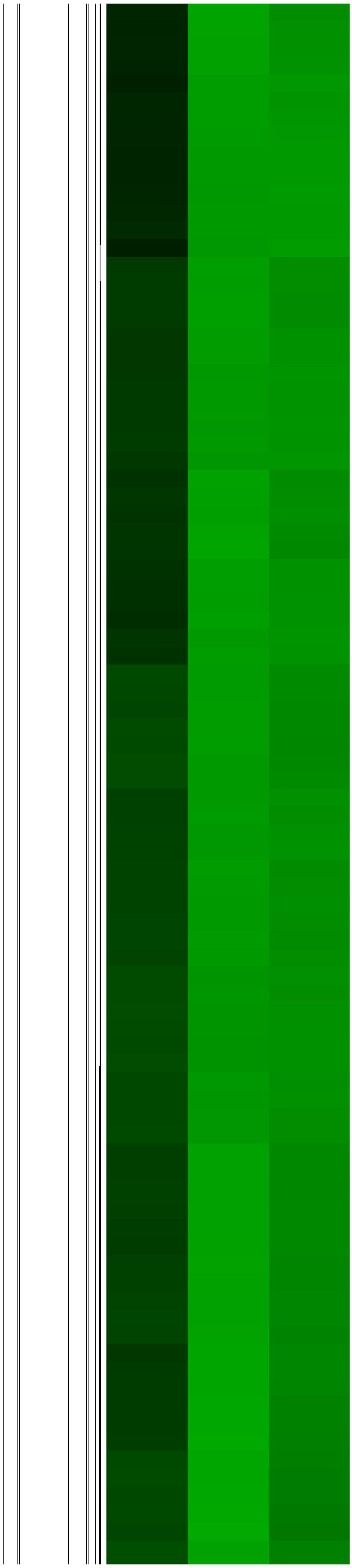

CCND3	-0.335	-1.453	-1.262	
SUMF1	-0.269	-1.195	-1.055	
TRIM29	-0.361	-1.562	-1.393	
LOC401630	-0.267	-1.173	-1.068	
SOX2	-0.248	-1.187	-1.143	
TMEM149	-0.534	-2.196	-2.08	
ASB9	-0.349	-1.469	-1.41	
VSIG10L	-0.539	-2.225	-2.164	
GALNTL1	-0.234	-1.009	-0.997	
ENPP1	-0.274	-1.166	-1.158	
PLD5	-0.328	-1.341	-1.37	
PLAT	-0.549	-2.118	-2.114	
LLGL2	-0.288	-1.023	-1.023	
ENTPD2	-0.261	-1.268	-1.295	
KCTD17	-0.64	-1.737	-1.565	
HCFC1R1	-0.388	-1.018	-0.922	
NECAB3	-0.423	-1.133	-1.003	
LOC402779	-0.434	-1.163	-1.028	
TMEM97	-0.534	-1.475	-1.374	
LOC100133145	-0.436	-1.2	-1.117	
THTPA	-0.512	-1.386	-1.351	
SLC25A29	-0.707	-1.859	-1.79	
FGD5	-0.627	-1.659	-1.589	
ICK	-0.787	-2.025	-1.994	
GGT5	-0.424	-1.07	-1.027	
SNAP25	-0.655	-1.737	-1.736	
ANXA6	-1.043	-3.439	-2.974	
LOC388242	-0.621	-1.813	-1.599	
ACY1	-0.451	-1.39	-1.246	
HSP90AA2	-0.476	-1.446	-1.22	
FADS2	-0.434	-1.354	-1.117	
TLE2	-0.383	-1.204	-1.108	
SLC5A11	-0.647	-2.108	-1.935	
KIF12	-0.707	-2.315	-2.154	
FBN2	-1.022	-3.554	-3.264	
DMRTC1	-0.452	-1.334	-1.285	
C9orf3	-0.467	-1.45	-1.367	
LOC646976	-0.633	-1.341	-1.187	
FUZ	-0.668	-1.44	-1.279	
DPYSL5	-1.25	-2.783	-2.439	
TSTA3	-0.885	-1.84	-1.611	
C17orf62	-0.79	-1.676	-1.436	
MYD88	-0.564	-1.124	-0.995	
DHFR	-0.564	-1.139	-1.027	
MRAP2	-1.437	-3.447	-3.231	
HSPB1	-0.449	-1.088	-0.997	
SLC1A1	-0.741	-1.692	-1.612	Down
RAB26	-0.865	-1.999	-1.933	Down
GYG2	-0.883	-2.052	-1.84	
GHDC	-0.444	-1.048	-0.953	
RNF144B	-0.462	-1.069	-0.995	Down
ZWINT	-0.494	-1.084	-1.001	Down
RASSF5	-0.578	-1.279	-1.16	Down
NCALD	-0.496	-1.113	-1.025	Down
SRD5A3	-0.704	-1.387	-1.342	Down
SLC25A10	-0.955	-1.919	-1.818	Down
RECQL4	-1.038	-2.005	-1.945	Down
CAND2	-0.862	-1.709	-1.665	Down
WDR54	-0.76	-1.501	-1.479	Down
SLFN11	-1.736	-3.334	-3.308	Down
PCDHB11	-0.514	-1.1	-1.036	Down
FH	-0.502	-1.058	-1.015	Down
ASL	-0.602	-1.261	-1.187	Down
ADPRHL1	-1.171	-2.394	-2.236	Down
CGNL1	-0.866	-2.218	-1.878	
CCDC24	-0.512	-1.296	-1.103	
TMEM141	-0.466	-1.154	-0.972	
ERBB3	-0.692	-1.757	-1.486	
GAMT	-0.579	-1.534	-1.304	
BPHL	-0.554	-1.481	-1.243	
NEURL2	-0.565	-1.424	-1.165	
EML4	-0.541	-1.341	-1.103	
PACSIN1	-1.572	-3.701	-3.079	
CBLN2	-0.96	-2.24	-1.87	
CRELD2	-0.469	-1.115	-0.896	
NUP133	-0.391	-1.137	-0.922	
LOC339807	-0.366	-1.023	-0.83	
HSP90AA1	-0.507	-1.411	-1.138	
NAMPT	-0.682	-1.933	-1.508	
FAM105A	-0.382	-1.055	-0.817	
OSBPL9	-0.489	-1.335	-1.002	
SLC44A3	-0.833	-1.852	-1.415	
ABCD3	-0.564	-1.233	-0.92	
NDRG4	-1.315	-3.083	-2.298	
SLC29A4	-0.438	-1.005	-0.726	
FLJ37798	-0.926	-2.243	-1.584	
FNTB	-0.582	-1.194	-0.946	
DDC	-1.132	-2.401	-1.87	

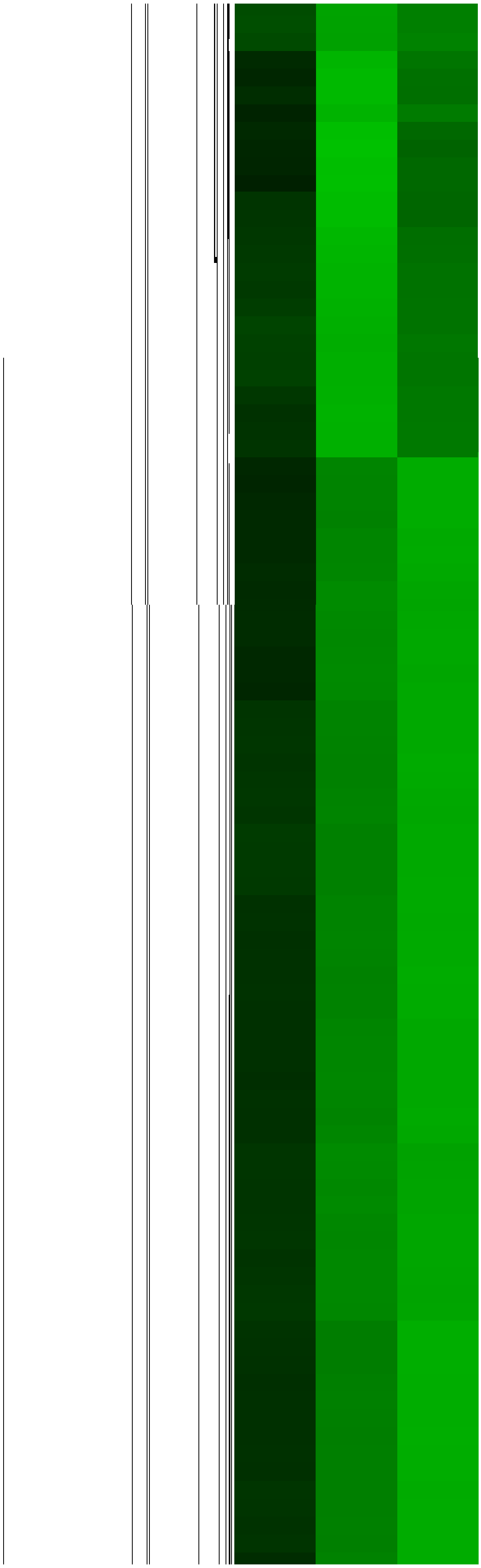

C19orf23	-0.597	-1.243	-0.971	
NDN	-0.517	-1.13	-0.913	
TM4SF5	-0.3	-1.273	-0.824	
SEPW1	-0.356	-1.706	-1.035	
C5orf35	-0.275	-1.112	-0.669	
HLF	-0.323	-1.637	-1.131	
NFKBIZ	-0.26	-1.195	-0.657	
HJURP	-0.225	-1.13	-0.578	
FLJ40125	-0.344	-1.775	-0.972	
AP4M1	-0.185	-1.081	-0.588	
MFSD4	-0.35	-1.253	-0.682	
MAN2B2	-0.38	-1.347	-0.723	
LOC643988	-0.591	-1.953	-1.175	
LOC285577	-0.458	-1.441	-0.887	
MEIG1	-0.414	-1.252	-0.795	
FGGY	-0.492	-1.531	-0.978	
LOC553137	-0.812	-2.333	-1.511	
U2AF1	-0.412	-1.055	-0.691	
PHTF1	-0.603	-1.608	-1.105	
PCOLCE	-0.564	-1.537	-1.024	
KIAA0317	-0.386	-1.04	-0.701	
PLSCR3	-0.364	-1.157	-0.789	
LOC728392	-0.636	-2.284	-1.536	
RNF125	-0.308	-1.072	-0.733	
LOC285501	-0.494	-1.659	-1.137	
ZNF219	-0.236	-0.783	-1.018	Down
LPPR3	-0.264	-0.919	-1.205	Down
CCDC106	-0.612	-1.99	-2.616	Down
SEPT6	-0.394	-1.244	-1.666	Down
FAM175A	-0.708	-2.268	-2.901	Down
C10orf10	-0.349	-1.11	-1.426	Down
TMEM143	-0.585	-1.767	-2.233	Down
PHEX	-0.472	-1.552	-1.854	Down
KIAA0146	-0.272	-0.874	-1.033	Down
MED25	-0.266	-0.83	-1.01	Down
C12orf27	-1.006	-3.096	-3.816	Down
SUOX	-0.556	-1.863	-2.287	Down
LQK1	-0.542	-1.864	-2.238	Down
VASH2	-0.37	-1.31	-1.598	Down
NAGPA	-0.34	-0.849	-1.095	Down
CNPY4	-0.386	-0.954	-1.229	Down
ZCWPW1	-0.692	-1.67	-2.171	Down
PRRC1	-0.466	-1.15	-1.519	Down
GALNT4	-0.68	-1.623	-2.141	Down
ZNF581	-0.476	-1.13	-1.448	Down
RAB37	-0.915	-2.259	-2.852	Down
LRRC8D	-0.686	-1.493	-1.963	Down
IFT122	-0.435	-0.948	-1.253	Down
EEF2	-0.397	-0.874	-1.157	Down
CYP2U1	-0.906	-2.014	-2.67	Down
SYT11	-0.573	-1.531	-1.982	Down
POP7	-0.353	-0.911	-1.175	Down
PAPSS1	-0.374	-1.015	-1.305	Down
MYO1F	-0.47	-1.264	-1.629	Down
GOLSYN	-0.817	-2.145	-2.851	Down
GNAI2	-0.332	-0.857	-1.125	Down
SFMBT2	-0.546	-1.475	-1.941	Down
LIG1	-0.584	-1.622	-2.047	Down
LDHD	-0.492	-1.372	-1.713	Down
C6orf165	-0.289	-0.806	-1.013	Down
ARMC7	-0.294	-0.848	-1.054	Down
CYB5D2	-0.313	-0.849	-1.073	Down
ACAT2	-0.565	-1.526	-1.972	Down
SETBP1	-1.018	-2.844	-3.567	Down
TIAM1	-0.583	-1.52	-1.769	Down
SLCO2B1	-0.478	-1.234	-1.464	Down
PHKA2	-0.513	-1.332	-1.604	Down
KRT4	-0.778	-2.06	-2.449	Down
FLJ10357	-0.494	-1.242	-1.529	Down
EIF2AK1	-0.376	-0.931	-1.16	Down
CDK5RAP3	-0.418	-1.105	-1.35	Down
C17orf97	-1.381	-3.522	-4.284	Down
SLC4A11	-0.605	-1.482	-1.791	Down
RDX	-0.418	-0.997	-1.221	Down
HOXA13	-0.367	-0.891	-1.245	Down
ACCS	-0.548	-1.361	-1.903	Down
SNX21	-0.521	-1.327	-1.846	Down
USH1C	-0.527	-1.423	-1.93	Down
RASSF4	-0.823	-2.158	-2.913	Down
PLCD1	-0.843	-2.24	-3.039	Down
PECR	-0.388	-1.014	-1.393	Down
ETV2	-0.409	-1.062	-1.447	Down
DAK	-0.371	-0.987	-1.341	Down
MORC4	-0.991	-2.386	-3.205	Down
FCRLB	-0.391	-0.954	-1.29	Down
DPYSL3	-0.709	-1.795	-2.406	Down
DHRS1	-0.559	-1.348	-1.83	Down
ZNF691	-0.298	-0.88	-1.169	Down

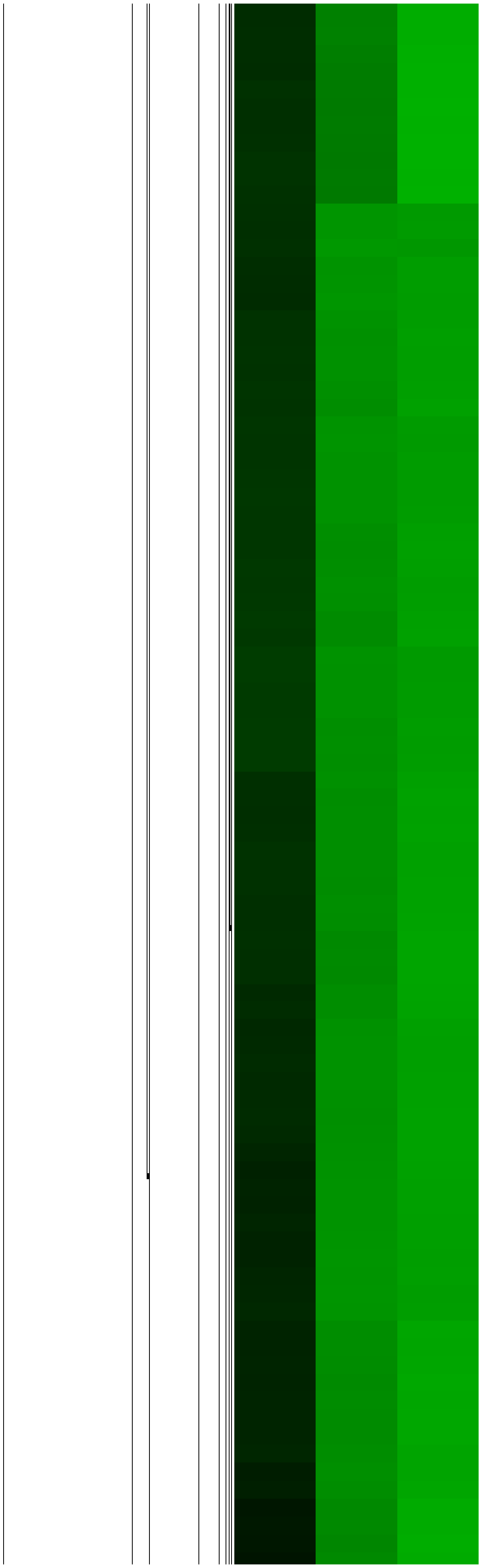

THYN1	-0.74	-2.158	-2.906	Down
NAT1	-0.603	-1.715	-2.295	Down
LOC388588	-0.444	-1.227	-1.699	Down
KRTAP19-8	-0.612	-1.712	-2.422	Down
STAT4	-0.507	-1.268	-1.813	Down
PLEKHG4	-0.628	-1.621	-2.348	Down
FBXO9	-0.654	-1.702	-2.441	Down
FAH	-0.295	-0.754	-1.087	Down
PBLD	-0.349	-0.818	-1.196	Down
C15orf58	-0.557	-1.314	-1.9	Down
TLE6	-0.466	-1.14	-1.665	Down
PTOV1	-0.371	-1.14	-1.175	
CCT3	-0.375	-1.185	-1.23	
MNS1	-0.807	-2.571	-2.553	
EPHX2	-0.777	-2.518	-2.692	
C17orf70	-0.323	-1.08	-1.148	
LOC100130906	-0.365	-1.261	-1.312	
PCSK1	-0.455	-1.315	-1.421	Down
DZIP3	-0.376	-1.08	-1.189	Down
CYP2S1	-0.43	-1.214	-1.321	Down
C19orf57	-0.358	-1.042	-1.128	Down
SLC22A3	-0.87	-2.388	-2.654	Down
GMPPA	-0.903	-2.47	-2.804	Down
NTNG2	-0.978	-2.706	-2.861	Down
BMP4	-0.773	-2.175	-2.253	
NLE1	-0.486	-1.352	-1.443	Down
MAP2K6	-1.286	-3.469	-3.678	Down
C14orf73	-0.678	-1.803	-1.908	Down
C10orf140	-0.519	-1.386	-1.478	Down
IFIT1	-0.89	-2.339	-2.612	Down
C2CD4B	-0.399	-1.027	-1.167	Down
NUDT18	-1.431	-3.636	-4.071	Down
C12orf52	-0.655	-1.717	-1.866	Down
ADCK5	-0.701	-1.794	-1.975	Down
PDLIM1	-0.545	-1.299	-1.498	Down
MAGED4B	-0.883	-2.184	-2.525	Down
PRKD1	-0.428	-1.038	-1.093	Down
C21orf125	-0.542	-1.313	-1.395	Down
APC2	-0.618	-1.544	-1.643	Down
ADORA2B	-0.468	-1.164	-1.238	Down
PCYT2	-1.167	-2.782	-3.062	Down
C3orf18	-1.504	-3.627	-3.95	Down
USP9X	-0.983	-2.362	-2.617	Down
TMEM37	-1.426	-4.374	-4.856	Down
GPR162	-0.794	-2.375	-2.733	Down
AP1G2	-0.466	-1.432	-1.621	Down
SLC38A6	-0.319	-0.958	-1.092	Down
KCTD18	-0.489	-1.389	-1.566	Down
C11orf35	-0.671	-1.932	-2.204	Down
VASH1	-1.316	-3.697	-4.287	Down
SIDT2	-0.697	-2.101	-2.39	Down
MYOM3	-0.544	-1.602	-1.853	Down
PON3	-0.311	-0.879	-1.058	Down
CCDC34	-0.307	-0.888	-1.057	Down
RIBC2	-0.719	-2.083	-2.506	Down
LOC554202	-0.507	-1.715	-1.988	Down
KCNJ8	-0.385	-1.232	-1.417	Down
KIAA0495	-0.343	-1.227	-1.35	
FNDC4	-0.329	-1.178	-1.279	
CD99L2	-0.392	-1.319	-1.44	
AGTRAP	-0.27	-0.956	-1.049	
FOXRED1	-0.395	-1.327	-1.487	
C4orf14	-0.339	-1.114	-1.261	Down
BAAT	-1.224	-4.26	-4.789	
TBXAS1	-0.308	-1.195	-1.335	
SYT3	-0.351	-1.552	-1.71	
CLDN2	-0.945	-3.945	-4.266	
BCKDHB	-0.421	-1.784	-1.931	
SELENBP1	-0.532	-2.123	-2.294	
RARRES3	-0.249	-1.057	-1.136	
TMEM80	-0.368	-1.606	-1.698	
FDPS	-0.28	-1.1	-1.182	
CCDC153	-0.244	-0.952	-1.006	
UGT1A6	-0.26	-0.957	-1.022	
MYO3B	-0.258	-1.03	-1.215	
GCNT3	-0.255	-1.029	-1.206	
THRA	-0.448	-1.697	-2.014	
SYT12	-0.665	-2.575	-3.12	Down
PAFAH1B3	-0.32	-1.241	-1.472	
ODF3L1	-0.239	-0.918	-1.104	Down
MAP4K2	-0.513	-1.985	-2.382	Down
RHPN1	-0.441	-1.605	-1.868	
LOC283710	-0.317	-1.524	-1.748	
HSD17B4	-0.306	-1.376	-1.627	
SGK2	-0.259	-1.59	-1.988	
FCGBP	-0.333	-1.859	-2.323	
PEX11B	-0.161	-0.934	-1.201	
LOC730011	-0.131	-0.887	-1.112	

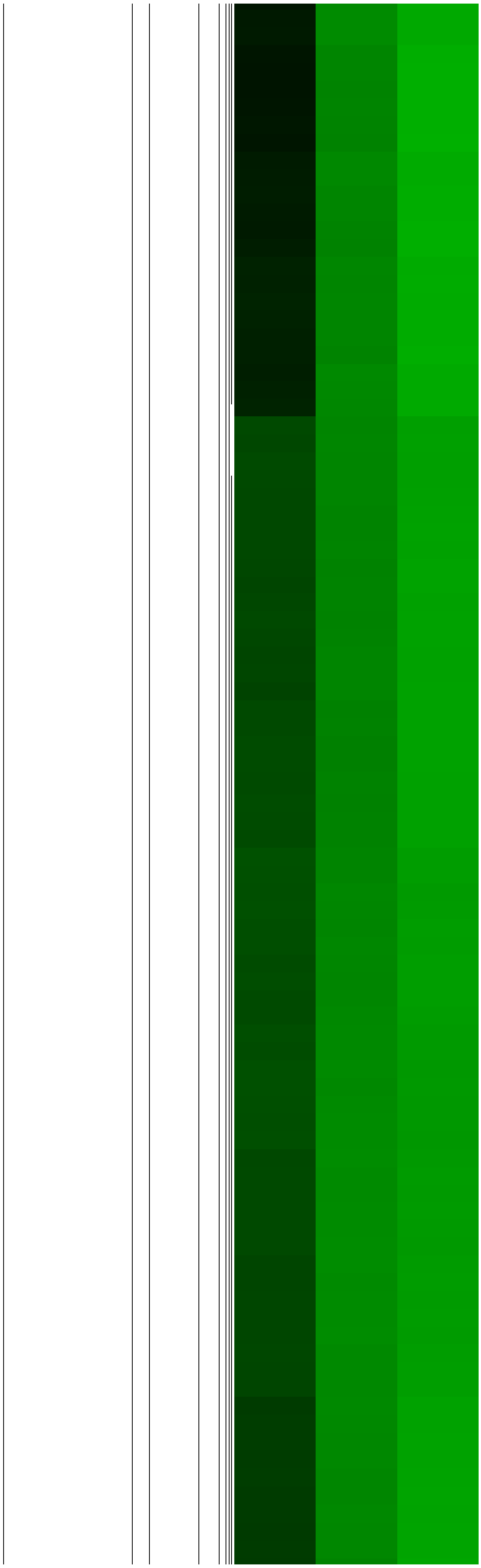

SPDEF	-0.562	-3.034	-3.696	
SLC38A10	-0.166	-0.887	-1.073	
TMBIM4	-0.16	-1.005	-1.314	
KRT19	-0.218	-1.436	-1.888	
CDH1	-0.354	-2.243	-2.991	
MBD4	-0.175	-1.052	-1.396	
BCAS1	-0.512	-2.812	-3.756	Down
RTDR1	-0.176	-1.05	-1.419	Down
MAOA	-0.365	-1.826	-2.302	
EME1	-0.381	-1.805	-2.272	
PRY2	-0.258	-1.186	-1.536	Down
PGD	-0.288	-1.41	-1.841	Down
B3GALT4	-0.542	-2.672	-3.542	Down
BANK1	-0.259	-1.147	-1.544	Down
PCDHA4	-0.307	-1.189	-1.519	Down
LOC731139	-0.318	-1.253	-1.617	Down
GABRA5	-0.318	-1.199	-1.531	Down
AP2M1	-0.248	-0.959	-1.243	Down
TMC5	-0.415	-1.678	-2.17	Down
MEGF9	-0.253	-1.038	-1.37	Down
PTPN6	-0.43	-1.925	-2.38	
NOVA1	-0.337	-1.395	-1.733	Down
VAV3	-0.919	-3.484	-4.363	Down
SLCO3A1	-0.634	-1.162	-1.387	Down
KCNMB4	-1.077	-2.008	-2.398	Down
DSP	-0.502	-0.898	-1.077	Down
DIS3L	-0.596	-1.079	-1.295	Down
C8orf51	-0.949	-1.759	-2.109	Down
C11orf80	-0.462	-0.842	-1.032	Down
BCL2L11	-0.558	-1.016	-1.255	Down
ARHGAP1	-0.648	-1.182	-1.444	Down
UROS	-0.89	-1.62	-2.027	Down
LOC80154	-0.669	-1.26	-1.573	Down
MAP2	-0.883	-1.624	-1.994	Down
LOC100128567	-0.499	-0.886	-1.104	Down
ZC4H2	-1.38	-2.541	-3.122	Down
TCF19	-0.717	-1.369	-1.659	Down
PET112L	-0.633	-1.203	-1.461	Down
ECHDC2	-0.869	-1.713	-2.078	Down
TTC39C	-0.566	-0.996	-1.251	Down
S1PR5	-0.673	-1.195	-1.489	Down
KIAA1737	-0.486	-0.829	-1.049	Down
IL22RA1	-1.215	-2.067	-2.621	Down
FLJ11235	-1.024	-1.789	-2.215	Down
BRE	-0.623	-1.052	-1.303	Down
EVC	-0.489	-0.844	-1.045	Down
ASRGL1	-0.686	-1.2	-1.489	Down
SOX4	-0.592	-0.964	-1.145	Down
DACT2	-2.479	-4.087	-4.873	Down
PRICKLE2	-0.815	-1.386	-1.583	Down
CLDN3	-1.117	-1.854	-2.148	Down
LRRC3	-0.499	-0.854	-1.004	Down
C6orf153	-1.056	-1.821	-2.104	Down
KRT222	-0.825	-1.472	-1.737	Down
APEX2	-0.623	-1.076	-1.281	Down
SH3GLB2	-0.475	-0.863	-1.013	Down
STOX2	-1.282	-2.36	-2.697	Down
WNT6	-0.996	-1.755	-1.965	Down
KIF26A	-1.054	-1.895	-2.124	Down
MUS81	-1.041	-1.8	-1.98	Down
AMDHD2	-1.119	-1.905	-2.146	Down
VPS33A	-0.588	-1.015	-1.121	Down
HSPA2	-1.546	-2.76	-3.001	Down
CDKN2A	-0.53	-0.925	-1.011	Down
RAB15	-1.03	-1.986	-2.173	Down
NDUFAF3	-0.5	-0.942	-1.051	Down
LRRC23	-0.536	-1.008	-1.124	Down
AHSA1	-0.652	-1.237	-1.379	Down
TRMT2A	-0.534	-1.018	-1.132	Down
RAD51L3	-0.989	-1.893	-2.059	Down
MEX3B	-0.814	-1.65	-1.835	Down
LOC729421	-0.821	-1.64	-1.853	Down
FDPSL2A	-0.597	-1.175	-1.309	Down
AFAP1L2	-0.62	-1.234	-1.383	Down
SNORA12	-2.241	-4.448	-4.916	Down
MSH6	-0.568	-1.095	-1.258	Down
HMGB3	-0.547	-1.062	-1.225	Down
SALL2	-2.084	-4.102	-4.763	Down
MREG	-0.75	-1.717	-2.036	Down
GCLC	-0.599	-1.327	-1.577	Down
ZNF643	-0.621	-1.371	-1.655	Down
ZNF575	-1.291	-2.889	-3.447	Down
SEPX1	-0.739	-1.61	-1.952	Down
RIMKLA	-1.188	-2.704	-3.306	Down
PCYOX1L	-0.837	-1.906	-2.282	Down
LOC100129958	-0.416	-0.969	-1.178	Down
CALCOCO1	-0.486	-1.112	-1.347	Down
GSR	-0.393	-0.88	-1.113	Down

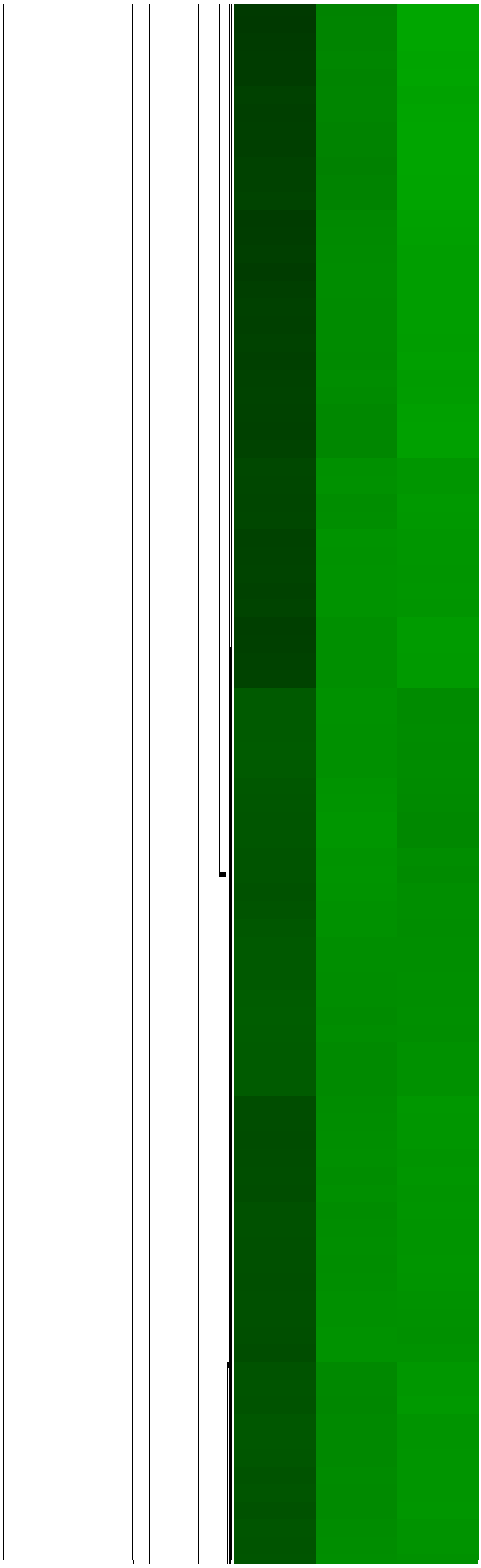

CAST	-0.452	-1.044	-1.309	Down
ZNF32	-1.075	-2.379	-2.98	Down
MXD4	-0.644	-1.417	-1.738	Down
MRPS27	-0.396	-0.866	-1.08	Down
RNF31	-0.567	-1.162	-1.414	Down
FBXO4	-1.076	-2.239	-2.759	Down
C11orf93	-0.687	-1.403	-1.776	Down
ARMC9	-0.629	-1.313	-1.648	Down
LOC100128191	-0.431	-0.837	-1.069	Down
IPO4	-0.427	-0.844	-1.057	Down
ZNF74	-0.665	-1.281	-1.604	Down
TNNT1	-0.475	-1.083	-1.268	Down
SFRS13B	-0.408	-0.911	-1.055	Down
PDK3	-0.477	-1.06	-1.203	Down
MID1	-0.622	-1.44	-1.626	Down
SAPS2	-0.441	-0.97	-1.094	Down
MAPK12	-0.444	-0.944	-1.075	Down
CPLX2	-0.617	-1.323	-1.512	Down
CACNB3	-0.695	-1.466	-1.652	Down
ZDHHC6	-0.946	-2.043	-2.339	Down
LOC375190	-0.646	-1.375	-1.524	Down
CYB5R2	-0.68	-1.411	-1.594	Down
WWOX	-0.706	-1.458	-1.716	Down
ALAD	-0.689	-1.44	-1.701	Down
ZNF879	-0.437	-0.876	-1.039	Down
SNRNP25	-0.942	-1.861	-1.941	Down
BATF3	-0.552	-1.128	-1.168	Down
E2F2	-0.655	-1.322	-1.435	Down
AQP11	-1.034	-2.075	-2.213	Down
INTS3	-1.033	-2.276	-2.321	Down
GATM	-0.874	-1.89	-1.943	Down
CHRNB2	-0.844	-1.823	-1.846	Down
A2LD1	-1.131	-2.492	-2.553	Down
MAST3	-0.708	-1.522	-1.536	Down
RPUSD4	-0.448	-1.018	-1.099	Down
C10orf11	-0.541	-1.197	-1.295	Down
STARD7	-0.777	-1.681	-1.804	Down
B9D1	-0.897	-1.882	-2.036	Down
PRAME	-0.667	-1.073	-1.025	
MCM4	-0.922	-1.493	-1.423	
LOC286161	-0.852	-1.368	-1.325	
CLYBL	-0.82	-1.293	-1.244	
ZG16B	-1.034	-1.648	-1.599	
SAPS1	-0.636	-1.067	-1.007	
LOC100128838	-0.902	-1.54	-1.442	
PEX10	-0.568	-1.005	-0.912	
CA11	-1.023	-1.773	-1.6	
MCM6	-0.657	-1.154	-1.105	
HSP90AB5P	-0.971	-1.711	-1.605	
TMSL1	-0.678	-1.212	-1.173	
LOC729409	-0.622	-1.065	-1.047	
ATAD4	-1.413	-2.35	-2.288	
SCNN1A	-1.331	-2.136	-2.135	
NCRNA00094	-1.584	-2.541	-2.541	
FBXO15	-1.752	-2.753	-2.803	Down
GPR20	-0.989	-1.507	-1.519	
ATP6AP1L	-0.741	-1.108	-1.137	Down
SLC2A6	-0.817	-1.249	-1.264	Down
FOXM1	-0.641	-0.967	-1.013	Down
DISP1	-0.855	-1.3	-1.359	Down
GSTO2	-0.66	-0.997	-1.048	Down
LOC643783	-0.623	-1.136	-1.22	Down
CCDC159	-0.844	-1.535	-1.626	Down
MMAB	-0.977	-1.814	-1.912	Down
BCMO1	-0.561	-1.047	-1.081	Down
ZNF177	-0.528	-0.956	-1.013	Down
SNX22	-0.912	-1.687	-1.753	Down
ARHGEF9	-1.209	-2.068	-2.206	Down
ADCY7	-0.57	-0.99	-1.043	Down
SLC9A3R1	-1.037	-1.827	-1.913	Down
MYL5	-0.844	-1.489	-1.565	Down
MGC23284	-1.061	-1.903	-1.989	Down
WBSCR27	-0.564	-1.015	-1.028	Down
RNF213	-1.163	-2.098	-2.113	Down
PGAP3	-0.733	-1.356	-1.34	Down
C18orf10	-0.644	-1.212	-1.211	Down
CCDC103	-0.902	-1.479	-1.637	Down
BAMBI	-0.878	-1.417	-1.576	Down
TIMM13	-0.797	-1.29	-1.465	Down
SCARNA12	-1.01	-1.591	-1.719	Down
PLCG1	-0.863	-1.351	-1.462	Down
SHISA4	-0.632	-1.009	-1.091	Down
KLHDC4	-0.636	-1.062	-1.145	Down
C15orf38	-0.686	-1.12	-1.202	Down
IKBKAP	-0.554	-0.927	-1.007	Down
ZNF287	-0.735	-1.208	-1.267	Down
PARP10	-0.592	-0.985	-1.026	Down
TCF25	-0.589	-0.983	-1.004	Down

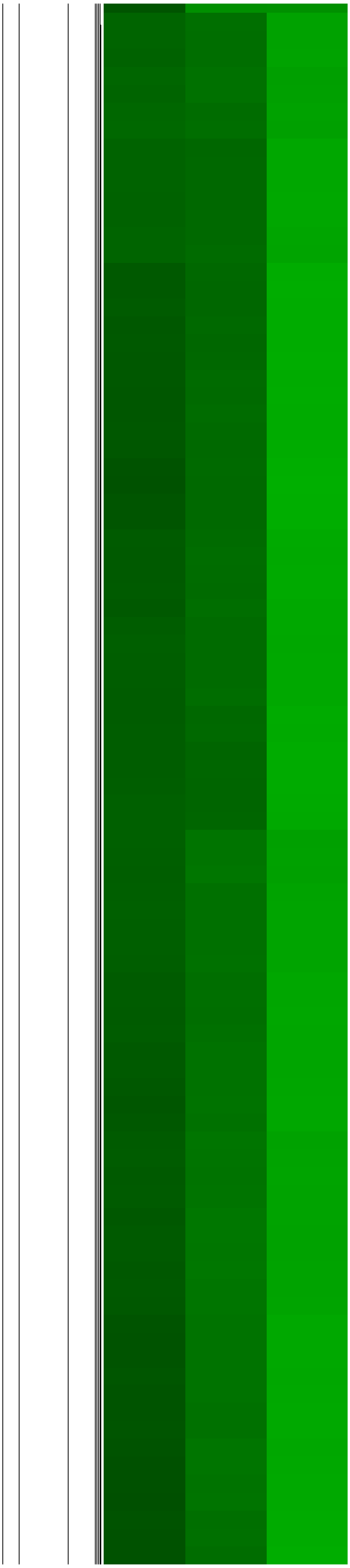

ARRB1	-1.564	-2.663	-2.684	Down
C9orf103	-0.988	-1.094	-1.591	Down
PRKDC	-0.677	-0.745	-1.096	Down
C7orf25	-1.669	-1.869	-2.771	Down
MSTO1	-1.657	-1.828	-2.576	Down
C16orf35	-1.173	-1.318	-1.867	Down
PSMC5	-0.902	-0.944	-1.419	Down
ODF3L2	-1.253	-1.328	-1.934	Down
SCARNA17	-1.296	-1.348	-2.182	Down
PRR20B	-1.07	-1.121	-1.798	Down
GSC	-1.334	-1.403	-2.253	Down
FXN	-0.9	-0.951	-1.535	Down
THNSL1	-2.055	-2.199	-3.479	Down
RASIP1	-1.177	-1.239	-1.936	Down
C9orf38	-0.698	-0.748	-1.138	Down
KRTAP1-3	-0.635	-0.737	-1.225	Down
IMPDH1	-0.516	-0.597	-1.004	Down
SPATA4	-0.543	-0.613	-1.025	Down
FBXL16	-0.656	-0.782	-1.271	Down
C1orf93	-1.158	-1.338	-2.225	Down
SPOCD1	-0.589	-0.699	-1.153	Down
HECW1	-0.612	-0.74	-1.182	Down
CEBPA	-0.883	-1.07	-1.729	Down
PARS2	-0.571	-0.707	-1.115	Down
MYBBP1A	-0.865	-1.045	-1.685	Down
LOC647086	-0.698	-0.843	-1.379	Down
ZNF252	-0.757	-0.961	-1.579	Down
SPRYD4	-0.591	-0.757	-1.256	Down
LOC222070	-0.996	-1.231	-2.045	Down
ANKRD16	-1.314	-1.616	-2.669	Down
POLR3H	-0.95	-1.116	-1.774	Down
LOC145474	-0.766	-0.924	-1.436	Down
KILLIN	-1.074	-1.287	-2.022	Down
FAM114A2	-0.658	-0.776	-1.228	Down
ZNF425	-0.99	-1.218	-1.859	Down
LOC257152	-0.566	-0.652	-1.017	Down
C21orf58	-0.663	-0.748	-1.171	Down
LOC401022	-0.716	-0.81	-1.268	Down
SHARPIN	-0.766	-0.878	-1.376	Down
CPT2	-0.717	-0.842	-1.297	Down
SCARNA16	-1.095	-1.247	-2.037	Down
LOC100147773	-1.286	-1.451	-2.343	Down
MRPL46	-0.825	-0.901	-1.524	Down
KCNH3	-0.552	-0.602	-1.012	Down
RWDD2B	-1.203	-1.299	-2.189	Down
RNASEL	-1.457	-1.532	-2.56	Down
EXOSC3	-0.702	-0.747	-1.233	Down
IFT52	-0.69	-0.836	-1.15	Down
ALG1	-0.66	-0.803	-1.117	Down
PYCRL	-0.854	-1.069	-1.442	Down
DQX1	-0.842	-0.996	-1.446	Down
C9orf125	-1.198	-1.392	-2.039	Down
THOC3	-0.682	-0.805	-1.173	Down
PARD6G	-1.254	-1.474	-2.162	Down
LOC644613	-1.05	-1.251	-1.82	Down
POLE2	-0.625	-0.752	-1.138	Down
ADRA1D	-1.46	-1.756	-2.629	Down
TMEM121	-1.059	-1.279	-1.932	Down
RPA1	-0.586	-0.71	-1.05	Down
FBXO16	-2.264	-2.89	-4.191	Down
ATP13A2	-1.339	-1.688	-2.448	Down
TRIP13	-0.739	-0.961	-1.394	Down
FANCG	-1.111	-1.473	-2.134	Down
CACYBP	-0.869	-1.12	-1.644	Down
ZNF114	-0.978	-1.267	-1.75	Down
VPS33B	-0.592	-0.747	-1.049	Down
PSMD4	-0.554	-0.711	-1.01	Down
FLJ40504	-0.679	-0.862	-1.206	Down
LOC729213	-0.586	-0.793	-1.083	Down
C21orf70	-0.636	-0.844	-1.143	Down
BRCC3	-0.604	-0.791	-1.086	Down
BAT4	-0.964	-1.291	-1.771	Down
ZNF792	-0.857	-1.123	-1.567	Down
GPRIN2	-2.57	-3.419	-4.798	Down
URM1	-0.548	-0.753	-1.093	Down
PAAF1	-0.895	-1.238	-1.805	Down
SETMAR	-0.595	-0.814	-1.187	Down
LOC730236	-1.423	-1.908	-2.756	Down
WDR31	-1.016	-1.394	-2.028	Down
FIBP	-0.665	-0.887	-1.327	Down
FAM78B	-0.526	-0.698	-1.041	Down
TEX19	-1.175	-1.657	-2.356	Down
LOC284014	-0.505	-0.731	-1.044	Down
LOC152286	-0.554	-0.784	-1.147	Down
FHOD3	-1.676	-2.449	-3.548	Down
PRR5	-0.667	-0.89	-1.364	Down
LOC401480	-0.529	-0.721	-1.099	Down
SLC35E3	-0.764	-1.019	-1.611	Down

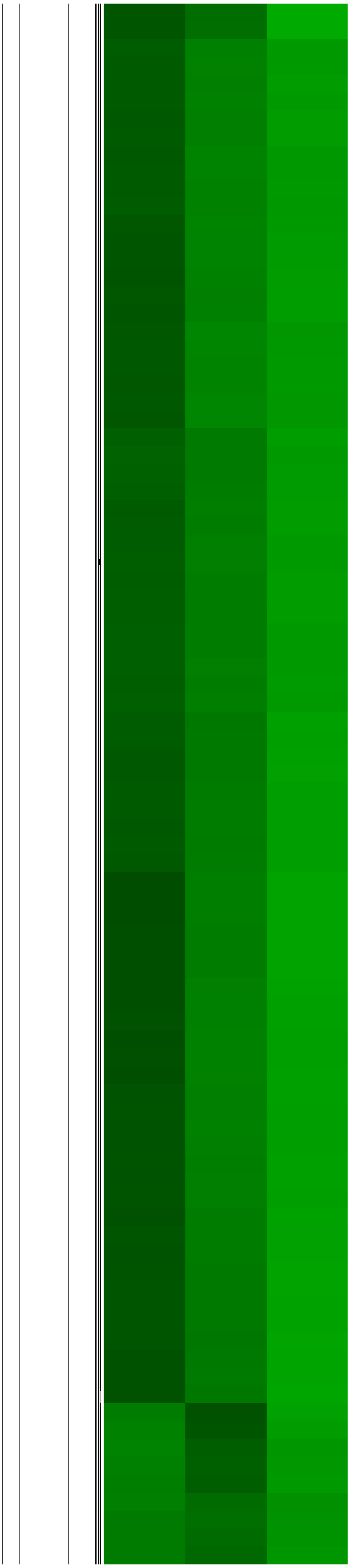

MYPOP	-1.101	-1.391	-2.185	Down
FAM21C	-0.532	-0.687	-1.058	Down
ZNF703	-0.71	-0.992	-1.192	Down
PGM2	-0.641	-0.908	-1.081	Down
FAM192A	-0.723	-1.02	-1.253	Down
AVIL	-0.707	-1.014	-1.217	Down
ZNF397	-0.601	-0.858	-1.053	Down
RTN4RL1	-0.797	-1.114	-1.368	Down
MYOM2	-1.048	-1.541	-1.781	Down
GTF2A1	-0.643	-0.922	-1.067	Down
ANXA2P3	-0.752	-1.09	-1.277	Down
SEPT5	-0.667	-0.942	-1.104	Down
PGAP2	-0.84	-1.276	-1.484	Down
LRRIQ3	-0.75	-1.152	-1.357	Down
ELAC1	-2.094	-3.228	-3.823	Down
ANXA2P1	-0.739	-1.138	-1.361	Down
KBTBD7	-0.818	-1.198	-1.473	Down
ARHGEF10L	-0.938	-1.411	-1.729	Down
WDR51A	-0.666	-1.02	-1.155	Down
TMEM86B	-0.893	-1.344	-1.533	Down
HSPA8	-2.473	-3.682	-4.274	Down
EXOC4	-0.907	-1.37	-1.597	Down
TET1	-0.869	-1.325	-1.488	Down
RGS9BP	-0.588	-0.909	-1.019	Down
SRD5A1	-1.148	-1.469	-1.894	Down
LOC645332	-0.993	-1.241	-1.564	Down
MMACHC	-1.122	-1.414	-1.788	Down
LOC642808	-0.697	-0.901	-1.13	Down
ORC3L	-0.724	-1.004	-1.24	Down
MYB	-0.963	-1.316	-1.635	Down
MGAT3	-1.837	-2.549	-3.095	Down
C9orf98	-1.199	-1.642	-1.985	Down
KRBA1	-1.005	-1.372	-1.701	Down
GPS2	-1.147	-1.534	-1.914	Down
IRF2BP2	-0.787	-1.047	-1.309	Down
CDC123	-0.672	-0.883	-1.087	Down
HOXC6	-1.079	-1.419	-1.741	Down
ANO7	-0.663	-0.885	-1.062	Down
NSDHL	-0.965	-1.292	-1.594	Down
LOH12CR2	-0.758	-1.006	-1.221	Down
C17orf100	-0.989	-1.29	-1.711	Down
B4GALNT1	-2.398	-3.137	-4.132	Down
PRODH	-0.834	-1.127	-1.491	Down
APBA2	-1.414	-1.94	-2.565	Down
FAM59A	-1.035	-1.397	-1.798	Down
DDX28	-0.927	-1.28	-1.631	Down
SEC14L4	-0.79	-1.116	-1.431	Down
SURF1	-0.694	-0.947	-1.216	Down
SLC3A1	-1.087	-1.519	-1.914	Down
LOC159110	-0.545	-0.885	-1.145	Down
LOC100127925	-0.52	-0.856	-1.103	Down
CCDC125	-0.713	-1.157	-1.498	Down
APEX1	-0.758	-1.196	-1.559	Down
OLFM1	-0.516	-0.821	-1.066	Down
MRPL20	-0.615	-0.985	-1.283	Down
LOC442249	-0.504	-0.82	-1.04	Down
GMPS	-0.563	-0.909	-1.133	Down
RNFT2	-0.741	-1.171	-1.462	Down
CDCA7L	-0.821	-1.358	-1.66	Down
B4GALT7	-0.749	-1.214	-1.491	Down
MTHFD1	-0.665	-1.098	-1.361	Down
C16orf62	-0.769	-1.18	-1.482	Down
ACD	-0.759	-1.153	-1.44	Down
PNPO	-1.101	-1.713	-2.095	Down
PAOX	-0.61	-0.935	-1.167	Down
PALM	-0.773	-1.155	-1.468	Down
MOCS1	-0.791	-1.207	-1.519	Down
GNG2	-0.869	-1.302	-1.633	Down
ZNF324B	-0.901	-1.357	-1.767	Down
TH1L	-0.531	-0.776	-1.004	Down
PLA2G2E	-0.529	-0.795	-1.022	Down
MAPKAPK3	-0.849	-1.213	-1.636	Down
ANKAR	-0.802	-1.147	-1.534	Down
TP53I3	-0.806	-1.144	-1.53	Down
PRMT7	-1.486	-2.087	-2.791	Down
GYS1	-0.862	-1.199	-1.665	Down
MCRS1	-0.519	-0.763	-1.033	Down
G6PD	-0.509	-0.766	-1.023	Down
SNORD116-19	-1.466	-2.166	-2.999	Down
YPEL4	-0.943	-0.618	-1.209	
COMMD7	-0.949	-0.629	-1.138	
RNF222	-1.079	-0.763	-1.232	
C1orf175	-1.383	-1.034	-1.628	Down
ADPRH	-1.326	-0.989	-1.627	Down
KPRP	-1.136	-0.969	-1.288	Down
MYBL2	-0.998	-0.897	-1.193	Down
KCTD14	-1.313	-1.139	-1.565	Down
RCOR2	-1.475	-1.252	-1.796	Down

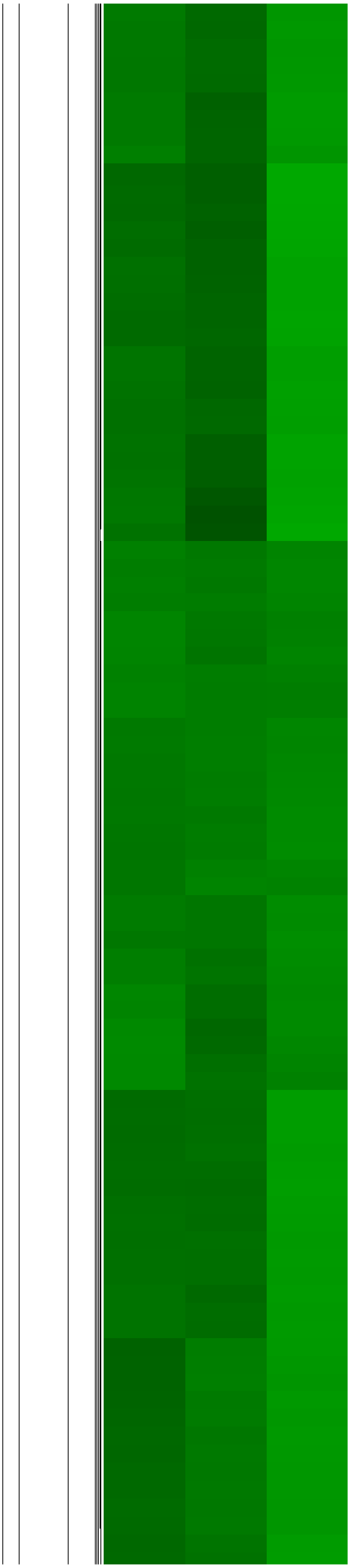

LOC100129540	-0.827	-0.708	-1.013	Down
WDR77	-1.117	-0.97	-1.42	Down
SNORA74A	-0.97	-0.865	-1.215	Down
SNORA62	-1.611	-1.455	-2.055	Down
LRRN3	-1.185	-1.054	-1.509	Down
TSC2	-0.799	-0.632	-1.011	Down
LOC400558	-1.485	-1.222	-1.872	Down
LOC100130520	-1.067	-0.883	-1.344	Down
HSPA5	-1.183	-0.938	-1.387	Down
FSCN1	-0.707	-0.646	-1.138	Down
EFNA4	-0.938	-0.847	-1.483	Down
SCARNA2	-1.04	-0.976	-1.651	Down
LOC400958	-0.664	-0.584	-1.013	Down
LOC100129235	-1.383	-1.261	-2.12	Down
RNF32	-0.702	-0.614	-1.016	Down
GATA5	-1.377	-1.232	-2.019	Down
CEP68	-0.919	-0.851	-1.369	Down
PPP1R3G	-0.739	-0.706	-1.141	Down
NADSYN1	-0.906	-0.883	-1.4	Down
LOC286063	-0.789	-0.678	-1.075	Down
CYGB	-1.006	-0.864	-1.369	Down
C11orf60	-0.933	-0.81	-1.304	Down
LOC100129534	-0.801	-0.742	-1.134	Down
DIRAS1	-0.93	-0.872	-1.311	Down
USP14	-1.061	-0.883	-1.512	Down
EGLN3	-1.299	-1.095	-1.876	Down
SYNGR4	-0.728	-0.593	-1.012	Down
PRKAR1B	-1.111	-0.812	-1.516	Down
LOC80054	-0.748	-0.51	-1.031	Down
SURF2	-0.73	-0.536	-1.076	Down
OR8B4	-1.313	-1.231	-1.346	
COASY	-1.126	-1.096	-1.207	
ACTR10	-1.054	-0.992	-1.11	
LCE4A	-1.526	-1.516	-1.61	
LOC100132330	-1.461	-1.321	-1.409	
BOK	-1.464	-1.314	-1.425	
DLGAP1	-1.217	-1.077	-1.22	
SP110	-1.327	-1.286	-1.32	
LOC100287919	-1.232	-1.169	-1.175	
CLEC4M	-1.165	-1.113	-1.123	
OR2M7	-1.058	-1.094	-1.17	
LOC644189	-2.25	-2.321	-2.46	
CYS1	-1.267	-1.326	-1.42	
CRYBG3	-0.923	-0.956	-1.049	
RANBP17	-1.175	-1.234	-1.351	
LOC100127920	-1.569	-1.585	-1.818	
PFAS	-1.16	-1.215	-1.362	
LOC648987	-0.997	-1.044	-1.19	
PCDHGB4	-0.957	-1.043	-1.079	
C9orf24	-1.061	-1.188	-1.169	
CDK5R2	-0.983	-0.941	-1.116	
CCT5	-1.246	-1.201	-1.408	
LOC100130116	-1.47	-1.453	-1.752	
LOC100132077	-1.029	-0.924	-1.141	
FAM120AOS	-1.398	-1.28	-1.533	
ZFHX4	-1.366	-1.111	-1.391	
RRP7A	-1.184	-0.978	-1.231	
OR10A6	-1.181	-0.892	-1.197	
LRRN2	-1.057	-0.805	-1.033	
HTR7	-1.827	-1.475	-1.751	
GLUL	-1.009	-0.841	-0.955	
MMP17	-0.74	-0.771	-1.084	Down
FAM134B	-1.976	-2.006	-2.872	Down
WDR4	-1.462	-1.521	-2.138	Down
PRINS	-0.926	-0.966	-1.329	Down
PNMA5	-1.043	-1.036	-1.495	Down
IL17RD	-1.6	-1.587	-2.337	Down
LOC728129	-0.841	-0.831	-1.182	Down
CDH15	-0.855	-0.827	-1.184	Down
SFTPA2	-0.733	-0.743	-1.012	Down
C13orf38	-0.777	-0.772	-1.066	Down
ADSL	-1.241	-1.223	-1.688	Down
PNPLA4	-0.848	-0.773	-1.143	Down
MX1	-0.763	-0.725	-1.016	Down
LOC648149	-1.394	-1.309	-1.868	Down
FBXL8	-0.657	-0.84	-1.027	Down
C19orf54	-1.4	-1.774	-2.135	Down
MPP2	-1.356	-1.736	-2.038	Down
LOC401431	-1.377	-1.675	-2.108	Down
DNAJC30	-1.215	-1.467	-1.797	Down
DBNL	-1.658	-1.904	-2.437	Down
BTBD6	-1.514	-1.773	-2.213	Down
LOC728940	-1.698	-1.95	-2.451	Down
LOC100128108	-0.938	-1.084	-1.353	Down
SBK1	-2.264	-2.544	-3.169	Down
LOC730091	-0.777	-0.883	-1.091	Down
JMJD4	-1.823	-2.032	-2.722	Down
ACOT4	-2.177	-2.354	-3.217	Down

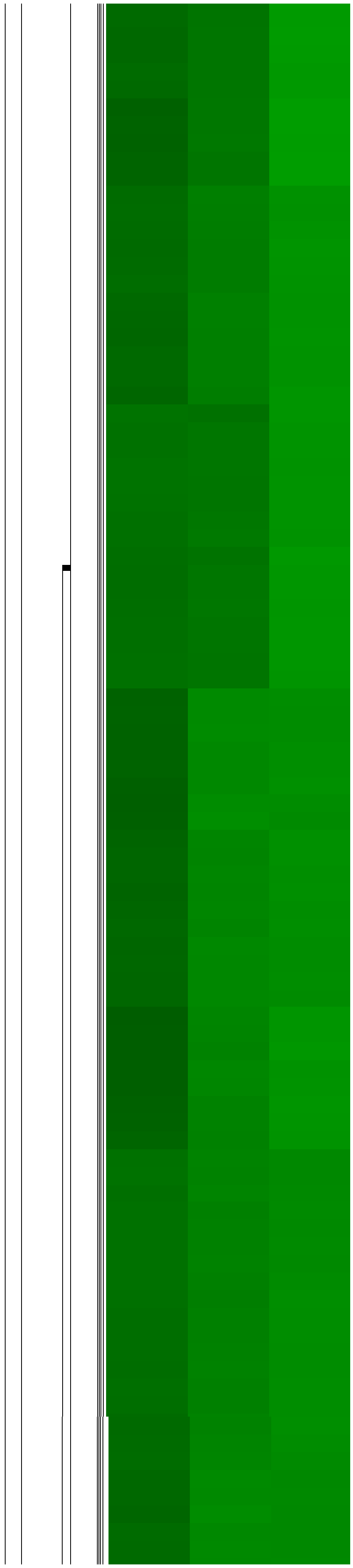

FAM176B	-0.753	-0.828	-1.1	Down
CD79A	-0.861	-0.963	-1.274	Down
ARMCX1	-0.72	-0.806	-1.061	Down
ZNF19	-1.699	-1.872	-2.428	Down
FASN	-0.965	-1.09	-1.407	Down
LOC440905	-0.773	-0.947	-1.247	Down
FTSJ3	-0.664	-0.802	-1.054	Down
ACLY	-0.812	-0.993	-1.288	Down
SNORA23	-0.942	-1.1	-1.468	Down
LOC440149	-0.865	-1.011	-1.357	Down
LOC100132839	-0.991	-1.172	-1.337	Down
KHK	-1.978	-2.299	-2.637	Down
ZSCAN16	-0.878	-1.022	-1.198	Down
RAB11FIP4	-1.378	-1.615	-1.92	Down
C2orf81	-1.601	-1.853	-2.192	Down
HOMEZ	-1.869	-2.114	-2.489	Down
DNAJC14	-0.78	-0.949	-1.078	Down
CCDC87	-0.85	-1.051	-1.203	Down
LOC649264	-0.815	-1.011	-1.171	Down
TTC12	-1.743	-2.093	-2.408	Down
LOC100131391	-1.127	-1.363	-1.57	Down
NUDT9P1	-1.083	-1.325	-1.576	Down
ARHGAP22	-1.644	-1.618	-2.133	Down
DBN1	-1.534	-1.595	-1.985	Down
CCDC102A	-0.908	-0.949	-1.181	Down
SPTBN2	-0.987	-1.013	-1.254	Down
NOL12	-1.34	-1.361	-1.71	Down
LOC339929	-1.718	-1.766	-2.208	Down
LOC100132832	-0.891	-0.951	-1.172	Down
DENND2D	-2.275	-2.456	-2.96	Down
LOC729680	-2.431	-2.539	-3.345	Down
ZC3H4	-1.148	-1.249	-1.58	Down
SRCRB4D	-0.95	-1.024	-1.3	Down
PSMG1	-0.816	-0.885	-1.102	Down
KLHL35	-0.789	-0.836	-1.065	Down
COL10A1	-1.327	-1.39	-1.782	Down
C21orf122	-1.174	-1.222	-1.572	Down
STOX1	-0.912	-0.95	-1.198	Down
NOC4L	-0.832	-1.17	-1.201	
ARL4D	-0.991	-1.387	-1.408	
SFXN2	-1.047	-1.488	-1.503	
LOC388152	-0.726	-1.005	-1.05	
GPRIN1	-1.073	-1.475	-1.541	
RIMS3	-0.716	-1.019	-1.072	Down
SCNM1	-0.939	-1.377	-1.379	
AXIN2	-1.661	-2.46	-2.401	
FAM66C	-0.858	-1.137	-1.233	Down
CFB	-1.729	-2.246	-2.445	Down
TLR1	-1.316	-1.736	-1.832	
RHBDL3	-1.213	-1.615	-1.734	Down
PFN4	-1.392	-1.819	-1.913	
HECTD3	-1.09	-1.392	-1.494	
PLCG2	-1.042	-1.378	-1.418	
LOC285178	-2.006	-2.617	-2.714	
LOC283674	-0.913	-1.209	-1.26	
BHLHB9	-0.76	-1.007	-1.028	
TBC1D9B	-0.842	-1.207	-1.347	Down
STARD5	-0.931	-1.302	-1.473	Down
LHX2	-1.394	-1.924	-2.235	Down
FBXW8	-0.983	-1.399	-1.511	Down
C5orf55	-1.141	-1.603	-1.76	Down
DNMT3B	-1.027	-1.374	-1.576	Down
BDH2	-0.88	-1.171	-1.324	Down
THOP1	-0.753	-0.964	-1.096	Down
PKN3	-1.113	-1.293	-1.343	
LOC400236	-1.788	-2.036	-2.129	
C21orf67	-1.005	-1.196	-1.247	
LOC100131552	-0.928	-1.052	-1.136	
GPRASP2	-1.719	-1.966	-2.085	
GPR120	-0.945	-1.08	-1.161	
EAF2	-1.222	-1.404	-1.48	
PCDHGA2	-1.697	-1.92	-2.08	
GJD3	-0.956	-1.076	-1.211	
ZNF70	-1.393	-1.616	-1.776	
KIAA1908	-2.315	-2.684	-2.957	
ZNRF1	-1.844	-2.152	-2.331	
HSPA1A	-2.339	-2.788	-2.993	
GJB2	-0.893	-1.024	-1.129	
AIFM3	-0.816	-0.951	-1.046	
LOC100144603	-1.288	-1.569	-1.692	
CCIN	-0.807	-0.992	-1.056	
LOC285593	-0.857	-1.048	-1.14	
MVK	-1.229	-1.524	-1.606	
LOC100128916	-0.844	-1.035	-1.078	
ZFP41	-1.566	-2.067	-2.031	
ZBED2	-0.947	-1.248	-1.241	
VPS52	-1.211	-1.66	-1.609	
PRPF6	-0.912	-1.156	-1.159	

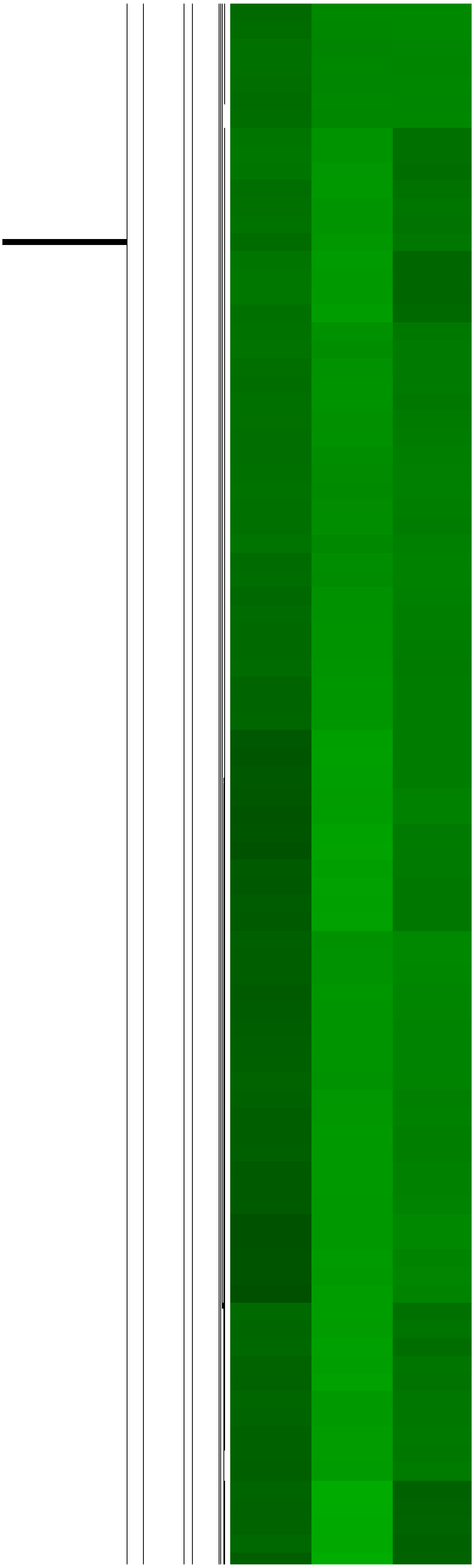

DNAH11	-1.074	-1.386	-1.382
CISH	-1.608	-2.067	-2.072
C20orf56	-1.642	-2.055	-2.08
TCP1	-1.113	-1.32	-1.332
CHAF1B	-1.169	-1.4	-1.38
RBM15B	-0.959	-1.165	-1.171
DMAP1	-0.825	-1.036	-1.022
CSRP2	-1	-1.231	-1.239
LRRC46	-1.159	-1.451	-1.119
FAM63A	-0.923	-1.128	-0.86
FAM66A	-0.986	-1.259	-0.908
LOC441722	-0.747	-1.03	-0.771
RMND1	-0.836	-1.116	-0.871
GDAP1	-1.486	-1.936	-1.496
CLIC3	-1.247	-1.746	-1.373
NLRP2	-0.952	-1.259	-0.83
SLC30A3	-1.132	-1.444	-0.971
ERP44	-0.834	-1.067	-0.709
PDCL2	-0.724	-1.009	-0.672
THEM4	-1.05	-1.331	-1.11
LOC650392	-0.977	-1.204	-1.029
STK36	-0.923	-1.217	-1.013
HSPA1B	-1.878	-2.526	-2.11
SLC4A3	-1.437	-1.889	-1.544
LOC100129936	-1.103	-1.422	-1.217
C21orf2	-0.829	-1.089	-0.934
LOC286121	-0.992	-1.278	-1.134
OR2A20P	-1.053	-1.296	-1.185
LOC144571	-1.582	-1.905	-1.775
PRNT	-1.174	-1.474	-1.32
GINS3	-1.06	-1.318	-1.169
TAAR9	-1.045	-1.223	-1.147
TMEM177	-1.185	-1.563	-1.427
GINS2	-1.097	-1.399	-1.304
PRKCQ	-1.623	-2.253	-1.989
STYXL1	-0.861	-1.17	-1.014
TELO2	-0.898	-1.257	-1.075
MOSC1	-1.175	-1.626	-1.371
VWA5B2	-1.376	-1.889	-1.572
GMPPB	-0.916	-1.362	-1.137
ALG12	-0.873	-1.308	-1.091
TMEM145	-0.92	-1.348	-1.124
HSP90AB1	-0.936	-1.693	-1.329
B3GNT4	-0.585	-1.09	-0.85
LGALS2	-1.04	-1.85	-1.473
SDF2L1	-0.627	-1.129	-0.93
KAZALD1	-0.989	-1.835	-1.514
HSP90AB2P	-0.932	-1.768	-1.326
GNG4	-0.664	-1.309	-0.985
SDR16C6	-0.62	-1.082	-0.831
OPLAH	-1.035	-1.857	-1.391
G6PC	-0.581	-1.011	-0.76
ANXA9	-1.137	-2.027	-1.515
PCDHB2	-1.052	-1.606	-1.515
LOC222699	-0.731	-1.146	-1.065
CAMK2N2	-1.044	-1.624	-1.499
CCDC3	-0.804	-1.316	-1.171
C15orf51	-0.678	-1.097	-0.982
TMEM63C	-1.277	-2.039	-1.809
SMARCC2	-0.651	-1.021	-0.892
OSGEP	-0.859	-1.32	-1.165
CHMP6	-0.987	-1.453	-1.316
TTC39A	-0.689	-1.073	-0.901
KHDC1	-1.136	-1.819	-1.549
RFC5	-0.742	-1.233	-1.009
NDRG2	-1.492	-2.406	-1.99
REEP2	-0.592	-1.014	-0.853
GALNT6	-1.072	-1.811	-1.519
LOC153811	-0.73	-1.221	-1.057
GANC	-1.048	-1.948	-1.737
GALNT10	-0.771	-1.427	-1.275
LOC100132857	-0.904	-1.653	-1.412
ACAD9	-0.864	-1.576	-1.375
DNA2	-0.606	-1.186	-1.004
SCAMP5	-1.934	-2.908	-2.082
E2F1	-0.765	-1.164	-0.864
LOC283332	-0.666	-1.03	-0.705
IKBKE	-0.924	-1.473	-1.099
GPR124	-1.172	-1.923	-1.374
hCG_2014417	-0.774	-1.147	-0.891
ARPM1	-0.805	-1.227	-0.958
SFRS2B	-0.768	-1.241	-0.959
LAYN	-1.12	-1.793	-1.382
PDX1	-0.722	-1.162	-0.918
TSPAN33	-0.585	-1.001	-0.568
ZNF189	-0.773	-1.336	-0.784
RRM2	-0.621	-1.054	-0.622
LOC100009676	-0.742	-1.201	-0.695

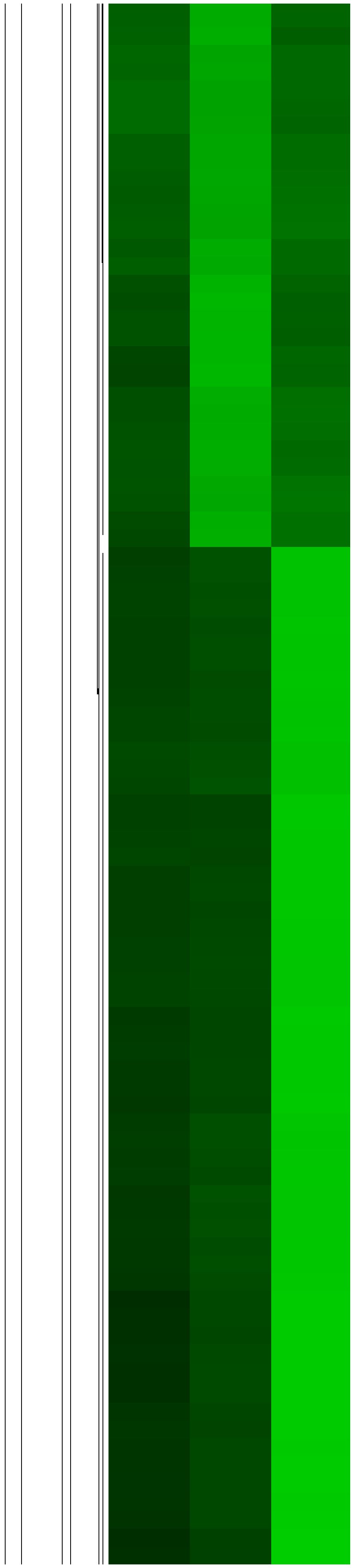

PRRT3	-1.522	-2.795	-1.606	
C1orf182	-1.087	-1.955	-1.143	
SHF	-0.699	-1.248	-0.674	
PRODH2	-0.84	-1.326	-0.848	
PCDHA3	-0.995	-1.632	-1.026	
POLA2	-0.928	-1.399	-0.901	
LOXL1	-0.78	-1.185	-0.755	
SF3A3	-0.682	-1.038	-0.645	
TMEM45A	-1.966	-3.433	-2.229	
NKAIN1	-0.976	-1.692	-1.108	
PRDM4	-0.57	-1.033	-0.681	
HNRNPD	-0.676	-1.25	-0.848	
TMEM98	-0.979	-1.749	-1.222	
LOC348751	-0.863	-1.497	-1.057	
EXO1	-0.801	-1.556	-0.955	
C12orf53	-0.775	-1.409	-0.872	
TMEM92	-0.533	-1.196	-0.664	
METTL7B	-1.017	-2.396	-1.244	
MAGIX	-0.856	-1.886	-1.009	
C19orf48	-0.456	-1.01	-0.527	
TM7SF2	-0.399	-1.026	-0.583	
ANGPTL2	-0.438	-1.177	-0.651	
UCP2	-1.867	-4.046	-2.604	
CBLN3	-0.679	-1.468	-0.968	
CCNE2	-0.552	-1.163	-0.744	
SLC44A2	-0.797	-1.651	-1.013	
EFNB3	-1.22	-2.54	-1.574	
FLJ39609	-0.521	-1.034	-0.709	
CACNA1H	-0.674	-1.384	-0.976	
FAM19A5	-0.58	-1.34	-0.871	
CES2	-0.487	-1.174	-0.756	
PLEKHF1	-0.401	-0.514	-1.225	Down
FAM127C	-0.918	-1.154	-2.751	Down
UNQ2963	-0.402	-0.475	-1.164	Down
CLDN10	-0.563	-0.674	-1.617	Down
LOC158863	-0.897	-1.052	-2.706	Down
INVS	-0.426	-0.506	-1.268	Down
ZBED3	-0.575	-0.693	-1.722	Down
RPL23AP32	-0.779	-0.893	-2.324	Down
METTL12	-0.849	-0.977	-2.476	Down
USP40	-0.862	-0.962	-2.414	Down
LRBA	-0.391	-0.423	-1.089	Down
LOC100288455	-0.549	-0.588	-1.417	Down
IMP3	-0.519	-0.584	-1.402	Down
C22orf40	-0.629	-0.74	-1.712	Down
STAG3L1	-0.345	-0.352	-1.045	Down
HOXA6	-0.907	-0.932	-2.786	Down
FLJ11710	-0.759	-0.786	-2.249	Down
C16orf53	-0.563	-0.562	-1.612	Down
ZNF205	-0.406	-0.459	-1.269	Down
ZFP62	-0.553	-0.64	-1.739	Down
LOC100132234	-0.412	-0.459	-1.301	Down
ACP6	-0.443	-0.504	-1.382	Down
SNORA59B	-0.478	-0.543	-1.464	Down
OSCAR	-0.372	-0.43	-1.14	Down
IFIT5	-0.38	-0.42	-1.133	Down
ANKS6	-0.868	-0.939	-2.558	Down
LOC284630	-0.503	-0.595	-1.718	Down
PSKH1	-0.455	-0.529	-1.518	Down
hCG_1645220	-0.436	-0.494	-1.423	Down
TMEM144	-0.469	-0.578	-1.594	Down
AGTPBP1	-0.461	-0.575	-1.586	Down
LOC728558	-0.45	-0.563	-1.606	Down
LOC283911	-0.319	-0.41	-1.031	Down
LOC151878	-0.51	-0.64	-1.605	Down
LOC642781	-0.493	-0.624	-1.599	Down
CCL7	-0.376	-0.46	-1.219	Down
FANCF	-1.307	-1.884	-4.566	Down
DERA	-0.453	-0.632	-1.569	Down
MEIS3	-0.547	-0.744	-1.833	Down
NEK9	-0.32	-0.425	-1.097	Down
LOC100133923	-0.57	-0.779	-1.972	Down
LOC100130711	-0.517	-0.707	-1.863	Down
LOC339803	-0.401	-0.632	-1.787	Down
IFIT2	-0.271	-0.405	-1.15	Down
TPCN2	-0.334	-0.484	-1.388	Down
LOC100289600	-0.407	-0.602	-1.66	Down
C17orf39	-0.287	-0.442	-1.203	Down
NUP37	-0.272	-0.428	-1.169	Down
F8A2	-0.539	-0.71	-2.041	Down
BRUNOL6	-0.271	-0.341	-1.006	Down
ZBTB37	-0.336	-0.444	-1.259	Down
SH3PXD2B	-0.723	-0.96	-2.727	Down
PIP4K2B	-0.301	-0.411	-1.166	Down
KLHL12	-0.266	-0.358	-1.007	Down
CCDC51	-0.491	-0.695	-1.932	Down
LOC728739	-0.436	-0.615	-1.941	Down
FLJ10213	-0.274	-0.374	-1.186	Down

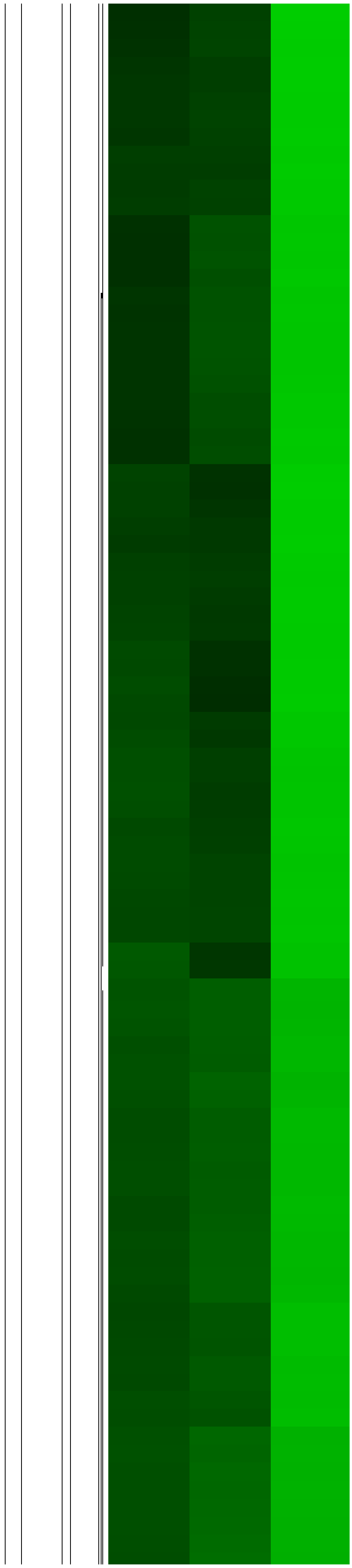

MAP2K5	-0.48	-0.658	-2.066	Down
LOC283070	-0.275	-0.379	-1.151	Down
LOC100128563	-0.596	-0.811	-2.42	Down
PITPNC1	-0.364	-0.426	-1.393	Down
PDCD2	-0.604	-0.684	-2.231	Down
C6orf122	-0.379	-0.453	-1.396	Down
ASB13	-0.32	-0.378	-1.138	Down
HAUS6	-0.412	-0.502	-1.553	Down
SLC30A6	-0.49	-0.495	-1.572	Down
RERE	-0.459	-0.465	-1.529	Down
LOC440525	-0.307	-0.346	-1.044	Down
GNAZ	-0.859	-0.919	-2.812	Down
ZDHHC4	-0.399	-0.673	-1.615	Down
SYNC	-0.298	-0.505	-1.24	Down
PDCD5	-0.263	-0.45	-1.078	Down
PRSS36	-0.534	-0.881	-2.211	Down
LOC100128430	-0.391	-0.604	-1.456	Down
HES6	-0.382	-0.615	-1.487	Down
NDUFB9	-0.349	-0.548	-1.305	Down
DTD1	-0.357	-0.578	-1.348	Down
XK	-0.433	-0.694	-1.634	Down
RNF144A	-0.366	-0.566	-1.369	Down
REEP4	-0.386	-0.571	-1.479	Down
BRMS1	-0.51	-0.771	-1.968	Down
NDRG3	-0.598	-0.911	-2.433	Down
FLJ25917	-0.469	-0.73	-1.896	Down
C17orf58	-0.357	-0.267	-1.076	Down
ATP6V1E2	-0.797	-0.608	-2.513	Down
MLYCD	-0.49	-0.4	-1.519	Down
RGL1	-0.82	-0.759	-2.669	Down
EID3	-0.413	-0.4	-1.416	Down
SYNJ2BP	-0.337	-0.319	-1.06	Down
LOC400987	-0.629	-0.595	-1.92	Down
PNMA2	-0.628	-0.573	-1.932	Down
WDSUB1	-0.405	-0.348	-1.223	Down
OIP5	-0.39	-0.336	-1.154	Down
NECAB2	-0.608	-0.401	-1.644	Down
FAM65A	-0.454	-0.306	-1.252	Down
VPS13D	-0.6	-0.365	-1.589	Down
MINA	-0.408	-0.256	-1.137	Down
SCARNA1	-0.379	-0.312	-1.048	Down
CCBL2	-0.603	-0.439	-1.561	Down
PLEKHA2	-0.564	-0.455	-1.4	Down
MOP-1	-0.606	-0.484	-1.485	Down
TCF3	-0.464	-0.352	-1.139	Down
STAG3L2	-0.504	-0.396	-1.263	Down
C9orf100	-0.38	-0.327	-1.02	Down
AGAP11	-0.579	-0.495	-1.511	Down
TCFL5	-0.625	-0.565	-1.61	Down
LOC142937	-0.686	-0.63	-1.806	Down
GAB2	-0.824	-0.784	-2.251	Down
DCP1B	-0.745	-0.724	-2.044	Down
TMEM69	-0.392	-0.376	-1.075	Down
STEAP1	-0.631	-0.382	-1.375	Down
LOC100125556	-0.516	-0.33	-1.149	Down
TRIM14	-0.545	-0.617	-1.179	Down
PACS2	-1.104	-1.227	-2.338	Down
C7orf49	-0.549	-0.619	-1.195	Down
LOC644450	-1.033	-1.212	-2.368	Down
GTF3C2	-0.559	-0.632	-1.254	Down
SCARNA13	-1.366	-1.67	-3.001	Down
ADCY3	-0.606	-0.744	-1.374	Down
XRRA1	-0.449	-0.54	-1.083	Down
RGS19	-0.937	-1.141	-2.284	Down
C1orf74	-0.6	-0.727	-1.437	Down
BHLHE23	-0.644	-0.75	-1.512	Down
SCARNA9	-1.321	-1.562	-3.107	Down
LOC653113	-1.016	-1.267	-2.553	Down
ASB4	-0.518	-0.66	-1.293	Down
SLC16A13	-0.776	-0.966	-1.838	Down
NLRX1	-0.574	-0.727	-1.39	Down
LOC100128719	-0.522	-0.662	-1.238	Down
FAM164C	-0.59	-0.781	-1.475	Down
ST6GAL1	-1.829	-2.206	-4.888	Down
METTL13	-0.785	-0.922	-2.053	Down
C15orf40	-0.39	-0.45	-1.012	Down
FAM43B	-0.603	-0.727	-1.529	Down
EIF3F	-0.634	-0.766	-1.614	Down
LOC100291206	-0.599	-0.658	-1.438	Down
LOC100129781	-1.019	-1.085	-2.49	Down
F2RL2	-0.663	-0.846	-1.457	Down
C5orf54	-1.268	-1.581	-2.765	Down
TERC	-0.754	-0.992	-1.694	Down
LOC401037	-0.719	-0.932	-1.613	Down
DOLK	-0.468	-0.613	-1.04	Down
C8orf30A	-0.762	-1.011	-1.685	Down
MRP63	-0.499	-0.683	-1.127	Down
TSFM	-0.474	-0.667	-1.074	Down

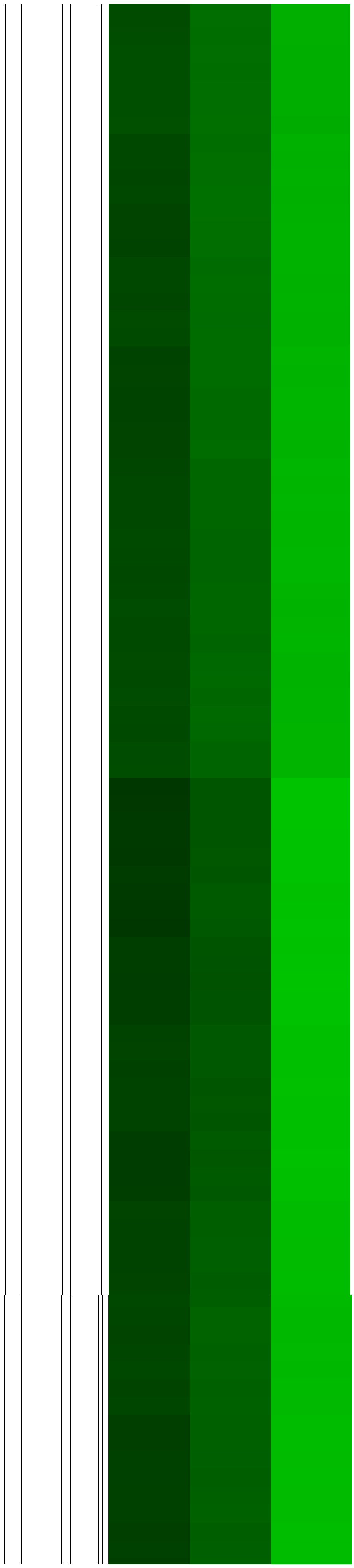

MANSC1	-1.408	-2.057	-3.277	Down
OR6C2	-0.736	-1.032	-1.662	Down
LRP3	-0.864	-1.222	-1.927	Down
EIF2B1	-0.555	-0.769	-1.221	Down
DECR1	-0.599	-0.835	-1.319	Down
KLHL31	-0.554	-0.773	-1.218	Down
C14orf169	-0.905	-1.258	-1.941	Down
SETD6	-0.482	-0.732	-1.181	Down
RGS14	-0.954	-1.49	-2.352	Down
PRIM1	-1.182	-1.874	-2.998	Down
LOC100132733	-0.796	-1.256	-1.979	Down
MSRB2	-0.659	-1.087	-1.716	Down
HS6ST1	-0.981	-1.594	-2.558	Down
PFKM	-0.525	-0.861	-1.39	Down
RNASEN	-0.659	-0.974	-1.613	Down
LOC100133315	-0.642	-0.977	-1.588	Down
ZNF497	-0.716	-1.106	-1.816	Down
OTX1	-0.558	-0.796	-1.313	Down
LOC143286	-0.651	-0.943	-1.545	Down
TRADD	-0.948	-1.553	-2.573	Down
KIAA0196	-0.621	-0.983	-1.63	Down
LOC158572	-0.434	-0.685	-1.171	Down
C14orf159	-0.704	-1.103	-1.884	Down
C14orf72	-0.752	-1.162	-1.979	Down
BCAS3	-0.599	-0.95	-1.578	Down
SMARCAL1	-1.251	-1.821	-3.247	Down
RABL2A	-0.415	-0.601	-1.053	Down
LOC645249	-1.62	-2.325	-4.135	Down
IMPACT	-0.938	-1.357	-2.384	Down
RIMBP3	-2.002	-2.749	-4.89	Down
DGKA	-0.552	-0.754	-1.365	Down
WDR34	-0.535	-0.752	-1.347	Down
SORBS3	-0.535	-0.751	-1.308	Down
DAB2	-0.785	-1.063	-1.854	Down
C4orf42	-1.365	-1.898	-3.297	Down
C1orf59	-1.189	-1.621	-2.89	Down
KIAA1549	-0.786	-1.085	-1.855	Down
CHTF18	-0.502	-0.716	-1.203	Down
TRIM34	-0.752	-1.025	-1.769	Down
PTPRS	-0.433	-0.618	-1.048	Down
CDX2	-0.619	-0.873	-1.514	Down
HSPA1L	-0.7	-0.935	-1.663	Down
C1orf220	-0.994	-1.289	-2.303	Down
NPEPL1	-0.51	-0.801	-1.83	Down
LOC728449	-0.478	-0.722	-1.65	Down
RBM45	-1.045	-1.53	-3.5	Down
PTCD2	-0.427	-0.623	-1.414	Down
GTF2IRD1	-0.434	-0.657	-1.457	Down
CCDC88C	-0.35	-0.507	-1.153	Down
VPS29	-0.356	-0.557	-1.179	Down
GUSBL2	-0.453	-0.712	-1.528	Down
PRTFDC1	-0.461	-0.752	-1.64	Down
MMAA	-0.711	-0.962	-2.199	Down
LRRC20	-0.934	-1.241	-2.885	Down
LOC158435	-0.368	-0.492	-1.161	Down
AIMP2	-0.59	-0.787	-1.831	Down
USP41	-0.477	-0.639	-1.487	Down
C14orf101	-0.51	-0.671	-1.431	Down
BRCA1	-0.512	-0.673	-1.428	Down
B3GALT6	-1.057	-1.444	-3.09	Down
PMS2L11	-0.375	-0.488	-1.09	Down
F8A1	-0.746	-0.977	-2.146	Down
ATP5S	-0.451	-0.575	-1.277	Down
TMEM8C	-0.331	-0.485	-1.024	Down
MRS2	-0.633	-0.91	-1.998	Down
SLC35E2	-0.436	-0.635	-1.352	Down
PRELID2	-0.75	-1.063	-2.286	Down
LOC730413	-0.487	-0.665	-1.334	Down
LOC729088	-0.651	-0.908	-1.84	Down
LOC203274	-0.529	-0.743	-1.458	Down
LGALS3BP	-0.386	-0.546	-1.072	Down
SYNM	-0.619	-0.837	-1.703	Down
TMEM169	-0.552	-0.743	-1.474	Down
C3orf25	-0.417	-0.555	-1.094	Down
FAM50B	-0.723	-1.027	-1.905	Down
DGCR5	-0.566	-0.815	-1.52	Down
ZNF232	-0.949	-1.329	-2.519	Down
RUNX3	-0.458	-0.63	-1.186	Down
MTMR4	-1.343	-1.881	-3.645	Down
MRPL38	-0.555	-0.782	-1.493	Down
MOCS2	-0.625	-0.949	-1.862	Down
LRFN3	-0.857	-1.293	-2.524	Down
LOC401387	-0.38	-0.557	-1.091	Down
LOC100129502	-0.344	-0.507	-1.009	Down
UBAC1	-0.821	-1.219	-2.367	Down
STAT6	-0.956	-1.425	-2.735	Down
FAM127B	-0.436	-0.651	-1.297	Down
C14orf126	-0.508	-0.749	-1.47	Down

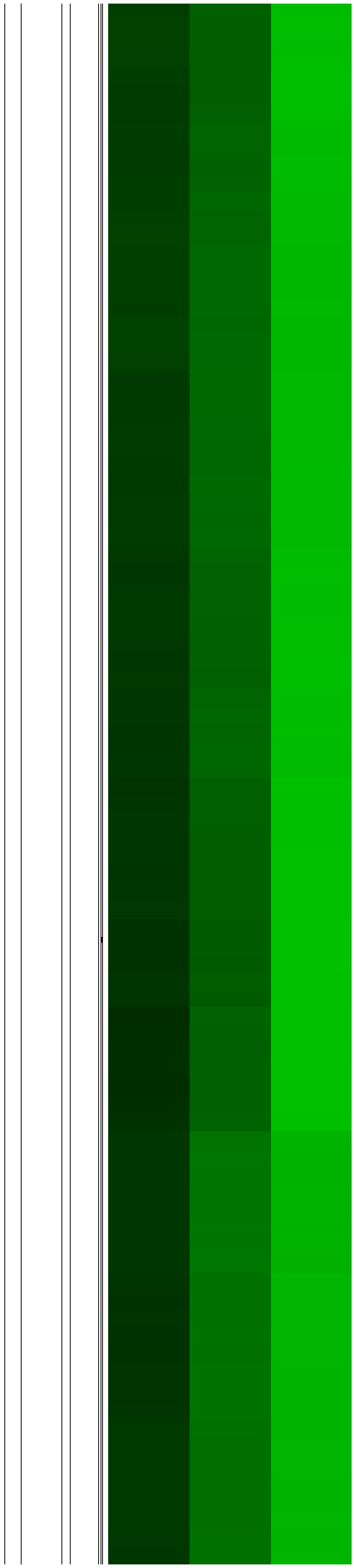

SPATA6	-0.464	-0.719	-1.404	Down
KCTD12	-0.596	-0.853	-1.73	Down
HVCN1	-0.395	-0.569	-1.145	Down
GYS2	-0.475	-0.695	-1.401	Down
A4GALT	-0.584	-0.874	-1.788	Down
TUBBP5	-0.687	-1.071	-2.187	Down
TCIRG1	-0.349	-0.552	-1.098	Down
POP4	-0.368	-0.594	-1.109	Down
LOC645937	-0.332	-0.551	-1.016	Down
EEF2K	-1.558	-2.507	-4.84	Down
C17orf80	-0.377	-0.593	-1.129	Down
INTS8	-0.366	-0.605	-1.085	Down
ADAT3	-0.551	-0.882	-1.616	Down
MKS1	-0.38	-0.584	-1.08	Down
ZBTB45	-0.58	-0.96	-1.68	Down
PLCH1	-0.808	-1.338	-2.352	Down
FZD1	-0.482	-0.795	-1.396	Down
FAM78A	-1.235	-2.056	-3.625	Down
LOC100288412	-0.448	-0.698	-1.247	Down
CTPS2	-0.612	-0.965	-1.697	Down
ZFP90	-1.253	-2.014	-3.537	Down
ZNF362	-0.979	-1.791	-3.107	Down
RASSF2	-0.629	-1.127	-1.98	Down
MPI	-0.758	-1.382	-2.409	Down
LOC728806	-0.475	-0.834	-1.47	Down
RPUSD1	-0.979	-1.661	-3.011	Down
LOC100129596	-0.616	-1.074	-1.967	Down
IMPA2	-0.788	-1.44	-2.542	Down
GTF2E1	-0.403	-0.714	-1.259	Down
C11orf49	-0.499	-0.869	-1.564	Down
AKAP1	-0.493	-0.855	-1.523	Down
TMEM106C	-0.377	-0.671	-1.254	Down
POMT1	-0.329	-0.583	-1.117	Down
TRIM27	-0.327	-0.567	-1.071	Down
HLCS	-0.406	-0.679	-1.3	Down
PIK3R2	-0.709	-1.181	-2.288	Down
GPS1	-0.392	-0.673	-1.315	Down
CTDSPL	-0.939	-1.681	-3.277	Down
C1orf25	-0.366	-0.625	-1.227	Down
ZNF174	-0.318	-0.594	-1.105	Down
SIRT3	-0.52	-0.981	-1.809	Down
ZNF25	-0.423	-0.797	-1.507	Down
SCO2	-0.498	-0.953	-1.754	Down
NTAN1	-0.392	-0.74	-1.337	Down
TCEA2	-1.15	-2.121	-4.279	Down
SLC2A4RG	-0.34	-0.609	-1.211	Down
ZNF485	-0.35	-0.618	-1.222	Down
ZFPM1	-0.343	-0.592	-1.204	Down
PRPSAP1	-0.454	-0.774	-1.592	Down
PRKCDBP	-0.708	-1.228	-2.534	Down
PIGZ	-0.569	-0.953	-1.963	Down
CCDC71	-0.626	-1.027	-2.119	Down
POP5	-0.516	-0.949	-2.015	Down
C16orf42	-0.723	-1.344	-2.824	Down
THAP4	-0.357	-0.665	-1.414	Down
PIR	-0.655	-1.169	-2.418	Down
DOK1	-0.871	-1.529	-3.227	Down
ZNF658	-0.718	-1.58	-3.097	Down
PAR-SN	-0.466	-0.981	-1.941	Down
NAPRT1	-0.35	-0.69	-1.396	Down
MRPL55	-0.285	-0.589	-1.166	Down
CDC16	-0.347	-0.744	-1.467	Down
ANUBL1	-0.256	-0.524	-1.038	Down
C6orf203	-0.549	-1.067	-2.082	Down
ANAPC4	-0.503	-1.077	-1.675	Down
ADAT1	-0.964	-2.091	-3.176	Down
SMO	-1.187	-2.505	-3.834	Down
RPP40	-0.622	-1.339	-2.067	Down
RNF43	-0.953	-2.047	-3.162	Down
PSMB8	-0.426	-0.904	-1.373	Down
CCDC6	-0.545	-1.143	-1.752	Down
SNAPIN	-0.712	-1.577	-2.335	Down
XRCC6BP1	-0.57	-1.235	-1.984	Down
RGS10	-0.625	-1.419	-2.284	Down
MUTYH	-0.727	-1.596	-2.58	Down
MARVELD1	-0.805	-1.852	-2.944	Down
LOC283481	-0.473	-1.06	-1.7	Down
EPN3	-0.302	-0.668	-1.065	Down
CPS1	-0.504	-1.126	-1.789	Down
C16orf13	-0.313	-0.678	-1.074	Down
PARN	-0.324	-0.63	-1.018	Down
NCRNA00203	-0.337	-0.646	-1.062	Down
FARSB	-0.421	-0.804	-1.324	Down
FAM46A	-0.564	-1.088	-1.777	Down
FABP5	-0.5	-0.972	-1.588	Down
APITD1	-0.736	-1.413	-2.345	Down
RNF157	-0.424	-0.842	-1.366	Down
NICN1	-1.103	-2.289	-3.678	Down

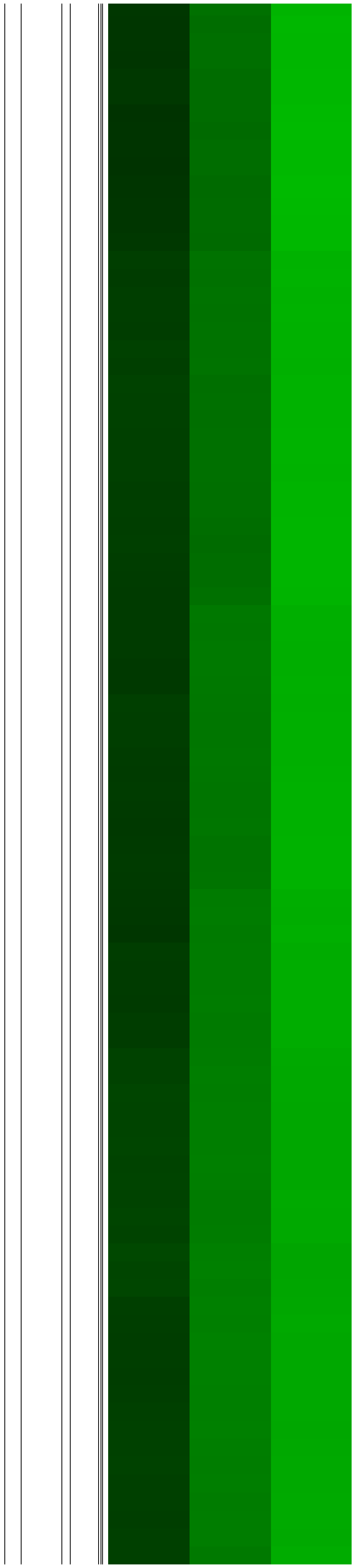

ZNHIT2	-0.453	-0.925	-1.465	Down
NCRNA00181	-0.408	-0.819	-1.373	Down
FLJ45248	-0.587	-1.199	-1.962	Down
VDAC3	-0.569	-1.177	-1.934	Down
RASSF10	-0.646	-1.248	-2.109	Down
NT5DC1	-0.499	-0.952	-1.609	Down
KIAA0100	-0.602	-1.274	-2.181	Down
HPS6	-0.965	-1.978	-3.441	Down
DOCK1	-0.347	-0.718	-1.211	Down
C7orf70	-0.423	-0.89	-1.507	Down
PLK1S1	-0.378	-0.755	-1.319	Down
HERC2P7	-0.387	-0.763	-1.312	Down
ACBD4	-0.389	-0.762	-1.312	Down
MARCH9	-0.344	-0.65	-1.126	Down
MRPL40	-0.497	-0.916	-1.426	Down
AZI1	-0.831	-1.566	-2.476	Down
C20orf27	-0.413	-0.764	-1.167	Down
ARRDC1	-0.424	-0.773	-1.204	Down
UBE2D4	-0.76	-1.432	-2.192	Down
SEPHS2	-0.522	-0.906	-1.403	Down
CXXC1	-0.384	-0.694	-1.06	Down
SCARNA5	-0.554	-0.934	-1.499	Down
NUBPL	-0.574	-0.99	-1.569	Down
KIAA1462	-0.576	-0.984	-1.573	Down
C16orf74	-1.047	-1.821	-2.911	Down
AKR1C4	-0.537	-0.937	-1.498	Down
AGBL5	-0.692	-1.209	-1.923	Down
PTPN18	-0.817	-1.457	-2.361	Down
NARG2	-0.755	-1.326	-2.15	Down
USE1	-0.435	-0.771	-1.272	Down
POLR2I	-0.496	-0.847	-1.428	Down
LOC100134702	-0.35	-0.628	-1.039	Down
DDTL	-0.45	-0.811	-1.347	Down
TTLL6	-0.52	-0.982	-1.573	Down
CRYBB2P1	-0.368	-0.748	-1.085	Down
CHST12	-0.695	-1.402	-2.054	Down
RBBP9	-0.803	-1.644	-2.359	Down
PALB2	-0.678	-1.421	-2.044	Down
MUC5B	-0.653	-1.373	-2.002	Down
MSTO2P	-0.374	-0.706	-1.033	Down
MRPL18	-0.387	-0.733	-1.084	Down
LIPE	-0.451	-0.858	-1.266	Down
CYP17A1	-0.365	-0.719	-1.059	Down
NMRAL1	-0.814	-1.629	-2.417	Down
LOH12CR1	-0.602	-1.169	-1.772	Down
LOC439949	-0.687	-1.381	-2.097	Down
ACADSB	-0.742	-1.526	-2.277	Down
FAM173B	-0.361	-0.715	-1.102	Down
ABCA3	-1.012	-1.989	-3.059	Down
XRCC1	-0.699	-1.389	-2.126	Down
TTL	-0.365	-0.787	-1.115	Down
TBC1D2B	-0.623	-1.371	-1.911	Down
DCTPP1	-0.518	-1.163	-1.663	Down
NSMCE1	-0.674	-1.356	-1.903	Down
MYLK	-0.897	-1.868	-2.618	Down
CBR3	-1.56	-3.266	-4.573	Down
BCKDK	-0.621	-1.322	-1.845	Down
TUB	-0.788	-1.576	-2.241	Down
PDE7B	-1.143	-2.325	-3.234	Down
F12	-0.869	-1.62	-2.199	Down
CDC45L	-0.548	-1.034	-1.381	Down
C9orf152	-0.582	-1.045	-1.404	Down
C20orf94	-1.217	-2.252	-2.985	Down
RARS2	-0.601	-1.117	-1.476	Down
ONECUT1	-0.797	-1.449	-1.918	Down
LOC283861	-0.951	-1.813	-2.367	Down
FSD1	-0.808	-1.489	-1.958	Down
KLF17	-0.453	-0.826	-1.137	Down
C20orf108	-0.964	-1.716	-2.366	Down
TBL3	-0.442	-0.82	-1.119	Down
ZNRF3	-0.61	-1.079	-1.401	Down
ZNF771	-0.787	-1.443	-1.866	Down
TMEM150A	-0.656	-1.178	-1.544	Down
LARGE	-1.121	-2.27	-2.959	Down
HDLBP	-0.375	-0.761	-1.01	Down
EBF4	-0.378	-0.793	-1.028	Down
BDH1	-0.783	-1.622	-2.127	Down
THAP8	-1.15	-2.393	-3.151	Down
NPDC1	-0.468	-0.947	-1.256	Down
TRPT1	-0.465	-0.924	-1.225	Down
SMARCD3	-0.827	-1.631	-2.123	Down
MEX3A	-1.24	-2.412	-3.213	Down
FLJ90757	-1.42	-2.672	-3.602	Down
PNMAL1	-1.092	-2.156	-2.875	Down
GSTA4	-0.698	-1.363	-1.86	Down
C5orf13	-0.46	-0.923	-1.252	Down
ADD2	-0.537	-1.049	-1.414	Down
NIT1	-0.399	-0.758	-1.072	Down

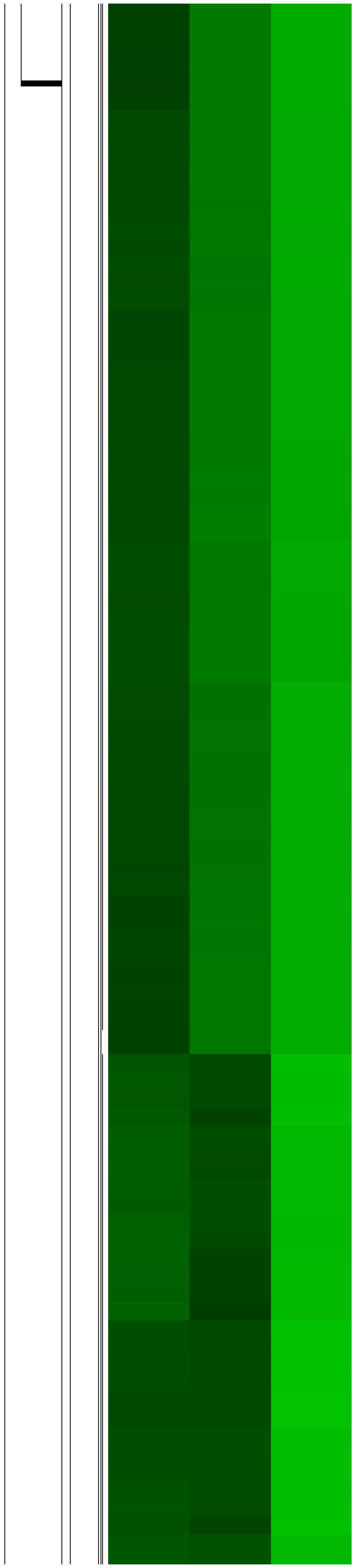

HYAL3	-0.482	-0.906	-1.264	Down
UBE2L6	-0.427	-0.805	-1.121	Down
TUSC4	-0.65	-1.271	-1.763	Down
TPD52L1	-0.487	-0.933	-1.302	Down
TBCD	-0.801	-1.549	-2.155	Down
SGSM2	-0.468	-0.89	-1.235	Down
RBKS	-0.514	-0.824	-1.167	Down
PTPRU	-0.557	-0.917	-1.282	Down
PGP	-0.526	-0.847	-1.212	Down
PCDHB9	-0.518	-0.856	-1.21	Down
SLC9A3R2	-1.005	-1.639	-2.349	Down
RFXANK	-0.669	-1.081	-1.561	Down
LOC100287593	-0.535	-0.823	-1.187	Down
IL4I1	-0.447	-0.71	-1.015	Down
TIGD5	-0.886	-1.358	-1.999	Down
TGFB1I1	-0.725	-1.126	-1.643	Down
MYO10	-0.895	-1.361	-1.987	Down
SIPA1L1	-0.504	-0.876	-1.211	Down
SENP8	-0.748	-1.29	-1.796	Down
ZBTB42	-1.526	-2.695	-3.685	Down
MLPH	-0.893	-1.51	-2.117	Down
KRBA2	-0.442	-0.741	-1.028	Down
DZIP1	-0.822	-1.367	-1.914	Down
COLEC11	-0.563	-0.961	-1.331	Down
ZCCHC17	-0.691	-1.157	-1.561	Down
TMEM8B	-0.703	-1.166	-1.569	Down
CBX5	-0.527	-0.889	-1.198	Down
C14orf133	-0.925	-1.544	-2.108	Down
RNF34	-0.565	-0.966	-1.258	Down
CYB561D2	-0.527	-0.895	-1.168	Down
SHMT1	-0.799	-1.252	-1.78	Down
PCDHGA3	-0.568	-0.879	-1.242	Down
LOC100129362	-0.471	-0.741	-1.033	Down
CCNB3	-0.51	-0.818	-1.127	Down
CPSF3	-0.67	-1.077	-1.462	Down
C1orf203	-0.485	-0.759	-1.035	Down
FIG4	-0.615	-0.947	-1.305	Down
LOC375295	-1.065	-1.651	-2.219	Down
DTNBP1	-0.927	-1.386	-2.12	Down
DARS2	-0.806	-1.231	-1.85	Down
CENPBD1	-0.908	-1.436	-2.143	Down
AMDHD1	-0.96	-1.474	-2.217	Down
POLDIP2	-0.72	-1.112	-1.703	Down
LOC645261	-0.444	-0.677	-1.059	Down
TIGD2	-0.697	-1.109	-1.681	Down
SLFN5	-0.444	-0.716	-1.084	Down
LOC100288106	-0.418	-0.669	-1.009	Down
EMILIN2	-0.793	-1.252	-1.897	Down
CT62	-0.673	-1.096	-1.663	Down
ATIC	-0.539	-0.874	-1.306	Down
ZNF688	-0.765	-1.343	-2.012	Down
TMCC1	-0.683	-1.19	-1.763	Down
PDK2	-0.808	-1.359	-2.045	Down
TNFAIP8L1	-0.449	-0.762	-1.126	Down
HERC6	-0.461	-0.812	-1.177	Down
ASPSCR1	-0.514	-0.904	-1.298	Down
PTDSS1	-0.902	-1.591	-2.297	Down
RPGRIP1L	-0.504	-0.912	-1.329	Down
ILDR1	-0.38	-0.704	-1.012	Down
CISD3	-0.753	-0.645	-1.675	Down
MEFV	-0.649	-0.526	-1.374	Down
LOC400128	-0.585	-0.493	-1.26	Down
PIGV	-0.619	-0.465	-1.308	Down
EN2	-0.708	-0.579	-1.402	Down
APOL2	-0.511	-0.417	-1.024	Down
TINAGL1	-0.831	-0.653	-1.646	Down
LOC645431	-1.124	-0.951	-2.251	Down
COQ4	-0.592	-0.511	-1.19	Down
NRG2	-1.212	-0.973	-2.31	Down
NMI	-0.675	-0.51	-1.257	Down
ASS1	-0.767	-0.523	-1.458	Down
LOC642826	-0.53	-0.37	-1.046	Down
HAGHL	-0.567	-0.406	-1.119	Down
SNAR-C4	-0.589	-0.373	-1.126	Down
LRFN1	-1.281	-1.182	-3.18	Down
RUNDC2A	-0.509	-0.481	-1.235	Down
RPUSD2	-0.6	-0.585	-1.518	Down
LOC284373	-0.419	-0.399	-1.026	Down
TBL1X	-0.497	-0.49	-1.303	Down
LOC100272216	-0.445	-0.435	-1.157	Down
EMILIN1	-0.666	-0.665	-1.581	Down
CCS	-0.43	-0.438	-1.051	Down
AUTS2	-0.78	-0.762	-1.893	Down
PFKL	-0.796	-0.741	-1.832	Down
FAM86A	-0.606	-0.553	-1.387	Down
PHOSPHO2	-0.687	-0.579	-1.637	Down
HAPLN2	-0.666	-0.647	-1.459	Down
FLJ22184	-0.752	-0.694	-1.595	Down

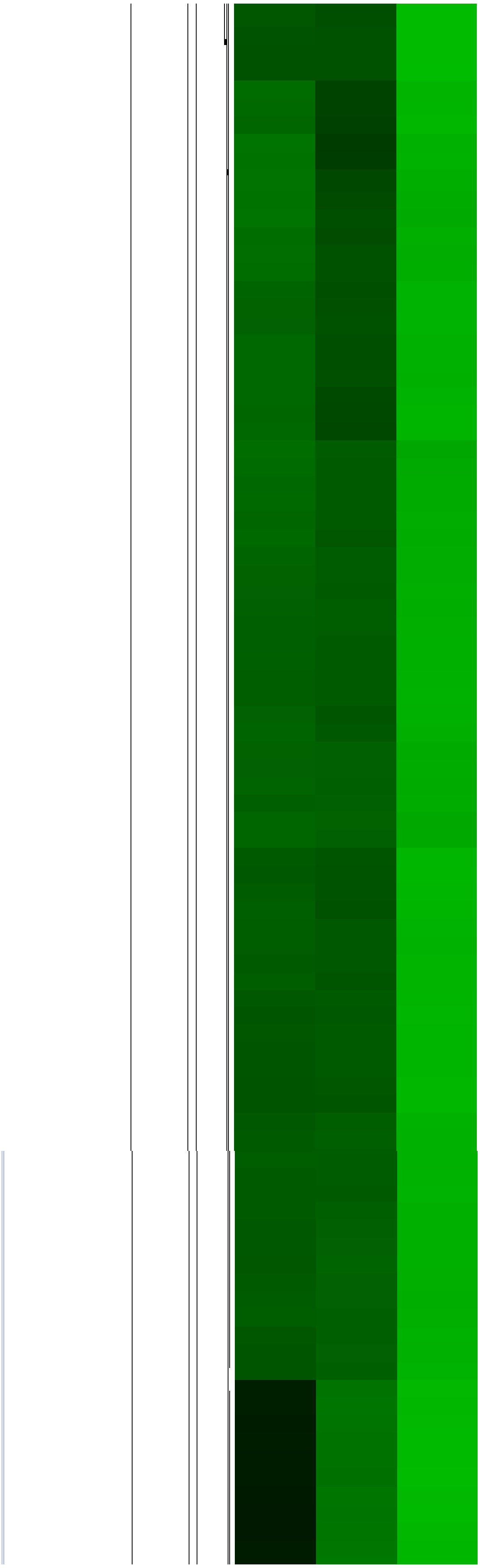

LOC283174	-0.578	-0.52	-1.242	Down
FERMT1	-0.987	-0.962	-2.22	Down
ELFN1	-0.591	-0.597	-1.358	Down
CEBPD	-0.643	-0.643	-1.483	Down
LOC389458	-0.803	-0.492	-1.34	Down
CCDC78	-0.613	-0.38	-1.046	Down
RHBDL1	-0.678	-0.425	-1.215	Down
LRRC45	-1.137	-0.592	-1.729	
LOC645781	-0.644	-0.346	-1.008	
LOC100134002	-0.793	-0.491	-1.2	Down
APOL1	-0.979	-0.645	-1.473	Down
ADAP2	-0.855	-0.58	-1.251	Down
LOC100130152	-0.693	-0.489	-1.106	Down
WDR18	-0.742	-0.546	-1.163	Down
MOCS3	-0.98	-0.729	-1.563	Down
UCP3	-1.054	-0.828	-1.859	Down
NAGK	-0.726	-0.584	-1.306	Down
LOC100134663	-0.643	-0.541	-1.177	Down
HOXA7	-0.972	-0.739	-1.66	Down
AMN	-0.771	-0.584	-1.309	Down
SCLY	-0.793	-0.613	-1.338	Down
TTLL8	-0.829	-0.591	-1.418	Down
SNORA71A	-0.763	-0.544	-1.341	Down
KCTD19	-1.192	-0.812	-2.066	Down
LOC100134253	-0.672	-0.572	-1.033	Down
FAM131C	-0.659	-0.55	-1.041	Down
LOC100129447	-0.862	-0.743	-1.411	Down
CCDC122	-1.179	-1.018	-1.907	Down
SCGB3A1	-0.642	-0.567	-1.086	Down
EVX1	-0.994	-0.815	-1.617	Down
LOC646999	-0.63	-0.581	-1.086	Down
LOC645553	-0.752	-0.697	-1.312	Down
HSD17B7	-0.778	-0.727	-1.39	Down
LOC113230	-0.81	-0.79	-1.456	Down
LOC100134237	-0.551	-0.537	-1.008	Down
CARS2	-0.636	-0.609	-1.172	Down
C8orf47	-0.812	-0.773	-1.492	Down
C18orf23	-0.605	-0.588	-1.134	Down
WDR81	-0.917	-0.888	-1.745	Down
DPEP3	-0.726	-0.641	-1.31	Down
C6orf163	-1.017	-0.885	-1.764	Down
TDRD7	-0.59	-0.583	-1.021	Down
DET1	-1.456	-1.437	-2.547	Down
CASP6	-1.223	-1.179	-2.084	Down
LCMT2	-1.443	-1.46	-2.578	Down
SNCB	-0.662	-0.647	-1.093	Down
NHLRC1	-0.664	-0.631	-1.094	Down
TTC5	-0.514	-0.479	-1.024	Down
NCK2	-0.748	-0.712	-1.55	Down
LOC100129702	-0.844	-0.756	-1.658	Down
FHL1	-0.893	-0.764	-1.709	Down
PFKFB1	-0.726	-0.693	-1.396	Down
BTC	-0.693	-0.658	-1.322	Down
DPY19L2P2	-0.584	-0.562	-1.149	Down
CDRT8	-0.647	-0.584	-1.231	Down
PNMA6A	-0.896	-0.916	-1.845	Down
CDC42EP5	-0.49	-0.504	-1.034	Down
C15orf39	-0.856	-0.885	-1.774	Down
BNIP3	-0.573	-0.602	-1.201	Down
NCRNA00176	-0.653	-0.701	-1.389	Down
LOC100133994	-0.817	-0.851	-1.781	Down
FAM124A	-0.871	-0.887	-1.893	Down
GINS4	-1.073	-1.122	-2.134	Down
FYCO1	-0.795	-0.834	-1.555	Down
PCSK1N	-0.758	-0.789	-1.492	Down
PSMB9	-0.573	-0.582	-1.107	Down
NCF2	-1.029	-1.021	-1.946	Down
UTS2R	-0.622	-0.635	-1.22	Down
LBX1	-0.506	-0.508	-1.003	Down
PELI3	-1.348	-1.43	-2.612	Down
LOC643551	-0.521	-0.57	-1.028	Down
NEIL2	-0.572	-0.634	-1.13	Down
GALR3	-0.604	-0.689	-1.209	Down
WDR61	-0.648	-0.708	-1.263	Down
LOC727788	-0.537	-0.571	-1.012	Down
DKFZp547G183	-0.743	-0.77	-1.396	Down
SSTR3	-0.521	-0.576	-1.063	Down
FAM86B1	-0.613	-0.699	-1.259	Down
RAG1	-0.63	-0.709	-1.325	Down
SYTL4	-0.172	-0.646	-1.039	Down
TMEM102	-0.389	-1.517	-2.393	Down
HNRNPA1L2	-0.332	-1.369	-2.168	Down
FLJ35776	-0.207	-0.801	-1.297	Down
AGGF1	-0.233	-0.954	-1.549	Down
FUK	-0.233	-0.931	-1.543	Down
RAB3A	-0.277	-1.236	-1.947	Down
C11orf2	-0.153	-0.705	-1.129	Down
ZC3H6	-0.197	-0.93	-1.452	Down

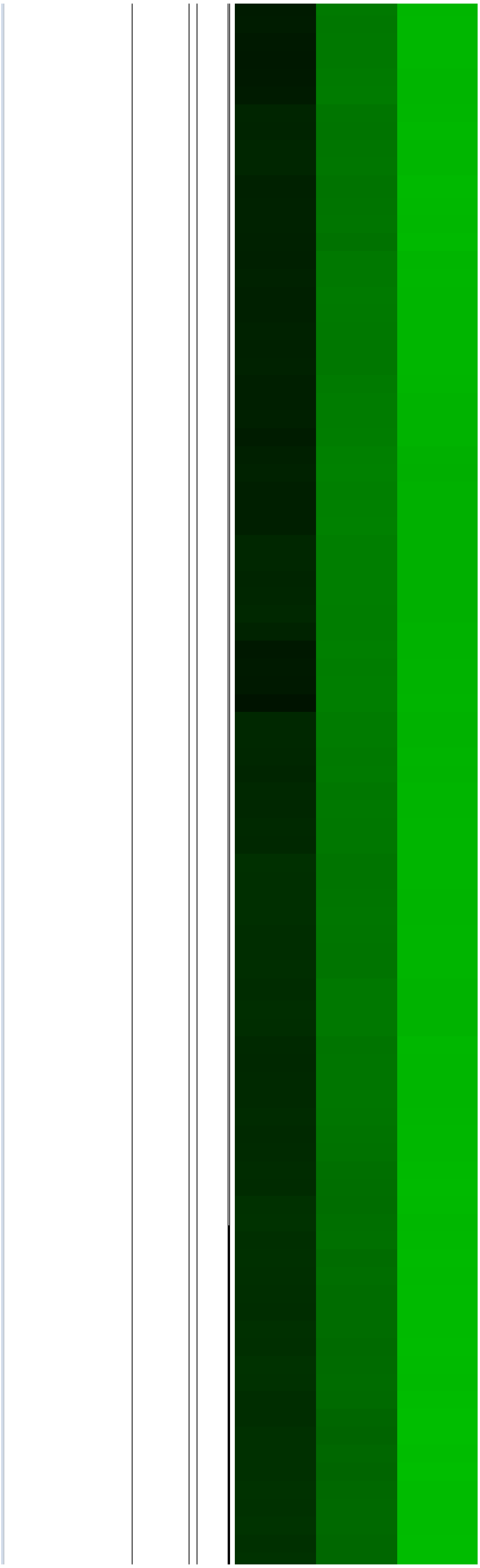

POPDC3	-0.28	-1.184	-1.821	Down
PEBP4	-0.363	-1.603	-2.414	Down
SCN1B	-0.204	-0.845	-1.303	Down
VMO1	-0.301	-1.402	-2.133	Down
MIF4GD	-0.206	-1.029	-1.562	Down
EVL	-0.213	-1.041	-1.54	Down
DFNB59	-0.268	-1.223	-1.792	Down
UBE2Q2	-0.222	-0.707	-1.096	Down
TTLL1	-0.474	-1.507	-2.394	Down
TCHP	-0.42	-1.301	-2.017	Down
RDBP	-0.523	-1.586	-2.451	Down
CSK	-0.364	-1.244	-1.995	Down
ALDH16A1	-0.198	-0.664	-1.049	Down
KIF3C	-0.326	-1.11	-1.736	Down
TRIM16L	-0.451	-1.543	-2.489	Down
HR	-0.257	-0.965	-1.453	Down
GATS	-0.384	-1.343	-2.036	Down
SERGEF	-0.188	-0.709	-1.051	Down
NT5DC2	-0.195	-0.732	-1.093	Down
KRTAP3-3	-0.205	-0.724	-1.088	Down
CCNI	-0.256	-0.908	-1.39	Down
C4orf33	-0.277	-0.965	-1.482	Down
HADH	-0.22	-0.866	-1.281	Down
ARRB2	-0.203	-0.815	-1.177	Down
PLA2G16	-0.186	-0.722	-1.036	Down
ANXA4	-0.34	-1.488	-2.122	Down
S1PR4	-0.198	-0.783	-1.073	Down
MAPT	-0.397	-1.503	-2.05	Down
HPDL	-0.393	-1.605	-2.239	Down
FRMD3	-0.241	-0.988	-1.36	Down
PTPRA	-0.362	-1.48	-2.021	Down
C3orf39	-0.295	-0.954	-1.334	Down
ACAA1	-0.323	-1.042	-1.448	Down
FAM13A	-0.259	-0.864	-1.202	Down
MC1R	-0.332	-1.09	-1.52	Down
CCDC53	-0.432	-1.341	-1.885	Down
BBS9	-0.231	-0.814	-1.158	Down
SARS2	-0.146	-0.761	-1.072	Down
RDM1	-0.324	-1.562	-2.23	Down
MAP6	-0.17	-0.847	-1.207	Down
ACOX2	-0.435	-2.809	-3.998	Down
TMEM66	-0.291	-0.881	-1.274	Down
S1PR3	-0.358	-1.098	-1.579	Down
SLC37A4	-0.413	-1.262	-1.881	Down
OAZ1	-0.331	-1.069	-1.568	Down
MYO5C	-0.261	-0.767	-1.159	Down
MYH10	-0.515	-1.576	-2.357	Down
MLST8	-0.288	-0.824	-1.254	Down
KAT2A	-0.504	-1.484	-2.245	Down
TTC30B	-1.068	-2.536	-3.963	Down
TPCN1	-0.463	-1.128	-1.76	Down
RNASEH2A	-0.466	-1.163	-1.785	Down
NIPSNAP1	-0.433	-1.076	-1.645	Down
POLL	-0.397	-1.034	-1.597	Down
HES2	-0.457	-1.188	-1.845	Down
ZNF358	-0.539	-1.345	-2.101	Down
NR2E3	-0.284	-0.78	-1.157	Down
LCMT1	-0.433	-1.132	-1.692	Down
PLD2	-0.56	-1.464	-2.188	Down
PDE8B	-0.788	-2.202	-3.475	Down
HGSNAT	-0.258	-0.753	-1.17	Down
UBL7	-0.265	-0.751	-1.168	Down
SLC27A2	-0.457	-1.301	-1.994	Down
SDSL	-0.614	-1.659	-2.577	Down
WFS1	-0.356	-1	-1.589	Down
ST6GALNAC4	-0.336	-0.91	-1.458	Down
TSEN54	-0.502	-1.25	-2.073	Down
LOC100130506	-0.302	-0.775	-1.302	Down
HEXDC	-0.853	-1.885	-3.188	Down
EPB41L4A	-0.941	-2.086	-3.446	Down
UFSP1	-0.609	-1.454	-2.384	Down
OR5L2	-0.676	-1.521	-2.613	Down
NR5A2	-0.298	-0.685	-1.151	Down
PREB	-0.357	-0.833	-1.425	Down
NIPAL3	-0.478	-1.153	-1.982	Down
PGLS	-0.346	-0.778	-1.342	Down
GGTLC1	-0.402	-0.904	-1.598	Down
FLJ32065	-0.333	-0.712	-1.238	Down
AMZ2	-0.445	-0.98	-1.68	Down
RECQL5	-0.37	-0.867	-1.531	Down
FCHO1	-0.569	-1.277	-2.368	Down
DOCK11	-0.52	-1.07	-2.02	Down
CPSF4	-0.29	-0.615	-1.124	Down
C17orf108	-0.819	-1.71	-3.2	Down
LOC400099	-1.029	-2.144	-3.854	Down
LOC100129888	-0.676	-1.435	-2.548	Down
OASL	-0.545	-1.12	-1.984	Down
DFNA5	-0.284	-0.602	-1.097	Down

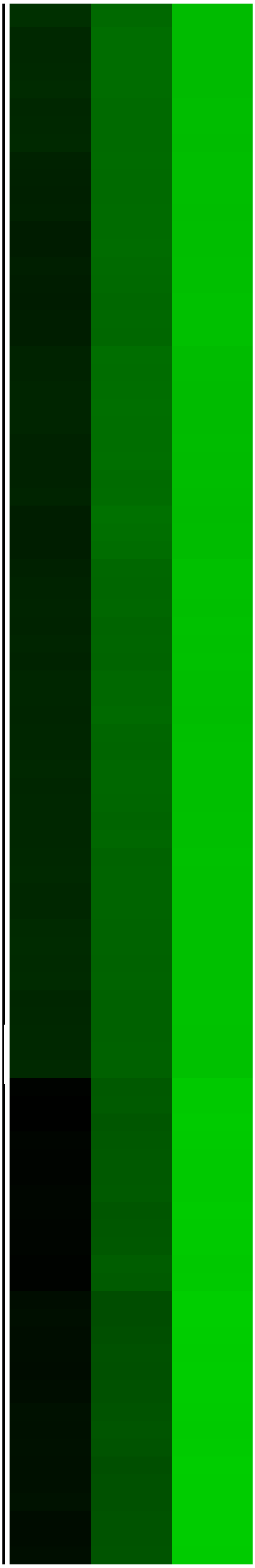

CREB3L4	-0.751	-1.531	-2.784	Down
ZNF395	-0.39	-0.844	-1.507	Down
SQRDL	-0.418	-1.123	-1.927	Down
LOC440104	-0.327	-0.871	-1.498	Down
LOC91948	-0.645	-1.676	-2.88	Down
LFNG	-0.35	-0.865	-1.501	Down
SLC22A18	-0.321	-0.87	-1.555	Down
FAM55C	-0.625	-1.619	-2.886	Down
ZMYND8	-0.564	-1.467	-2.591	Down
GALT	-0.316	-0.989	-1.753	Down
DYRK4	-0.21	-0.671	-1.208	Down
RSPH3	-0.189	-0.623	-1.112	Down
RICH2	-0.221	-0.717	-1.262	Down
PGM5P2	-0.156	-0.583	-1.037	Down
HEXIM1	-0.276	-1.055	-1.848	Down
MEGF8	-0.311	-1.061	-1.907	Down
GSDMD	-0.345	-1.241	-2.252	Down
FAM81A	-0.377	-1.356	-2.532	Down
CD44	-0.235	-0.799	-1.472	Down
MDP1	-0.297	-1.01	-1.887	Down
HEXIM2	-0.786	-2.445	-4.179	Down
AMACR	-0.378	-1.16	-2.009	Down
TMEM159	-0.292	-0.863	-1.463	Down
CXorf42	-0.432	-1.262	-2.126	Down
C2orf74	-0.333	-0.974	-1.655	Down
OSBPL1A	-0.396	-1.27	-2.162	Down
NIPSNAP3A	-0.43	-1.404	-2.364	Down
PHKB	-0.348	-1.081	-1.905	Down
MTUS1	-0.488	-1.455	-2.529	Down
LOC730202	-0.29	-1.089	-1.803	Down
CDON	-0.341	-1.255	-2.12	Down
SRI	-0.215	-0.763	-1.311	Down
KBTBD3	-0.247	-0.756	-1.406	Down
DEPDC6	-0.235	-0.684	-1.286	Down
ZNF483	-0.268	-0.755	-1.404	Down
C6orf176	-0.719	-2.018	-3.868	Down
AKR1C3	-0.272	-0.746	-1.409	Down
RBL2	-0.318	-0.89	-1.712	Down
P2RY6	-0.424	-1.153	-2.097	Down
MSH2	-0.34	-0.934	-1.706	Down
PWP2	-0.206	-0.584	-1.038	Down
RBBP7	-0.223	-0.592	-1.111	Down
LOC149401	-0.221	-0.576	-1.088	Down
IMPDH2	-0.434	-1.104	-2.046	Down
FASTK	-0.292	-0.788	-1.449	Down
FAM189B	-0.396	-1.016	-1.904	Down
EIF2B3	-0.423	-1.072	-2.053	Down
RHOBTB1	-0.659	-1.744	-3.225	Down
CNPY2	-0.783	-1.931	-3.756	Down
ARSE	-0.398	-0.961	-1.844	Down
SNX7	-0.235	-0.604	-1.154	Down
LOC100144604	-0.259	-0.652	-1.254	Down
HAUS4	-0.244	-0.581	-1.118	Down
FECH	-0.24	-0.553	-1.073	Down
FAM71E1	-0.283	-0.657	-1.285	Down
SPATA20	-0.244	-0.564	-1.086	Down
DDIT4L	-0.647	-1.648	-3.284	Down
BRD3	-0.208	-0.507	-1.014	Down
TACC2	-0.259	-0.634	-1.255	Down
hCG_2003663	-0.832	-2.02	-3.976	Down
ZNF18	-0.754	-1.762	-3.484	Down
LOC81691	-0.017	-0.48	-1.083	Down
C6orf154	-0.013	-1.163	-2.605	Down
NPHP1	-0.014	-0.635	-1.489	Down
TWF2	-0.033	-0.565	-1.286	Down
STEAP3	-0.041	-0.762	-1.715	Down
PEX6	-0.036	-0.76	-1.703	Down
DTWD2	-0.034	-0.471	-1.045	Down
PYGB	-0.048	-0.641	-1.513	Down
ATG4C	-0.039	-0.609	-1.408	Down
ACOT13	-0.038	-0.558	-1.269	Down
OSBPL7	-0.049	-1.154	-2.471	Down
LOC100131938	-0.025	-0.571	-1.256	Down
PYROXD2	-0.104	-0.582	-1.542	Down
MYO19	-0.111	-0.556	-1.472	Down
HS3ST1	-0.22	-1.288	-3.338	Down
DPH5	-0.12	-0.709	-1.782	Down
DALRD3	-0.082	-0.533	-1.349	Down
ATP7B	-0.115	-0.678	-1.716	Down
LOC100130111	-0.187	-0.884	-2.15	Down
FAM167A	-0.09	-0.459	-1.092	Down
PDK4	-0.125	-0.589	-1.429	Down
LIMK1	-0.156	-0.732	-1.889	Down
CAPN5	-0.255	-1.23	-3.096	Down
MAML3	-0.141	-0.604	-1.521	Down
RSAD1	-0.141	-0.942	-2.299	Down
MRPS26	-0.131	-0.866	-2.125	Down
FAHD2A	-0.075	-0.443	-1.073	Down

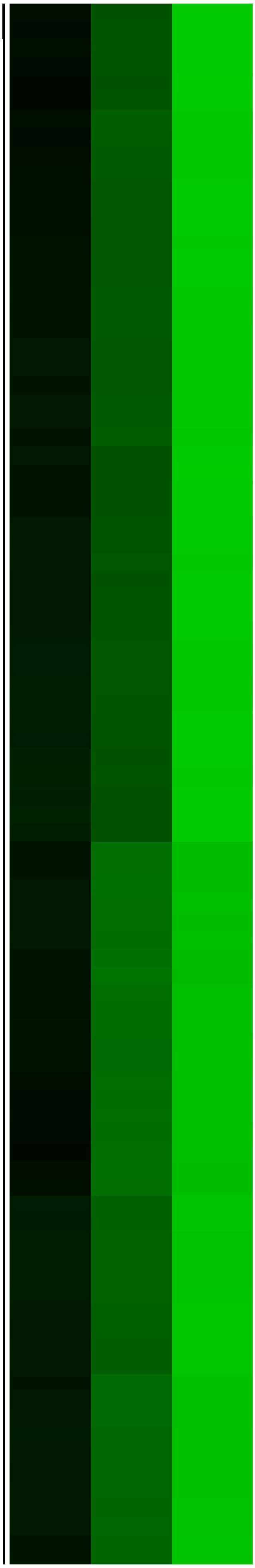

DGKG	-0.148	-0.925	-2.239	Down
RTEL1	-0.076	-0.509	-1.205	Down
PSD4	-0.081	-0.479	-1.117	Down
MVP	-0.08	-0.545	-1.273	Down
PALMD	-0.047	-0.42	-1.02	Down
MPST	-0.109	-0.974	-2.283	Down
TMEM117	-0.104	-0.68	-1.453	Down
HSPC157	-0.121	-0.869	-1.887	Down
CYBASC3	-0.109	-0.729	-1.6	Down
ALDH3B1	-0.136	-0.811	-1.773	Down
RNF135	-0.172	-0.93	-2.121	Down
GLI4	-0.141	-0.785	-1.758	Down
TUBAL3	-0.09	-0.46	-1.031	Down
PPOX	-0.181	-0.852	-1.903	Down
MYO15B	-0.15	-0.645	-1.468	Down
PDCD2L	-0.238	-1.007	-2.282	Down
MRPL24	-0.151	-0.618	-1.373	Down
LEPREL2	-0.188	-0.791	-1.723	Down
FHOD1	-0.131	-0.53	-1.17	Down
PPAPDC2	-0.141	-0.518	-1.171	Down
C12orf10	-0.165	-0.596	-1.352	Down
ZNF133	-0.134	-0.53	-1.185	Down
POLR3GL	-0.301	-1.082	-2.382	Down
NT5M	-0.298	-1.047	-2.308	Down
FAM120C	-0.121	-0.557	-1.183	Down
NDUFB10	-0.281	-0.974	-2.405	Down
NARF	-0.138	-0.518	-1.297	Down
NCRNA00173	-0.395	-1.538	-3.757	Down
ITFG3	-0.182	-0.741	-1.791	Down
CUL9	-0.283	-0.963	-2.252	Down
AGPHD1	-0.169	-0.542	-1.269	Down
ZNF839	-0.291	-1.001	-2.284	Down
TBC1D22A	-0.127	-0.438	-1.045	Down
MBOAT1	-0.191	-0.689	-1.639	Down
RNF130	-0.118	-0.426	-1.005	Down
GALK1	-0.248	-0.811	-1.916	Down
LOC100170939	-0.22	-0.694	-1.569	Down
C21orf59	-0.183	-0.571	-1.291	Down
ZNF514	-0.192	-0.548	-1.233	Down
CPOX	-0.339	-1.014	-2.341	Down
C10orf114	-0.331	-0.94	-2.206	Down
PBX1	-0.461	-1.361	-3.206	Down
VTI1B	-0.171	-0.437	-1.049	Down
LOC283267	-0.169	-0.432	-1.014	Down
GNPAT	-0.202	-0.519	-1.284	Down
C9orf23	-0.347	-0.833	-2.057	Down
PYDC1	-0.35	-0.923	-2.252	Down
RAB3D	-0.197	-1.01	-1.726	Down
LXN	-0.322	-1.713	-2.935	Down
TAF6L	-0.165	-0.751	-1.257	Down
C20orf54	-0.17	-0.748	-1.299	Down
ACACB	-0.178	-0.807	-1.394	Down
ST3GAL2	-0.167	-0.699	-1.233	Down
TENC1	-0.262	-1.425	-2.367	Down
LOC100134259	-0.407	-2.176	-3.517	Down
C7orf27	-0.15	-0.839	-1.47	Down
AAAS	-0.159	-0.882	-1.551	Down
JUP	-0.212	-1.141	-2.016	Down
LTBP1	-0.115	-0.661	-1.191	Down
C11orf65	-0.102	-0.576	-1.058	Down
C14orf167	-0.073	-0.594	-1.038	Down
BCS1L	-0.065	-0.635	-1.117	Down
TEX264	-0.102	-0.878	-1.51	Down
MRI1	-0.069	-0.699	-1.237	Down
DLL3	-0.068	-0.809	-1.386	Down
SLC7A7	-0.089	-0.733	-1.235	Down
NDUFV2	-0.098	-0.691	-1.177	Down
MON1A	-0.227	-0.781	-1.562	Down
ETHE1	-0.181	-0.632	-1.274	Down
LOC93622	-0.344	-1.143	-2.233	Down
GGT1	-0.185	-0.595	-1.169	Down
STAT2	-0.215	-0.686	-1.348	Down
LRRC37A3	-0.203	-0.681	-1.341	Down
EML3	-0.187	-0.691	-1.431	Down
C22orf36	-0.249	-1.002	-2.075	Down
NTHL1	-0.578	-2.053	-4.312	Down
HOXB13	-0.66	-2.424	-5.066	Down
LIPT2	-0.433	-2.074	-3.758	Down
CXXC5	-0.337	-1.47	-2.68	Down
NSMCE4A	-0.254	-1.062	-1.939	Down
LOC100129791	-0.156	-0.588	-1.103	Down
HOXB5	-0.29	-1.123	-2.103	Down
GGT3P	-0.125	-0.561	-1.053	Down
APBB1IP	-0.144	-0.586	-1.096	Down
TST	-0.435	-1.758	-3.378	Down
QARS	-0.335	-1.308	-2.487	Down
PYGL	-0.158	-0.84	-1.618	Down
FAM185A	-0.259	-1.184	-2.265	Down

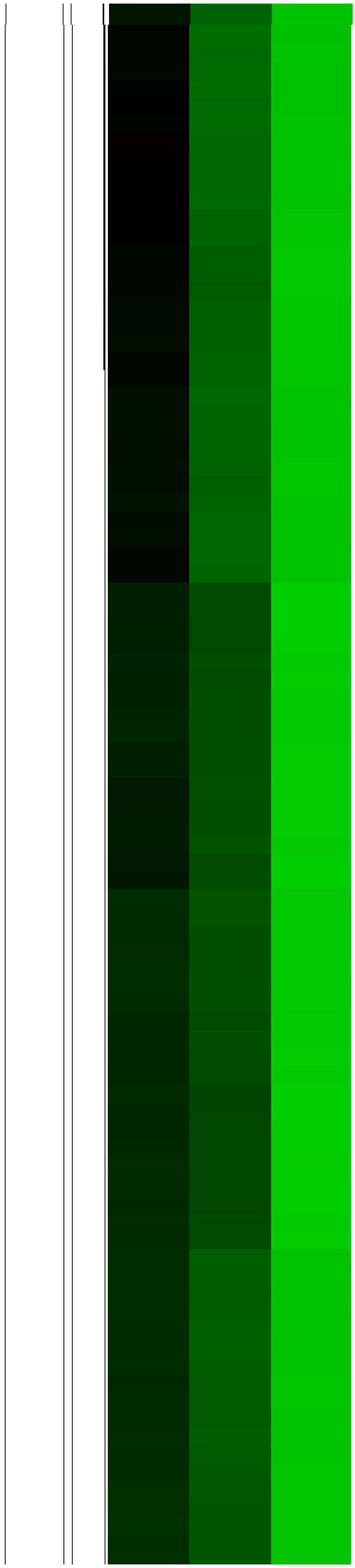

ANKRD30A	-0.186	-0.906	-1.757	Down
DIXDC1	-0.493	-2.069	-4.091	Down
ZMYM3	-0.06	-0.999	-1.789	Down
COL4A4	-0.031	-0.596	-1.065	Down
NEIL3	-0.061	-1.025	-1.904	Down
LANCL1	-0.075	-0.816	-1.506	Down
CCDC52	-0.016	-0.737	-1.329	Down
BACE1	-0.011	-0.635	-1.149	Down
NETO2	-0.018	-0.92	-1.701	Down
LOC100289097	0.027	-0.564	-1.059	Down
SSBP4	0.014	-0.852	-1.635	Down
TSHZ1	0.006	-0.732	-1.396	Down
SPATA17	0.006	-0.578	-1.073	Down
SAMD11	0	-0.633	-1.259	Down
MAF1	0	-0.607	-1.204	Down
C10orf75	-0.052	-0.645	-1.362	Down
BFSP1	-0.101	-1.19	-2.561	Down
SCARA5	-0.284	-2.588	-5.719	Down
FGA	-0.056	-0.516	-1.058	Down
ADCK2	-0.106	-1.009	-2.085	Down
TREX1	-0.158	-1.156	-2.364	Down
RRBP1	-0.037	-0.527	-1.047	Down
LOC147727	-0.064	-0.85	-1.698	Down
NOL3	-0.095	-0.579	-1.103	Down
GGTLC2	-0.101	-0.631	-1.217	Down
LOC100128501	-0.092	-0.593	-1.173	Down
ABCA7	-0.145	-1.056	-2.066	Down
PHF14	-0.123	-0.762	-1.524	Down
FUT8	-0.207	-1.202	-2.471	Down
AKR1C1	-0.12	-0.626	-1.248	Down
SPSB2	-0.167	-1.482	-2.824	Down
SIPA1L2	-0.075	-0.594	-1.118	Down
LTA4H	-0.077	-0.864	-1.645	Down
LENG9	-0.069	-0.76	-1.459	Down
RTKN	-0.407	-0.982	-2.759	Down
ALKBH2	-0.287	-0.678	-1.866	Down
TMEM116	-0.277	-0.638	-1.775	Down
EBAG9	-0.28	-0.618	-1.766	Down
CBR1	-0.467	-0.999	-2.645	Down
C20orf141	-0.383	-0.829	-2.244	Down
MLKL	-0.303	-0.719	-1.861	Down
IFT172	-0.319	-0.703	-1.836	Down
SPATA21	-0.241	-0.501	-1.311	Down
LOC653391	-0.257	-0.616	-1.629	Down
ALX1	-0.312	-0.776	-2.031	Down
FAM69B	-0.206	-0.635	-1.617	Down
CCR7	-0.586	-1.721	-4.475	Down
C7orf68	-0.349	-0.97	-2.506	Down
C8orf40	-0.234	-0.682	-1.7	Down
IER5L	-0.154	-0.454	-1.234	Down
C1orf131	-0.189	-0.602	-1.617	Down
HMBS	-0.46	-0.856	-2.063	Down
ALDH3A2	-0.311	-0.585	-1.445	Down
MAGEH1	-0.794	-1.486	-3.767	Down
C5orf58	-0.904	-1.723	-4.385	Down
LOC100290344	-0.471	-0.828	-2.159	Down
PCIF1	-0.231	-0.404	-1.022	Down
MRPL17	-0.345	-0.631	-1.59	Down
LOC100133008	-0.396	-0.736	-1.976	Down
HGD	-0.693	-1.352	-3.532	Down
WNK1	-0.257	-0.496	-1.349	Down
TBC1D16	-0.484	-0.909	-2.443	Down
LOC153684	-0.505	-0.798	-2.423	Down
ETV6	-0.411	-0.695	-2.113	Down
FZD3	-0.267	-0.495	-1.443	Down
C8orf55	-0.431	-0.756	-2.192	Down
KRTAP19-2	-0.416	-0.704	-2.035	Down
ANKRD36B	-0.441	-0.71	-2.101	Down
HOXC4	-0.528	-0.917	-2.657	Down
ZNF618	-0.43	-0.768	-2.12	Down
C3orf1	-0.405	-0.686	-1.924	Down
VPS26B	-0.449	-0.946	-1.958	Down
SYT17	-0.925	-1.903	-4.023	Down
SFXN3	-0.23	-0.474	-1.022	Down
LOC100293090	-0.504	-1.047	-2.204	Down
KRCC1	-0.814	-1.791	-3.642	Down
CSRP2BP	-1.357	-3.055	-6.16	Down
TRIM45	-0.702	-1.509	-3.099	Down
GOLGA5	-0.204	-0.46	-1.002	Down
CYB5B	-0.604	-1.292	-2.82	Down
LOC390282	-0.338	-0.734	-1.57	Down
C3orf31	-0.724	-1.615	-3.408	Down
QDPR	-0.443	-0.943	-2.029	Down
LPIN3	-0.313	-0.66	-1.459	Down
KIAA0485	-0.682	-1.244	-2.803	Down
GOLGA3	-0.356	-0.631	-1.456	Down
ZNF580	-0.269	-0.464	-1.077	Down
TBCK	-0.618	-1.172	-2.669	Down

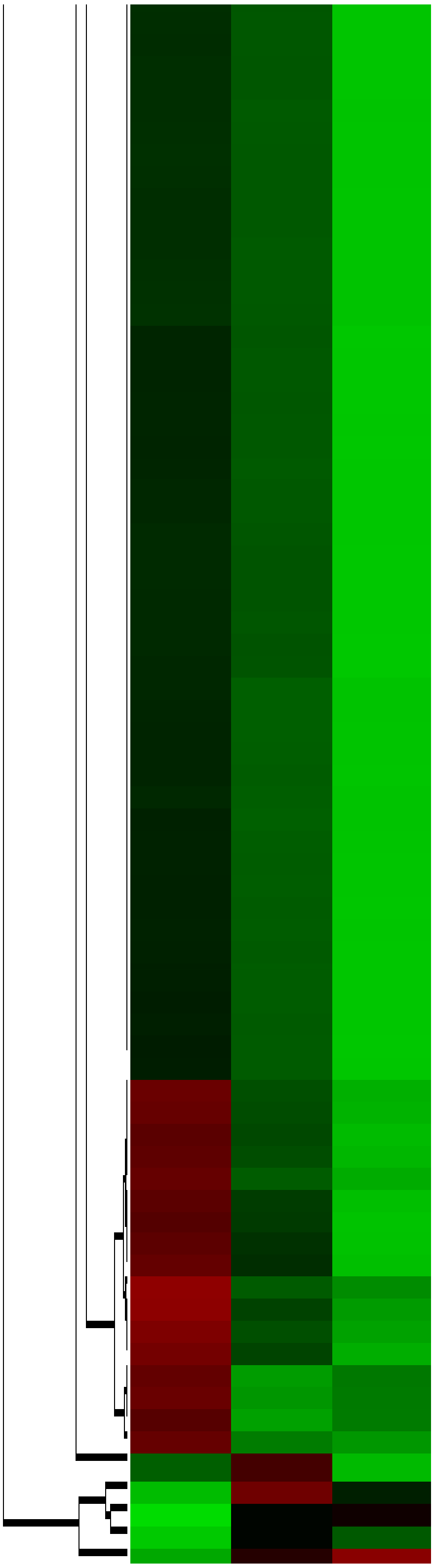

NEURL	-0.544	-0.997	-2.29	Down
RTN4IP1	-0.469	-0.896	-2.026	Down
GEMIN6	-0.595	-1.172	-2.641	Down
DKFZp566H0824	-0.492	-0.903	-2.071	Down
C9orf75	-0.339	-0.625	-1.435	Down
HSPA12A	-0.281	-0.545	-1.179	Down
hCG_1659830	-0.6	-1.125	-2.478	Down
EHMT2	-0.867	-1.586	-3.499	Down
DIRC1	-0.384	-0.711	-1.581	Down
C14orf143	-0.329	-0.64	-1.421	Down
ABHD15	-0.415	-0.795	-1.764	Down
SIX1	-0.337	-0.649	-1.414	Down
SAC3D1	-0.638	-1.192	-2.599	Down
MUTED	-0.31	-0.56	-1.228	Down
SNX17	-0.331	-0.575	-1.27	Down
PLSCR1	-0.193	-0.449	-1.033	Down
LOC100288578	-0.427	-1.009	-2.267	Down
LOC100288418	-0.696	-1.695	-3.834	Down
C21orf33	-0.21	-0.493	-1.125	Down
RILPL2	-0.417	-0.977	-2.19	Down
R3HDM2	-0.229	-0.558	-1.263	Down
TGIF1	-0.269	-0.642	-1.412	Down
RFX5	-0.394	-0.888	-2.004	Down
DUSP19	-0.335	-0.742	-1.666	Down
MAB21L2	-0.442	-0.897	-2.072	Down
LCE1E	-0.224	-0.447	-1.053	Down
ZNF512B	-0.348	-0.684	-1.617	Down
NUDT22	-0.375	-0.757	-1.794	Down
MIIP	-0.545	-1.121	-2.591	Down
DCLRE1A	-0.306	-0.626	-1.492	Down
ATOH8	-0.496	-1.079	-2.546	Down
LOC285696	-0.56	-1.355	-2.785	Down
HDGF2	-0.38	-0.979	-1.996	Down
SLC24A6	-0.531	-1.397	-2.882	Down
PYCARD	-0.457	-1.193	-2.478	Down
LOC100131829	-0.451	-1.156	-2.455	Down
WDR41	-0.22	-0.52	-1.072	Down
C21orf63	-0.228	-0.652	-1.319	Down
C14orf80	-0.258	-0.704	-1.479	Down
ARPC1B	-0.205	-0.551	-1.179	Down
ZDHHC14	-0.195	-0.549	-1.154	Down
C17orf75	-0.214	-0.585	-1.27	Down
C16orf59	-0.485	-1.306	-2.79	Down
HDDC3	-0.421	-1.157	-2.53	Down
C18orf22	-0.294	-0.852	-1.835	Down
C14orf93	-0.649	-2.005	-4.318	Down
ANGEL2	-0.227	-0.649	-1.421	Down
NAE1	-0.161	-0.523	-1.138	Down
LOC283352	-0.326	-0.994	-2.172	Down
SR140	0.617	-0.459	-1.024	
GAL3ST1	0.641	-0.478	-1.121	
HIRIP3	0.617	-0.489	-1.274	
BDKRB1	0.685	-0.56	-1.33	
C1orf130	0.683	-0.623	-1.162	
LOC126987	0.586	-0.386	-1.22	
HOXD8	0.705	-0.487	-1.637	
NCOA7	0.754	-0.406	-1.588	
GALNTL6	0.527	-0.237	-1	
NOD1	1.019	-0.652	-1.002	
PAN2	1.029	-0.48	-1.129	
SLC4A4	0.846	-0.53	-1.09	
PRDXDD1P	0.834	-0.49	-1.247	
PDGFD	0.632	-1.001	-0.768	
CYTIP	0.829	-1.19	-0.964	
SERPINB3	0.619	-1.156	-0.889	
RALGAPA2	0.705	-0.862	-1.046	
KIAA1107	-0.587	0.418	-1.147	
ATP1A2	-1.052	0.622	-0.176	
NCDN	-1.077	-0.028	0.075	
LOC644578	-1.048	-0.028	-0.467	
CPEB1	-1.265	0.282	1.034	

Supplementary Table 3. Summary of reaction conditions and primer sequences for semi-qPCR.

Genes	Primers	Sequence (5'→3')	Tm(°C)	Cycles	Size (bp)
FOSB	Sense<br>Antisense	ACCCTCTGCCGAGTCTCAAT<br>GAAGGAACCGGGCATTTC	60	30	109
IL8	Sense<br>Antisense	CAAGAGCCAGGAAGAAACCA<br>AGCACTCCTTGGCAAAACTG	60	30	137
MAFF	Sense<br>Antisense	AGACGCGCGTGTGTGA<br>CTGGATAGGGGATCCACAGA	60	30	100
DDIT3	Sense<br>Antisense	CCAAAATCAGAGCTGGAACC<br>CCATCTCTGCAGTTGGATCA	60	30	115
SERPINE1	Sense<br>Antisense	ACAACAGGAGGAGAAACCCA<br>AGCTCCTTGTACAGATGCCG	60	30	101
CSRP2BP	Sense<br>Antisense	GGAGCCAAAGAAGGAGGAAT<br>GTCGAAATCCCTTCCTGGAC	60	30	124
SCARA5	Sense<br>Antisense	TCTTCCTGATTCTTGTGGGC<br>AAGTCCCGGAAGCTCTCATT	60	30	118
HOXB13	Sense<br>Antisense	GTTGCCAGGGAGAACAGAAC<br>GCTGTACGGAATGCGTTTCT	60	30	125
FOXS1	Sense<br>Antisense	GACAACCACACAGAAAGCCA<br>GAGTAAATCCCAAGAGGCCC	60	30	105
DACT2	Sense<br>Antisense	GGCAACATCATATCCCCATC<br>AGGCCTGTCCAGTAGAAGCA	60	30	192
β-Actin	Sense<br>Antisense	CTGGAGAAGAGCTACGAGCTGC<br>CTAGAAGCATTTGCGGTGGACG	60	30	423
GAPDH	Sense<br>Antisense	AAGGCTGAGAACGGGAAG<br>GGACTCCACGACGTACTC	60	30	114

Supplementary Table 4. Full list of GO terms (FDR<0.01) associated with the Up- and Down-patterns by EEDS treatment in A549 cells.

GO ID	GO terms	*p*-value*	FDR**
**Down-pattern**			
GO:0051188	Cofactor biosynthetic process	9.26E-08	2.57E-04
GO:0051186	Cofactor metabolic process	1.79E-07	2.49E-04
GO:0018130	Heterocycle biosynthetic process	8.03E-07	7.42E-04
GO:0044271	Nitrogen compound biosynthetic process	6.15E-06	4.25E-03
GO:0006399	tRNA metabolic process	1.65E-05	9.12E-03
**Up-pattern**			
GO:0045449	Regulation of transcription	4.21E-26	1.55E-22
GO:0006350	Transcription	3.75E-21	6.91E-18
GO:0006355	Regulation of transcription, DNA-dependent	3.11E-14	3.82E-11
GO:0051252	Regulation of RNA metabolic process	3.78E-14	3.49E-11
GO:0006357	Regulation of transcription from RNA polymerase II promoter	2.05E-13	1.51E-10
GO:0042325	Regulation of phosphorylation	2.03E-11	1.25E-08
GO:0051173	Positive regulation of nitrogen compound metabolic process	2.39E-11	1.26E-08
GO:0045859	Regulation of protein kinase activity	2.44E-11	1.12E-08
GO:0019220	Regulation of phosphate metabolic process	2.68E-11	1.10E-08
GO:0051174	Regulation of phosphorus metabolic process	2.68E-11	1.10E-08
GO:0043549	Regulation of kinase activity	1.23E-10	4.54E-08
GO:0045935	Positive regulation of nucleobase, nucleoside, nucleotide and nucleic acid metabolic process	1.80E-10	6.04E-08
GO:0009891	Positive regulation of biosynthetic process	2.68E-10	8.23E-08
GO:0051338	Regulation of transferase activity	3.18E-10	9.02E-08
GO:0012501	Programmed cell death	3.42E-10	9.00E-08
GO:0042981	Regulation of apoptosis	4.67E-10	1.15E-07
GO:0031328	Positive regulation of cellular biosynthetic process	4.89E-10	1.13E-07
GO:0010558	Negative regulation of macromolecule biosynthetic process	7.90E-10	1.71E-07
GO:0043067	Regulation of programmed cell death	8.82E-10	1.81E-07
GO:0009890	Negative regulation of biosynthetic process	9.00E-10	1.75E-07
GO:0010941	Regulation of cell death	1.11E-09	2.04E-07
GO:0010557	Positive regulation of macromolecule biosynthetic process	1.29E-09	2.26E-07
GO:0006915	Apoptosis	1.49E-09	2.50E-07
GO:0016265	Death	1.91E-09	3.06E-07
GO:0008219	Cell death	2.52E-09	3.88E-07

GO:0051254	Positive regulation of RNA metabolic process	2.91E-09	4.30E-07
GO:0031327	Negative regulation of cellular biosynthetic process	6.29E-09	8.92E-07
GO:0010629	Negative regulation of gene expression	6.81E-09	9.29E-07
GO:0045941	Positive regulation of transcription	6.87E-09	9.04E-07
GO:0010604	Positive regulation of macromolecule metabolic process	6.87E-09	8.73E-07
GO:0010628	Positive regulation of gene expression	7.07E-09	8.69E-07
GO:0045893	Positive regulation of transcription, DNA-dependent	9.74E-09	1.16E-06
GO:0016481	Negative regulation of transcription	2.21E-08	2.54E-06
GO:0043066	Negative regulation of apoptosis	4.16E-08	4.64E-06
GO:0043069	Negative regulation of programmed cell death	7.16E-08	7.76E-06
GO:0060548	Negative regulation of cell death	7.93E-08	8.35E-06
GO:0010627	Regulation of protein kinase cascade	1.81E-07	1.85E-05
GO:0051172	Negative regulation of nitrogen compound metabolic process	2.38E-07	2.37E-05
GO:0045934	Negative regulation of nucleobase, nucleoside, nucleotide and nucleic acid metabolic process	2.68E-07	2.60E-05
GO:0010605	Negative regulation of macromolecule metabolic process	3.36E-07	3.18E-05
GO:0045944	Positive regulation of transcription from RNA polymerase II promoter	5.41E-07	4.99E-05
GO:0033673	Negative regulation of kinase activity	6.15E-07	5.52E-05
GO:0006469	Negative regulation of protein kinase activity	1.24E-06	1.09E-04
GO:0045892	Negative regulation of transcription, DNA-dependent	1.47E-06	1.26E-04
GO:0043405	Regulation of MAP kinase activity	1.56E-06	1.31E-04
GO:0045860	Positive regulation of protein kinase activity	1.98E-06	1.62E-04
GO:0051348	Negative regulation of transferase activity	2.17E-06	1.74E-04
GO:0051253	Negative regulation of RNA metabolic process	2.56E-06	2.01E-04
GO:0033674	Positive regulation of kinase activity	5.05E-06	3.88E-04
GO:0051347	Positive regulation of transferase activity	5.77E-06	4.34E-04
GO:0010740	Positive regulation of protein kinase cascade	8.54E-06	6.29E-04
GO:0009967	Positive regulation of signal transduction	1.05E-05	7.61E-04
GO:0000122	Negative regulation of transcription from RNA polymerase II promoter	1.49E-05	1.05E-03
GO:0001932	Regulation of protein amino acid phosphorylation	1.63E-05	1.13E-03
GO:0006796	Phosphate metabolic process	2.08E-05	1.42E-03
GO:0006793	Phosphorus metabolic process	2.08E-05	1.42E-03
GO:0007243	Protein kinase cascade	2.12E-05	1.41E-03
GO:0010647	Positive regulation of cell communication	2.36E-05	1.55E-03
GO:0007242	Intracellular signaling cascade	2.58E-05	1.66E-03
GO:0042127	Regulation of cell proliferation	2.70E-05	1.71E-03
GO:0000165	MAPKKK cascade	2.77E-05	1.73E-03
GO:0051726	Regulation of cell cycle	2.79E-05	1.71E-03

GO:0044093	Positive regulation of molecular function	3.03E-05	1.82E-03
GO:0010033	Response to organic substance	3.50E-05	2.08E-03
GO:0033554	Cellular response to stress	3.70E-05	2.16E-03
GO:0032268	Regulation of cellular protein metabolic process	3.87E-05	2.22E-03
GO:0050920	Regulation of chemotaxis	3.99E-05	2.26E-03
GO:0008285	Negative regulation of cell proliferation	4.36E-05	2.43E-03
GO:0006916	Anti-apoptosis	4.54E-05	2.49E-03
GO:0000079	Regulation of cyclin-dependent protein kinase activity	4.63E-05	2.50E-03
GO:0032774	RNA biosynthetic process	4.71E-05	2.51E-03
GO:0006468	Protein amino acid phosphorylation	4.72E-05	2.48E-03
GO:0043122	Regulation of I-kappab kinase/NF-kappab cascade	4.93E-05	2.55E-03
GO:0006402	mRNA catabolic process	5.60E-05	2.86E-03
GO:0016071	mRNA metabolic process	5.90E-05	2.97E-03
GO:0006366	Transcription from RNA polymerase II promoter	6.79E-05	3.37E-03
GO:0044265	Cellular macromolecule catabolic process	6.84E-05	3.35E-03
GO:0006351	Transcription, DNA-dependent	6.90E-05	3.34E-03
GO:0043065	Positive regulation of apoptosis	7.22E-05	3.45E-03
GO:0043068	Positive regulation of programmed cell death	8.84E-05	4.17E-03
GO:0010942	Positive regulation of cell death	1.01E-04	4.69E-03
GO:0040017	Positive regulation of locomotion	1.08E-04	4.98E-03
GO:0051341	Regulation of oxidoreductase activity	1.17E-04	5.32E-03
GO:0050921	Positive regulation of chemotaxis	1.19E-04	5.31E-03
GO:0000956	Nuclear-transcribed mrna catabolic process	1.41E-04	6.25E-03
GO:0045768	Positive regulation of anti-apoptosis	1.64E-04	7.16E-03
GO:0032570	Response to progesterone stimulus	1.76E-04	7.58E-03
GO:0032768	Regulation of monooxygenase activity	1.79E-04	7.62E-03
GO:0043408	Regulation of MAPKKK cascade	1.90E-04	8.00E-03
GO:0030335	Positive regulation of cell migration	2.39E-04	9.96E-03

* *p*-values were calculated using Fischer's test.

** FDR corrections were calculated using the Benjamini-Hochberg procedure [11].

Supplementary Figure 1

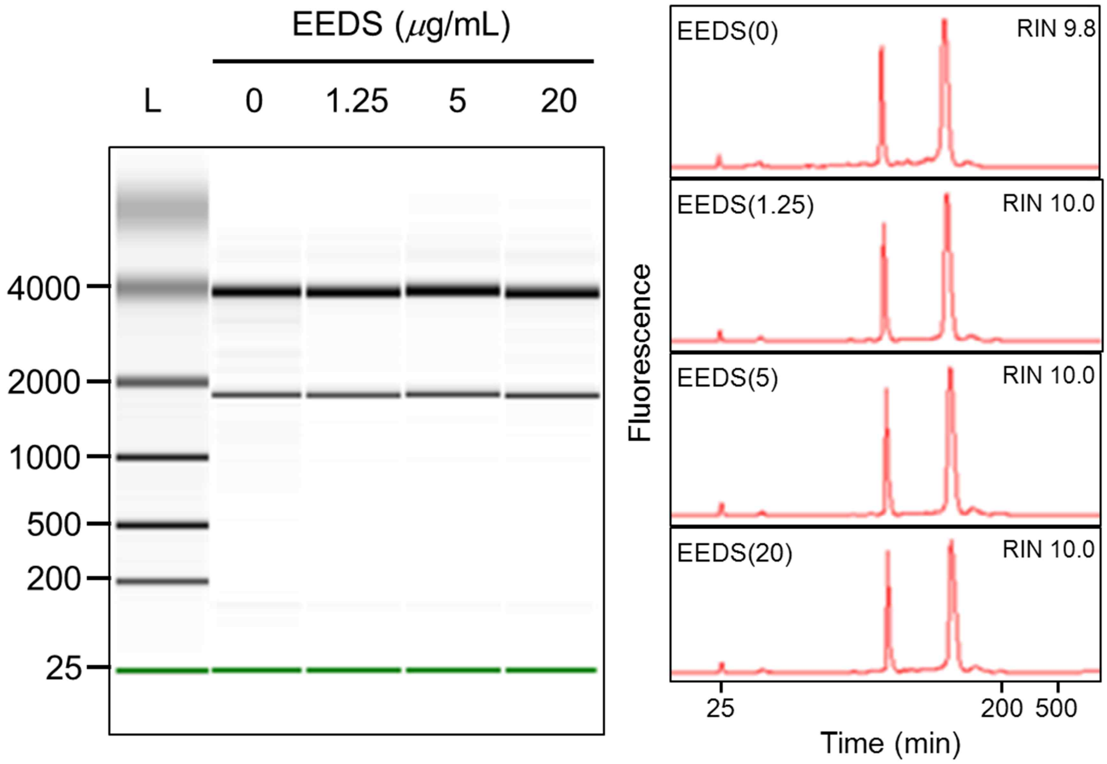

Supplementary Figure 2

Enriched pathways in Down-pattern

Base excision repair

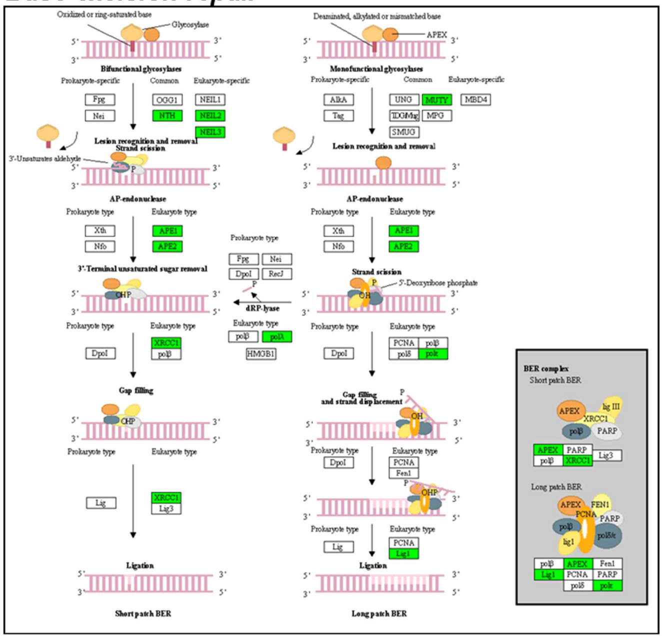

Pentose phosphate pathway

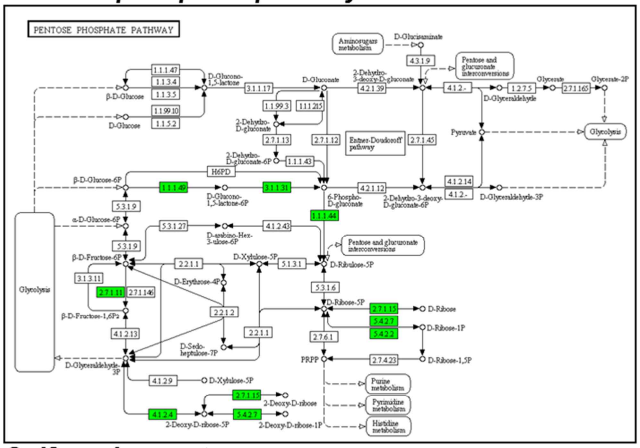

Sulfur relay system

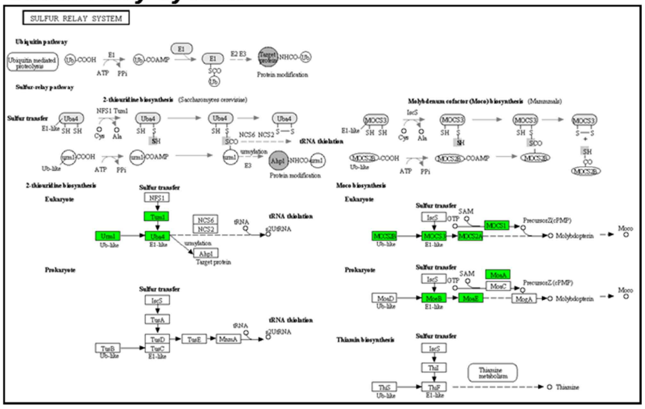

Supplementary Figure 2 (continued)

MAPK signaling pathway

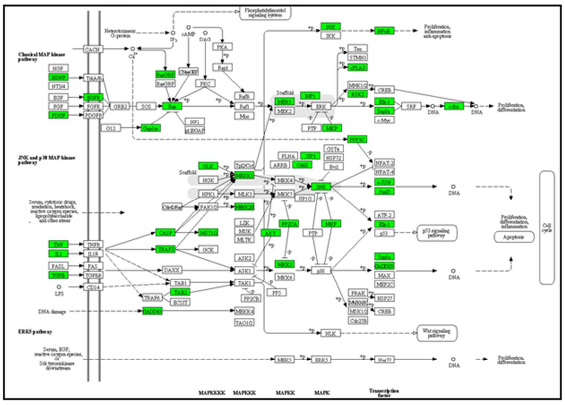

Apoptosis

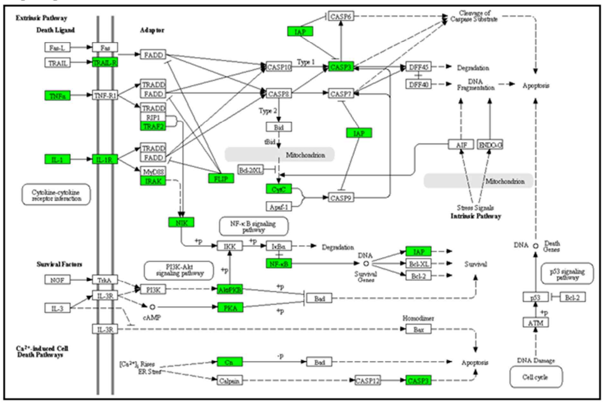

p53 signaling pathway

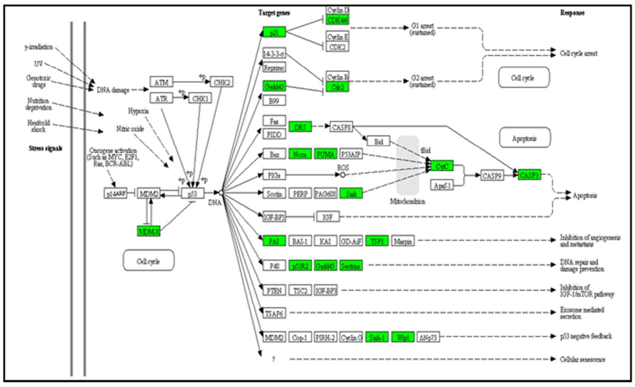

TGF-beta signaling pathway

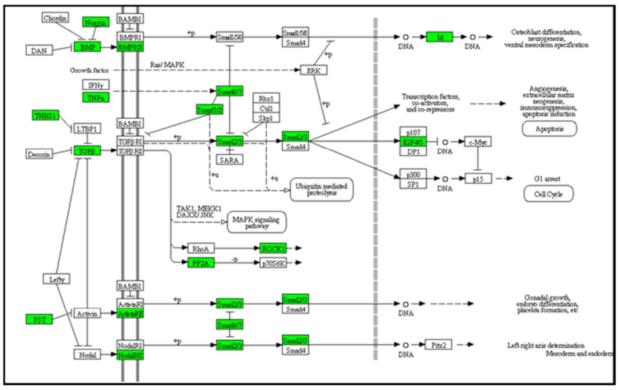

Supplementary Figure 2 (continued)

Enriched pathways in Up-pattern

Cytokine-cytokine receptor interaction

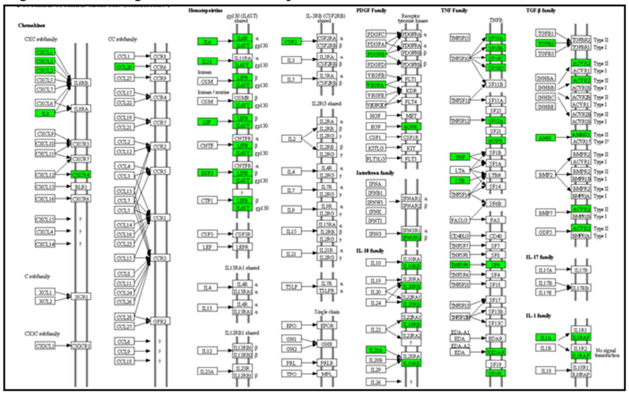

NOD-like receptor signaling pathway

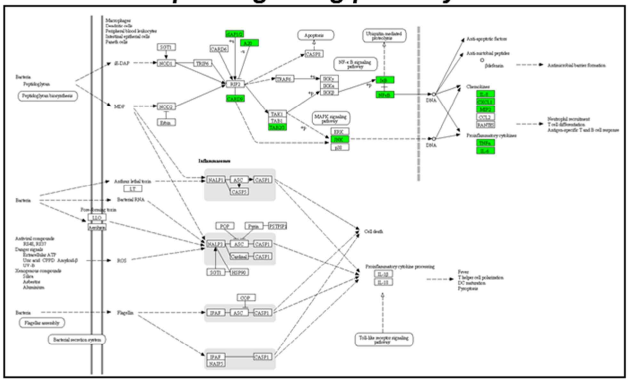

Circadian rhythm

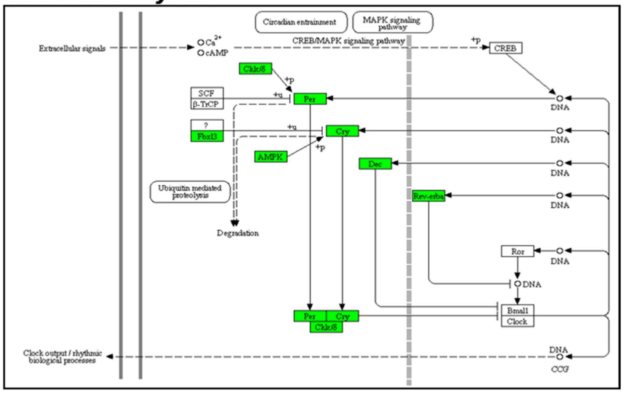

Adipocytokine signaling pathway

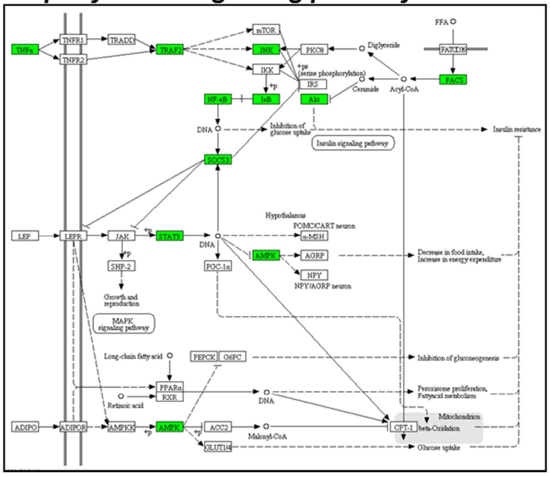

Supplementary Figure 2 (continued)

Enriched pathways in Up-pattern

Osteoclast differentiation

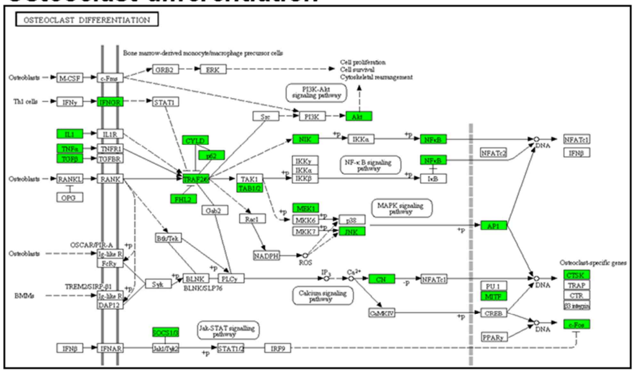

NF-kappa B signaling pathway

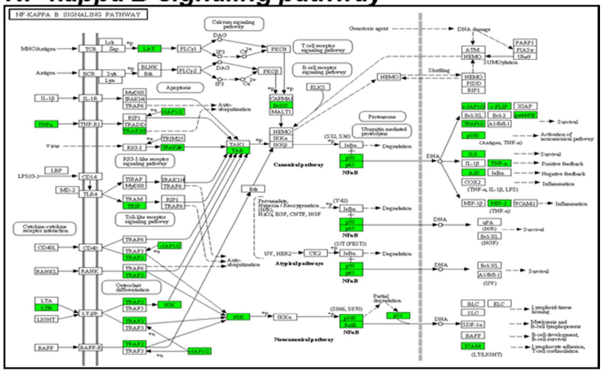

Transcriptional misregulation in cancer

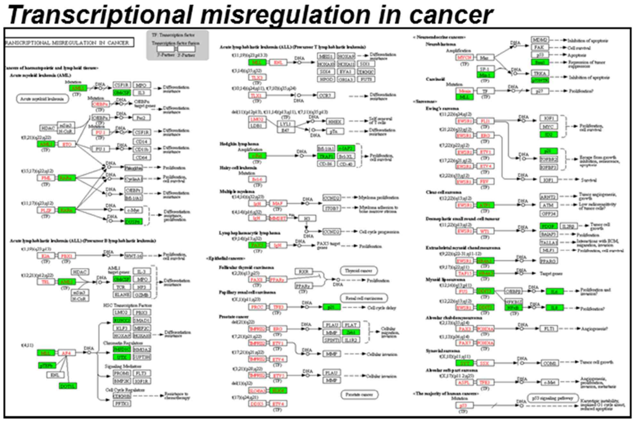

Salmonella infection

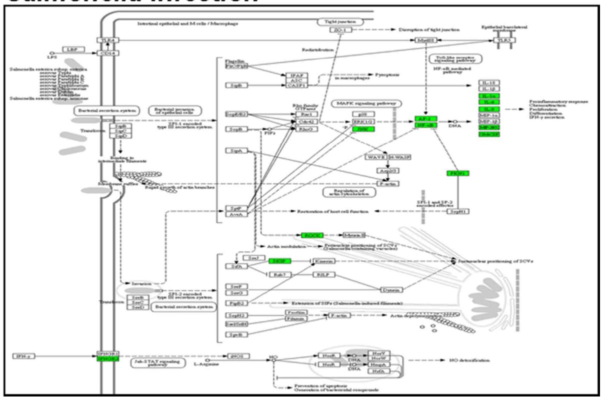

Supplementary Figure 2 (continued)

Enriched pathways in Up-pattern

Legionellosis

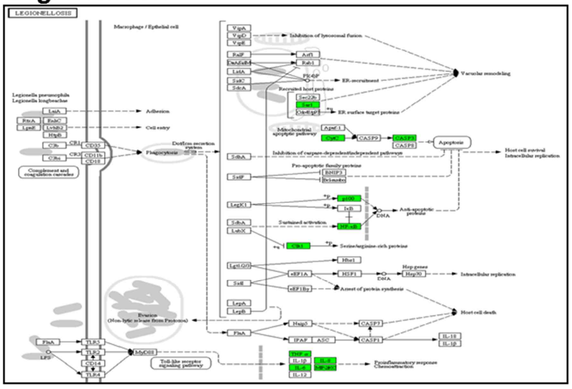

GnRH signaling pathway

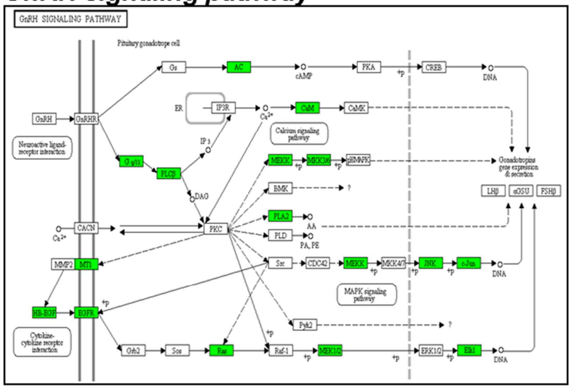

ErbB signaling pathway

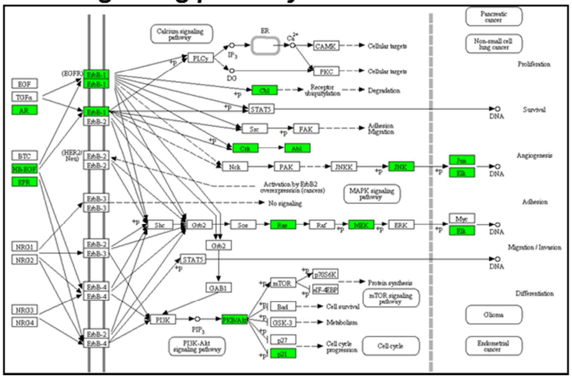

Epithelial cell signaling in Helicobacter pylori infection

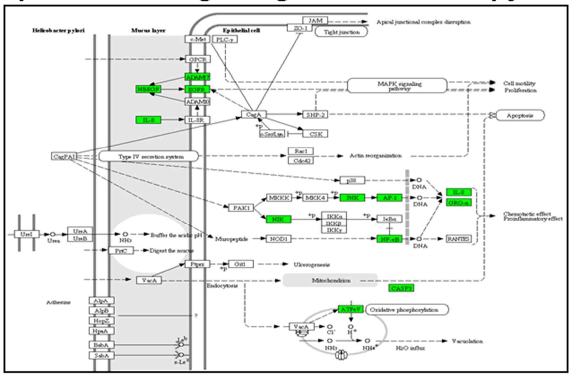

Supplementary Figure 3

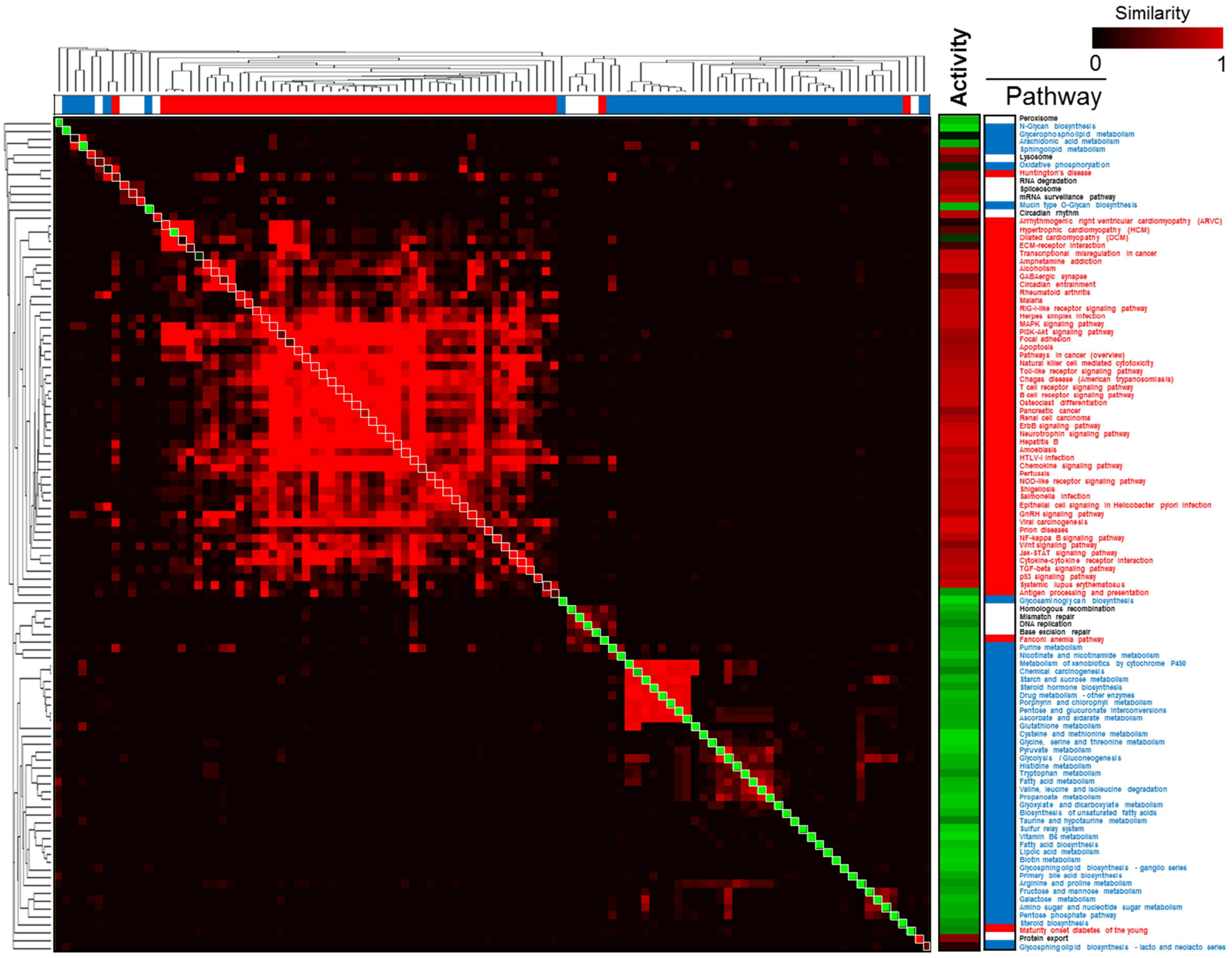

Supplement: Supplementary file 1 — The integrities of RNA samples used for gene expression analyses were determined by performing electropherogram (Figure S1 and Table S1). The positions of significant genes in each enriched pathways were depicted in Figure S2. The activities of pathways were clustered based on similarity of pathways in Figure S3. Full lists of genes were summarized in Table S2 with expression ratios. Expression of representative genes was confirmed by semiquantitative PCR. The reaction conditions and primer sequences were summarized in Table S3. The enriched GO terms were listed in Table S4. [file 584604.f1.pdf]
